# Remote 1,5-Difunctionalization of Alkenes via a Sulfinyl-Smiles Rearrangement and Vinylcyclopropane Ring-Opening Cascade

**DOI:** 10.1021/jacsau.6c00241

**Published:** 2026-05-21

**Authors:** Yawen Hu, Klorenta Nitaj, Cristina Nevado

**Affiliations:** Department of Chemistry, 27217 University of Zurich, Winterthurerstrasse 190, CH 8057 Zurich, Switzerland

**Keywords:** sulfinyl-Smiles rearrangement, vinylcyclopropanes, asymmetric remote difunctionalization, photoredox catalysis

## Abstract

The enantioselective remote difunctionalization of alkenes via radical pathways remains a major challenge due to limited stereocontrol in open-shell processes. We report a photoredox-catalyzed asymmetric remote 1,5-difunctionalization of alkenes that combines the strain-release ring opening of vinylcyclopropanes with a stereocontrolled sulfinyl-Smiles rearrangement. This multistep radical cascade bypasses kinetically disfavored hydrogen atom transfer pathways and enables precise site selectivity with excellent enantioselectivity. Both sulfur- and carbon-centered radicals are accommodated, providing efficient access to enantioenriched α-aryl γ,δ-unsaturated amides and derivatives thereof under mild conditions.

## Graphical abstract

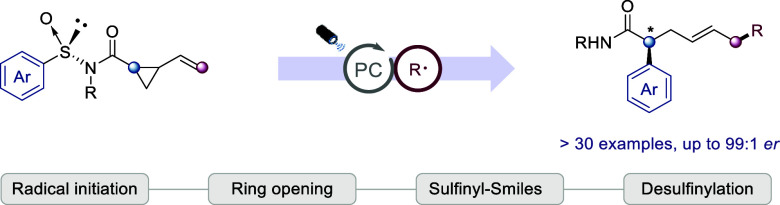

Alkene difunctionalization enables the simultaneous introduction of two distinct functional groups across the C=C π system, thereby rapidly increasing the molecular complexity.^1–3^ Substantial progress has been made in the 1,2-difunctionalization of olefins using both two- and one-electron-mediated processes.^4–12^ In sharp contrast, strategies that allow for the installation of functional groups at distal positions from the initial π-reactive site remain comparatively underdeveloped, despite being highly desired. Transition-metal-mediated “chain-walking” methods^13–15^ have played a prominent role in this arena recently, complemented by radical-mediated strategies.^16–20^ Despite these advances, imparting absolute stereocontrol in radical-mediated remote difunctionalizations of alkenes persist as a formidable challenge due to the high reactivity and transient nature of the open-shell intermediates involved, so that, up to now, only a handful of examples have been reported.^21–23^ A recent contribution from our group^24^ showcased an asymmetric remote 1,6- and 1,7-dicarbofunctionalization of alkenes combining a 1,5- or 1,6-HAT with a sulfinyl-Smiles rearrangement. In this transformation, chiral arylsulfinylamides^25–30^ enabled smooth chirality transfer with concomitant self-immolation of the chiral sulfinyl auxiliary, affording a broad array of chiral amides with excellent levels of absolute stereocontrol and site selectivity. Nonetheless, migration via the HAT process is kinetically disfavored for transformations occurring at sites five carbon atoms away from the initial reactive center, and despite our best efforts, 1,5-asymmetric dicarbofunctionalizations using this concept could not be realized.^31^

Vinylcyclopropanes (VCPs) are privileged carbon synthons characterized by their distinctive structural features, including a strained three-membered ring and an appended alkene moiety. Extensively studied in the well-known vinylcyclopropane–cyclopentene rearrangement and cycloaddition reactions, more recently, their ring-opening reactivity has been widely exploited to construct linear or branched unsaturated frameworks via ionic pathways.^32–36^ Within the radical regime, VCPs are frequently utilized as radical probes to gain mechanistic insight into alkene difunctionalizations. However, a handful of examples have recently unraveled their promising role for chirality introduction at distal sites, thereby unlocking asymmetric remote 1,5-difunctionalizations (Scheme 1A). The first was disclosed by Wang and co-workers, who achieved enantioselective 1,5-cyanotrifluoromethylation of VCPs via chiral copper catalysis in the presence of Togni’s reagent and trimethylsilyl cyanide.^37^ Further, an iron-catalyzed asymmetric intramolecular dicarbofunctionalization of VCPs with alkyl halides and Grignard reagents was developed through a radical cyclization/ring-opening/cross-coupling sequence. The intermolecular variant was also demonstrated, albeit with only two examples reported and moderate enantioselectivity.^38^ A third approach, utilizing synergistic chiral phosphoric acid (CPA)/photoredox catalysis, enabled the synthesis of enantioenriched unnatural α-amino acid derivatives bearing two contiguous stereogenic centers.^39^ More recently, Wang’s group^40^ and ours^41^ independently reported a nickel-catalyzed asymmetric 1,5-difunctionalization of alkenes, delivering various remotely difunctionalized products with high enantioselectivity. Notwithstanding this significant progress, challenges persist with respect to scope diversity and achieving high stereocontrol for widespread synthetic applications.

**Scheme 1:**
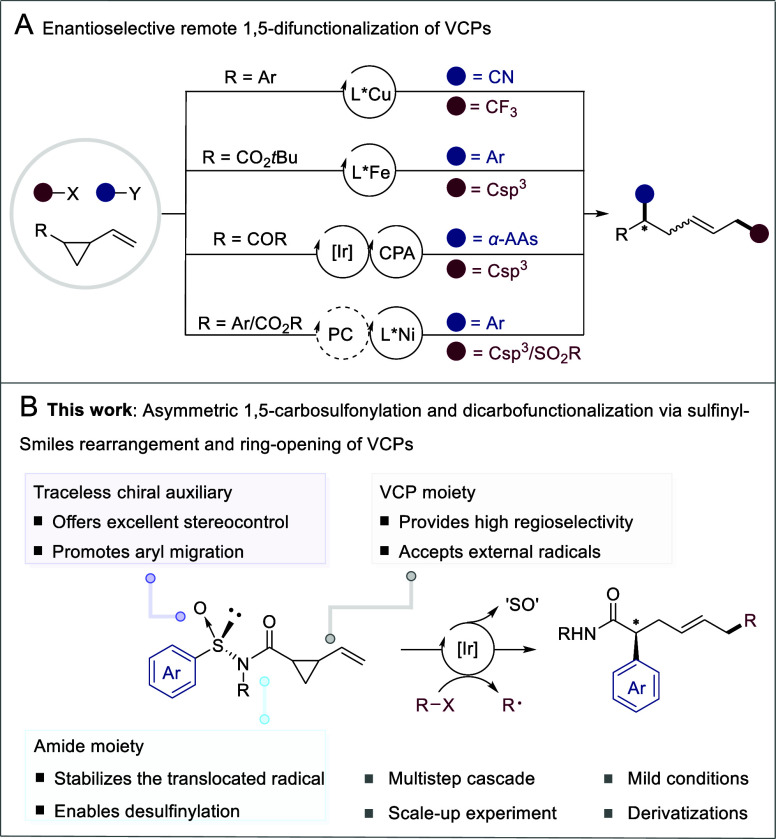
(A) State of the Art of Enantioselective Remote 1,5-Difunctionalization of VCPs; (B) This Work: Photoredox Asymmetric Remote 1,5-Carbosulfonylation and Dicarbofunctionalization of VCPs via Sulfinyl-Smiles Rearrangement and Ring-Opening Cascade

Herein, VCPs and a stereocontrolled sulfinyl-Smiles rearrangement are leveraged to enable a photoredox-catalyzed remote enantioselective 1,5-difunctionalization of alkenes (Scheme 1B). Compared to previous methods, this multistep radical cascade offers enhanced levels of stereocontrol and enables the incorporation of both sulfur- and carbon-centered radicals, thus facilitating the synthesis of a diverse variety of enantioenriched α-aryl γ,δ-unsaturated amides under mild conditions. Furthermore, this protocol is readily scalable, and the reaction products are efficiently derivatized into valuable building blocks for both synthetic and biologically relevant applications, demonstrating the effectiveness of chiral sulfinyl auxiliaries for controlling asymmetric radical transformations.

An *N*-benzyl-*N*-((*S*)-*p*-tolylsulfinyl)-2-vinylcyclopropane-1-carboxamide substrate **1.1** was synthesized to evaluate the feasibility of the proposed transformation. Upon exposure to 4-toluenesulfonyl chloride, Ir­(ppy)_3_ and K_3_PO_4_ under white LED irradiation for 20 h in acetonitrile (MeCN), the desired α-aryl-amide **2.1** could be obtained in 66% yield with an excellent 99:1 enantiomeric ratio (er) as shown in entry 1 of Scheme 2A.^42^ Subsequent examination of various photocatalysts such as Ir­(dtbbpy)­(ppy)_2_PF_6_, Ir­[(dF­(CF_3_)­ppy)_2_(dtppy)]­PF_6_, and Eosin Y disodium salt (entries 2–4 and Supporting Information) furnished **2.1** in lower yields compared to Ir­(ppy)_3_.^42^ Several solvents including MeCN/water mixtures, tetrahydrofuran (THF), dichloromethane (DCM), and acetone were then tested with acetone providing the product with an improved yield of 72% (entries 5–9 and Supporting Information).^42^ Extensive screening of both inorganic and organic bases in acetone (entries 10–13 and Supporting Information) revealed Na_2_HPO_4_ as the base of choice for this transformation.^40^ Reducing the amount of base to 1 equiv maintained the efficiency with **2.1** isolated in 76% yield and 99:1 er (entry 14). Lowering the amount of base to 0.5 equiv (entry 15) led to a slight drop in yield (74%), while the absence of base drastically reduced product formation to 37% (entry 16). Control experiments confirmed that both light and photocatalyst are essential for a fruitful outcome (Scheme 2B, entries 1–2). Additionally, a substantial decrease in yield was observed in the presence of radical inhibitors (entry 3 and Supporting Information).^42^ On the basis of these experimental results and previous reports,^24–30^ a catalytic cycle for this asymmetric remote difunctionalization of alkenes is proposed in Scheme 2C. Upon visible-light irradiation, the photocatalyst is excited to undergo single electron oxidation in the presence of radical precursors, releasing a reactive radical (R•). This undergoes addition to the terminal position of the double bond in starting material **1**. The resulting radical intermediate **I** fragments via spontaneous homolytic C–C bond cleavage driven by the release of ring strain to furnish a distal carbon-centered radical **II**. This newly formed intermediate **II** undergoes the sulfinyl-Smiles rearrangement, wherein the translocation of aryl groups occurs with high stereofidelity. The photocatalytic cycle is then closed by a desulfinylation event to deliver the remote difunctionalized product in high efficiency and enantioselectivity with the SO linker likely being released as bisulfite salts under the basic reaction conditions.^25,28,42^

**Scheme 2:**
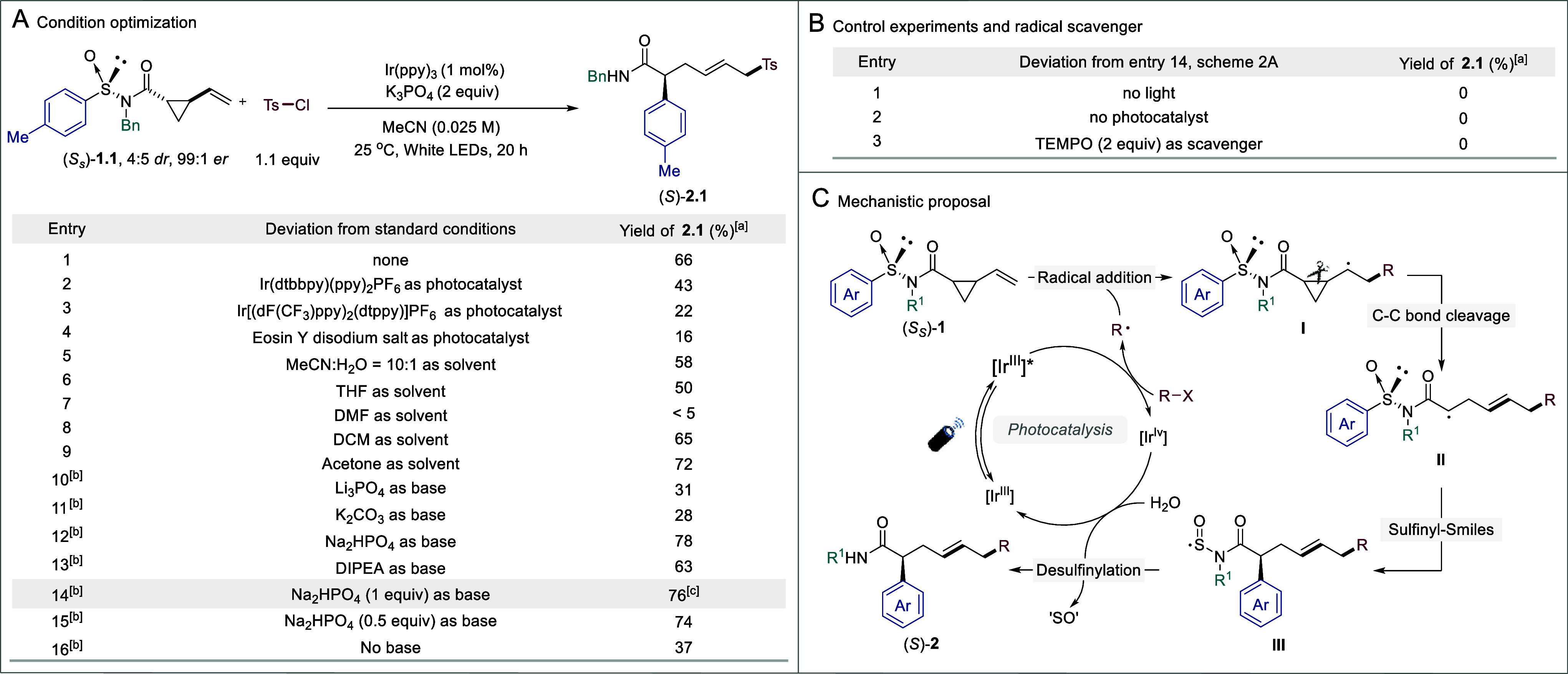
(A) Condition Optimization; (B) Control Experiment and Radical Scavenger Details; (C) Mechanistic Proposal

a Yield was determined by ^1^H NMR using mesitylene as the internal standard.
b Acetone was used instead of MeCN.
c Isolated yield.

With the optimized condition in hand, the generality of the reaction was then investigated (Table 1). First, we explored different aryl migrating groups tethered to the *S*-chiral auxiliary. Derivatives bearing either electron-donating (−Me, −OMe) or electron-withdrawing (−Br, –F) groups at the *para* position of the benzene ring yielded the corresponding amides (**2.1–2.4**) with excellent yields and enantiomeric ratios. Notably, the (*S*,*E*)-absolute configuration of the products was unambiguously assigned by X-ray diffraction analysis of single crystals of compound **2.1**. The stereospecific nature of this transformation was confirmed by the identical yield and enantiomeric ratio obtained when the (*R*_*s*_)-diastereomer of *p*-Tol-substituted substrate **1.1** was subjected to the same reaction conditions (see Supporting Information).^42^ Sulfinylamides with *ortho*-substituents on the migrating arene were smoothly converted into the desired products with stereochemical information well preserved but with slightly diminished yields (**2.5–2.6**) compared to their *para*-substituted analogs. The transposition of 1-naphthyl and 2-naphthyl groups was well tolerated, delivering products **2.7** and **2.8** in 49 and 51% yield with 88:12 and 92:8 er, respectively. The slightly reduced enantioenrichment observed in **2.7**, which was obtained from a starting material with 91:9 er, likely results from steric interactions between the naphthyl moiety and the alkyl chain during the transposition process. A 2-thiophenyl moiety was effectively incorporated into product **2.9** with 70% yield and a 95:5 er value. Substrates with benzylic or alkyl-substituted nitrogens were subsequently examined, and the corresponding products **2.10–2.12** were successfully isolated in excellent yields and levels of stereocontrol. Next, we turned our attention to variations of vinyl cyclopropane scaffolds. A substrate featuring an isopropenyl group was well accommodated under the standard reaction conditions, furnishing the desired remote functionalized product **2.13** in good yield and high enantioselectivity with a 2:1 *E*/*Z* ratio. Gratifyingly, a 2-methyl-substituted vinyl cyclopropane moiety was proven to be eligible for this transformation so that the corresponding enantioenriched amide **2.14** was obtained in a 64% yield with maintained enantioselectivity (90:10 er). Next, a diverse range of sulfonyl chlorides was explored as radical precursors. Aryl sulfonyl chlorides bearing *para*-substituents with varied electronic properties were viable partners for the radical addition process, delivering chiral amides **2.15–2.18** with excellent yields and enantioselectivities. The *meta*- and *ortho*-methyl-substituted phenyl sulfonyl chlorides were also compatible within this protocol, affording **2.19** and **2.20** in 78 and 74% yields, respectively, both with 99:1 er values. Moreover, other unsaturated systems such as 2-thiophenyl and styrene sulfonyl chlorides performed well under the reaction conditions, furnishing products **2.21** and **2.22** with excellent enantiomeric ratios (99:1 er). Alkyl sulfonyl motifs were also successfully installed into enantioenriched α-arylated amides **2.23–2.24** in moderate to excellent yields and 99:1 er values.

**Table 1:**
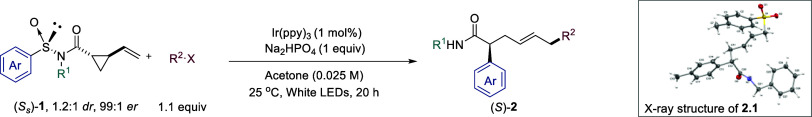

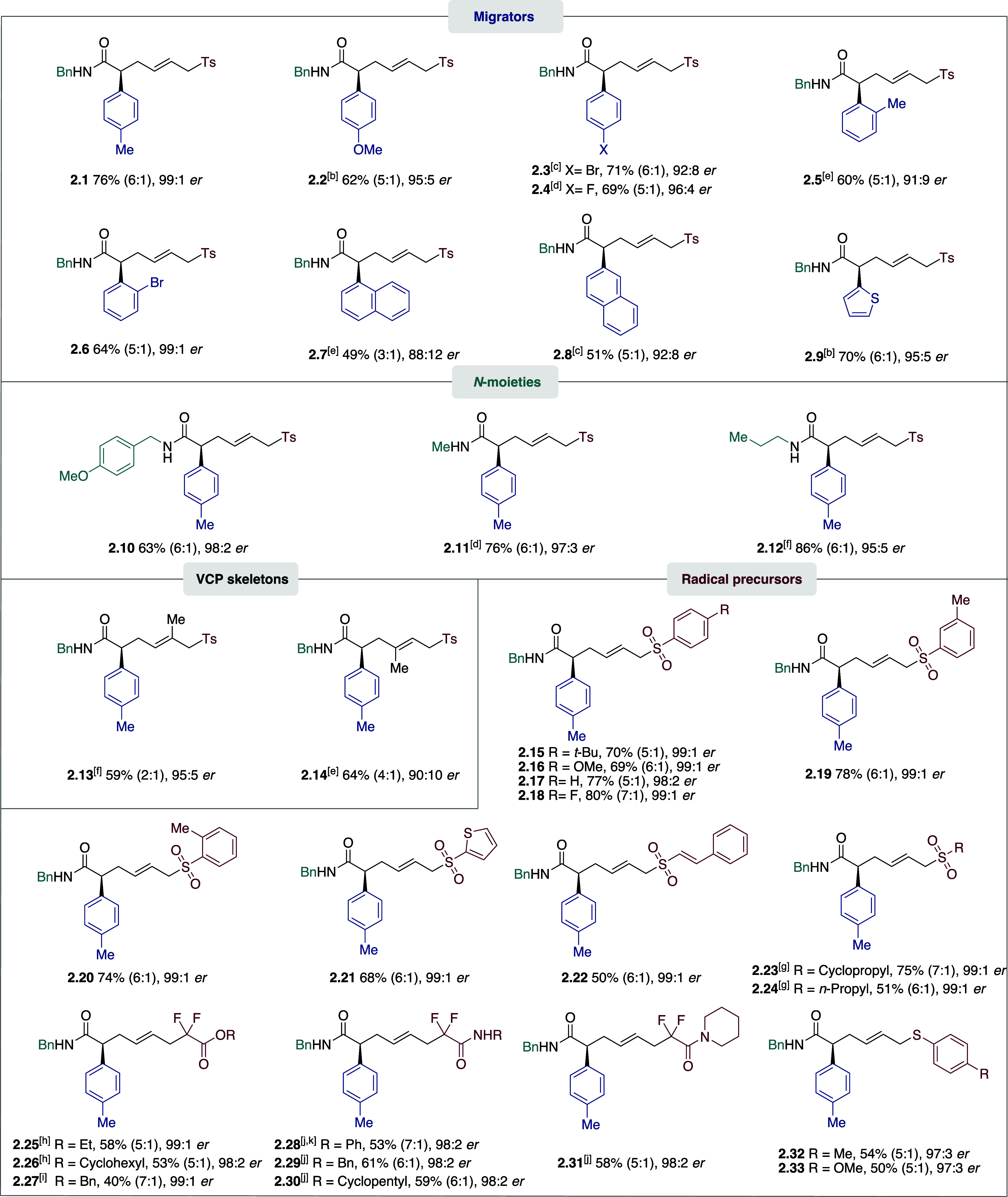
Substrate Scope^a^

a The er of starting material is 99:1, unless otherwise indicated. The ratios given in parentheses correspond to the *E*/*Z* alkene geometry.

b The er of the starting material is 96:4.

c The er of the starting material is 92:8.

d The er of the starting material is 97:3.

e The er of the starting material is 91:9.

f The er of the starting material is 95:5.

g A total of 2 equiv of alkyl sulfonyl chloride was used.

h Condition deviations from standard conditions: Ir­(ppy)_3_ (2 mol %), K_3_PO_4_ (1 equiv), EA (0.025 M), 35 °C.

i Condition deviations from standard conditions: Ir­(ppy)_3_ (2 mol %), K_3_PO_4_ (1 equiv), EA (0.025 M), 15 °C.

j The reactions were conducted under 427 nm irradiation with 4-CzIPN (2 mol %) and DIPEA (2 equiv) in EA (0.025 M) at 25 °C for 24 h.

k A 370 nm blue LED was used instead of 427 nm.

To expand the versatility of this transformation, we set out to investigate additional radical precursors. To our delight, a diverse array of 2-bromo-2,2-difluoroacetates as well as difluoroacetamides were also competent partners for this reaction under modified conditions.^27^ Ethyl and cyclohexyl ester bromides released corresponding radicals smoothly, affording amides **2.25** and **2.26** in 58 and 53% yields with 99:1 and 98:2 er, respectively, while the benzylic precursor delivered **2.27** with high stereoselectivity but in a diminished yield (40%). Furthermore, secondary as well as tertiary difluoroacetamides were well incorporated into the respective enantioenriched amides **2.28–2.31** in moderate yields with high enantiomeric ratios under blue (or in some cases ultraviolet) light irradiation. In addition, aromatic disulfides were compatible with the standard reaction conditions, furnishing amides **2.32** and **2.33** in moderate yields with 97:3 er values.

The synthetic utility of this asymmetric remote difunctionalization was further demonstrated through a scale-up experiment and subsequent derivatizations of the chiral amide products (Scheme 3). Our protocol operated efficiently on a 1.0 mmol scale (20-fold), affording the corresponding enantioenriched amides **2.1** in 77% yield with excellent enantioselectivity (98:2 er). Derivatizations of compounds **2.1**, **2.6**, and **2.10** were envisioned to rapidly access structural diversity. Thus, a Pd/C-catalyzed hydrogenation of **2.1** proceeded smoothly in a THF/methanol mixture at room temperature to deliver the fully saturated analog **3.1** in a quantitative yield with complete retention of stereochemical information (99:1 er). Interestingly, we found that in the presence of 15 equiv of *meta*-chloroperbenzoic acid (*m*-CPBA), upon double bond epoxidation and amide hydrolysis, compound **2.1** furnished diastereomeric lactones (*S*,*S*,*S*)- and (*S*,*R*,*R*)-**3.2** in an overall yield of 65% with enantiomeric ratios of 93:7 and 98:2 after recrystallization, respectively. Further, treatment of compound **2.10** with 2,3-dichloro-5,6-dicyano-1,4-benzoquinone (DDQ) in a DCM/water mixture led to efficient cleavage of the *p*-methoxyphenyl (PMP) group, affording primary amide **3.3** in 88% yield with an excellent enantiomeric ratio (99:1 er).^43^ Lastly, an intramolecular Heck coupling of **2.6**, employing Pd­(PPh_3_)­Cl_2_ and K_2_CO_3_ in toluene at 100 °C, successfully delivered product **3.4** in a satisfactory yield with 96:4 er.^44^

**Scheme 3:**
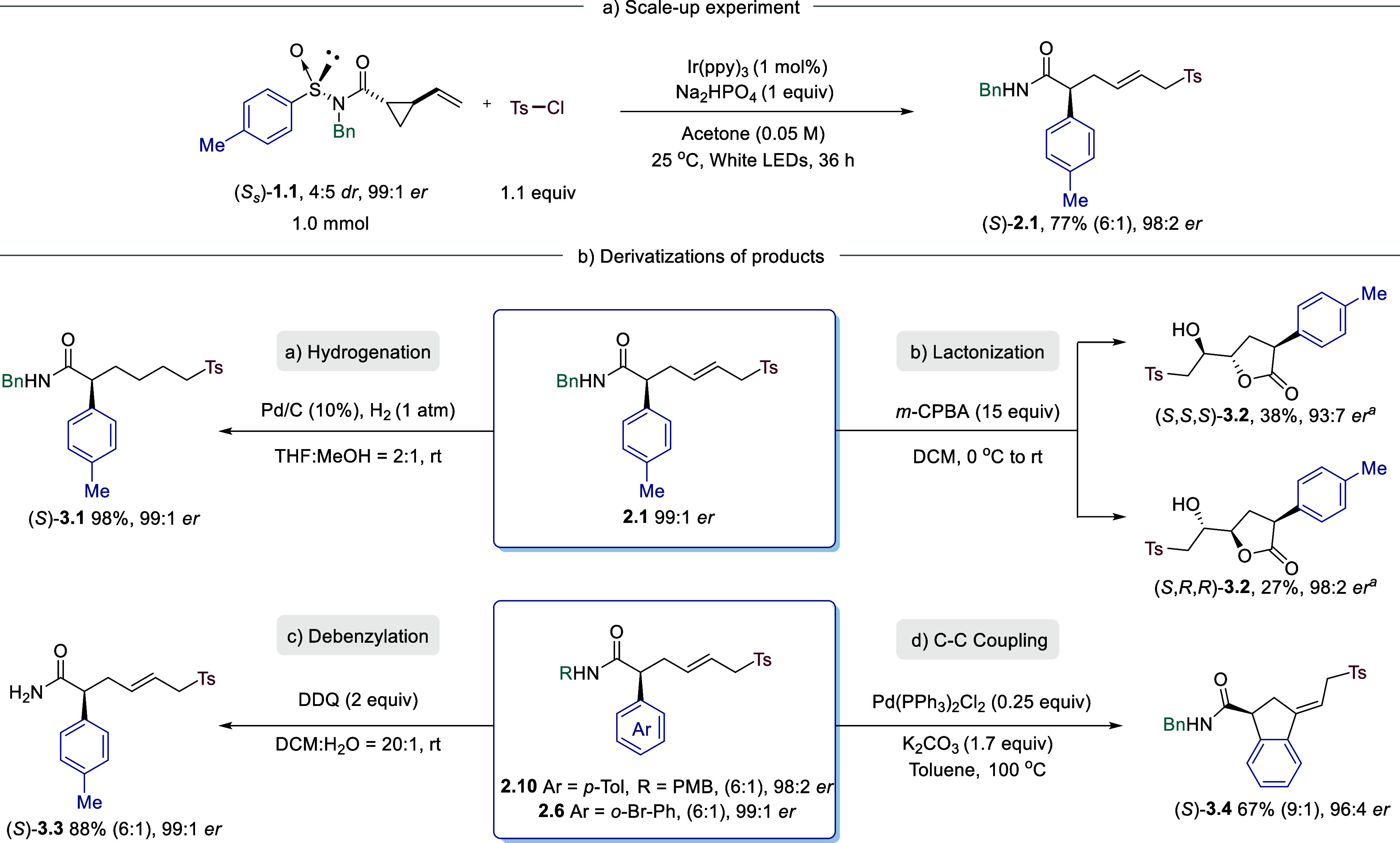
Synthetic Applications^a^

a After recrystallization in *n*-hexane/EtOAc.

## Supplementary Material



## Notes

Experimental procedures, spectroscopic data for compounds including ^1^H, ^13^C, and ^19^F NMR spectra, HPLC traces, and crystallographic data (PDF)
