# Supplementary material for: Remote 1,5-Difunctionalization of Alkenes via a Sulfinyl-Smiles Rearrangement and Vinylcyclopropane Ring-Opening Cascade
Source: JACS Au. 2026 May 21;6(6):3249–54. doi: 10.1021/jacsau.6c00241 (PMC13291956; doi:10.1021/jacsau.6c00241)
Supplement: Supplementary file 1 [file au6c00241_si_001.pdf]

Supporting Information

Remote 1,5-Difunctionalization of Alkenes via a Sulfinyl-Smiles Rearrangement and Vinylcyclopropane Ring-Opening Cascade

Yawen Hu[1], Klorenta Nitaj[1], Cristina Nevado[1]*

[1]Department of Chemistry, University of Zurich, Winterthurerstrasse 190, CH 8057 Zurich, Switzerland.

Table of Contents

1. General Information 2
2. Optimization of reaction conditions and control experiments 4
3. Synthesis and characterization of chiral sulfinamides (**1.1-1.14**) 9
4. General procedure and characterization of amides (**2.1-2.33**) 16
5. Scale-up experiment and derivatizations 31
6. X-Ray Crystallographic data for **2.1** (CCDC 2500555) 35
7. NMR spectra and HPLC traces 36
8. References 158

1. General Information

NMR spectra were recorded on AV2 400 MHz or AV2-500 MHz Bruker spectrometers. Chemical shifts are given in ppm. The spectra are calibrated to the residual ^{1}H and ^{13}C signals of the solvents. Multiplicities are abbreviated as follows: singlet (s), doublet (d), triplet (t), quartet (q), doublet-doublet (dd), quintet (quint), septet (sept), multiplet (m), and broad (br). Infrared spectra were recorded on a JASCO FT/IR-4100 spectrometer. High-resolution electrospray ionization and electronic impact mass spectrometry were performed on a Finnigan MAT 900 (Thermo Finnigan, San Jose, CA; USA) double focusing magnetic sector mass spectrometer. Ten spectra were acquired. A mass accuracy ≤ 2 ppm was obtained in the peak matching acquisition mode by using a solution containing 2 <l PEG200, 2 <l PPG450, and 1.5 mg NaOAc (all obtained from Sigma-Aldrich, CH-Buchs) dissolved in 100 mL MeOH (HPLC Supra grade, Scharlau, E-Barcelona) as internal standard. GC-MS analysis was done on a Finnigan Voyager GC8000 Top. The enantiomeric ratios were determined by chiral HPLC analysis performed on JASCO HPLC system equipped with a PU-980 pump, a UV-970 detector, measured at 254 or 220 nm and a chiral column. Optical rotations were measured on a JASCO P-1010 polarimeter.

Unless otherwise stated, reactions were carried out using dry solvents under nitrogen atmosphere. Starting materials were purchased from Aldrich, Fluka and TCI. Solvents were purchased in HPLC quality, degassed by purging with nitrogen and dried over activated molecular sieves of appropriate size. Alternatively, they were purged with argon and passed through alumina columns in a solvent purification system (Innovative Technology). Conversion was monitored by thin layer chromatography (TLC) using Merck TLC silica gel 60 F254 and visualized by UV-light at 254 nm, or by dipping the plates in an aqueous potassium permanganate solution followed by heating or using Advion MS. Flash column chromatography was performed over silica gel (230-400 mesh).

Figure S1: Set-up of the photoredox reaction

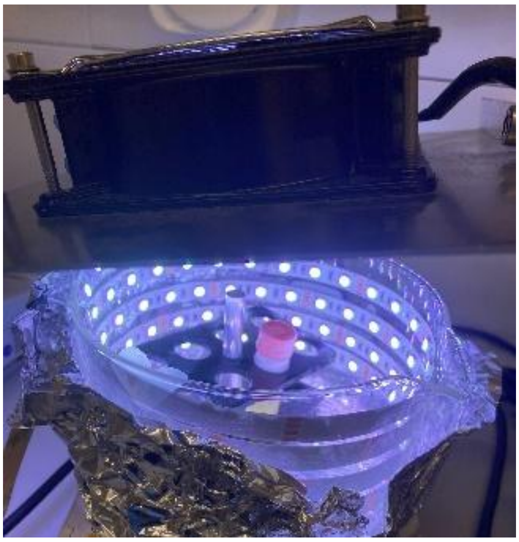

All photoredox reactions were performed in a 4 mL vial under maximum stirring rate (1400 rpm). The set-up of the photoredox reaction was composed of a crystallization dish (ø = 16 cm), covered with aluminum foil set on a stirring plate. A LED strip (7.2 W/m, white LED) was fixed to the inner wall of the crystallization dish in 5 rows. In addition, a fan (5 cm on the top of the crystallization dish) was used to keep the temperature at 25-27 °C. The vial was placed with a holder as shown in the center of the reactor at 8 cm from the light. One reaction was set-up per reactor (**Figure S1**).

2. Optimization of reaction conditions and control experiments

Table S1. Optimization of photocatalysts and solvents

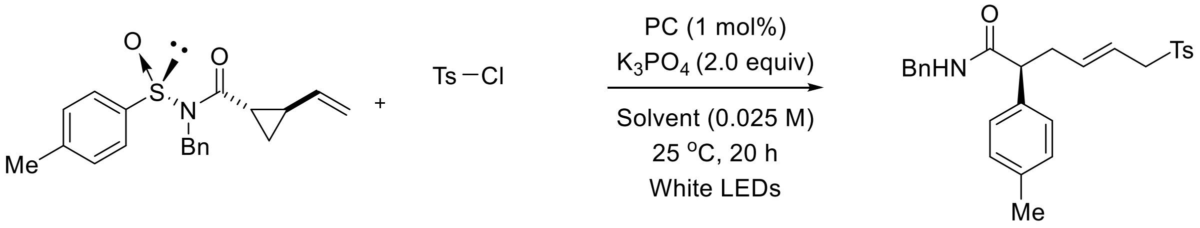

(S_s)-**1.1**, 1.2:1 *dr*, 99:1 *er* 1.1 equiv (S)-**2.1**

Entry	Photocatalyst	Solvent	Yield of **2.1** (%)[a]
1	$Ir(ppy)_3$	MeCN	66
2	Eosin Y	MeCN	16
3	$Ir[(dF(CF_3)ppy)_2(dtppy)]PF_6$	MeCN	22
4	$Ru(bpy)_3{\cdot}6H_2O$	MeCN	14
5	$Ir(dtbbpy)(ppy)_2PF_6$	MeCN	43
6	$[Ir(dFCF_3ppy)_2\text{-}(5,5'\text{-}dCF_3bpy)]PF_6$	MeCN	5
7	$Ir(ppy)_3$	$MeCN{:}H_2O$ = 9:1	58
8	$Ir(ppy)_3$	THF	50
9	$Ir(ppy)_3$	Acetone	72
10	$Ir(ppy)_3$	DMF	0
11	$Ir(ppy)_3$	DCM	65

[a] Yield was determined by ^{1}H-HMR using mesitylene as the internal standard

Table S2. Evaluation of bases

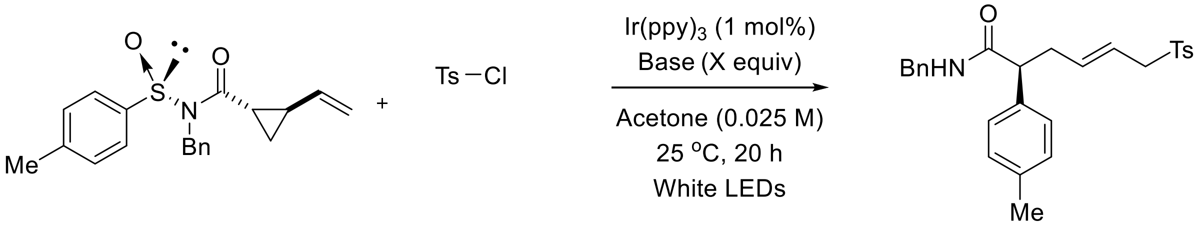

(S_s)-**1.1**, 1.2:1 *dr*, 99:1 *er* 1.1 equiv (S)-**2.1**

Entry	Base (X equiv)	Yield of **2.1** (%)[a]
1	K_2CO_3 (2.0)	28
2	KOAc (2.0)	44
3	K_2HPO_4 (2.0)	74
4	KH_2PO_4 (2.0)	74
5	Na_3PO_4 (2.0)	73
6	Li_3PO_4 (2.0)	31
7	Na_2CO_3 (2.0)	71
8	Na_2HPO_4 (2.0)	78
9	DIPEA (2.0)	63
10	NEt_3 (2.0)	67
11	Na_2HPO_4 (1.0)	77
12	Na_2HPO_4 (0.5)	74

[a] Yield was determined by ^{1}H-HMR using mesitylene as the internal standard.

Table S3. Optimization of product **2.27**

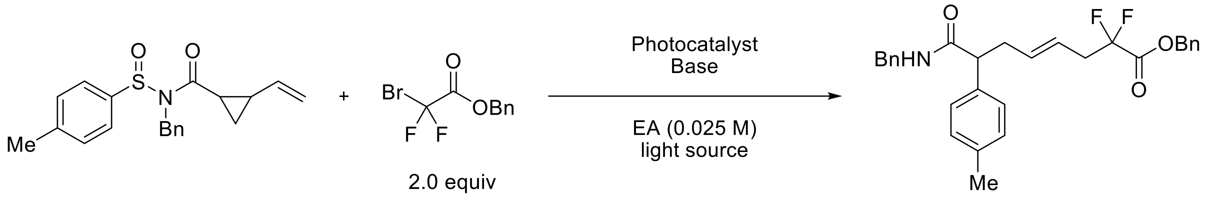

Entry	Photocatalyst	Base	Temperature (°C)	Light source	Time (h)	Yield of **2.27** (%)[a]
1	Ir(ppy)$_3$ (1 mol%)	K_3PO_4 (1 equiv)	35	White LEDs	20	40
2	Ir(ppy)$_3$ (1 mol%)	K_3PO_4 (1 equiv)	15	White LEDs	20	47
3	4CZIPN (2 mol%)	DIPEA (2 equiv)	25	370 nm	24	42

[a] Yield was determined by ^{1}H-HMR using mesitylene as the internal standard.

Table S4. Investigation of Ph_2POH as radical precursors

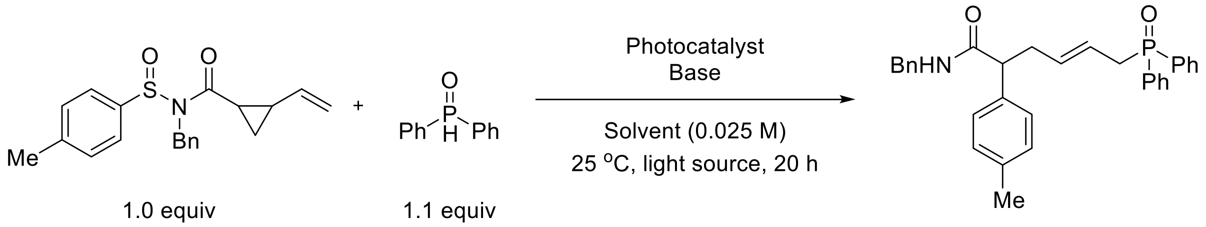

Entry	Photocatalyst	Base	Solvent	Additive	Light source	Yield of product (%)
1	Ir(dtbbpy)(ppy)$_2$PF$_6$ (1 mol%)	Cs_2CO_3 (2 equiv)	DMF	$K_2S_2O_8$ (3 equiv)	White LEDs	ND
2	4CZIPN (2 mol%)	Et_3N (2 equiv)	EA	/	427 nm	ND
3	4CZIPN (2 mol%)	Cs_2CO_3 (1.5 equiv)	DMF	/	427 nm	ND
4	Eosin Y (5 mol%)	$NaHCO_3$ (2 equiv)	DMSO	/	427 nm	ND

Table S35. Investigation of disulfide as radical precursors

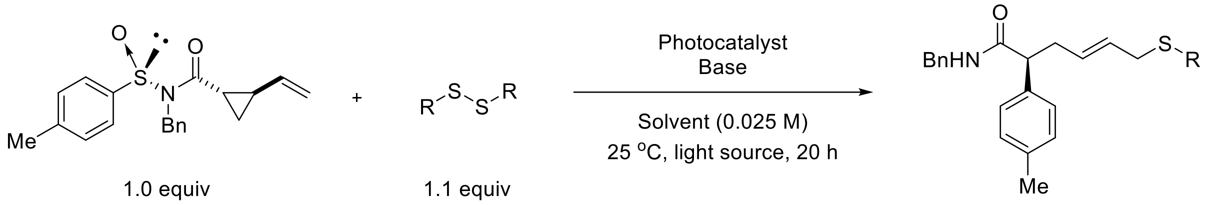

Entry	R	Photocatalyst	Base	Solvent	Light source	Result
1	*p*-Tol	$Ir(ppy)_3$ (1 mol%)	Na_2HPO_4 (1 equiv)	Acetone	White LEDs	54%, 97:3 er (5:1)
2	*p*-Tol	$Ir[(dF(CF_3)ppy)_2(dtbpy)]PF_6$ (1 mol%)	Na_2HPO_4 (1 equiv)	EA	White LEDs	37[a]
3	*p*-MeO-Ph	$Ir(ppy)_3$ (1 mol%)	Na_2HPO_4 (1 equiv)	Acetone	White LEDs	50%, 97:3 er (5:1)
4	cyclohexyl	$Ir(ppy)_3$ (1 mol%)	Na_2HPO_4 (1 equiv)	Acetone	White LEDs	ND

[a] Yield was determined by ^{1}H-HMR using mesitylene as the internal standard.

Table S5. Effect of VCP substrate configuration (*cis* vs *trans*)

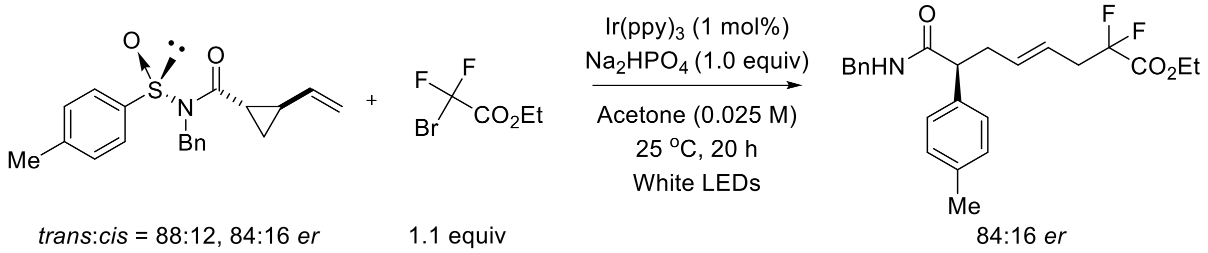

First, in the substrate scope, the precursor of the substrate that delivered product **2.14** was obtained as a trans/cis mixture (71:29). Nevertheless, product **2.14** was isolated with an er value (90:10) similar to that of the substrate (91:9). In addition, during the early stage of our investigation, we employed a VCP precursor obtained as a cis/trans mixture (ca. 12:88 with 84:16 er). When ethyl bromodifluoroacetate was used as the radical precursor, this substrate afforded the corresponding product with an unchanged er value (84:16) thus confirming that the stereochemical outcome of the process is independent of the VCP configuration.

Table S6. Control experiments

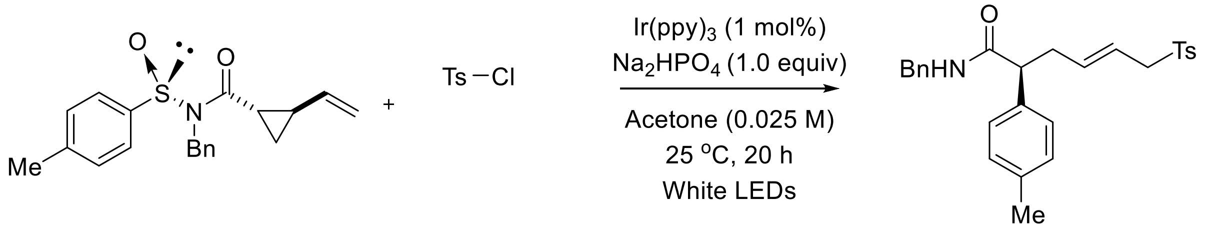

(S_s)-**1.1**, 1.2:1 *dr*, 99:1 *er* | 1.1 equiv | (S)-**2.1**

Entry	Additive	Condition	Yield of **2.1** (%)[a]
1	none	without light	0
2	none	without photocatalyst	0
3	none	without Na_2HPO_4	37
4	TEMPO	as standard	0
5	BHT	as standard	48

[a] Yield was determined by ^{1}H-HMR using mesitylene as the internal standard.

Table S7. Crossover experiment

The intramolecular nature of the aryl migration was confirmed by a crossover experiment, in which no crossover products were detected in the reaction mixture.

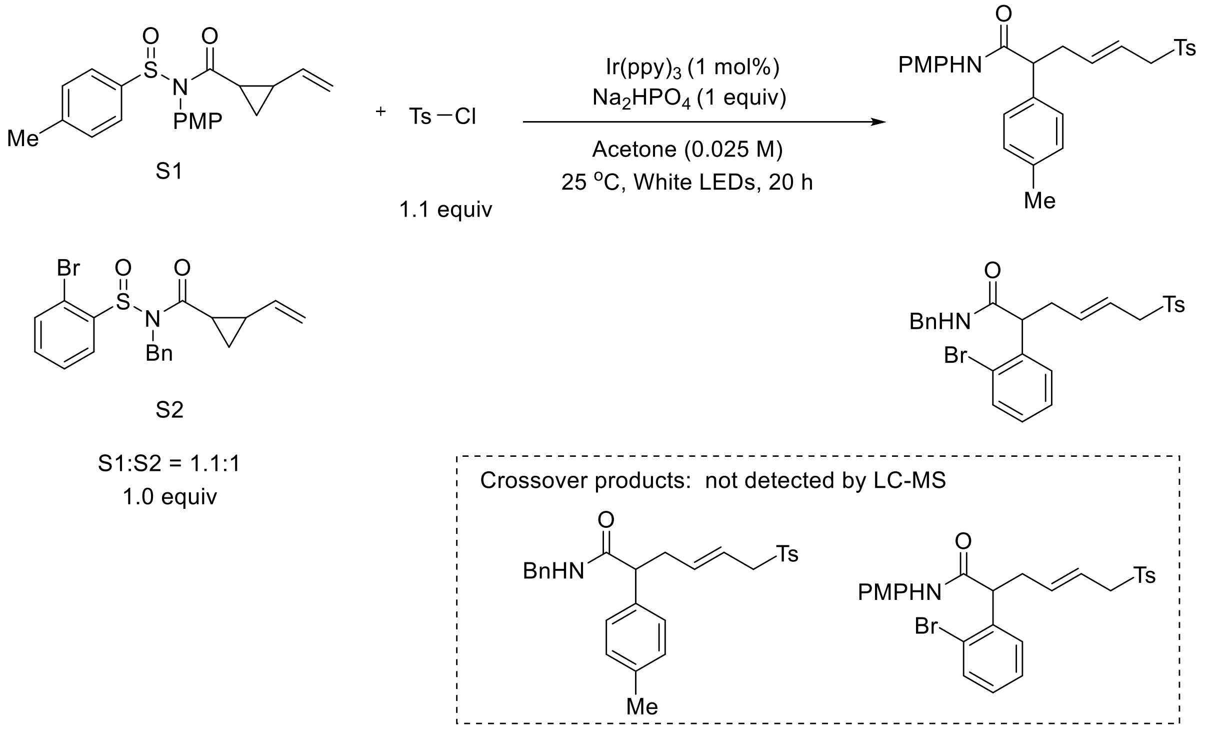

Figure S2. Sulfite detection test

Using commercially available colorimetric test strips (MQuant® Sulfite test, Merck), a positive result was obtained for the formation of bisulfite salts the crude reaction mixture.

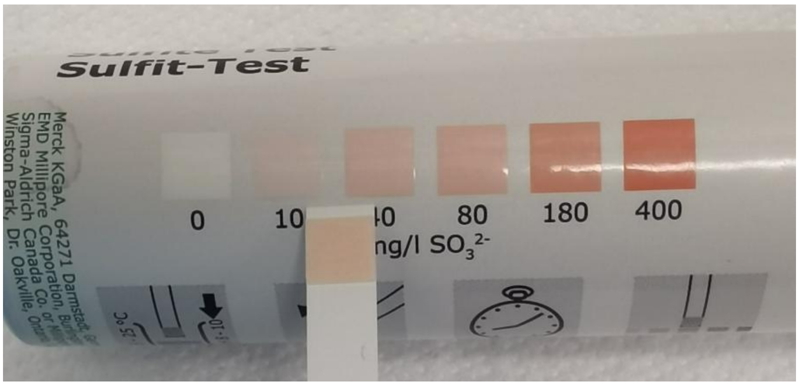

3. Synthesis and characterization of chiral sulfinamides (1.1-1.14)

3.1 Preparation of vinylcyclopropane (VCP) intermediates

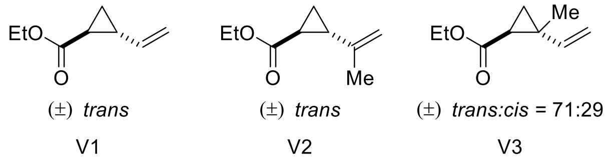

VCP intermediates V1[1] and V2-3[2] were prepared according to reported procedures and analytical data were in agreement with previously reported values.

3.2 Preparation of chiral sulfinamides

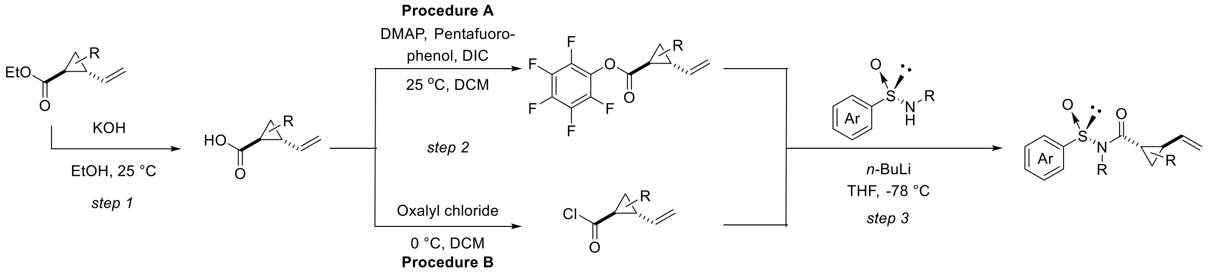

Step 1

The VCP carboxylic acids were prepared by basic hydrolysis with KOH following a previously reported procedure and analytical data were in agreement with the reported values.[3]

Step 2

Procedure A:

To a solution of the VCP carboxylic acid (24 mmol, 1.0 equiv), pentafuorophenol (26 mmol, 1.1 equiv) and DMAP (2.4 mmol, 0.10 equiv) in CH_2Cl_2 (30 mL) was added *N*,*N*'-diisopropylcarbodiimide at 25 °C. The mixture was stirred for 18 h and the resulted suspension was filtrated through a pad of celite. The filtrate was concentrated and purified by flash chromatography to obtain the corresponding PFP ester.

Procedure B:

A solution of VCP carboxylic acid (20 mmol, 1.0 equiv) and DMF (2.0 mmol, 0.10 equiv) in CH_2Cl_2 (20 mL) was cooled to 0 °C under nitrogen atmosphere followed by the dropwise addition of oxalyl chloride (24 mmol, 1.2 equiv). After stirring at room temperature for 2 h, the mixture was purified by distillation at 180 °C to obtain the corresponding pure acyl chloride as a yellow liquid.

Step 3

In a 50 mL two-necked round bottomed flask, a solution of sulfinamide (1.0 mmol, 1.0 equiv, synthesized using a previously reported procedure [4]) in THF (15 mL) was cooled down to -78 °C under nitrogen followed by dropwise addition of *n*-BuLi (2.5 M in *n*-hexane, 1.2 mmol, 1.2 equiv). After 20 min, the solution of corresponding PFP ester or acyl chloride in THF (1 mL)

was added dropwise. To achieve satisfactory reactivity and enantioselectivity of chiral substrates, the reactions were quenched at specified times by adding saturated aqueous $NaHCO_3$ solution (10 mL). The extraction was performed with EtOAc (3 x 20 mL) and the crude mixture was purified by flash chromatography. LiHMDS (1.0 M in THF) was used instead of *n*-BuLi when preparing substrates **1.3** and **1.6**.

Table S4. Specified starting material and reaction time for chiral substrate synthesis

Entry	Substrate	Starting material	Reaction time (min)
1	**1.1**, **1.8**, **1.10**, **1.11**, **1.13**	PFP ester	5
2	**1.2**, **1.7**,	PFP ester	10
3	**1.4**	PFP ester	8
4	**1.12**	PFP ester	2
5	**1.14**	PFP ester	15
6	**1.3**	Acyl chloride	1
7	**1.5**, **1.6**, **1.9**	Acyl chloride	2

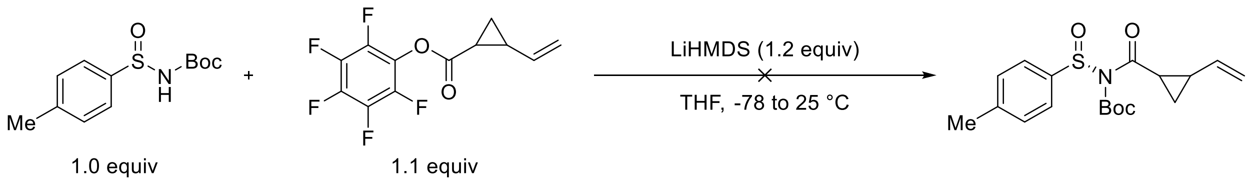

Unfortunately, a *N*-Boc protected substrate was attempted, but the synthesis did not work.

***N*-Benzyl-*N*-((*S*)-*p*-tolylsulfinyl)-2-vinylcyclopropane-1-carboxamide (1.1)**

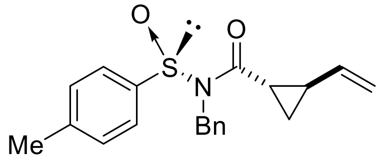

Colorless oil. ^{1}H NMR (400 MHz, Acetone-d_6) δ 7.62–7.54 (m, 2H), 7.40–7.32 (m, 2H), 7.18–7.07 (m, 4H), 7.07–7.03 (m, 1H), 5.61–5.44 (m, 1H), 5.30–5.13 (m, 1H), 4.98 (m, 1H), 4.49–4.32 (m, 2H), 2.57 (brs, 1H), 2.39 (s, 3H), 2.09–2.06 (m, 1H), 1.49–1.41 (m, 1H), 1.18–1.07 (m, 1H). ^{13}C NMR (101 MHz, Acetone-d_6) δ 173.9, 173.5, 143.7, 143.7, 141.0, 140.9, 139.4, 139.3, 138.7, 131.0, 130.9, 128.9, 128.8, 127.6, 127.6, 126.3, 126.2, 115.4, 115.2, 43.9, 43.8, 27.8, 27.4, 22.9, 22.8, 21.5, 16.7, 16.3. IR (v/cm^{-1}): 3088, 3058, 3033, 3004, 1677, 1637, 1533, 1515, 1393, 1222, 1102, 994, 978, 908, 810, 607, 478. HRMS (ESI-MS): calcd for $C_{20}H_{22}NO_2S$ $[M+H]^+$ 340.1366, found: 340.1367. HPLC conditions: AD-H column, *n*-Hexane/*i*-PrOH = 95/5, 1.0 mL/min, t_{major} = 18.2 min, t_{major} = 25.7 min, t_{minor} = 27.5 min, t_{minor} = 31.3 min, 1.2:1 dr, 99:1 er.

***N*-Benzyl-*N*-((*S*)-(4-methoxyphenyl)sulfinyl)-2-vinylcyclopropane-1-carboxamide (1.2)**

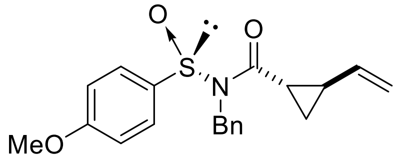

Colorless oil. ^{1}H NMR (400 MHz, Acetone-d_6) δ 7.63–7.55 (m, 2H), 7.16–7.00 (m, 7H), 5.58–5.41 (m, 1H), 5.26–5.12 (m, 1H), 5.02–4.89 (m, 1H), 4.55–4.28 (m, 2H), 3.83 (s, 3H), 2.54 (brs, 1H), 2.02–1.96 (m, 1H), 1.45–1.38 (m, 1H), 1.15–1.05 (m, 1H). ^{13}C NMR (101 MHz, Acetone-d_6) δ 173.8, 173.4, 163.7, 163.7, 139.3, 139.2, 138.7,

134.7, 134.6, 128.8, 128.7, 128.7, 128.0, 127.9, 127.5, 127.5, 115.7, 115.6, 115.3, 115.2, 56.1, 56.1, 43.4, 43.4, 27.6, 27.2, 22.8, 22.7, 16.6, 16.2. IR (v/cm^{-1}): 3062, 3030, 3006, 2839, 1673, 1592, 1495, 1393, 1307, 1254, 1222, 1101, 1077, 1021, 830, 734, 697, 462. HRMS (ESI-MS): calcd for $C_{20}H_{21}NNaO_3S$ [M+Na]$^+$ 378.1134, found: 378.1134. HPLC conditions: AD-H column, *n*-Hexane/*i*-PrOH = 95/5, 1.0 mL/min, t_{major} = 29.2 min, t_{minor} = 35.9 min, t_{minor} = 38.7 min, t_{major} = 46.2 min, 1.2:1 dr, 96:4 er.

***N*-Benzyl-*N*-((*S*)-(4-bromophenyl)sulfinyl)-2-vinylcyclopropane-1-carboxamide (1.3)**

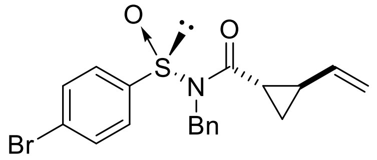

Colorless oil. ^{1}H NMR (500 MHz, Acetone-d_6) δ 7.73–7.63 (m, 2H), 7.66–7.60 (m, 2H), 7.19–7.11 (m, 3H), 7.10–7.01 (m, 2H), 5.61–5.45 (m, 1H), 5.30–5.16 (m, 1H), 5.04–4.94 (m, 1H), 4.61–4.47 (m, 1H), 4.46–4.38 (m, 1H), 2.57 (brs, 1H), 2.14–2.09 (m, 1H), 1.51–1.44 (m, 1H), 1.21–1.11 (m, 1H). ^{13}C NMR (126 MHz, Acetone-d_6) δ 174.0, 173.7, 143.3, 143.2, 139.3, 139.1, 138.5, 133.4, 133.3, 128.9, 128.7, 128.7, 128.3, 128.2, 127.7, 127.1, 115.5, 115.4, 43.8, 43.6, 27.8, 27.6, 22.9, 22.8, 16.8, 16.5. IR (v/cm^{-1}): 3082, 3033, 2926, 2852, 1734, 1677, 1572, 1387, 1220, 1118, 1065, 1007, 907, 818, 726, 696, 528, 485. HRMS (ESI-MS): calcd for $C_{19}H_{19}BrNO_2S$ [M+H]$^+$ 404.0314, found: 404.0319. HPLC conditions: IC column, *n*-Hexane/*i*-PrOH = 70/30, 1.0 mL/min, t_{minor} = 10.3 min, t_{major}= 13.2 min, t_{minor} = 14.2 min, t_{major} = 20.8 min, 1:1 dr, 92:8 er.

***N*-Benzyl-*N*-((*S*)-(4-fluorophenyl)sulfinyl)-2-vinylcyclopropane-1-carboxamide (1.4)**

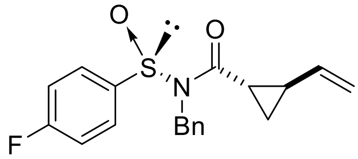

Colorless oil. ^{1}H NMR (400 MHz, Acetone-d_6) δ 7.79–7.71 (m, 2H), 7.32–7.22 (m, 2H), 7.18–7.10 (m, 3H), 7.08–7.00 (m, 2H), 5.60–5.43 (m, 1H), 5.30–5.16 (m, 1H), 5.03–4.94 (m, 1H), 4.58–4.37 (m, 2H), 2.56 (brs, 1H), 2.15–2.05 (m, 1H), 1.50–1.43 (m, 1H), 1.19–1.09 (m, 1H). ^{13}C NMR (126 MHz, Acetone-d_6) δ 174.0, 173.6, 165.7 (d, J = 250.7 Hz), 139.6, 139.6, 139.3, 139.1, 138.5, 129.0, 129.0, 128.9, 128.8, 128.6 (d, J = 11.3 Hz), 127.7, 127.6, 117.4 (d, J = 23.1 Hz), 117.3 (d, J = 22.9 Hz), 115.4, 115.3, 43.4, 43.4, 27.7, 27.5, 22.8, 22.8, 16.7, 16.4. ^{19}F NMR (376 MHz, $CDCl_3$) δ -104.2, -104.3. IR (v/cm^{-1}): 3065, 3034, 3011, 2933, 1678, 1587, 1491, 1392, 1222, 1115, 1011, 910, 836, 697, 491. HRMS (ESI-MS): calcd for $C_{19}H_{18}FNNaO_2S$ [M+Na]$^+$ 366.0934, found: 366.0979. HPLC conditions: AD-H column, *n*-Hexane/*i*-PrOH = 95/5, 1.0 mL/min, t_{minor} = 17.9 min, t_{minor} = 25.2 min, t_{major} = 26.9 min, t_{major} = 30.6 min, 1.2:1 dr, 96:4 er.

***N*-Benzyl-*N*-((*S*)-*o*-tolylsulfinyl)-2-vinylcyclopropane-1-carboxamide (1.5)**

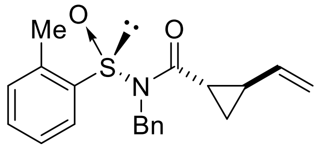

Colorless oil. ^{1}H NMR (400 MHz, Acetone-d_6) δ 8.02 (d, J = 7.8 Hz, 1H), 7.51 (t, J = 7.5 Hz, 1H), 7.47–7.40 (m, 1H), 7.16–7.11 (m, 1H), 7.11–7.04 (m, 3H), 6.97–6.87 (m, 2H), 5.64–5.48 (m, 1H), 5.30–5.19 (m, 1H), 5.01 (d, J = 10.3 Hz, 1H), 4.59–4.66 (m, 1H), 4.37–4.29 (m, 1H), 2.69 (brs, 1H), 2.28 (s, 3H), 2.17–2.10 (m, 1H), 1.57–1.42 (m, 1H), 1.24–1.14

(m, 1H). ^{13}C NMR (101 MHz, Acetone-d_6) δ 174.3, 174.2, 140.9, 140.8, 139.4, 139.2, 138.4, 138.3, 137.1, 137.0, 133.1, 133.1, 132.2, 132.2, 128.7, 128.7, 127.8, 127.6, 126.1, 115.4, 115.4, 43.7, 43.6, 28.5, 27.4, 22.2, 22.1, 18.7, 18.6, 16.9, 16.4. IR (v/cm^{-1}): 3061, 3032, 3007, 2981, 2929, 1673, 1290, 1219, 1195, 1109, 1012, 848, 754, 733, 696, 553, 468. HRMS (ESI-MS): calcd for $C_{20}H_{21}NNaO_2S$ $[M+Na]^+$ 362.1185, found: 362.1187. HPLC conditions: IC column, *n*-Hexane/*i*-PrOH = 70/30, 1.0 mL/min, t_{minor} = 10.9 min, t_{major} = 16.0 min, t_{minor} = 18.6 min, t_{major} = 34.5 min, 1.2:1 dr, 91:9 er.

***N*-Benzyl-*N*-((*S*)-(2-bromophenyl)sulfinyl)-2-vinylcyclopropane-1-carboxamide (1.6)**

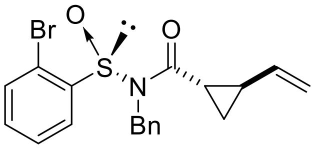

Colorless oil. ^1H NMR (500 MHz, Acetone-d_6) δ 8.03 (t, J = 6.9 Hz, 1H), 7.65 (q, J = 7.2 Hz, 1H), 7.49–7.45 (m, 1H), 7.44–7.39 (m, 1H), 7.09–7.02 (m, 3H), 6.98–6.90 (m, 2H), 5.65–5.55 (m, 1H), 5.30–5.21 (m, 1H), 5.02 (t, J = 9.9 Hz, 1H), 4.64–4.54 (m, 1H), 4.40–4.32 (m, 1H), 2.78 (brs, 1H), 2.14–2.09 (m, 1H), 1.56–1.48 (m, 1H), 1.27–1.18 (m, 1H). ^{13}C NMR (126 MHz, Acetone-d_6) δ 174.3, 174.2, 141.8, 141.7, 140.1, 139.3, 138.0, 137.9, 134.9, 134.8, 134.4, 134.4, 129.5, 129.4, 129.1, 129.0, 128.6, 128.6, 128.4, 128.3, 127.5, 127.5, 121.2, 121.0, 115.4, 115.3, 43.2, 43.1, 28.9, 27.7, 22.5, 22.5, 16.9. IR (v/cm^{-1}): 3065, 3030, 3007, 2929, 1677, 1445,1432, 1391, 1220, 1196, 1113, 1018, 907, 854, 695, 517, 467. HRMS (ESI-MS): calcd for $C_{19}H_{19}BrNO_2S$ $[M+H]^+$ 404.0314, found: 404.0319. HPLC conditions: IC column, *n*-Hexane/*i*-PrOH = 90/10, 1.0 mL/min, t_{minor} =10.4 min, t_{major} = 14.8 min, t_{minor} = 18.2 min, t_{major} = 39.5 min, 1.2:1 dr, 99:1 er.

***N*-Benzyl-*N*-((*S*)-naphthalen-1-ylsulfinyl)-2-vinylcyclopropane-1-carboxamide (1.7)**

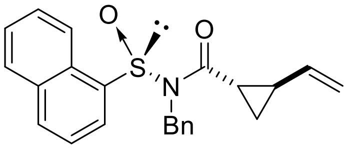

Colorless oil. ^1H NMR (400 MHz, Acetone-d_6) δ 8.25–8.18 (m, 1H), 8.10–7.97 (m, 2H), 7.91 (d, J = 8.4 Hz, 1H), 7.70–7.52 (m, 3H), 6.87–6.76 (m, 3H), 6.73–6.65 (m, 2H), 5.79–4.87 (m, 3H), 4.48 (t, J = 17.0 Hz, 1H), 4.36–4.21 (m, 1H), 2.59 (brs, 1H), 2.19–2.09 (m, 1H), 1.62–1.46 (m, 1H), 1.31–1.22 (m, 1H). ^{13}C NMR (101 MHz, Acetone-d_6) δ 174.1, 173.7, 139.5, 139.1, 138.2, 138.0, 137.7, 137.7, 134.6, 134.6, 133.9, 133.8, 130.0, 129.9, 129.8, 129.7, 128.5, 128.2, 128.2, 128.0, 127.9, 127.7, 127.7, 127.1, 127.1, 126.0, 126.0, 122.6, 122.4, 115.6, 115.4, 43.3, 43.2, 28.9, 27.6, 22.3, 17.1, 16.5. IR (v/cm^{-1}): 3061, 3007, 2985, 2928, 1675, 1392, 1276, 1223, 1108, 801, 749. HRMS (ESI-MS): calcd for $C_{23}H_{21}NNaO_2S$ $[M+Na]^+$ 398.1185, found: 398.1237. HPLC conditions: IC column, *n*-Hexane/*i*-PrOH = 70/30, 1.0 mL/min, t_{minor} = 13.7 min, t_{minor} = 21.5 min, t_{major} = 25.6 min, t_{major} = 61.3 min, 3:1 dr, 91:9 er.

***N*-Benzyl-*N*-((*S*)-naphthalen-2-ylsulfinyl)-2-vinylcyclopropane-1-carboxamide (1.8)**

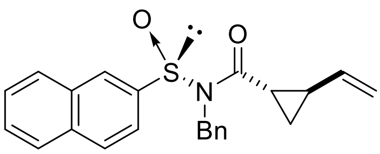

Colorless oil. ^1H NMR (400 MHz, Acetone-d_6) δ 8.36 (d, J = 6.0 Hz, 1H), 8.10 (d, J = 7.8 Hz, 1H), 8.04–7.96 (m, 2H), 7.71–7.57 (m, 3H), 7.14–6.99 (m, 5H), 5.64–5.44 (m, 1H), 5.35–5.15 (m, 1H), 5.07–4.88 (m, 1H), 4.57–4.36 (m, 2H), 2.64 (brs,

1H), 2.15–2.09 (m, 1H), 1.52–1.45 (m, 1H), 1.23–1.10 (m, 1H). ^{13}C NMR (101 MHz, Acetone-*d*$_6$) δ 173.9, 173.5, 141.1, 141.0, 139.3, 139.1, 138.5, 138.5, 135.8, 133.7, 133.7, 130.5, 130.4, 129.8, 129.3, 129.3, 128.9, 128.9, 128.8, 128.6, 128.4, 128.3, 127.5, 127.3, 127.2, 121.8, 121.6, 115.4, 115.2, 44.1, 43.9, 27.8, 27.4, 22.9, 22.8, 16.7, 16.3. IR (v/cm^{-1}): 3058, 3033, 3007, 2926, 2851, 1675, 1516, 1391, 1220, 1107, 1013, 906, 812, 746, 696, 474, 407. HRMS (ESI-MS): calcd for $C_{23}H_{22}NO_2S$ $[M+H]^+$ 376.1366, found: 376.1363. HPLC conditions: IG-3 column, *n*-Hexane/*i*-PrOH = 80/20, 1.0 mL/min, t_{minor} = 18.3 min, t_{minor} = 19.7 min, t_{major} = 20.9 min, t_{major} = 22.7 min, 1.2:1 dr, 92:8 er.

***N*-Benzyl-*N*-((*S*)-thiophen-2-ylsulfinyl)-2-vinylcyclopropane-1-carboxamide (1.9)**

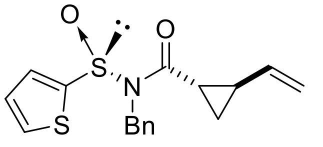

Colorless oil. ^{1}H NMR (400 MHz, Acetone-*d*$_6$) δ 7.88–7.82 (m, 1H), 7.53–7.46 (m, 1H), 7.24–7.06 (m, 6H), 5.55–5.35 (m, 1H), 5.2 –5.04 (m, 1H), 4.94–4.85 (m, 1H), 4.70–4.40 (m, 2H), 2.38 (brs, 1H), 2.00–1.92 (m, 1H), 1.43–1.34 (m, 1H), 1.11–0.99 (m, 1H). ^{13}C NMR (101 MHz, Acetone-*d*$_6$) δ 173.4, 173.0, 147.0, 146.9, 139.1, 139.0, 138.5, 133.4, 133.4, 130.9, 130.9, 129.1, 129.0, 128.9, 128.7, 128.6, 127.8, 115.4, 115.3, 43.6, 43.5, 27.6, 27.3, 22.9, 22.9, 16.6, 16.2. IR (v/cm^{-1}): 3084, 2927, 2851, 1638, 1544, 1399, 1316, 1225, 1139, 1098, 1034, 730, 698, 598, 539. HRMS (ESI-MS): calcd for $C_{17}H_{17}FNNaO_2S_2$ $[M+Na]^+$ 354.0593, found: 354.0595. HPLC conditions: IC column, *n*-Hexane/*i*-PrOH = 70/30, 1.0 mL/min, t_{minor} = 12.1 min, t_{minor} = 14.7 min, t_{major} = 17.0 min, t_{major} = 19.7 min, 1:1 dr, 96:4 er.

***N*-(4-Methoxybenzyl)-*N*-((*S*)-*p*-tolylsulfinyl)-2-vinylcyclopropane-1-carboxamide (1.10)**

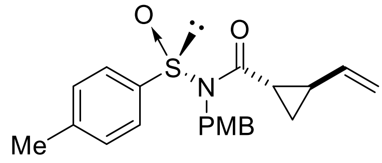

Colorless oil. ^{1}H NMR (400 MHz, Acetone-*d*$_6$) δ 7.61–7.54 (m, 2H), 7.42–7.35 (m, 2H), 7.01 (dd, *J* = 17.2, 8.7 Hz, 2H), 6.75–6.64 (m, 2H), 5.61–5.45 (m, 1H), 5.30–5.15 (m, 1H), 5.05–4.94 (m, 1H), 4.42–4.27 (m, 2H), 3.74 (s, 3H), 2.56 (brs, 1H), 2.42 (s, 3H), 2.05–2.00 (m, 1H), 1.50–1.41 (m, 1H), 1.20–1.06 (m, 1H). ^{13}C NMR (101 MHz, Acetone-*d*$_6$) δ 174.4, 173.8, 159.7, 159.7, 143.6, 143.6, 141.1, 141.0, 139.4, 139.3, 131.0, 130.9, 130.6, 130.6, 130.5, 126.3, 126.2, 115.4, 115.2, 114.2, 55.5, 43.5, 43.3, 27.7, 27.3, 22.9, 22.9, 21.5, 16.7, 16.3. IR (v/cm^{-1}): 2997, 2932, 2833, 1673, 1609, 1512, 1438, 1391, 1245, 1221, 1176, 1102, 1074, 1034, 917, 809, 604, 519, 475. HRMS (ESI-MS): calcd for $C_{21}H_{24}NO_3S$ $[M+H]^+$ 370.1471, found: 370.1475. HPLC conditions: IC column, *n*-Hexane/*i*-PrOH = 70/30, 1.0 mL/min, t_{minor} = 18.6 min, t_{major} = 24.5 min, t_{minor} = 32.3 min, t_{major} = 36.5 min, 1.5:1 dr, 99:1 er.

***N*-Methyl-*N*-((*S*)-*p*-tolylsulfinyl)-2-vinylcyclopropane-1-carboxamide (1.11)**

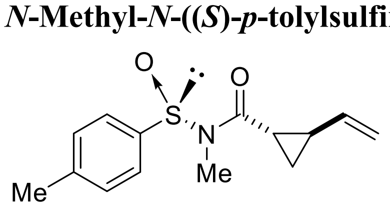

Colorless oil. ^{1}H NMR (500 MHz, Acetone-*d*$_6$) δ 7.60 (d, *J* = 8.3 Hz, 1H), 7.57 (d, *J* = 8.3 Hz, 1H), 7.48 (d, *J* = 3.0 Hz, 1H), 7.47 (d, *J* = 3.0 Hz, 1H), 5.65–5.53 (m, 1H), 5.33–5.21 (m, 1H), 5.06–4.99 (m, 1H), 2.68–2.56 (m, 4H), 2.45 (s, 3H), 2.13–2.07

(m, 1H), 1.55–1.44 (m, 1H), 1.21–1.14 (m, 1H). ^{13}C NMR (101 MHz, Acetone-d_6) δ 173.5, 173.2, 143.5, 143.5, 140.5, 139.5, 139.4, 131.1, 126.1, 126.0, 115.3, 115.2, 27.5, 27.2, 24.7, 24.4, 22.3, 22.1, 21.4, 16.4, 16.2. IR (v/cm^{-1}): 3084, 3058, 3007, 2981, 2939, 2925, 1673, 1416, 1384, 1245, 1101, 1073, 1024, 990, 945, 907, 878, 811, 625, 574, 499, 476. HRMS (ESI-MS): calcd for $C_{14}H_{18}NO_2S$ [M+H]$^+$ 264.1053, found: 264.1051. HPLC conditions: IC column, *n*-Hexane/*i*-PrOH = 70/30, 1.0 mL/min, t_{minor} = 13.3 min, t_{major} = 16.6 min, t_{minor} = 21.8 min, t_{major} = 43.6 min, 1:1 dr, 97:3 er.

_N_-Propyl-_N_-((_S_)-_p_-tolylsulfinyl)-2-vinylcyclopropane-1-carboxamide (1.12)

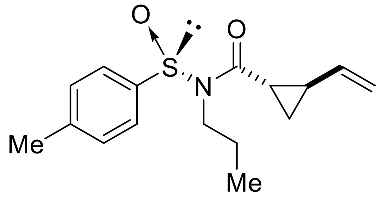

Colorless oil. ^{1}H NMR (400 MHz, Acetone-d_6) δ 7.60 (d, J = 8.3 Hz, 1H), 7.56 (d, J = 8.3 Hz, 1H), 7.42 (d, J = 6.2 Hz, 2H), 5.61–5.45 (m, 1H), 5.30–5.15 (m, 1H), 5.02–4.92 (m, 1H), 3.31–3.16 (m, 1H), 3.10–2.95 (m, 1H), 2.55 (brs, 1H), 2.40 (s, 3H), 2.07–2.02 (m, 1H), 1.50–1.40 (m, 1H), 1.40–1.32 (m, 1H), 1.15–1.07 (m, 1H), 1.06–0.93 (m, 1H), 0.66–0.56 (m, 3H). ^{13}C NMR (101 MHz, Acetone-d_6) δ 173.4, 173.1, 143.5, 143.5, 141.2, 139.6, 139.4, 130.9, 126.1, 126.0, 115.3, 115.2, 42.2, 42.1, 27.5, 27.3, 23.4, 22.5, 22.4, 21.5, 16.5, 16.2, 11.6. IR (v/cm^{-1}): 2967, 2932, 2871, 1637, 1514, 981, 902, 811, 681, 558. HRMS (ESI-MS): calcd for $C_{16}H_{21}NNaO_2S$ [M+Na]$^+$ 314.1185, found: 314.1185. HPLC conditions: IG-3 column, *n*-Hexane/*i*-PrOH = 80/20, 1.0 mL/min, t_{minor} = 8.6 min, t_{minor} = 9.5 min, t_{major} = 11.4 min, t_{major} = 14.0 min, 1.2:1 dr, 95:5 er.

_N_-Benzyl-2-(prop-1-en-2-yl)-_N_-((_S_)-_p_-tolylsulfinyl)cyclopropane-1-carboxamide (1.13)

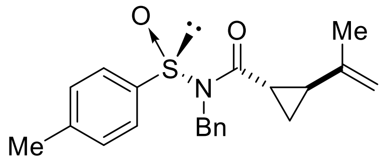

Colorless oil. ^{1}H NMR (400 MHz, Acetone-d_6) δ 7.62–7.54 (m, 2H), 7.40–7.32 (m, 2H), 7.20–7.09 (m, 4H), 7.04–7.00 (m, 1H), 4.89–4.74 (m, 2H), 4.52–4.31 (m, 2H), 2.55 (brs, 1H), 2.39 (s, 3H), 2.08–2.06 (m, 1H), 1.66 (s, 3H), 1.40–1.34 (m, 1H), 1.27–1.16 (m, 1H). ^{13}C NMR (101 MHz, Acetone-d_6) δ 174.3, 144.1, 144.0, 143.7, 143.6, 141.0, 140.8, 138.8, 131.0, 130.9, 128.9, 128.9, 128.8, 128.7, 127.7, 127.6, 126.3, 126.2, 111.7, 111.4, 44.0, 43.8, 21.7, 21.6, 21.4, 20.6, 20.3, 15.0, 14.8. IR (v/cm^{-1}): 3084, 3031, 2917, 1643, 1532, 1514, 1232, 1012, 979, 809, 697, 504. HRMS (ESI-MS): calcd for $C_{21}H_{23}NNaO_2S$ [M+Na]$^+$ 376.1342, found: 376.1300. HPLC conditions: AD-H column, *n*-Hexane/*i*-PrOH = 80/20, 1.0 mL/min, t_{major} = 7.8 min, t_{major} = 9.0 min, t_{minor} = 10.2 min, t_{minor} = 11.6 min, 1.2:1 dr, 95:5 er.

_N_-Benzyl-2-methyl-_N_-((_S_)-_p_-tolylsulfinyl)-2-vinylcyclopropane-1-carboxamide (1.14)

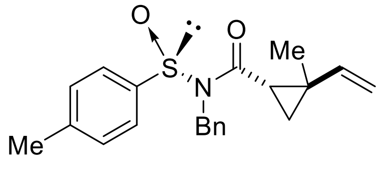

Colorless oil. ^{1}H NMR (500 MHz, Acetone-d_6) δ 7.65–7.50 (m, 2H), 7.39–7.31 (m, 2H), 7.16–7.09 (m, 3H), 7.04–6.93 (m, 2H), 5.64–5.54 (m, 1H), 5.12 (d, J = 17.2 Hz, 1H), 5.05–4.98 (m, 1H), 4.54–4.42 (m, 1H), 4.41–4.28 (m, 1H), 2.56 (brs, 1H), 2.38 (s, 3H), 1.57–1.41 (m, 1H), 1.33–1.26 (m, 1H), 1.15 (s, 3H). ^{13}C NMR (126 MHz, Acetone-d_6) δ 172.3, 144.8, 144.6, 143.7, 143.5, 143.5, 140.8, 139.7, 138.7, 138.7, 131.0, 130.9,

130.8, 130.8, 128.9, 128.8, 128.7, 128.6, 127.6, 127.5, 126.4, 126.3, 126.1, 114.8, 114.6, 112.9, 112.8, 44.3, 44.0, 43.9, 43.8, 31.1, 28.8, 21.6, 21.4, 21.2, 21.1, 20.6, 20.3, 15.2, 15.1, 14.6, 14.5. IR (v/cm^{-1}): 3088, 2061, 2956, 2926, 1679, 1379, 1217, 1101, 980, 905, 812, 697, 480. HRMS (ESI-MS): calcd for $C_{21}H_{23}NNaO_2S$ $[M+Na]^+$ 376.1342, found: 376.1306. HPLC conditions: AD-3 column, *n*-Hexane/*i*-PrOH = 90/10, 1.0 mL/min, t_{minor} = 15.4 min, t_{major} = 18.4 min, 2.3:1 dr, 91:9 er.

4. General procedure and characterization of amides (2.1-2.31)

To a 4 mL vial, the corresponding enantioenriched sulfinamide **1** (0.050 mmol, 1.0 equiv), Na_2HPO_4 (0.050 mmol, 1.0 equiv), Ir(ppy)$_3$ (0.00050 mmol, 1 mol%) and sulfonyl chloride (0.055 mmol, 1.1 equiv) were sequentially added. The vial was evacuated and then filled back with nitrogen. Acetone (2.0 mL, 0.025 M) was added followed by purging with nitrogen for 30 seconds. Next, the vial was sealed, and the reaction mixture was stirred at 1400 rpm in the photoreactor under white light irradiation (7.2 W/m, white LED). After 20 h, the mixture was filtered through a short silica plug with EtOAc as eluent. The solvent was removed, and the crude was purified by flash chromatography using a mixture EtOAc:Hexane.

(*S*,*E*)-*N*-Benzyl-2-(*p*-tolyl)-6-tosylhex-4-enamide (2.1)

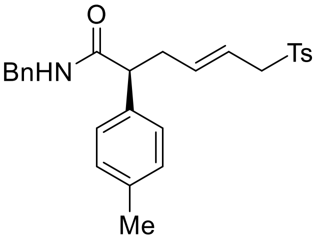

Colorless oil (76% yield, E/Z = 6:1). ^{1}H NMR (400 MHz, $CDCl_3$) δ 7.64 (d, J = 8.3 Hz, 2H), 7.31–7.24 (m, 5H), 7.15–7.11 (m, 6H), 5.66 (t, J = 5.9 Hz, 1H), 5.53–5.37 (m, 2H), 4.41 (dd, J = 14.9 Hz, 5.8 Hz, 1H), 4.33 (dd, J = 15.1 Hz, 5.8 Hz, 1H), 3.63 (d, J = 6.8 Hz, 2H), 3.31 (t, J = 7.5 Hz, 1H), 2.90 (dt, J = 14.0, 6.8 Hz, 1H), 2.52–2.43 (m, 4H), 2.33 (s, 3H). ^{13}C NMR (101 MHz, $CDCl_3$) δ 172.7, 172.6, 144.8, 144.7, 138.6, 138.3, 137.4, 136.6, 136.0, 135.9, 135.6, 129.9, 129.8, 129.7, 128.7, 128.6, 128.0, 127.9, 127.7, 127.7, 127.5, 118.4, 117.3, 60.2, 55.3, 52.5, 52.3, 43.7, 36.3, 31.3, 29.8, 21.8, 21.2. IR (v/cm^{-1}): 3312, 3031, 2925, 1650, 1511, 1454, 1315, 1301, 1149, 1135, 1087, 972, 815, 748, 699, 560, 513. HRMS (ESI-MS): calcd for $C_{27}H_{30}NO_3S$ $[M+H]^+$ 448.1941, found: 448.1949. $[\alpha]_D^{25}$ = 54.30 (c = 0.08, $CHCl_3$). HPLC conditions: IC column, n-Hexane/i-PrOH = 70/30, 1.0 mL/min, t_{major} = 43.5 min, t_{minor} = 46.5 min, t_{major} = 51.6 min, t_{minor} = 59.9 min, 99:1 er.

(*S*,*E*)-*N*-Benzyl-2-(4-methoxyphenyl)-6-tosylhex-4-enamide (2.2)

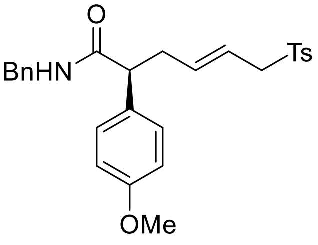

Colorless oil (62% yield, E/Z = 5:1). ^{1}H NMR (400 MHz, $CDCl_3$) δ 7.70 (d, J = 8.3 Hz, 2H), 7.37–7.30 (m, 5H), 7.22–7.16 (m, 4H), 6.91 (d, J = 8.6 Hz, 2H), 5.78 (t, J = 5.9 Hz, 1H), 5.59–5.43 (m, 2H), 4.46 (dd, J = 14.9, 5.8 Hz, 1H), 4.39 (dd, J = 15.0, 5.8 Hz, 1H), 3.85 (s, 3H), 3.69 (d, J = 7.0 Hz, 2H), 3.36 (t, J = 7.5 Hz, 1H), 2.98–2.89 (m, 1H), 2.57–2.50 (m, 1H), 2.49 (s, 3H). ^{13}C NMR (101 MHz, $CDCl_3$) δ 172.8, 172.8, 159.1, 144.8, 144.7, 138.6, 138.3, 136.6, 136.0, 135.7, 131.1, 131.0, 129.9, 129.8, 129.2, 129.1, 128.8, 128.6, 127.7, 127.7, 127.5, 118.4, 117.3, 114.4, 114.4, 60.2, 55.4, 55.3, 52.0, 51.9, 43.7, 36.4, 31.4, 29.8, 21.8. IR (v/cm^{-1}): 3312, 2920, 2856, 1650, 1509, 1301, 1247, 1179, 1135, 1086, 1031, 816, 731, 699, 665, 561, 514. HRMS (ESI-MS): calcd for $C_{27}H_{30}NO_4S$ $[M+H]^+$ 464.1890, found: 464.1893. $[\alpha]_D^{25}$ = 36.03 (c = 0.13, $CHCl_3$). HPLC conditions: AD-H column, n-Hexane/i-PrOH = 70/30, 1.0 mL/min, t_{major} = 13.9 min, t_{major} = 15.5 min, t_{minor} = 28.0 min, t_{minor} = 73.0 min, 95:5 er.

(*S*,*E*)-*N*-Benzyl-2-(4-bromophenyl)-6-tosylhex-4-enamide (2.3)

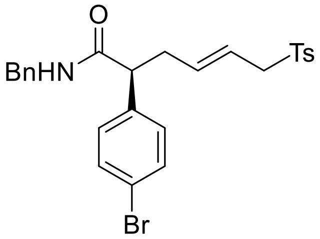

Colorless oil (71% yield, E/Z = 6:1). ^{1}H NMR (500 MHz, $CDCl_3$) δ 7.66 (d, *J* = 8.0 Hz, 2H), 7.46 (d, *J* = 8.1 Hz, 2H), 7.35–7.26 (m, 5H), 7.18–7.13 (m, 4H), 5.82 (t, *J* = 5.8 Hz, 1H), 5.56–5.49 (m, 1H), 5.47–5.38 (m, 1H), 4.42 (dd, *J* = 14.8, 5.8 Hz, 1H), 4.36 (dd, *J* = 14.6, 5.6 Hz, 1H), 3.65 (d, *J* = 7.1 Hz, 2H), 3.33 (t, *J* = 7.5 Hz, 1H), 2.91–2.83 (m, 1H), 2.52–2.45 (m, 4H). ^{13}C NMR (126 MHz, $CDCl_3$) δ 171.9, 171.8, 144.9, 144.8, 138.2, 138.1, 138.1, 138.1, 136.3, 136.0, 135.8, 132.1, 132.0, 130.0, 129.8, 129.8, 129.7, 128.8, 128.5, 127.8, 127.7, 121.6, 118.8, 117.6, 60.1, 55.3, 52.4, 52.2, 43.8, 43.8, 36.5, 31.6, 21.8. IR (v/cm^{-1}): 3316, 3068, 3034, 2917, 1650, 1537, 1487, 1288, 1134, 1086, 1011, 815, 728, 698, 664, 563, 514. HRMS (ESI-MS): calcd for $C_{26}H_{27}BrNO_3S$ $[M+H]^+$ 512.0890, found: 512.0898. $[\alpha]_D^{25}$ = 21.65 (c = 0.15, $CHCl_3$). HPLC conditions: IG-3 column, *n*-Hexane/*i*-PrOH = 60/40, 1.0 mL/min, t_{major} = 19.1 min, t_{major} = 21.6 min, t_{minor} = 43.7 min, t_{minor} = 52.5 min, 92:8 er.

(*S*,*E*)-*N*-Benzyl-2-(4-fluorophenyl)-6-tosylhex-4-enamide (2.4)

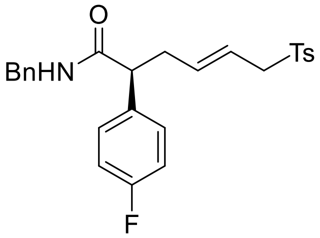

Colorless oil (69% yield, E/Z = 5:1). ^{1}H NMR (400 MHz, $CDCl_3$) δ 7.75 (d, *J* = 8.3 Hz, 2H), 7.41–7.31 (m, 7H), 7.25–7.22 (m, 2H), 7.13–7.07 (m, 2H), 5.86 (t, *J* = 5.9 Hz, 1H), 5.64–5.47 (m, 2H), 4.50–4.42 (m, 2H), 3.73 (d, *J* = 7.0 Hz, 2H), 3.43 (t, *J* = 7.5 Hz, 1H), 2.60–2.54 (m, 1H), 3.00–2.92 (m, 1H), 2.54 (s, 3H). ^{13}C NMR (101 MHz, $CDCl_3$) δ 172.3, 172.2, 162.3 (d, *J* = 247.5 Hz), 144.9, 144.8, 138.2, 138.2, 136.4, 136.1, 135.8, 135.0 (d, *J* = 3.0 Hz), 134.8 (d, *J* = 3.3 Hz), 129.9, 129.8, 129.6 (d, *J* = 8.1 Hz), 129.6 (d, *J* = 8.1 Hz), 128.8, 128.5, 127.9, 127.7, 127.6, 118.7, 117.5, 115.9 (d, *J* = 21.2 Hz), 115.8 (d, *J* = 21.2 Hz), 60.1, 55.3, 52.2, 52.0, 44.1, 43.8, 38.5, 36.6, 31.8, 21.8. ^{19}F NMR (377 MHz, $CDCl_3$) δ -114.8, -114.9. IR (v/cm^{-1}): 3304, 2923, 2852, 1650, 1541, 1507, 1301, 1223, 1134, 1086, 815, 732, 699, 560, 517. HRMS (ESI-MS): calcd for $C_{26}H_{26}FNNaO_3S$ $[M+Na]^+$ 474.1510, found: 474.1507. $[\alpha]_D^{25}$ = 29.87 (c = 0.15, $CHCl_3$). HPLC conditions: AD-3 column, *n*-Hexane/*i*-PrOH = 80/20, 1.0 mL/min, t_{major} = 24.2 min, t_{major} = 31.7 min, t_{minor} = 42.5 min, t_{minor} = 54.8 min, 96:4 er.

(*S*,*E*)-*N*-Benzyl-2-(*o*-tolyl)-6-tosylhex-4-enamide (2.5)

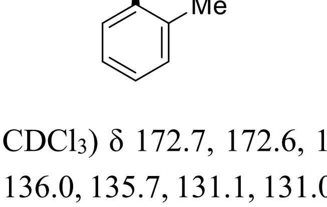

Colorless oil (60% yield, E/Z = 5:1). ^{1}H NMR (400 MHz, $CDCl_3$) δ 7.62 (d, *J* = 8.3 Hz, 2H), 7.29–7.18 (m, 9H), 7.14–7.10 (m, 2H), 5.59–5.48 (m, 2H), 5.46–5.36 (m, 1H), 4.37 (t, *J* = 5.6 Hz, 2H), 3.67–3.60 (m, 3H), 2.95 (dt, *J* = 14.0, 6.8 Hz, 1H), 2.48 (dt, *J* = 15.2, 7.7 Hz, 1H), 2.42 (s, 3H), 2.27 (s, 3H). ^{13}C NMR (101 MHz, $CDCl_3$) δ 172.7, 172.6, 144.8, 144.7, 138.7, 138.3, 138.3, 137.2, 137.1, 136.7, 136.3, 136.2, 136.0, 135.7, 131.1, 131.0, 129.9, 129.8, 128.8, 128.6, 128.5, 127.7, 127.6, 127.6, 127.6, 127.5, 126.9, 126.9, 118.5, 117.3, 60.2, 55.3, 48.7, 48.4, 43.7, 35.7, 30.7, 29.4, 27.0, 21.8, 20.0, 19.9.

IR (v/cm^{-1}): 3309, 3068, 3031, 2923, 2856, 1651, 1522, 1313, 1301, 1289, 1135, 1086, 730, 699, 664, 629, 562, 514, 472. HRMS (ESI-MS): calcd for $C_{27}H_{29}NNaO_3S$ $[M+Na]^+$ 470.1760, found: 470.1759. $[\alpha]_D^{25}$ = 66.12 (c = 0.03, $CHCl_3$). HPLC conditions: AD-H column, *n*-Hexane/*i*-PrOH = 80/20, 1.0 mL/min, t_{major} = 17.8 min, t_{major} = 20.0 min, t_{minor}= 39.5 min, t_{minor} = 52.3 min, 91:9 er.

(*S*,*E*)-*N*-Benzyl-2-(2-bromo-tolyl)-6-tosylhex-4-enamide (2.6)

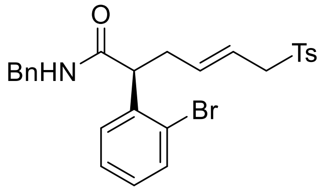

Colorless oil (64% yield, E/Z = 5:1). ^{1}H NMR (500 MHz, $CDCl_3$) δ 7.58 (dd, *J* = 8.2, 1.5 Hz, 2H), 7.48 (d, *J* = 8.1 Hz, 1H), 7.36 (d, *J* = 7.9 Hz, 1H), 7.23–7.18 (m, 6H), 7.08–7.05 (m, 3H),5.74 (t, *J*=5.9 Hz, 1H), 5.50–5.42 (m, 1H), 5.42–5.33 (m, 1H), 4.34 (dd, *J* = 15.0, 5.6 Hz, 1H), 4.27 (dd, *J* = 15.0, 5.7 Hz, 1H), 3.84 (t, *J* = 7.4 Hz, 1H), 3.62–3.52 (m, 2H), 2.84–2.77 (m, 1H), 2.43–2.36 (m, 1H), 2.34 (s, 3H). ^{13}C NMR (126 MHz, $CDCl_3$) δ 171.4, 144.8, 144.7, 138.4, 138.3, 138.1, 137.9, 136.0, 135.8, 135.6, 133.1, 133.1, 129.9, 129.8, 129.1, 129.1, 129.0, 128.8, 128.8, 128.6, 128.6, 128.3, 128.3, 127.7, 127.6, 127.6, 124.7, 124.6, 119.0, 118.0, 60.2, 55.3, 50.9, 50.8, 43.8, 43.7, 35.6, 30.6, 21.8. IR (v/cm^{-1}): 3324, 3058, 3031, 2928, 1660, 1524, 1315, 1301, 1240, 1136, 1087, 1021, 975, 818, 733, 699, 563, 515. HRMS (ESI-MS): calcd for $C_{26}H_{27}BrNO_3S$ $[M+H]^+$ 512.0890, found: 512.0892. $[\alpha]_D^{25}$ = 46.43 (c = 0.08, $CHCl_3$). HPLC conditions: IC-3 column, *n*-Hexane/*i*-PrOH = 70/30, 1.0 mL/min, t_{major} = 44.0 min, t_{minor} = 48.0 min, t_{minor} = 56.4 min, t_{major} = 66.3 min, 99:1 er.

(*S*,*E*)-*N*-Benzyl-2-(naphthalen-1-yl)-6-tosylhex-4-enamide (*E*-2.7)

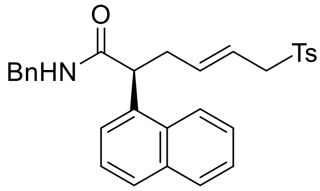

Colorless oil (37% yield). ^{1}H NMR (400 MHz, $CDCl_3$) δ 7.99–7.93 (m, 1H), 7.92–7.88 (m, 1H), 7.81 (dd, *J* = 6.9, 2.5 Hz, 1H), 7.57 (d, *J* = 8.2 Hz, 2H), 7.55–7.50 (m, 2H), 7.47–7.41 (m, 2H), 7.21–7.15 (m, 5H), 7.02–6.96 (m, 2H), 5.62–5.52 (m, 2H), 5.50–5.40 (m, 1H), 4.34 (d, *J* = 5.9 Hz, 2H), 4.13 (t, *J* = 7.3 Hz, 1H), 3.64 (d, *J* = 7.2 Hz, 2H), 3.17–3.06 (m, 1H), 2.76–2.65 (m, 1H), 2.37 (s, 3H). ^{13}C NMR (101 MHz, $CDCl_3$) δ 172.8, 144.6, 138.8, 138.1, 135.6, 134.6, 134.3, 131.6, 129.7, 129.3, 128.7, 128.6, 128.5, 127.5, 127.5, 127.5, 126.9, 126.1, 126.1, 125.8, 123.2, 118.6, 60.1, 49.2, 43.7, 35.4, 21.7. IR (v/cm^{-1}): 3304, 3054, 2925, 2848, 1650, 1512, 1315, 1301, 1232, 1134, 1086, 779, 733, 699, 664, 563, 516. HRMS (ESI-MS): calcd for $C_{30}H_{30}NO_3S$ $[M+H]^+$ 484.1941, found: 484.1940. $[\alpha]_D^{25}$ = 36.03 (c = 0.13, $CHCl_3$). HPLC conditions: AD-3 column, *n*-Hexane/*i*-PrOH = 70/30, 1.0 mL/min, t_{major} = 15.6 min, t_{minor} = 23.4 min, 88:12 er.

(*S*,*Z*)-*N*-Benzyl-2-(naphthalen-1-yl)-6-tosylhex-4-enamide (*Z*-2.7)

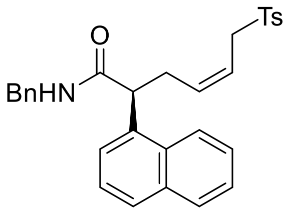

Colorless oil (12% yield). ^{1}H NMR (500 MHz, $CDCl_3$) δ 8.01 (d, *J* = 7.5 Hz, 1H), 7.91–7.87 (m, 1H), 7.79 (dd, *J* = 6.8, 2.9 Hz, 1H), 7.73 (d, *J* = 8.1 Hz, 2H), 7.56–7.49 (m, 2H), 7.46–7.41 (m, 2H), 7.30 (d, *J* = 8.0 Hz, 2H), 7.22–7.18 (m, 3H), 7.03 (dd, *J* = 6.6, 2.8 Hz, 2H), 5.81–5.73 (m, 1H), 5.70 (t, *J* = 6.0 Hz, 1H), 5.45–5.37 (m, 1H), 4.38 (dd, *J* = 14.9, 5.9 Hz, 1H), 4.32 (dd, *J* = 14.9, 5.8 Hz, 1H), 4.12 (t, *J* = 7.5 Hz, 1H), 3.79 (d, *J* = 7.9 Hz, 2H), 2.98–2.90 (m, 1H), 2.53–2.45 (m, 1H), 2.40 (s, 3H). ^{13}C NMR (126 MHz, $CDCl_3$) δ 172.8, 144.8, 138.2, 136.9, 136.0, 135.0, 134.3, 131.5, 129.9, 129.3, 128.7, 128.5, 128.5, 127.6, 127.5, 126.9, 126.2, 126.1, 125.8, 123.2, 117.3, 55.3, 48.9, 43.7, 30.6, 21.8. IR (v/cm^{-1}): 3358, 3054, 2925, 2856, 1655, 1512, 1314, 1136, 1086, 779, 717, 562, 515. HRMS (ESI-MS): calcd for $C_{30}H_{30}NO_3S$ $[M+H]^+$ 484.1941, found: 484.1945. $[\alpha]_D^{25}$ = 98.08 (c = 0.05, $CHCl_3$). HPLC conditions: AD-3 column, *n*-Hexane/*i*-PrOH = 70/30, 1.0 mL/min, t_{major} = 14.9 min, t_{minor} = 24.9 min, 88:12 er.

(*S*,*E*)-*N*-Benzyl-2-(naphthalen-2-yl)-6-tosylhex-4-enamide (2.8)

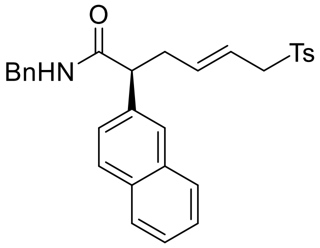

Colorless oil (51% yield, E/Z = 5:1). ^{1}H NMR (400 MHz, $CDCl_3$) δ 7.87–7.81 (m, 3H), 7.70 (s, 1H), 7.60 (d, *J* = 8.3 Hz, 2H), 7.53–7.49 (m, 2H), 7.40 (dd, *J* = 8.5, 1.9 Hz, 1H), 7.27–7.20 (m, 5H), 7.16–7.12 (m, 2H), 5.78 (t, *J* = 5.9 Hz, 1H), 5.61–5.52 (m, 1H), 5.50–5.41 (m, 1H), 4.44 (dd, *J* = 14.9, 5.8 Hz, 1H), 4.36 (dd, *J* = 14.7, 5.9 Hz, 1H), 3.65 (d, *J* = 7.1 Hz, 2H), 3.56 (t, *J* = 7.5 Hz, 1H), 3.07–2.98 (m, 1H), 2.68–2.58 (m, 1H), 2.42 (s, 3H). ^{13}C NMR (101 MHz, $CDCl_3$) δ 172.4, 172.3, 144.8, 144.6, 138.4, 138.2, 136.6, 136.6, 136.5, 135.6, 133.6, 133.5, 132.8, 129.9, 129.7, 128.9, 128.9, 128.7, 128.5, 128.5, 127.9, 127.9, 127.8, 127.7, 127.7, 127.5, 127.1, 127.1, 126.6, 126.2, 126.2, 125.9, 125.7, 118.6, 117.4, 60.1, 55.3, 52.9, 52.8, 43.8, 36.3, 31.3, 29.4, 27.0, 21.7. IR (v/cm^{-1}): 3304, 3058, 2922, 2848, 1650, 1535, 1301, 1147, 1086, 970, 817, 734, 700, 560, 516. HRMS (ESI-MS): calcd for $C_{30}H_{30}NO_3S$ $[M+H]^+$ 484.1941, found: 484.1944. $[\alpha]_D^{25}$ = 3.41 (c = 0.07, $CHCl_3$). HPLC conditions: IG-3 column, *n*-Hexane/*i*-PrOH = 50/50, 1.0 mL/min, t_{minor} = 20.3 min, t_{minor} = 30.1 min, t_{major} = 48.6 min, t_{major} = 85.8 min, 92:8 er.

(*R*,*E*)-*N*-Benzyl-2-(thiophen-2-yl)-6-tosylhex-4-enamide (2.9)

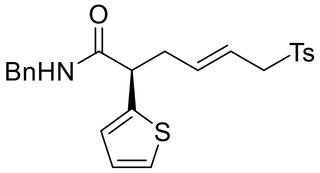

Colorless oil (70% yield, E/Z = 6:1). ^{1}H NMR (400 MHz, $CDCl_3$) δ 7.58 (d, *J* = 8.3 Hz, 2H), 7.25–7.16 (m, 6H), 7.12–7.08 (m, 2H), 6.89 (dd, *J* = 5.2, 3.5 Hz, 1H), 6.82 (d, *J* = 2.8 Hz, 1H), 5.84 (t, *J* = 5.9 Hz, 1H), 5.49–5.36 (m, 2H), 4.35–4.29 (m, 2H), 3.62 (t, *J* = 7.4 Hz, 1H), 3.57 (d, *J* = 6.5 Hz, 2H), 2.88–2.78 (m, 1H), 2.54–2.45 (m, 1H), 2.36 (s, 3H). ^{13}C NMR (101 MHz, $CDCl_3$) δ 171.5, 171.4, 144.8, 144.7, 141.3, 141.2, 138.0, 137.7, 135.8, 135.8, 135.5, 129.9, 129.7, 128.7, 128.5, 128.4, 127.6, 127.6, 127.5, 127.0, 126.9, 125.9, 125.7, 125.2, 125.2, 119.0, 117.8, 60.0, 55.2, 48.0, 47.9, 43.7, 37.3, 32.4, 31.6, 22.7, 21.7. IR (v/cm^{-1}): 3312,

3065, 2920, 2852, 1652, 1536, 1301, 1265, 1238, 1147, 1086, 970, 816, 732, 698, 629, 562, 514, 473. HRMS (ESI-MS): calcd for $C_{24}H_{26}NO_3S_2$ $[M+H]^+$ 440.1349, found: 440.1349. $[\alpha]_D^{25}$ = 24.11 (c = 0.10, $CHCl_3$). HPLC conditions: AD-H column, *n*-Hexane/*i*-PrOH = 80/20, 1.0 mL/min, t_{major} = 22.2 min, t_{major} =24.4 min, t_{minor} = 34.3 min, t_{minor} = 43.9min, 95:5 er.

(*S*,*E*)-*N*-(4-Methoxybenzyl)-2-(*p*-tolyl)-6-tosylhex-4-enamide (2.10)

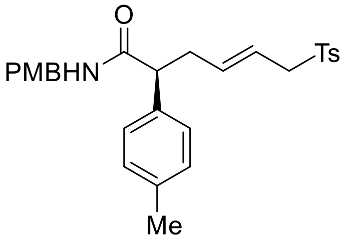

Colorless oil (63% yield, E/Z = 6:1). ^{1}H NMR (500 MHz, $CDCl_3$) δ 7.64 (d, *J* = 8.0 Hz, 2H), 7.29 (d, *J* = 7.9 Hz, 2H), 7.13–7.08 (m, 4H), 7.06 (d, *J* = 8.6 Hz, 2H), 6.80 (d, *J* = 8.7 Hz, 2H), 5.62 (t, *J* = 5.8 Hz, 1H), 5.51–5.45 (m, 1H), 5.44–5.36 (m, 1H), 4.33 (dd, *J* = 14.6, 5.8 Hz, 1H), 4.25 (dd, *J* = 14.7, 5.7 Hz, 1H), 3.77 (s, 3H), 3.63 (d, *J* = 7.0 Hz, 2H), 3.29 (t, *J* = 7.5 Hz, 1H), 2.92–2.84 (m, 1H), 2.50–2.45 (m, 1H), 2.43 (s, 3H), 2.33 (s, 3H). ^{13}C NMR (126 MHz, $CDCl_3$) δ 172.5, 172.5, 159.3, 159.1, 144.8, 144.7, 138.7, 137.4, 137.3, 136.6, 136.1, 136.0, 135.7, 134.7, 130.4, 130.4, 129.9, 129.8, 129.7, 129.7, 129.1, 129.1, 128.6, 128.0, 127.9, 118.4, 117.3, 114.3, 114.1, 60.2, 55.4, 55.3, 52.5, 52.3, 43.6, 43.2, 43.2, 38.4, 36.3, 31.3, 21.8, 21.2. IR (v/cm^{-1}): 3304, 2925, 1650, 1512, 1301, 1246, 1177, 1135, 1087, 1033, 815, 732, 561, 515. HRMS (ESI-MS): calcd for $C_{28}H_{32}NO_4S$ $[M+H]^+$ 478.2047, found: 478.2047. $[\alpha]_D^{25}$ = 37.21 (c = 0.12, $CHCl_3$). HPLC conditions: AD-H column, *n*-Hexane/*i*-PrOH = 80/20, 1.0 mL/min, t_{major} = 29.1 min, t_{major} = 35.5 min, t_{minor} = 41.3 min, t_{minor} = 62.4 min, 98:2 er.

(*S*,*E*)-*N*-Methyl-2-(*p*-tolyl)-6-tosylhex-4-enamide (2.11)

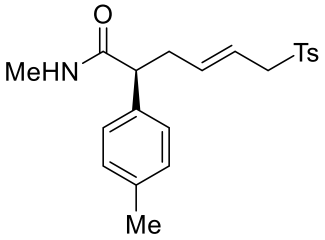

Colorless oil (76% yield, E/Z = 6:1). ^{1}H NMR (500 MHz, $CDCl_3$) δ 7.64 (d, *J* = 8.3 Hz, 2H), 7.30 (d, *J* = 8.1 Hz, 2H), 7.14–7.08 (m, 4H), 5.53–5.45 (m, 1H), 5.45–5.34 (m, 2H), 3.65 (d, *J* = 7.1 Hz, 2H), 3.27 (t, *J* = 7.5 Hz, 1H), 2.91–2.84 (m, 1H), 2.71 (d, *J* = 4.9 Hz, 3H), 2.48–2.42 (m, 4H), 2.33 (s, 3H). ^{13}C NMR (126 MHz, $CDCl_3$) δ 173.3, 173.3, 144.8, 144.7, 138.8, 137.4, 137.4, 136.8, 136.2, 136.1, 136.1, 135.7, 129.9, 129.8, 129.7, 128.6, 128.6, 128.1, 128.0, 118.3, 117.1, 60.2, 55.4, 52.5, 52.3, 36.2, 31.2, 26.6, 26.6, 21.8, 21.2. IR (v/cm^{-1}): 3316, 3054, 2917, 1650, 1512, 1314, 1265, 1135, 1265, 1135, 1087, 816, 732, 701, 665, 629, 559, 508. HRMS (ESI-MS): calcd for $C_{21}H_{26}NO_3S$ $[M+H]^+$ 372.1628, found: 372.1632. $[\alpha]_D^{25}$ = 74.93 (c = 0.09, $CHCl_3$). HPLC conditions: AD-H column, *n*-Hexane/EtOH = 70/30, 1.0 mL/min, t_{major} = 8.1 min, t_{minor} = 12.6 min, t_{minor} = 14.6 min, t_{major} = 34.6 min, 97:3 er.

(*S*,*E*)-*N*-Propyl-2-(*p*-tolyl)-6-tosylhex-4-enamide (2.12)

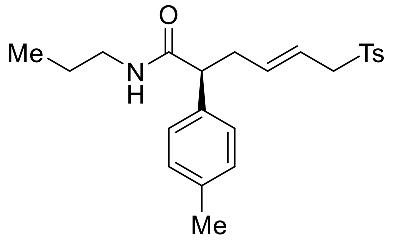

Colorless oil (86% yield, E/Z = 6:1). ^{1}H NMR (400 MHz, $CDCl_3$) δ 7.63 (d, *J* = 8.3 Hz, 2H), 7.30 (d, *J* = 8.0 Hz, 2H), 7.14–7.09 (m, 4H), 5.53–5.33 (m, 3H), 3.64 (d, *J* = 6.9 Hz, 2H), 3.26 (t, *J* = 7.4 Hz, 1H), 3.14–3.09 (m, 2H), 2.90–2.81 (m, 1H), 2.48–2.40 (m, 4H), 2.33 (s, 3H), 1.43–1.37 (m, 2H), 0.78 (t, *J* = 7.4 Hz, 3H). ^{13}C NMR (101 MHz, $CDCl_3$) δ 172.7, 172.6, 144.8, 144.7, 138.8, 137.3, 136.8, 136.4, 136.2, 136.1, 135.7, 129.9, 129.8, 129.7, 129.7, 128.6, 128.6, 128.0, 127.9, 118.2, 117.1, 60.2, 55.4, 52.5, 52.3, 41.5, 36.2, 31.3, 29.8, 22.9, 22.8, 21.8, 21.2, 14.2, 11.3. IR (v/cm^{-1}): 3312, 2923, 2856, 1647, 1512, 1301, 1135, 1086, 966, 815, 728, 562, 505. HRMS (ESI-MS): calcd for $C_{23}H_{29}NNaO_3S$ $[M+Na]^+$ 422.1760, found: 422.1756. $[\alpha]_D^{25}$ = 34.31 (c = 0.21, $CHCl_3$). HPLC conditions: OD-H column, *n*-Hexane/*i*-PrOH = 95/5, 1.0 mL/min, t_{major} = 36.5 min, t_{major} = 40.2 min, t_{minor} = 46.9 min, t_{minor} = 56.0 min, 95:5 er.

(*S*,*E*)-*N*-Benzyl-5-methyl-2-(*p*-tolyl)-6-tosylhex-4-enamide (*E*-2.13)

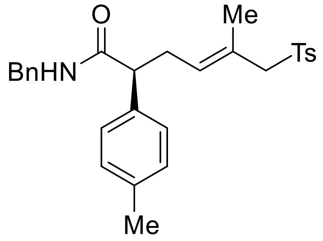

Colorless oil (40% yield). ^{1}H NMR (400 MHz, $CDCl_3$) δ 7.65 (d, *J* = 8.2 Hz, 2H), 7.31–7.24 (m, 5H), 7.16–7.09 (m, 6H), 5.67 (t, *J* = 5.8 Hz, 1H), 5.13 (t, *J* = 7.3 Hz, 1H), 4.40 (dd, *J* = 14.8, 5.8 Hz, 1H), 4.33 (dd, *J* = 14.9, 5.8 Hz, 1H), 3.62 (s, 2H), 3.24 (t, *J* = 7.5 Hz, 1H), 2.91–2.81 (m, 1H), 2.50–2.41 (m, 4H), 2.33 (s, 3H), 1.68 (s, 3H). ^{13}C NMR (101 MHz, $CDCl_3$) δ 172.9, 144.6, 138.4, 137.3, 136.3, 136.1, 133.4, 129.7, 128.8, 128.5, 128.0, 127.7, 127.5, 125.4, 66.3, 52.5, 43.8, 32.3, 21.8, 21.2, 17.2. IR (v/cm^{-1}): 3297, 3031, 2925, 2856, 1739, 1651, 1537, 1511, 1312, 1301, 1289, 1131, 1086, 816, 733, 699, 670, 628, 594, 567, 534, 512. HRMS (ESI-MS): calcd for $C_{28}H_{32}NO_3S$ $[M+H]^+$ 462.2097, found: 461.2098. $[\alpha]_D^{25}$ = 19.56 (*c* = 0.06, $CHCl_3$). HPLC conditions: IC column, *n*-Hexane/*i*-PrOH = 70/30, 1.0 mL/min, t_{major} = 30.9 min, t_{minor} = 43.6 min, 95:5 er.

(*S*,*Z*)-*N*-Benzyl-5-methyl-2-(*p*-tolyl)-6-tosylhex-4-enamide (*Z*-2.13)

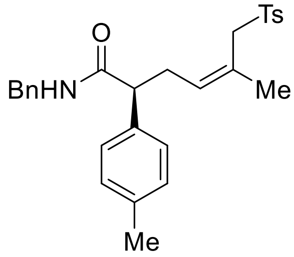

Colorless oil (19% yield). ^{1}H NMR (500 MHz, $CDCl_3$) δ 7.75 (d, *J* = 8.1 Hz, 2H), 7.33–7.22 (m, 5H), 7.17–7.11 (m, 6H), 5.80 (t, *J* = 5.8 Hz, 1H), 5.46 (t, *J* = 7.2 Hz, 1H), 4.46 (dd, *J* = 14.9, 6.1 Hz, 1H), 4.28 (dd, *J* = 14.9, 5.5 Hz, 1H), 3.89 (d, *J* = 13.7 Hz, 1H), 3.73 (d, *J* = 13.7 Hz, 1H), 3.25 (t, *J* = 7.5 Hz, 1H), 2.63–2.55 (m, 1H), 2.42 (s, 3H), 2.33 (s, 3H), 2.16–2.09 (m, 1H), 1.78 (s, 3H). ^{13}C NMR (126 MHz, $CDCl_3$) δ 172.9, 144.8, 138.5, 137.2, 136.6, 136.4, 132.2, 130.0, 129.6, 128.8, 128.5, 127.9, 127.7, 127.5, 125.0, 59.6, 52.6, 43.7, 32.4, 24.5, 21.8, 21.2. IR (v/cm^{-1}): 3309, 3031, 2923, 2856, 1742, 1650, 1511, 1313, 1302, 1289, 1131, 1086, 816, 734, 700, 669, 631, 585, 569, 516. HRMS (ESI-MS): calcd for $C_{28}H_{32}NO_3S$ $[M+H]^+$ 462.2097, found: 462.2096. $[\alpha]_D^{25}$ = 32.70

(c = 0.03, $CHCl_3$). HPLC conditions: IC column, *n*-Hexane/*i*-PrOH = 70/30, 1.0 mL/min, t_{major} = 30.5 min, t_{minor} = 35.3 min, 95:5 er.

(*S*,*E*)-*N*-Benzyl-4-methyl-2-(*p*-tolyl)-6-tosylhex-4-enamide (*E*-2.14)

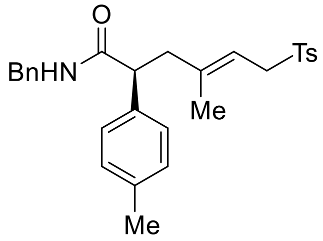

Colorless oil (51% yield). ^1H NMR (500 MHz, $CDCl_3$) δ 7.60 (d, *J* = 8.1 Hz, 2H), 7.31–7.25 (m, 5H), 7.19–7.13 (m, 6H), 5.74 (t, *J* = 5.8 Hz, 1H), 5.17 (t, *J* = 7.8 Hz, 1H), 4.42 (dd, *J* = 14.9, 5.8 Hz, 1H), 4.36 (dd, *J* = 14.9, 5.8 Hz, 1H), 3.74–3.65 (m, 2H), 3.54 (t, *J* = 7.5 Hz, 1H), 2.97 (dd, *J* = 14.6, 6.9 Hz, 1H), 2.51–2.43 (m, 4H), 2.36 (s, 3H), 1.35 (s, 3H). ^{13}C NMR (126 MHz, $CDCl_3$) δ 172.9, 144.6, 143.7, 138.3, 137.4, 136.1, 135.9, 129.8, 129.7, 128.7, 128.6, 128.0, 127.7, 127.5, 112.7, 56.2, 51.1, 43.8, 42.6, 21.8, 21.2, 16.6. IR (v/cm^{-1}): 3304, 3054, 2925, 2848, 1650, 1512, 1315, 1301, 1232, 1134, 1086, 779, 733, 699, 664, 563, 516. HRMS (ESI-MS): calcd for $C_{28}H_{32}NO_3S$ $[M+H]^+$ 462.2097, found: 462.2100. $[\alpha]_D^{25}$ = 2.16 (c = 0.09, $CHCl_3$). HPLC conditions: AD-3 column, *n*-Hexane/*i*-PrOH = 70/30, 1.0 mL/min, t_{major} = 10.7 min, t_{minor} = 20.8 min, 90:10 er.

(*S*,*Z*)-*N*-Benzyl-4-methyl-2-(*p*-tolyl)-6-tosylhex-4-enamide (*Z*-2.14)

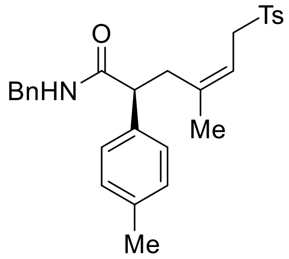

Colorless oil (13% yield). ^1H NMR (400 MHz, $CDCl_3$) δ 7.70 (d, *J* = 8.3 Hz, 2H), 7.34–7.25 (m, 5H), 7.18–7.10 (m, 6H), 5.96 (t, *J* = 5.6 Hz, 1H), 5.19 (t, *J* = 7.8 Hz, 1H), 4.45 (dd, *J* = 14.8, 6.1 Hz, 1H), 4.28 (dd, *J* = 14.8, 5.4 Hz, 1H), 3.73 (dd, *J* = 14.4, 8.1 Hz, 1H), 3.66 (dd, *J* = 14.5, 7.7 Hz, 1H), 3.46 (t, *J* = 7.6 Hz, 1H), 2.74 (dd, *J* = 13.8, 8.1 Hz, 1H), 2.43 (s, 3H), 2.32 (s, 3H), 2.22 (dd, *J* = 13.8, 7.1 Hz, 1H), 1.67 (s, 3H). ^{13}C NMR (101 MHz, $CDCl_3$) δ 172.6, 144.6, 143.9, 138.2, 137.3, 136.4, 136.1, 129.8, 129.5, 128.6, 128.3, 127.8, 127.7, 127.4, 112.9, 56.0, 51.5, 43.7, 35.9, 24.4, 21.7, 21.1. IR (v/cm^{-1}): 3358, 3054, 2925, 2856, 1655, 1512, 1314, 1136, 1086, 779, 717, 562, 515. HRMS (ESI-MS): calcd for $C_{28}H_{32}NO_3S$ $[M+H]^+$ 462.2097, found: 462.2100. $[\alpha]_D^{25}$ = 9.89 (c = 0.09, $CHCl_3$). HPLC conditions: IC column, *n*-Hexane/*i*-PrOH = 70/30, 1.0 mL/min, t_{major} = 30.7 min, t_{minor} = 42.4 min, 91:9 er.

(*S*,*E*)-*N*-Benzyl-6-((4-(*tert*-butyl)phenyl)sulfonyl)-2-(*p*-tolyl)hex-4-enamide (2.15)

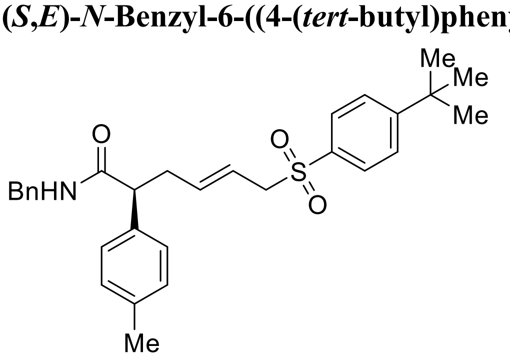

Colorless oil (70% yield, E/Z = 5:1). ^1H NMR (400 MHz, $CDCl_3$) δ 7.61 (d, *J* = 8.6 Hz, 2H), 7.44 (d, *J* = 8.6 Hz, 2H), 7.22–7.15 (m, 3H), 7.07–7.04 (m, 6H), 5.64 (t, *J* = 5.8 Hz, 1H), 5.52–5.42 (m, 1H), 5.40–5.31 (m, 1H), 4.34 (dd, *J* = 14.9, 5.7 Hz, 1H), 4.25 (dd, *J* = 15.0, 5.7 Hz, 1H), 3.57 (d, *J* = 7.5 Hz, 2H), 3.26 (t, *J* = 7.5 Hz, 1H), 2.89–2.79 (m, 1H), 2.47–2.39 (m, 1H), 2.26 (s, 3H), 1.27 (s, 9H). ^{13}C NMR (101 MHz, CDCl3) δ 172.7, 172.6, 157.8, 157.7, 138.6, 138.3, 137.4, 136.7, 136.1, 136.0, 135.9, 135.7, 129.8, 129.7, 128.7, 128.4, 128.4, 128.0, 127.9,

127.7, 127.7, 127.5, 126.3, 126.2, 118.4, 117.3, 60.1, 55.3, 52.5, 52.4, 43.7, 36.3, 35.4, 31.4, 31.2, 31.2, 29.8, 27.0, 21.2, 21.2. IR (v/cm-1): 3304, 2958, 2928, 2868, 1450, 1512, 1315, 1290, 1152, 1107, 1084, 841, 736, 699, 573, 507. HRMS (ESI-MS): calcd for $C_{30}H_{36}NO_3S$ $[M+H]^+$ 490.2410, found: 490.2409. $[\alpha]_D^{25}$ = -26.44 (c = 0.01, $CHCl_3$). HPLC conditions: IC column, *n*-Hexane/*i*-PrOH = 70/30, 1.0 mL/min, t_{major} = 34.2 min, t_{minor} = 37.5 min, t_{major} = 44.3 min, t_{minor} = 48.9 min, 99:1 er.

(*S*,*E*)-*N*-Benzyl-6-((4-methoxyphenyl)sulfonyl)-2-(*p*-tolyl)hex-4-enamide (2.16)

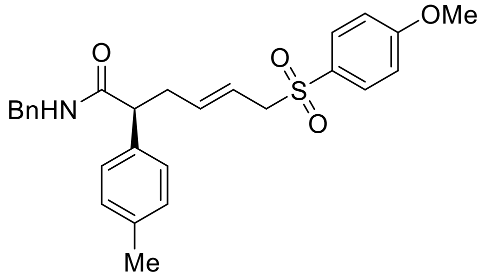

Colorless oil (69% yield, E/Z = 6:1). ^{1}H NMR (400 MHz, $CDCl_3$) δ 7.59 (d, *J* = 8.9 Hz, 2H), 7.20–7.16 (m, 3H), 7.06–7.02 (m, 6H), 6.87 (d, *J* = 8.9 Hz, 2H), 5.65 (t, *J* = 5.8 Hz, 1H), 5.44–5.29 (m, 2H), 4.32 (dd, *J* = 14.9, 5.7 Hz, 1H), 4.24 (dd, *J* = 14.9, 5.7 Hz, 1H), 3.79 (s, 3H), 3.54 (d, *J* = 6.7 Hz, 2H), 3.24 (t, *J* = 7.5 Hz, 1H), 2.86–2.77 (m, 1H), 2.44–2.36 (m, 1H), 2.25 (s, 3H). ^{13}C NMR (101 MHz, $CDCl_3$) δ 172.7, 172.6, 163.8, 163.8, 138.5, 138.3, 137.4, 136.5, 136.1, 135.9, 130.7, 130.4, 130.1, 129.8, 129.7, 128.7, 128.0, 127.9, 127.7, 127.5, 118.6, 117.5, 114.4, 114.3, 60.4, 55.8, 55.5, 52.5, 52.3, 43.7, 36.3, 31.3, 21.2. IR (v/cm^{-1}): 3308, 2921, 2856, 1650, 1595, 1498, 1296, 1260, 1135, 1089, 1027, 704, 506. HRMS (ESI-MS): calcd for $C_{27}H_{30}NO_4S$ $[M+H]^+$ 464.1890, found: 464.1900. $[\alpha]_D^{25}$ = 50.37 (c = 0.07, $CHCl_3$). HPLC conditions: AD-H column, *n*-Hexane/*i*-PrOH = 80/20, 1.0 mL/min, t_{major} = 26.8 min, t_{major} = 31.1 min, t_{minor} = 73.4 min, t_{minor} = 79.8 min, 99:1 er.

(*S*,*E*)-*N*-Benzyl-6-(phenylsulfonyl)-2-(*p*-tolyl)hex-4-enamide (2.17)

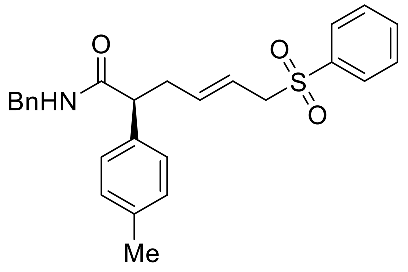

Colorless oil (77% yield, E/Z = 5:1). ^{1}H NMR (500 MHz, $CDCl_3$) δ 7.69 (d, *J* = 7.3 Hz, 2H), 7.54 (t, *J* = 7.4 Hz, 1H), 7.46–7.41 (m, 2H), 7.20–7.16 (m, 3H), 7.08–7.02 (m, 6H), 5.60 (t, *J* = 6.2 Hz, 1H), 5.45–5.38 (m, 1H), 5.38–5.31 (m, 1H), 4.32 (dd, *J* = 14.8, 5.8 Hz, 1H), 4.25 (dd, *J* = 15.0, 5.8 Hz, 1H), 3.58 (d, *J* = 6.9 Hz, 2H), 3.22 (t, *J* = 7.5 Hz, 1H), 2.86–2.78 (m, 1H), 2.44–2.36 (m, 1H), 2.26 (s, 3H). ^{13}C NMR (126 MHz, $CDCl_3$) δ 172.6, 172.6, 138.9, 138.6, 138.3, 137.4, 137.4, 136.8, 136.0, 135.9, 133.8, 133.7, 129.8, 129.7, 129.3, 129.1, 128.8, 128.6, 128.0, 127.9, 127.7, 127.5, 118.2, 117.1, 60.1, 55.3, 52.5, 52.3, 43.7, 43.6, 36.3, 31.3, 27.0, 21.2. IR (v/cm^{-1}): 3301, 3061, 3031, 2921, 1649, 1533, 1511, 1448, 1305, 1238, 1150, 1136, 1085, 970, 729, 689, 598, 557, 530, 509. HRMS (ESI-MS): calcd for $C_{26}H_{28}NO_3S$ $[M+H]^+$ 434.1784, found: 434.1786. $[\alpha]_D^{25}$ = 67.00 (c = 0.07, $CHCl_3$). HPLC conditions: IC column, *n*-Hexane/*i*-PrOH = 70/30, 1.0 mL/min, t_{major} = 39.2 min, t_{minor} = 43.5 min, t_{major} = 47.7 min, t_{minor} = 57.2 min, 98:2 er.

(*S*,*E*)-*N*-Benzyl-6-((4-fluorophenyl)sulfonyl)-2-(*p*-tolyl)hex-4-enamide (2.18)

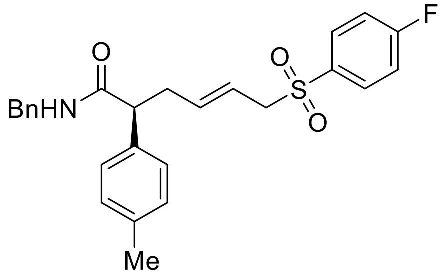

Colorless oil (80% yield, E/Z = 7:1). ^{1}H NMR (400 MHz, $CDCl_3$) δ 7.80–7.73 (m, 2H), 7.30–7.26 (m, 3H), 7.20–7.12 (m, 8H), 5.70 (t, *J* = 6.0 Hz, 1H), 5.56–5.48 (m, 1H), 5.48–5.39 (m, 1H), 4.42 (dd, *J* = 14.8, 5.9 Hz, 1H), 4.35 (dd, *J* = 15.1, 5.8 Hz, 1H), 3.67 (d, *J* = 6.9 Hz, 2H), 3.35 (t, *J* = 7.5 Hz, 1H), 2.97–2.88 (m, 1H), 2.57–2.47 (m, 1H), 2.36 (s, 3H). ^{13}C NMR (101 MHz, $CDCl_3$) δ 172.6, 172.5, 165.9 (d, *J* = 257.2 Hz), 165.9 (d, *J* = 257.1 Hz), 139.1, 138.3, 137.5, 137.5, 136.9, 135.9, 135.8, 134.8 (d, *J* = 3.4 Hz), 134.5 (d, *J* = 3.0 Hz), 131.5 (d, *J* = 9.6 Hz), 129.8, 129.8, 128.8, 128.0, 127.9, 127.6, 127.5, 116.6 (d, *J* = 22.7 Hz), 116.4 (d, *J* = 22.7 Hz), 60.2, 55.3, 52.3, 52.2, 43.7, 36.2, 31.3, 29.8, 22.8, 21.2. ^{19}F NMR (376 MHz, $CDCl_3$) δ -103.4, -103.5. IR (v/cm^{-1}): 3301, 3068, 3023, 2917, 1649, 1590, 1511, 1493, 1317, 1289, 1229, 1136, 1085, 970, 840, 818, 733, 698, 558, 513. HRMS (ESI-MS): calcd for $C_{26}H_{27}FNO_3S$ $[M+H]^+$ 452.1690, found: 452.1689. $[\alpha]_D^{25}$ = 53.76 (c = 0.10, $CHCl_3$). HPLC conditions: IC column, *n*-Hexane/*i*-PrOH = 70/30, 1.0 mL/min, t_{major} = 25.0 min, t_{minor} = 27.9 min, t_{major} = 31.5 min, t_{minor} = 35.6 min, 99:1 er.

(*S*,*E*)-*N*-Benzyl-2-(*p*-tolyl)-6-(*m*-tolylsulfonyl)hex-4-enamide (2.19)

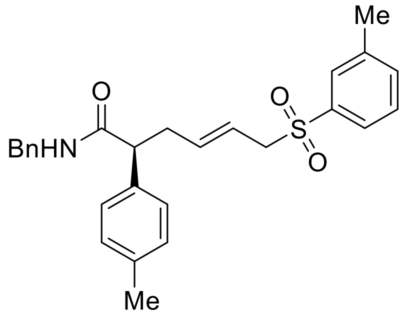

Colorless oil (78% yield, E/Z = 6:1). ^{1}H NMR (400 MHz, $CDCl_3$) δ 7.63 (s, 1H), 7.55 (dt, *J* = 7.4, 1.9 Hz, 1H), 7.44–7.37 (m, 2H), 7.30–7.23 (m, 4H), 7.13–7.10 (m, 5H), 5.67 (t, *J* = 5.9 Hz, 1H), 5.53–5.41(m, 2H), 4.42–4.29 (m, 2H), 3.65 (d, *J* = 6.7 Hz, 2H), 3.30 (t, *J* = 7.5 Hz, 1H), 2.93–2.85 (m, 1H), 2.52–2.44 (m, 1H), 2.43 (s, 3H), 2.33 (s, 3H). ^{13}C NMR (101 MHz, $CDCl_3$) δ 172.6, 172.6, 139.6, 139.4, 138.8, 138.8, 138.5, 138.3, 137.4, 137.4, 136.7, 136.1, 135.9, 134.6, 134.5, 129.8, 129.7, 129.1, 129.0, 128.8, 128.8, 128.0, 127.9, 127.7, 127.5, 125.7, 125.7, 118.2, 117.2, 60.1, 55.2, 52.5, 52.3, 43.7, 36.3, 31.3, 29.8, 21.5, 21.2. IR (v/cm-1): 3301, 3031, 2925, 2856, 1650, 1537, 1511, 1298, 1132, 1083, 726, 688, 506. HRMS (ESI-MS): calcd for $C_{27}H_{30}NO_3S$ $[M+H]^+$ 448.1941, found: 448.1942. $[\alpha]_D^{25}$ = 65.05 (c = 0.08, $CHCl_3$). HPLC conditions: IC column, *n*-Hexane/*i*-PrOH = 70/30, 1.0 mL/min, t_{major} = 35.8 min, t_{minor} = 39.0 min, t_{major} = 43.0 min, t_{minor} = 50.8 min, 99:1 er.

(*S*,*E*)-*N*-Benzyl-2-(*p*-tolyl)-6-(*o*-tolylsulfonyl)hex-4-enamide (2.20)

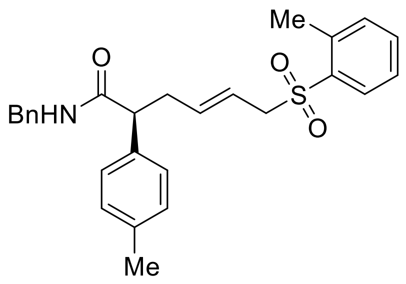

Colorless oil (74% yield, E/Z = 6:1). ^{1}H NMR (500 MHz, $CDCl_3$) δ 7.87 (d, *J* = 7.9 Hz, 1H), 7.50 (t, *J* = 7.4 Hz, 1H), 7.35 (t, *J* = 7.7 Hz, 1H), 7.32–7.26 (m, 4H), 7.16–7.10 (m, 6H), 5.68 (t, *J* = 6.2 Hz, 1H), 5.57–5.49 (m, 1H), 5.47–5.39 (m, 1H), 4.42 (dd, *J* = 15.1, 5.8 Hz, 1H), 4.34 (dd, *J* = 15.0, 5.7 Hz, 1H), 3.73 (d, *J* = 7.1 Hz, 2H), 3.25 (t, *J* = 7.5 Hz, 1H),

2.92–2.85 (m, 1H), 2.66 (s, 3H), 2.49–2.42 (m, 1H), 2.35 (s, 3H). ^{13}C NMR (126 MHz, $CDCl_3$) δ 172.6, 172.6, 138.7, 138.4, 138.3, 138.3, 138.2, 137.4, 137.4, 136.8, 136.7, 136.0, 136.0, 133.8, 133.7, 132.9, 132.7, 130.9, 130.8, 129.8, 129.7, 128.8, 127.9, 127.9, 127.7, 127.5, 126.6, 126.5, 118.1, 117.0, 59.4, 54.3, 52.6, 52.3, 43.7, 36.3, 31.4, 21.2, 20.6. IR (v/cm^{-1}): 3309, 3058, 3027, 2928, 2860, 1649, 1537, 1511, 1454, 1309, 1240, 1151, 1126, 807, 730, 699, 605, 560, 527, 508, 479. HRMS (ESI-MS): calcd for $C_{27}H_{30}NO_3S$ [M+H]$^+$ 448.1941, found: 448.1941. $[\alpha]_D^{25}$ = 88.25 (c = 0.03, $CHCl_3$). HPLC conditions: AD-H column, *n*-Hexane/*i*-PrOH = 80/20, 1.0 mL/min, t_{major} = 16.8 min, t_{major} = 19.9 min, t_{minor} = 31.6 min, t_{minor} = 36.3 min, 99:1 er.

(*S*,*E*)-*N*-Benzyl-6-(thiophen-2-ylsulfonyl)-2-(*p*-tolyl)hex-4-enamide (2.21)

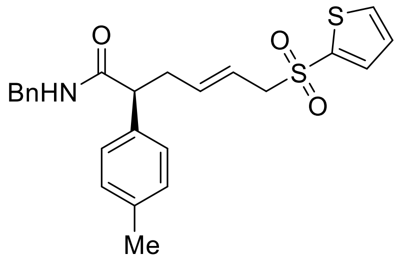

Colorless oil (68% yield, E/Z = 6:1). ^{1}H NMR (400 MHz, $CDCl_3$) δ 7.66 (dd, *J* = 5.0, 1.4 Hz, 1H), 7.51 (dd, *J* = 3.7, 1.4 Hz, 1H), 7.29–7.24 (m, 3H), 7.13 (d, *J* = 2.2 Hz, 6H), 7.09 (dd, *J* = 5.0, 3.7 Hz, 1H), 5.70 (t, *J* =5.4Hz, 1H), 5.61–5.44 (m, 2H), 4.41 (dd, *J* = 14.9, 5.8 Hz, 1H), 4.32 (dd, *J* = 15.0, 5.7 Hz, 1H), 3.74 (d, *J* = 6.7 Hz, 2H), 3.34 (t, *J* = 7.6 Hz, 1H), 2.98–2.89 (m, 1H), 2.51–2.47 (m, 1H), 2.33 (s, 3H). ^{13}C NMR (101 MHz, $CDCl_3$) δ 172.6, 172.5, 139.7, 139.4, 139.2, 138.3, 137.4, 137.2, 136.0, 135.9, 134.6, 134.6, 134.3, 134.1, 129.8, 129.8, 128.8, 128.0, 127.9, 127.9, 127.7, 127.6, 118.3, 117.2, 61.3, 56.5, 52.5, 52.3, 43.7, 36.3, 31.3, 27.0, 21.2. IR (v/cm^{-1}): 3297, 3091, 3027, 2924, 1648, 1533, 1510, 1315, 1232, 1132, 1015, 724, 698, 578, 533, 508, 455. HRMS (ESI-MS): calcd for $C_{24}H_{25}NNaO_3S_2$ [M+Na]$^+$ 462.1168, found: 462.1168. $[\alpha]_D^{25}$ = 57.36 (c = 0.09, $CHCl_3$). HPLC conditions: IC column, *n*-Hexane/*i*-PrOH = 70/30, 1.0 mL/min, t_{major} = 30.8 min, t_{minor} = 36.9 min, t_{major} = 40.2 min, t_{minor} = 47.0 min, 99:1 er.

(*S*,*E*)-*N*-Benzyl-6-(((*E*)-styryl)sulfonyl)-2-(*p*-tolyl)hex-4-enamide (2.22)

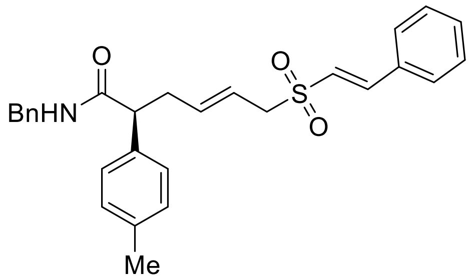

Colorless oil (50% yield, E/Z = 6:1). ^{1}H NMR (500 MHz, $CDCl_3$) δ 7.56–7.49 (m, 3H), 7.47–7.42 (m, 3H), 7.30–7.26 (m, 3H), 7.16–7.12 (m, 6H), 6.74 (d, *J* = 15.5 Hz, 1H), 5.78–5.72 (m, 1H), 5.68 (t, *J* = 5.8 Hz, 1H), 5.79–5.72 (m, 1H), 4.40 (dd, *J* = 14.8, 5.8 Hz, 1H), 4.34 (dd, *J* = 15.1, 5.6 Hz, 1H), 3.68 (d, *J* = 7.4 Hz, 2H), 3.40 (t, *J* = 7.6 Hz, 1H), 3.03–2.96 (m, 1H), 2.62–2.55 (m, 1H), 2.33 (s, 3H). ^{13}C NMR (126 MHz, $CDCl_3$) δ 172.7, 172.7, 145.4, 139.0, 138.4, 138.4, 137.6, 137.5, 136.8, 136.1, 136.0, 132.5, 132.4, 131.6, 131.6, 129.9, 129.9, 129.4, 128.9, 128.8, 128.1, 128.0, 127.7, 127.6, 124.8, 124.4, 118.5, 117.2, 59.2, 54.3, 52.6, 52.5, 43.8, 36.4, 31.8, 21.3. IR (v/cm^{-1}): 3312, 3058, 3031, 2921, 2856, 1650, 1533, 1511, 1300, 1122, 973, 823, 731, 698, 505. HRMS (ESI-MS): calcd for $C_{28}H_{29}NNaO_3S$ [M+Na]$^+$ 482.1760, found: 482.1759. $[\alpha]_D^{25}$ = 86.16 (c = 0.07, $CHCl_3$). HPLC conditions: AD-H column, *n*-Hexane/*i*-PrOH = 70/30, 1.0 mL/min, t_{major} = 10.3 min, t_{major} = 13.6 min, t_{minor} = 21.4 min, t_{minor} = 26.0 min, 99:1 er.

(*S*,*E*)-*N*-Benzyl-6-(cyclopropylsulfonyl)-2-(*p*-tolyl)hex-4-enamide (2.23)

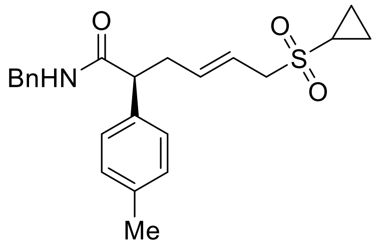

Colorless oil (75% yield, E/Z = 7:1). ^{1}H NMR (400 MHz, $CDCl_3$) δ 7.23–7.16 (m, 3H), 7.12–7.04 (m, 6H), 5.80–5.62 (m, 2H), 5.58–5.47 (m, 1H), 4.34 (dd, *J* = 14.9, 5.8 Hz, 1H), 4.26 (dd, *J* = 14.9, 5.6 Hz, 1H), 3.56–3.45 (m, 2H), 3.39 (dd, *J* = 8.4, 6.9 Hz, 1H), 2.97–2.89 (m, 1H), 2.61–2.52 (m, 1H), 2.25 (s, 3H), 1.99–1.91 (m, 1H), 1.10–1.04 (m, 1H), 1.03–0.97 (m, 1H), 0.79–0.77 (m, 1H), 0.77–0.75 (m, 1H). ^{13}C NMR (101 MHz, $CDCl_3$) δ 172.6, 172.6, 138.3, 138.3, 137.5, 136.4, 136.1, 135.9, 129.8, 129.8, 128.8, 128.7, 128.1, 128.0, 127.6, 127.6, 127.5, 119.0, 117.4, 57.7, 52.8, 52.5, 52.4, 43.7, 36.1, 31.8, 29.8, 29.0, 27.9, 21.2, 4.9, 4.8, 4.5. IR (v/cm^{-1}): 3304, 3051, 2921, 2856, 1736, 1650, 1541, 1512, 1288, 1240, 1188, 1125, 1042, 889, 723, 698, 502. HRMS (ESI-MS): calcd for $C_{23}H_{28}NO_3S$ $[M+H]^+$ 398.1784, found: 398.1784. $[\alpha]_D^{25}$ = 69.43 (c = 0.08, $CHCl_3$). HPLC conditions: IC column, *n*-Hexane/*i*-PrOH = 70/30, 1.0 mL/min, t_{majorr} = 28.7 min, t_{minor} = 37.2 min, t_{minor} = 44.6 min, t_{major} = 52.4 min, 99:1 er.

(*S*,*E*)-*N*-Benzyl-6-(propylsulfonyl)-2-(*p*-tolyl)hex-4-enamide (2.24)

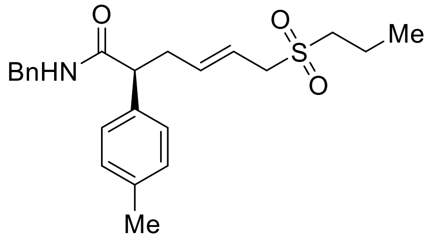

Colorless oil (51% yield, E/Z = 6:1). ^{1}H NMR (500 MHz, $CDCl_3$) δ 7.32–7.25 (m, 4H), 7.20–7.15 (m, 5H), 5.81–5.70 (m, 2H), 5.61–5.54 (m, 1H), 4.43 (dd, *J* = 14.9, 5.8 Hz, 1H), 4.37 (dd, *J* = 14.9, 5.8 Hz, 1H), 3.55 (d, *J* = 7.4 Hz, 2H), 3.49 (t, *J* = 7.6 Hz, 1H), 3.05–2.98 (m, 1H), 2.71–2.61 (m, 3H), 2.35 (s, 3H), 1.80–1.74 (m, 2H), 1.03 (t, *J* = 7.4 Hz, 3H). ^{13}C NMR (126 MHz, $CDCl_3$) δ 172.6, 138.4, 138.3, 137.6, 136.4, 136.1, 135.8, 129.9, 129.8, 128.8, 128.8, 128.1, 128.0, 127.7, 127.6, 127.6, 127.6, 119.1, 117.4, 56.9, 53.7, 52.5, 52.4, 52.4, 52.1, 43.8, 43.8, 36.1, 31.9, 21.2, 15.7, 15.6, 13.3, 13.2. IR (v/cm^{-1}): 3312, 3027, 2974, 2925, 2878, 1650, 1533, 1511, 1454, 1286, 1245, 1123, 974, 731, 699, 507. HRMS (ESI-MS): calcd for $C_{23}H_{30}O_3NS$ $[M+H]^+$ 400.1941, found: 400.1940. $[\alpha]_D^{25}$ = 70.12 (c = 0.09, $CHCl_3$). HPLC conditions: IC column, *n*-Hexane/*i*-PrOH = 70/30, 1.0 mL/min, t_{minor} = 42.1 min, t_{minor}= 46.1 min, t_{majorr} = 50.4 min, t_{major} = 57.9 min, 99:1 er.

Ethyl (*S*,*E*)-8-(benzylamino)-2,2-difluoro-8-oxo-7-(*p*-tolyl)oct-4-enoate (2.25)

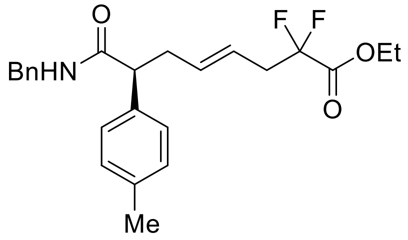

Colorless oil (58% yield, E/Z = 5:1). ^{1}H NMR (500 MHz, $CDCl_3$) δ 7.23–7.14 (m, 4H), 7.12–7.04 (m, 5H), 5.61 (t, *J* = 5.9 Hz, 1H), 5.53–5.48 (m, 1H), 5.36–5.28 (m, 1H), 4.39–4.33 (m, 1H), 4.29–4.22 (m, 1H), 4.21–4.16 (m, 2H), 3.29 (t, *J* = 7.6 Hz, 1H), 2.92–2.80 (m, 1H), 2.62 (td, *J* = 16.1, 7.1 Hz, 2H), 2.48–2.41 (m, 1H), 2.26 (s, 3H), 1.24 (t, *J* = 8.2 Hz, 3H). ^{13}C NMR (101MHz, $CDCl_3$) δ 172.9, 164.0 (t, *J* = 33.0 Hz), 138.3, 138.3, 137.4, 137.3, 136.2, 136.2, 135.3, 133.4, 129.8, 129.7, 128.8, 128.0, 127.7, 127.5, 120.7 (t, *J* = 5.3 Hz), 119.6 (t, *J* = 5.3 Hz), 115.4 (t, *J* = 251.4 Hz), 63.0, 62.9, 53.0, 52.6, 43.8, 43.7, 38.1 (t, *J* = 24.1 Hz),

36.3, 33.0 (t, J = 23.6 Hz), 31.3, 29.8, 21.2, 14.3, 14.1. ^{19}F NMR (376 MHz, $CDCl_3$) δ -105.3, -105.3, -105.6. IR (v/cm^{-1}): 3289, 2928, 2856, 1759, 1646, 1512, 1454, 1373, 1219, 1187, 1083, 1032, 973, 749, 698, 511. HRMS (ESI-MS): calcd for $C_{24}H_{28}F_2NO_3$ $[M+H]^+$ 416.2032, found: 416.2031. $[\alpha]_D^{25}$ = 39.23 (c = 0.09, $CHCl_3$). HPLC conditions: AD-3 column, *n*-Hexane/*i*-PrOH = 90/10, 1.0 mL/min, t_{major} = 16.7 min, t_{major} = 18.5 min, t_{minor} = 33.3 min, t_{minor} = 56.9 min, 99:1 er.

Cyclohexyl (*S,E*)-8-(benzylamino)-2,2-difluoro-8-oxo-7-(*p*-tolyl)oct-4-enoate (2.26)

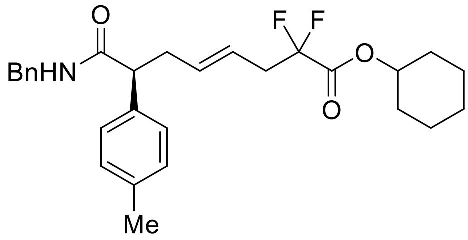

Colorless oil (53% yield, E/Z = 5:1). ^{1}H NMR (500 MHz, $CDCl_3$) δ 7.30–7.24 (m, 3H), 7.18–7.11 (m, 6H), 5.67 (t, J = 6.0 Hz, 1H), 5.61–5.54 (m, 1H), 5.43–5.34 (m, 1H), 4.90–4.83 (m, 1H), 4.49–4.27 (m, 2H), 3.36 (t, J = 7.6 Hz, 1H), 2.94–2.87 (m, 1H), 2.67 (dt, J = 16.0, 7.1 Hz, 2H), 2.56–2.47 (m, 1H), 2.33 (s, 3H), 1.84–1.82 (m, 2H), 1.77–1.71 (m, 2H), 1.56–1.44 (m, 3H), 1.41–1.27 (m, 3H). ^{13}C NMR (126 MHz, $CDCl_3$) δ 172.9, 163.5 (t, J = 32.4 Hz), 163.4 (t, J = 32.8 Hz), 138.4, 138.3, 137.4, 137.3, 136.3, 136.2, 135.2, 133.3, 129.8, 129.7, 128.8, 128.0, 127.7, 127.5, 120.9 (t, J = 5.5 Hz), 119.8 (t, J = 5.3 Hz), 115.5 (t, J = 251.1 Hz), 115.4 (t, J = 251.3 Hz), 75.9, 75.7, 53.0, 52.7, 43.8, 43.7, 38.2 (t, J = 24.1 Hz), 36.3, 33.2 (t, J = 24.1 Hz), 31.4, 31.3, 31.3, 25.3, 23.6, 21.2. ^{19}F NMR (376 MHz, $CDCl_3$) δ -105.3, -105.3, -105.7, -105.7. IR (v/cm^{-1}): 3301, 2935, 2860, 1752, 1647, 1537, 1512, 1454, 1355, 1225, 1187, 1083, 1037, 973, 905, 749, 698, 506. HRMS (ESI-MS): calcd for $C_{28}H_{34}F_2NO_3$ $[M+H]^+$ 470.2501, found: 470.2498. $[\alpha]_D^{25}$ = 37.71 (c = 0.06, $CHCl_3$). HPLC conditions: IC-3 column, *n*-Hexane/*i*-PrOH = 90/10, 1.0 mL/min, t_{major} = 20.6 min, t_{major} = 22.4 min, t_{minor} = 23.7 min, t_{minor} = 31.1 min, 98:2 er.

Benzyl (*S,E*)-8-(benzylamino)-2,2-difluoro-8-oxo-7-(*p*-tolyl)oct-4-enoate (2.27)

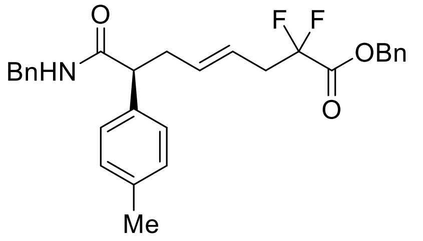

Colorless oil (40% yield, E/Z = 7:1). ^{1}H NMR (500 MHz, $CDCl_3$) δ 7.39–7.32 (m, 5H), 7.29–7.23 (m, 3H), 7.16–7.11 (m, 6H), 5.62 (t, J = 5.9 Hz, 1H), 5.59–5.47 (m, 1H), 5.38–5.32 (m, 1H), 5.23 (s, 2H), 4.47–4.26 (m, 2H), 3.29 (t, J = 7.7 Hz, 1H), 2.89–2.82 (m, 2H), 2.86 (dt, J = 14.9, 7.4 Hz, 1H), 2.48–2.42 (m, 1H), 2.32 (s, 3H). ^{13}C NMR (126 MHz, $CDCl_3$) δ 172.7, 163.8 (t, J = 33.2 Hz), 138.3, 138.2, 137.3, 137.2, 136.2, 136.1, 135.4, 134.4, 134.3, 133.5, 129.7, 129.6, 128.9, 128.9, 128.8, 128.8, 128.7, 128.5, 128.5, 127.9, 127.6, 127.4, 120.5 (t, J = 5.4 Hz), 119.3 (t, J = 5.3 Hz), 115.5 (t, J = 251.6 Hz), 115.4 (t, J = 251.6 Hz), 68.3, 68.2, 52.9, 52.5, 43.7, 43.6, 38.06 (t, J = 23.9 Hz), 36.1, 33.04 (t, J = 23.9 Hz), 31.2, 21.1. ^{19}F NMR (376 MHz, $CDCl_3$) δ -105.0, -105.0, -105.3. IR (v/cm^{-1}): 3297, 2925, 2852, 1762, 1646, 1512, 1455, 1217, 1179, 1082, 1029, 973, 750, 697, 603, 511. HRMS (ESI-MS): calcd for $C_{29}H_{30}F_2NO_3$ $[M+H]^+$ 478.2188, found: 478.2186. $[\alpha]_D^{25}$ = 52.93 (c = 0.03, $CHCl_3$). HPLC conditions: IC-3 column, *n*-Hexane/*i*-PrOH = 95/5, 1.0 mL/min, t_{major} = 58.3 min, t_{major} = 61.2

min, t_{minor} = 65.1 min, t_{minor} = 80.8 min, 99:1 er.

(*S,E*)-*N*^{8}-Benzyl-2,2-difluoro-*N*^{1}-phenyl-7-(*p*-tolyl)oct-4-enediamide (2.28)

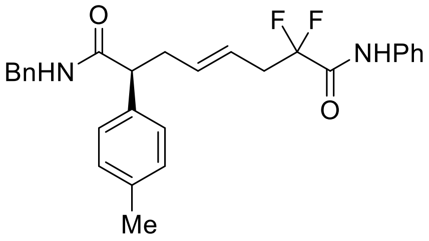

Yellow solid (53% yield, E/Z = 7:1). M.p.: 135.2-138.6 °C. ^{1}H NMR (400 MHz, $CDCl_3$) δ 7.96 (s, 1H), 7.62–7.54 (m, 2H), 7.37–7.32 (m, 2H), 7.28–7.24 (m, 4H), 7.20–7.15 (m, 1H), 7.13–7.09 (m, 5H), 5.71–5.54 (m, 2H), 5.48–5.40 (m, 1H), 4.42–4.26 (m, 2H), 3.31 (t, *J* = 7.4 Hz, 1H), 2.98–2.74 (m, 3H), 2.54–2.45 (m, 1H), 2.31 (s, 3H). ^{13}C NMR (126 MHz, $CDCl_3$) δ 172.9, 172.7, 161.9 (t, *J* = 28.5 Hz), 138.2, 138.1, 137.2, 137.2, 136.3, 136.2, 136.2, 136.1, 135.6, 133.8, 129.6, 129.2, 128.7, 127.9, 127.9, 127.6, 127.5, 125.6, 125.5, 120.8 (t, *J* = 5.4 Hz), 120.3, 120.2, 119.6, 117.2 (t, *J* = 254.7 Hz), 52.8, 52.6, 43.7, 43.6, 37.4 (t, *J* = 24.1 Hz), 36.4, 32.6 (t, *J* = 24.2 Hz), 31.4, 21.09. ^{19}F NMR (376 MHz, $CDCl_3$) *δ* -104.7 (d, *J* = 249.7 Hz), 105.1 (d, *J* = 250.0 Hz), -105.5 (d, *J* = 250.0 Hz), -105.9 (d, *J* = 249.7 Hz). IR (v/cm^{-1}): 3297, 2928, 2848, 1650, 1540, 1447, 1260, 1183, 1079, 1030, 750, 693, 505. HRMS (ESI-MS): calcd for $C_{28}H_{29}F_2N_2O_2$ $[M+H]^+$ 463.2192, found: 463.2196. $[\alpha]_D^{25}$ = 30.54 (c = 0.05, $CHCl_3$). HPLC conditions: AD-H column, *n*-Hexane/*i*-PrOH = 80/20, 1.0 mL/min, t_{major} = 7.5 min, t_{major} = 8.6 min, t_{minor} = 11.0 min, t_{minor} = 15.0 min, 98:2 er.

(*S,E*)-*N*^{1},*N*^{8}-Dibenzyl-2,2-difluoro-7-(*p*-tolyl)oct-4-enediamide (2.29)

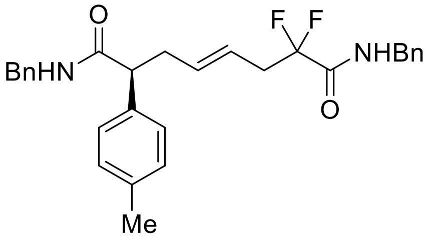

Yellow oil (61% yield, E/Z = 6:1). ^{1}H NMR (500 MHz, $CDCl_3$) δ 7.34–7.22 (m, 8H), 7.19–7.10 (m, 6H), 6.59 (t, *J* = 6.2 Hz, 1H), 5.73 (t, *J* = 5.8 Hz, 1H), 5.63–5.55 (m, 1H), 5.40–5.34 (m, 1H), 4.49–4.38 (m, 3H), 4.34–4.28 (m, 1H), 3.34 (t, *J* = 7.5 Hz, 1H), 2.91–2.85 (m, 1H), 2.74 (td, *J* = 16.5, 7.2 Hz, 2H), 2.56–2.44 (m, 1H), 2.31 (s, 3H). ^{13}C NMR (126 MHz, $CDCl_3$) δ 172.7, 163.9 (t, *J* = 32.8 Hz), 163.8 (t, *J* = 33.0 Hz), 163.5, 138.3, 138.2, 137.3, 137.2, 136.2, 136.1, 135.4, 134.4, 134.3, 133.5, 129.7, 129.7, 128.9, 128.9, 128.8, 128.8, 128.7, 128.5, 128.5, 127.9, 127.6, 127.4, 120.5 (t, *J* = 5.3 Hz), 119.4 (t, *J* = 5.2 Hz), 115.5 (t, *J* = 251.5 Hz), 115.4 (t, *J* = 251.1 Hz), 68.3, 68.2, 52.9, 52.5, 43.7, 43.6, 38.1 (t, *J* = 23.8 Hz), 36.1, 33.1 (t, *J* = 23.8 Hz), 31.2, 21.1. ^{19}F NMR (376 MHz, $CDCl_3$) *δ* -105.4, -106.1. IR (v/cm^{-1}): 3285, 2923, 2852, 1739, 1692, 1656, 1538, 1454, 1188, 1032, 750, 697, 510. HRMS (ESI-MS): calcd for $C_{29}H_{31}F_2N_2O_2$ $[M+H]^+$ 477.2357, found: 477.2352. $[\alpha]_D^{25}$ = 37.66 (c = 0.08, $CHCl_3$). HPLC conditions: AD-H column, *n*-Hexane/*i*-PrOH = 80/20, 1.0 mL/min, t_{major} = 7.8 min, t_{major} = 9.4 min, t_{minor} = 12.3 min, t_{minor} = 15.1 min, 98:2 er.

(*S,E*)-*N*[8]-Benzyl-*N*[1]-cyclopentyl-2,2-difluoro-7-(*p*-tolyl)oct-4-enediamide (2.30)

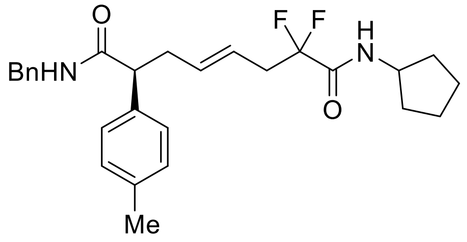

Yellow solid (59% yield, E/Z = 6:1). M.p.: 110.4-114.1 °C. ^{1}H NMR (400 MHz, $CDCl_3$) δ 7.30–7.23 (m, 3H), 7.19–7.10 (m, 6H), 6.20 (d, *J* = 7.7 Hz, 1H), 5.71 (t, *J* = 5.8 Hz, 1H), 5.73–5.53 (m, 1H), 5.43–5.31 (m, 1H), 4.47–4.27 (m, 2H), 4.22–4.13 (m, 1H), 3.37 (t, *J* = 7.4 Hz, 1H), 2.93–2.85 (m, 1H), 2.71 (td, *J* = 16.7, 7.1 Hz, 2H), 2.57–2.45 (m, 1H), 2.33 (s, 3H), 2.03–1-95 (m, 2H), 1.72–1.60 (m, 4H), 1.46–1.36 (m, 2H). ^{13}C NMR (126 MHz, $CDCl_3$) δ 172.9, 172.8, 163.5 (t, *J* = 28.2 Hz), 163.5 (t, *J* = 28.3 Hz), 138.3, 137.2, 137.2, 136.2, 136.2, 134.9, 133.2, 129.6, 129.6, 128.7, 128.6, 128.0, 127.9, 127.6, 127.4, 127.4, 121.1 (t, *J* = 5.3 Hz), 120.0 (t, *J* = 5.3 Hz), 117.3 (t, *J* = 253.3 Hz), 117.1 (t, *J* = 253.5 Hz), 52.9, 52.6, 51.3, 51.3, 43.7, 43.6, 37.5 (t, *J* = 24.4 Hz), 36.3, 32.9, 32.9, 32.5 (t, *J* = 24.1 Hz), 31.3, 23.7, 21.1. ^{19}F NMR (376 MHz, $CDCl_3$) *δ* -105.6, -105.7, -106.0 (d, *J* = 249.3 Hz), -106.8 (d, *J* = 248.9 Hz). IR (v/cm^{-1}): 3308, 2958, 2920, 2871, 1735, 1656, 1543, 1369, 1276, 750, 506. HRMS (ESI-MS): calcd for $C_{27}H_{33}F_2N_2O_2$ $[M+H]^+$ 455.2505, found: 455.2502. $[\alpha]_D^{25}$ = 36.81 (c = 0.06, $CHCl_3$). HPLC conditions: AD-H column, *n*-Hexane/*i*-PrOH = 90/10, 1.0 mL/min, t_{major} = 13.0 min, t_{major} = 16.2 min, t_{minor} = 25.8 min, t_{minor} = 28.9 min, 98:2 er.

(*S,E*)-*N*-Benzyl-7,7-difluoro-8-oxo-8-(piperidin-1-yl)-2-(*p*-tolyl)oct-4-enamide (2.31)

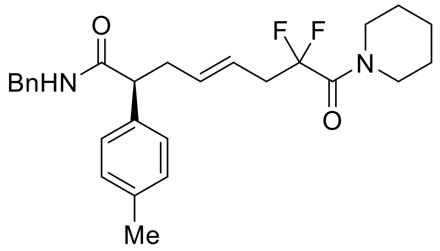

Colorless oil (58% yield, E/Z = 5:1). ^{1}H NMR (500 MHz, $CDCl_3$) δ 7.30–7.25 (m, 3H), 7.19–7.12 (m, 6H), 5.72 (t, *J* = 5.8 Hz, 1H), 5.61–5.47 (m, 2H), 4.48–4.31 (m, 2H), 3.62–3.59 (m, 2H), 3.55–3.51 (m, 2H), 3.38 (t, *J* = 7.6 Hz, 1H), 2.96–2.88 (m, 1H), 2.77 (td, *J* = 18.0, 5.8 Hz, 2H), 2.56–2.47 (m, 1H), 2.32 (s, 3H), 1.69–1.64 (m, 2H), 1.61–1.57 (m, 4H). ^{13}C NMR (126 MHz, $CDCl_3$) δ 173.0, 172.9, 161.6 (t, *J* = 28.8 Hz), 161.5 (t, *J* = 28.9 Hz), 138.4, 137.1, 137.0, 136.4, 136.3, 134.1, 132.1, 129.6, 129.5, 128.6, 128.6, 128.0, 127.6, 127.4, 127.4, 122.3 (t, *J* = 5.0 Hz), 121.2 (t, *J* = 5.4 Hz), 119.2 (t, *J* = 255.8 Hz), 118.5 (t, *J* = 255.0 Hz), 53.1, 52.6, 46.9 (t, *J* = 6.7 Hz), 46.8 (t, *J* = 6.6 Hz), 44.4, 43.6, 43.6, 38.5 (t, *J* = 24.0 Hz), 36.4, 33.5 (t, *J* = 24.0 Hz), 31.3, 26.6, 25.7, 25.6, 24.5, 24.5, 21.1. ^{19}F NMR (376 MHz, $CDCl_3$) *δ* -98.4 , -98.4 , -98.9, -98.9. IR (v/cm^{-1}): 3308, 2924, 2860, 1739, 1648, 1541, 1512, 1453, 1259, 1185, 1023, 750, 699. HRMS (ESI-MS): calcd for $C_{27}H_{33}F_2N_2O_2$ $[M+H]^+$ 455.2505, found: 455.2502. $[\alpha]_D^{25}$ = 33.19 (c = 0.08, $CHCl_3$). HPLC conditions: IC column, *n*-Hexane/*i*-PrOH = 50/50, 1.0 mL/min, t_{major} = 7.1 min, t_{major} = 7.9 min, t_{minor} = 8.7 min, t_{minor} = 9.2 min, 98:2 er.

(*S*,*E*)-*N*-benzyl-2-(*p*-tolyl)-6-(*p*-tolylthio)hex-4-enamide (2.32)

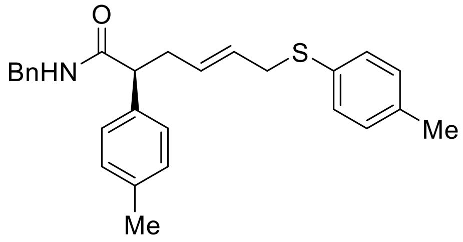

Colorless oil (54% yield, E/Z = 5:1). ^{1}H NMR (400 MHz, $CDCl_3$) δ 7.22–7.19 (m, 2H), 7.18–7.15 (m, 1H), 7.11–7.03 (m, 8H), 6.97 (d, *J* = 8.0 Hz, 2H), 5.51–5.41 (m, 2H), 5.36–5.29 (m, 1H), 4.34 (dd, *J* = 14.8, 6.0 Hz, 1H), 4.23 (dd, *J* = 14.9, 5.7 Hz, 1H), 3.30 (d, *J* = 6.9 Hz, 2H), 3.17 (t, *J* = 7.6 Hz, 1H), 2.83–2.75 (m, 1H), 2.41–2.33 (m, 1H), 2.25 (s, 3H), 2.22 (s, 3H). ^{13}C NMR (101 MHz, $CDCl_3$) δ 173.0, 172.9, 138.4, 137.3, 137.2, 136.4, 136.4, 132.4, 131.6, 131.1, 130.9, 130.1, 129.8, 129.7, 129.6, 128.8, 128.0, 127.8, 127.8, 127.7, 127.5, 53.2, 52.9, 43.7, 37.1, 36.1, 32.3, 31.0, 29.9, 21.2, 21.2. IR (v/cm^{-1}): 3301, 3026, 2925, 1648, 1547, 1513, 1495, 1456, 1245, 1029, 967, 806, 749, 720, 699, 505. HRMS (ESI-MS): calcd for $C_{27}H_{30}NOS$ $[M+H]^+$ 416.2043, found: 416.2043. $[\alpha]_D^{25}$ = 38.57 (*c* = 0.26, $CHCl_3$). HPLC conditions: IC column, *n*-Hexane/*i*-PrOH = 90/10, 1.0 mL/min, t_{major} = 23.1 min, t_{minor} = 26.6 min, t_{major} = 29.9 min, t_{minor} = 37.3 min, 97:3 er.

(*S*,*E*)-*N*-benzyl-6-((4-methoxyphenyl)thio)-2-(*p*-tolyl)hex-4-enamide (2.33)

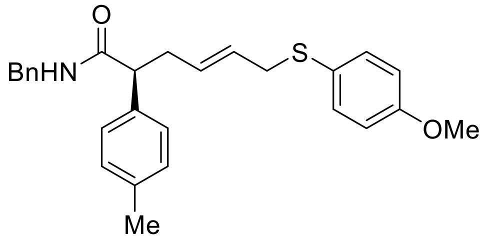

Colorless oil (50% yield, E/Z = 5:1). ^{1}H NMR (400 MHz, $CDCl_3$) δ 7.24–7.20 (m, 2H), 7.19–7.17 (m, 2H), 7.10–7.01 (m, 7H), 6.75 –6.71 (m, 2H), 5.52–5.40 (m, 2H), 5.27–5.20 (m, 1H), 4.35 (dd, *J* = 14.9, 5.9 Hz, 1H), 4.25 (dd, *J* = 14.9, 5.6 Hz, 1H), 3.71 (s, 3H), 3.24 (d, *J* = 7.1 Hz, 2H), 3.15 (t, *J* = 7.5 Hz, 1H), 2.84–2.75 (m, 1H), 2.41–2.32 (m, 1H), 2.26 (s, 3H). ^{13}C NMR (101 MHz, $CDCl_3$) δ 173.0, 172.9, 159.4, 159.1, 138.4, 137.2, 137.2, 136.4, 134.8, 134.2, 131.0, 129.9, 129.7, 128.8, 128.0, 128.0, 127.7, 127.7, 127.5, 127.5, 127.2, 126.1, 114.6, 114.5, 55.5, 53.2, 52.8, 43.7, 43.7, 38.6, 36.1, 30.8, 29.8, 21.2. IR (v/cm^{-1}): 3293, 2917, 1646, 1591, 1511, 1493, 1454, 1283, 1243, 1179, 1029, 967, 824, 734, 698, 637, 509, 423. HRMS (ESI-MS): calcd for $C_{27}H_{30}NO_2S$ $[M+H]^+$ 432.1992, found: 432.1986. $[\alpha]_D^{25}$ = 16.77 (*c* = 0.08, $CHCl_3$). HPLC conditions: IC column, *n*-Hexane/*i*-PrOH = 90/10, 1.0 mL/min, t_{major} = 34.0 min, t_{minor} = 39.6 min, t_{major} = 45.5 min, t_{minor} = 57.3 min, 97:3 er.

5. Scale-up experiment and derivatizations

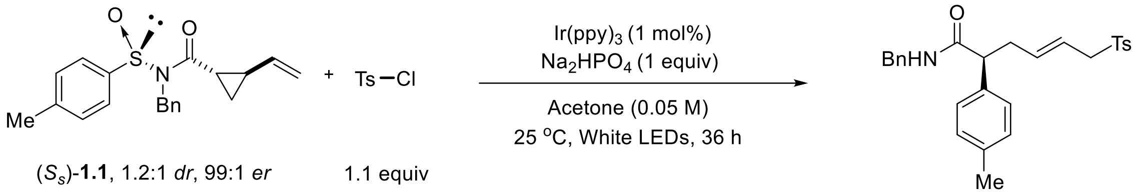

(S_S)-**1.1**, 1.2:1 *dr*, 99:1 *er* 1.1 equiv
0.1 mmol
(*S*)-**2.1**, 77% (6:1), 98:2 *er*

To a 30 mL Schlenk tube equipped with a magnetic stirring bar was added amide **1.1** (339.2 mg, 1.0 mmol, 1.0 equiv), Na_2HPO_4 (142.0, 1.0 mmol, 1.0 equiv), Ir(ppy)$_3$ (6.6 mg, 0.010 mmol, 1 mol%) and sulfonyl chloride (209.7 mg, 1.1 mmol, 1.1 equiv). The Schlenk tube was evacuated and then filled back with nitrogen three times followed by the addition of acetone (20 mL). The mixture was purged with nitrogen for 1 min. Next, the tube was sealed and the reaction was stirred at 1400 rpm in the photoreactor for 36 h under white light irradiation (7.2 W/m, white LED). The reaction mixture was filtered through a short silica plug with EtOAc as eluent. The solvent was removed and the residue was purified by flash chromatography using a mixture EtOAc:Hexane (3:1) to afford (*S*)-**2.1** (344.3 mg, 77% yield).

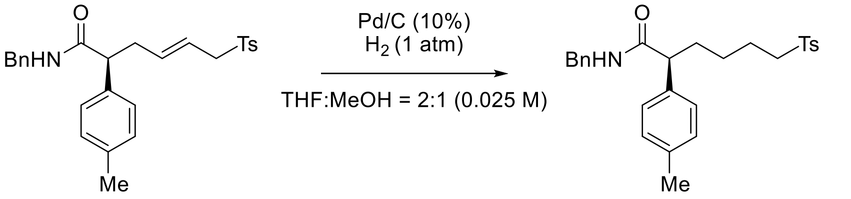

(*S*)-**2.1** (*S*)-**3.1**

To an oven-dried 10 mL microwave vial equipped with a magnetic stirring bar was added amide **2.1** (22.4 mg, 0.050 mmol, 1.0 equiv) and Pd/C (5.3 mg, 0.050 mmol, 0.10 equiv). The vial was sealed, evacuated and then filled back with nitrogen three times followed by the addition of 2 mL THF:MeOH (2:1) mixture. Then the vial was evacuated and backfilled with hydrogen gas (1 atm) 5 times. The resulting mixture was stirred at 25 °C for 18 h followed by filtration through a pad of celite. The filtrate was concentrated to afford the corresponding product (*S*)-**3.1** (22.0 mg, 98% yield) as a colorless oil without further purification.

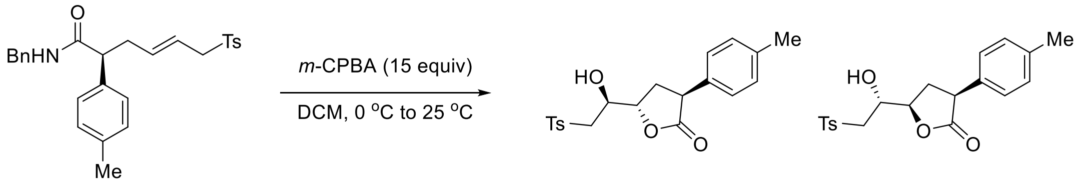

(*S*)-**2.1** (*S,S,S*)-**3.2** (*S,R,R*)-**3.2**

To a 4 mL vial equipped with a magnetic stirring bar was added amide **2.1** (31.8 mg, 0.070 mmol, 1.0 equiv) and 0.5 mL DCM. The solution was cooled to 0 °C followed by the addition of *m*CPBA (239 mg, 1.1 mmol, 15.0 equiv) in 0.5 mL DCM. The mixture was stirred vigorously for 21 h at 25 °C. The reaction was quenched with Na_2SO_3 (5 mL) solution and extracted with DCM (3 x 5 mL). The combined organic phases were washed with Na_2SO_3 (5

mL) solution twice. The resulting organic phase was concentrated, and the crude was purified by flash column chromatography over silica gel to afford the corresponding lactone (*S*,*S*,*S*)-**3.2** (11.4 mg, 43% yield, 88:12 *er*) and (*S*,*R*,*R*)-**3.2** (10.1 mg, 38% yield, 86:14 *er*) as white solids. After recrystallizing in EtOAc:Hexane, the er values of (*S*,*S*,*S*)-**3.2** (10.1 mg, 38% yield, 93:7 *er*) and (*S*,*R*,*R*)-**3.2** (7.2 mg, 27% yield, 98:2 *er*) were improved.

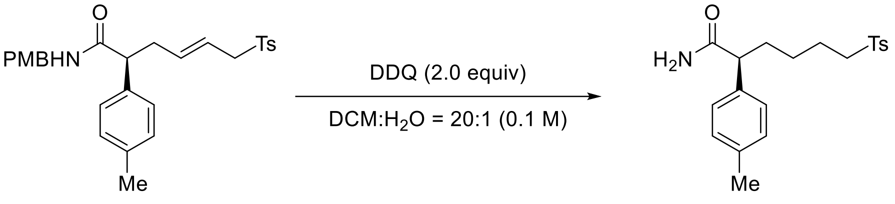

(*S*)-**2.10** (*S*)-**3.3**

To a 4 mL vial equipped with a magnetic stirring bar was added amide **2.10** (53 mg, 0.11 mmol, 1.0 equiv) and 1.2 mL DCM:H_2O (20:1) followed by the addition of 2,3-dichloro-5,6-dicyano-*p*-benzoquinone (50.4 mg, 0.22 mmol, 2.0 equiv). The mixture was stirred vigorously for 48 h at 25 °C. The reaction was quenched with Na_2SO_3 (5 mL) solution and extracted with DCM (3 x 5 mL). The organic phases were concentrated, and the crude was purified by flash column chromatography over silica gel to afford the corresponding primary amide **3.3** (39.9 mg, 88% yield) as a colorless oil.[5]

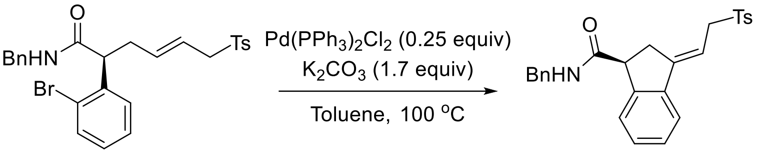

(*S*)-**2.6** (*S*)-**3.4**

To a Schlenk tube was added **2.6** (25.6 mg, 0.05 mmol, 1.0 equiv), $Pd(PPh_3)_2Cl_2$ (8.8 mg, 0.010 mmol, 0.25 equiv) and potassium carbonate (11.8 mg, 0.085 mmol, 1.7 equiv). In a glovebox filled with nitrogen, 0.5 mL toluene was added and Schlenk tube was sealed. The reaction mixture was heated at 100 °C for 18 h and then cooled down to room temperature. After filtration on a short plug of silica, the filtrate was concentrated, and the crude was purified by flash column chromatography over silica gel to afford **3.4** (21.6 mg, 67% yield) as a yellow oil.[6]

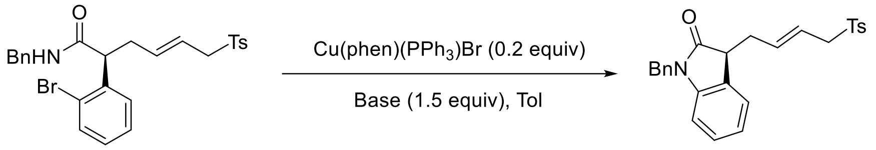

Entry	Base	Temperature (°C)	Time (h)	Starting material (%)[b]	Yield of product (%)[b]	*Er* of product
1	K_3PO_4	115	48	0	80	50:50
2[a]	K_3PO_4	100	48	0	92	50:50
3[a]	K_3PO_4	100	21	32	56	50:50
4[a]	K_2CO_3	100	24	22	69	50:50

[a] Cu(phen)(PPh_3)Br was recrystallized. [b] Yield was determined by ^{1}H-HMR using mesitylene as the internal standard.

(*S*)-*N*-Benzyl-2-(*p*-tolyl)-6-tosylhexanamide (3.1)

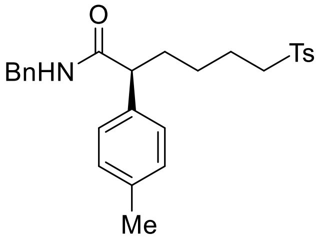

Colorless oil (98% yield). ^{1}H NMR (400 MHz, $CDCl_3$) δ 7.76 (d, *J* = 8.3 Hz, 2H), 7.36 (d, *J* = 8.0 Hz, 2H), 7.32–7.25 (m, 3H), 7.22–7.09 (m, 6H), 5.69 (t, *J* = 5.8 Hz, 1H), 4.42 (dd, *J* = 14.9, 5.9 Hz, 1H), 4.33 (dd, *J* = 14.9, 5.7 Hz, 1H), 3.28 (dd, *J* = 8.3, 6.9 Hz, 1H), 3.06–2.99 (m, 2H), 2.47 (s, 3H), 2.35 (s, 3H), 2.21–2.11 (m, 1H), 1.76–1.67 (m, 3H), 1.38–1.27 (m, 2H). ^{13}C NMR (101 MHz, $CDCl_3$) δ 173.3, 144.7, 138.4, 137.3, 136.5, 136.2, 130.0, 129.8, 128.8, 128.2, 127.9, 127.6, 127.5, 56.2, 52.7, 43.7, 32.6, 26.5, 22.8, 21.8, 21.2. IR (v/cm^{-1}): 3312, 3027, 2928, 2864, 1742, 1650, 1511, 1453, 1286, 1225, 1145, 1087, 815, 725, 698, 665, 563, 518. HRMS (ESI-MS): calcd for $C_{27}H_{32}NO_3S$ $[M+H]^+$ 450.2097, found: 450.2094. $[\alpha]_D^{25}$ = 5.41 (*c* = 0.15, $CHCl_3$). HPLC conditions: AD-H column, *n*-Hexane/*i*-PrOH = 70/30, 1.0 mL/min, t_{major} = 12.3 min, t_{minor} = 38.7 min, 99:1 er.

(3*S*,5*S*)-5-((*S*)-1-Hydroxy-2-tosylethyl)-3-(*p*-tolyl)dihydrofuran-2(3H)-one (*S*,*S*,*S*-3.2)

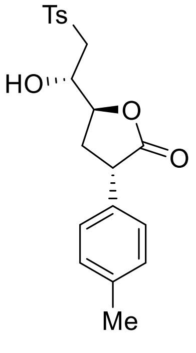

White solid (38% yield). M.p.: 166.6-167.3 °C. ^{1}H NMR (400 MHz, $CDCl_3$) δ 7.82 (d, *J* = 7.4 Hz, 2H), 7.40 (d, *J* = 7.9 Hz, 2H), 7.19–7.09 (m, 4H), 4.47–4.41 (m, 1H), 4.32–4.25 (m, 1H), 3.91 (t, *J* = 9.0 Hz, 1H), 3.42 (d, *J* = 14.1 Hz, 1H), 3.25 (dd, *J* = 14.0, 9.6 Hz, 1H), 2.79–2.68 (m, 1H), 2.51–2.41 (m, 4H), 2.33 (s, 3H). ^{13}C NMR (101 MHz, $CDCl_3$) δ 177.0, 145.8, 137.7, 135.8, 133.8, 130.4, 129.9, 128.1, 127.6, 78.6, 67.7, 59.1, 44.7, 32.2, 21.9, 21.2. IR (v/cm^{-1}): 3471, 2925, 2848, 1772, 1301, 1289, 1141, 1019, 811, 541. HRMS (ESI-MS): calcd for $C_{20}H_{26}NO_5S$ $[M+NH_4]^+$ 392.1526, found: 392.1528. $[\alpha]_D^{25}$ = 43.48 (*c* = 0.07, $CHCl_3$). HPLC conditions: AD column, *n*-Hexane/*i*-PrOH = 70/30, 1.0 mL/min, t_{minor} = 18.6 min, t_{major} = 26.7 min, 93:7 er.

(3*S*,5*R*)-5-((*R*)-1-Hydroxy-2-tosylethyl)-3-(*p*-tolyl)dihydrofuran-2(3H)-one (*S*,*R*,*R*-3.2)

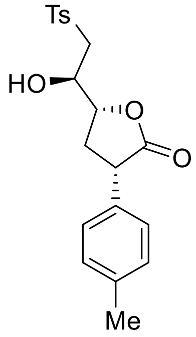

White solid (27% yield). M.p.: 179.3-180.1 °C. ^{1}H NMR (400 MHz, $CDCl_3$) δ 7.81 (d, *J* = 7.6 Hz, 2H), 7.39(d, *J* = 7.7 Hz, 2H), 7.20–7.08 (m, 4H), 4.43–4.33 (m, 1H), 4.29–4.21 (m, 1H), 3.84 (t, *J* = 9.0 Hz, 1H), 3.68 (s, 1H), 3.47 (d, *J* = 14.3 Hz, 1H), 3.28 (dd, *J* = 14.5, 9.2 Hz, 1H), 2.85–2.75 (m, 1H), 2.48 (s, 3H), 2.37–2.27 (m, 4H). ^{13}C NMR (101 MHz, $CDCl_3$) δ 176.2, 145.8, 137.8, 135.8, 132.9, 130.4, 129.7, 128.1, 128.0, 78.2, 68.2, 59.1, 46.0, 33.6, 21.9, 21.2. IR (v/cm^{-1}): 3475, 2925, 1722, 1300, 1286, 1142, 1086, 1034, 808, 774, 549. HRMS (ESI-MS): calcd for $C_{20}H_{26}NO_5S$ $[M+NH_4]^+$ 392.1526, found: 392.1527. $[\alpha]_D^{25}$ = -62.82 (*c* = 0.07, $CHCl_3$). HPLC conditions: AD column, *n*-Hexane/*i*-PrOH = 70/30, 1.0 mL/min, t_{major} = 22.0 min, t_{minor} = 25.5 min, 98:2 er.

(*S*,*E*)-2-(*p*-Tolyl)-6-tosylhexanamide (3.3)

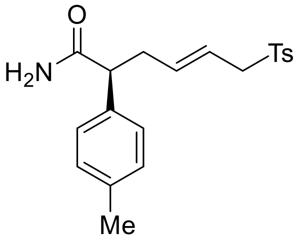

Colorless oil (88% yield, E/Z = 6:1). ^{1}H NMR (500 MHz, $CDCl_3$) δ 7.64 (d, *J* = 8.5 Hz, 2H), 7.30 (d, *J* = 7.9 Hz, 2H), 7.15–7.09 (m, 4H), 5.55–5.47 (m, 1H), 5.46–5.41 (m, 1H), 5.36 (brs, 2H), 3.66 (d, *J* = 7.1 Hz, 2H), 3.34 (t, *J* = 7.5 Hz, 1H), 2.88–2.81 (m, 1H), 2.49–2.42 (m, 4H), 2.33 (s, 3H). ^{13}C NMR (126 MHz, $CDCl_3$) δ 175.4, 175.3, 144.9, 144.7, 138.5, 137.4, 137.4, 136.5, 136.0, 135.9, 135.6, 129.9, 129.8, 129.7, 129.7, 128.5, 127.9, 127.9, 118.4, 117.2, 60.1, 55.3, 51.7, 51.5, 36.0, 31.0, 21.7, 21.2. IR (v/cm^{-1}): 3441, 3354, 3201, 3011, 2970, 2925, 1739, 1670, 1377, 1276, 1261, 1135, 765, 750, 560, 508. HRMS (ESI-MS): calcd for $C_{20}H_{24}NO_3S$ $[M+H]^+$ 358.1471, found: 358.1466. $[\alpha]_D^{25}$ = -71.66 (*c* = 0.19, $CHCl_3$). HPLC conditions: IC column, *n*-Hexane/*i*-PrOH = 50/50, 1.0 mL/min, t_{major} = 39.0 min, t_{major} = 43.1min, t_{minor} = 59.4 min, t_{minor} = 76.8 min, 99:1 er.

(S,*E*)-*N*-Benzyl-3-(2-tosylethylidene)-2,3-dihydro-1H-indene-1-carboxamide (3.4)

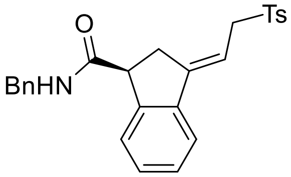

Yellow oil (67% yield, E/Z = 9:1). ^{1}H NMR (400 MHz, $CDCl_3$) δ 7.73 (d, *J* = 8.2 Hz, 2H), 7.54–7.46 (m, 1H), 7.40–7.36 (m, 1H), 7.34–7.26 (m, 7H), 7.22–7.16 (m, 2H), 6.14 (t, *J* = 5.7 Hz, 1H), 5.93–5.85 (m , 1H), 4.47 (dd, *J* = 14.9, 6.1 Hz, 1H), 4.38 (dd, *J* = 14.8, 5.5 Hz, 1H), 4.06–3.90 (m, 3H), 2.98–2.90 (m, 1H), 2.86–2.77 (m, 1H), 2.45 (s, 3H). ^{13}C NMR (101 MHz, $CDCl_3$) δ 172.8, 149.6, 145.0, 144.3, 140.4, 138.3, 136.1, 130.0, 129.9, 129.9, 128.8, 128.5, 128.4, 127.7, 127.5, 125.5, 121.3, 108.3, 105.4, 57.7, 49.9, 43.7, 39.9, 33.2, 21.8. IR (v/cm^{-1}): 3304, 3065, 3027, 2925, 2848, 1739, 1650, 1535, 1369, 1301, 1230, 1138, 1086, 732, 700, 517. HRMS (ESI-MS): calcd for $C_{20}H_{26}NO_3S$ $[M+H]^+$ 432.1628, found: 432.1629. $[\alpha]_D^{25}$ = -51.75 (*c* = 0.09, $CHCl_3$). HPLC conditions: AD column, *n*-Hexane/*i*-PrOH = 60/40, 1.0 mL/min, t_{minor} = 9.4 min, t_{minor} = 11.1 min, t_{major} = 12.6 min, t_{major} = 18.3 min, 96:4 er.

6. X-Ray Crystallographic data for 2.1 (CCDC 2500555)

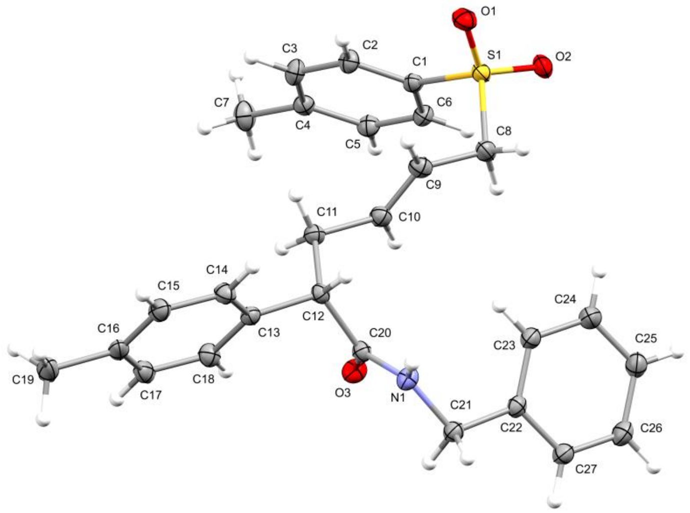

Empirical formula	$C_{27}H_{29}NO_3S$
Formula weight	447.57
Temperature/K	160.0(1)
Crystal system	orthorhombic
Space group	$P2_12_12_1$
a/Å	5.87880(10)
b/Å	17.3407(2)
c/Å	23.2010(3)
α/°	90
β/°	90
γ/°	90
Volume/Å^3	2365.17(6)
Z	4
ρ_{calc}g/cm^3	1.257
μ/mm^{-1}	1.438
F(000)	952.0
Crystal size/mm^3	0.23 × 0.08 × 0.05
Radiation	Cu Kα (λ = 1.54184)
2Θ range for data collection/°	6.364 to 153.228
Index ranges	$-7 \le h \le 7$, $-13 \le k \le 21$, $-29 \le l \le 29$
Reflections collected	32954
Independent reflections	4979 [R_{int} = 0.0382, R_{sigma} = 0.0252]
Data/restraints/parameters	4979/0/295
Goodness-of-fit on F^2	1.053
Final R indexes [I>=2σ (I)]	R_1 = 0.0282, wR_2 = 0.0695
Final R indexes [all data]	R_1 = 0.0301, wR_2 = 0.0711
Largest diff. peak/hole / e Å^{-3}	0.17/-0.21
Flack parameter	-0.004(7)

7. NMR spectra and HPLC traces

N-Benzyl-*N*-((*S*)-*p*-tolylsulfinyl)-2-vinylcyclopropane-1-carboxamide (1.1)

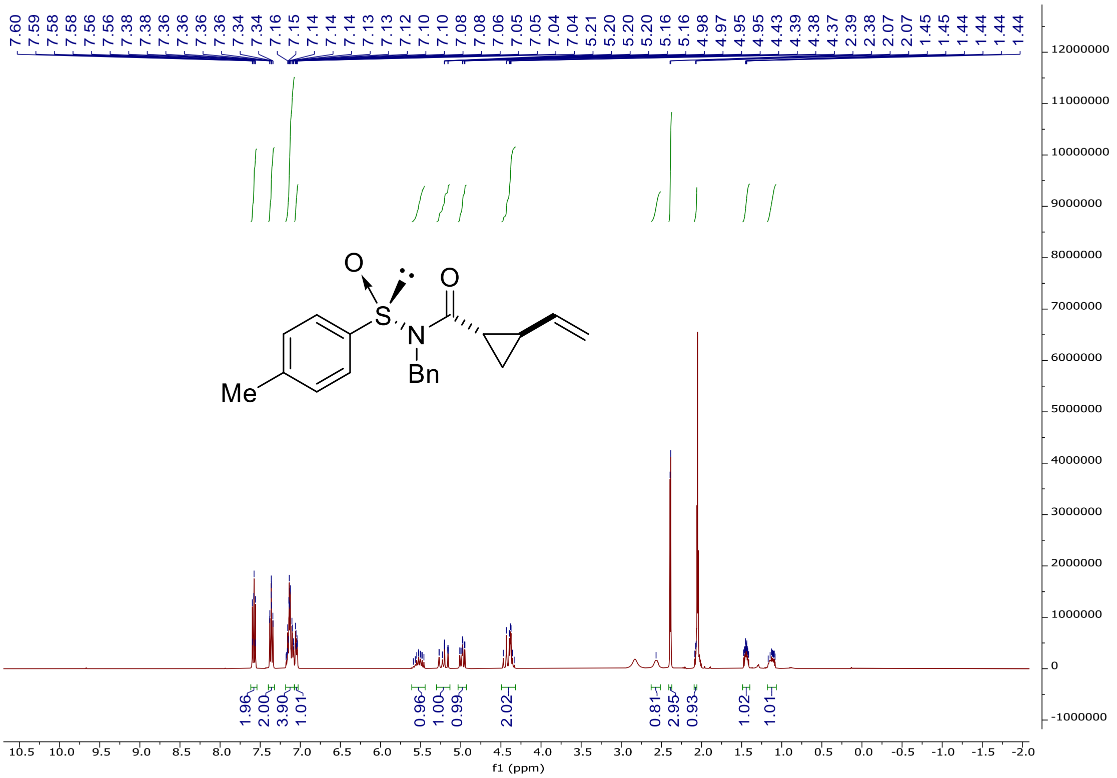

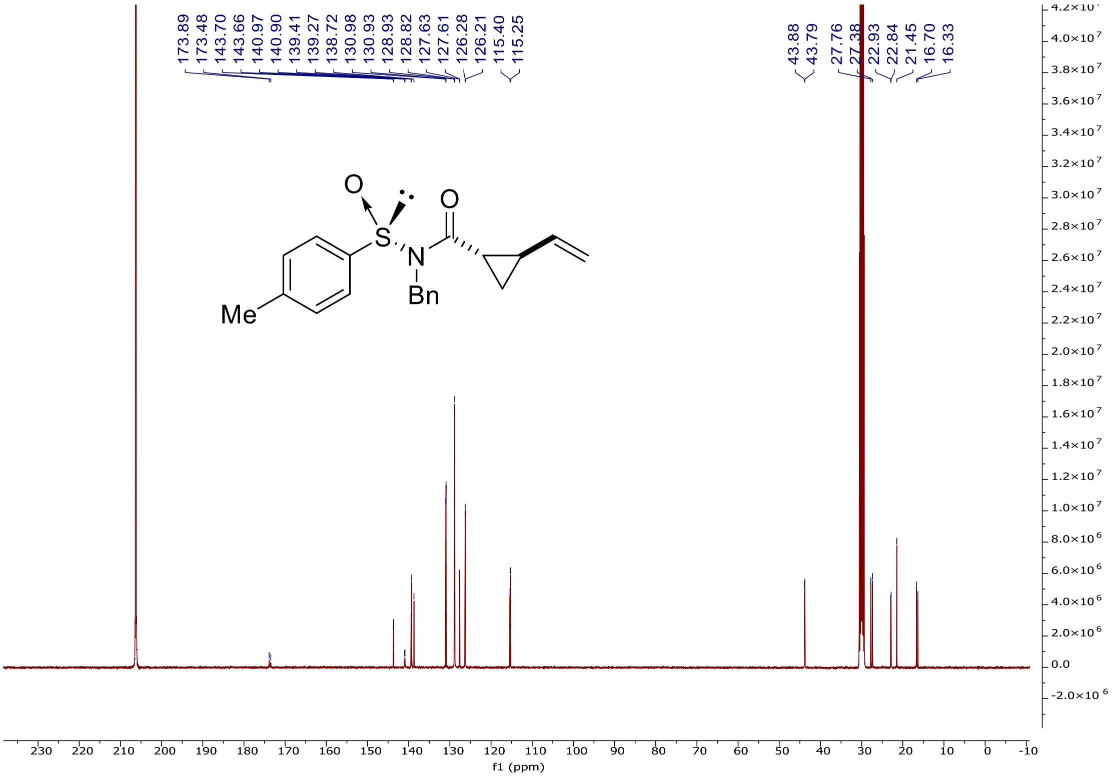

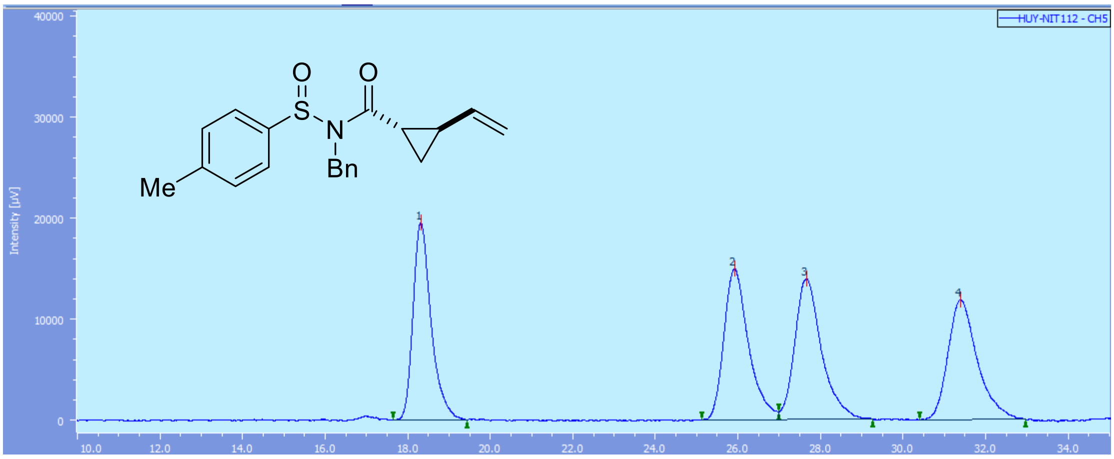

#	Peak Name	CH	tR	Area	Height	Area%	Height%	Quantity	NTP	Resolution	Symmetry Factor	Warning
1	Unknown	5	18.307	593270	19496	24.528	32.415	N/A	9241	8.435	1.387	
2	Unknown	5	25.907	616738	14915	25.498	24.799	N/A	9831	1.620	1.454	
3	Unknown	5	27.650	625625	13889	25.866	23.092	N/A	9875	3.171	N/A	
4	Unknown	5	31.373	583095	11845	24.108	19.694	N/A	10215	N/A	1.371	

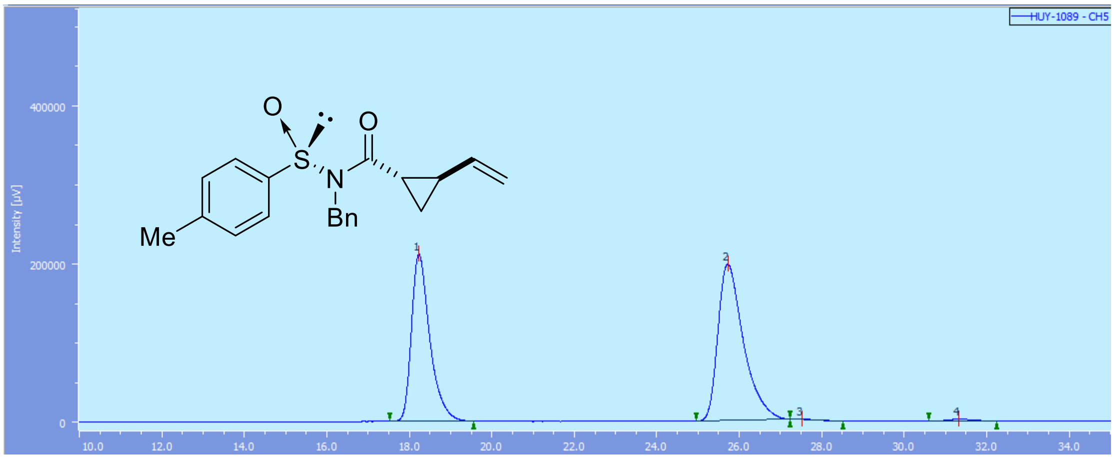

#	Peak Name	CH	tR	Area	Height	Area%	Height%	Quantity	NTP	Resolution	Symmetry Factor	Warning
1	Unknown	5	18.227	6496237	210687	42.921	51.157	N/A	8877	8.055	1.464	
2	Unknown	5	25.707	8509253	197855	56.221	48.041	N/A	8925	1.973	1.561	
3	Unknown	5	27.503	18960	759	0.125	0.184	N/A	22251	4.024	1.307	
4	Unknown	5	31.307	111008	2546	0.733	0.618	N/A	11700	N/A	1.157	

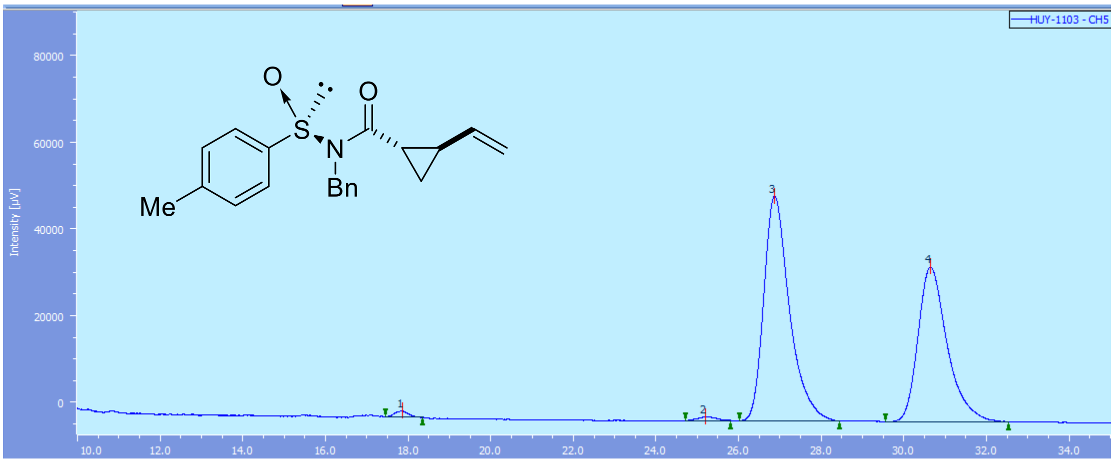

#	Peak Name	CH	tR	Area	Height	Area%	Height%	Quantity	NTP	Resolution	Symmetry Factor	Warning
1	Unknown	5	17.850	31550	1493	0.781	1.666	N/A	14065	10.252	0.997	
2	Unknown	5	25.187	25987	843	0.643	0.940	N/A	14626	1.749	1.240	
3	Unknown	5	26.860	2232106	51686	55.259	57.660	N/A	9792	3.248	1.388	
4	Unknown	5	30.623	1749701	35616	43.316	39.733	N/A	9791	N/A	1.382	

N-Benzyl-*N*-((*S*)-(4-methoxyphenyl)sulfinyl)-2-vinylcyclopropane-1-carboxamide (1.2)

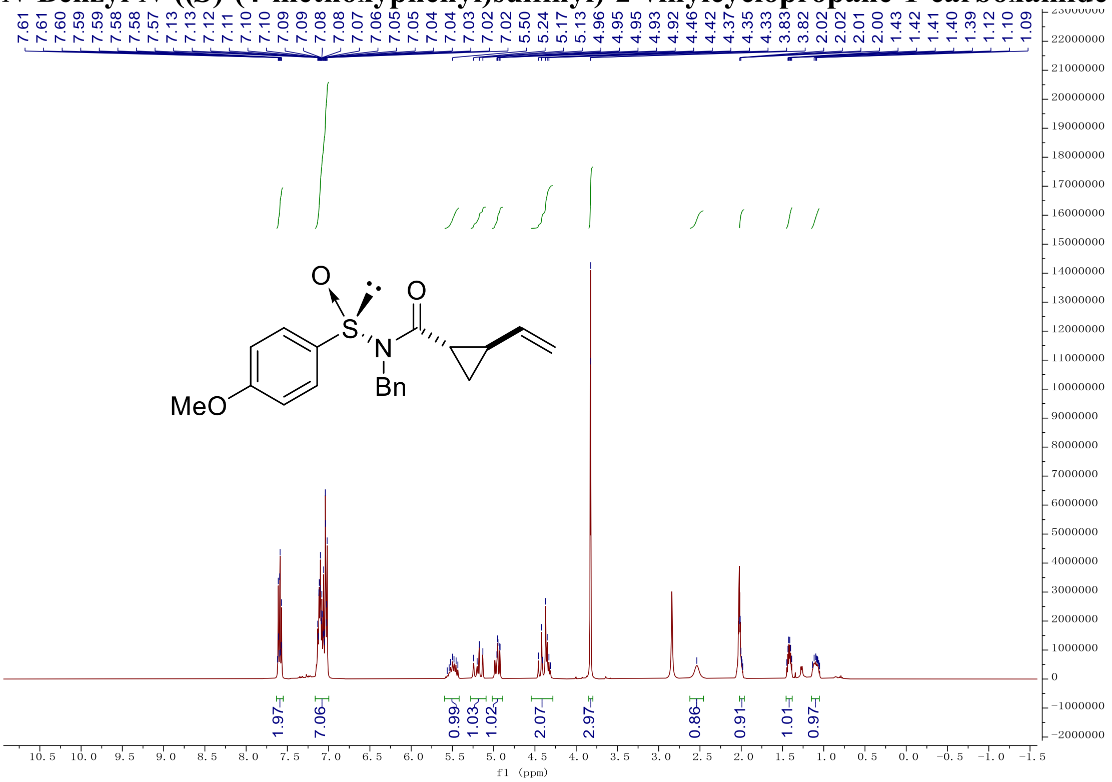

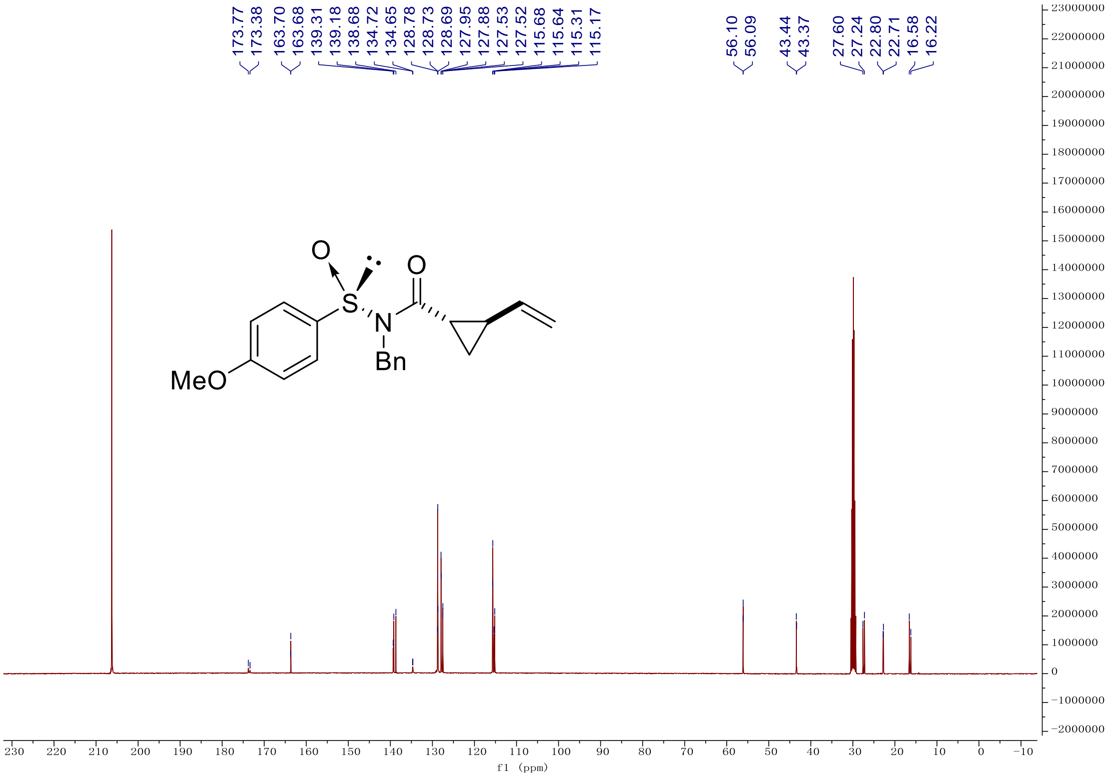

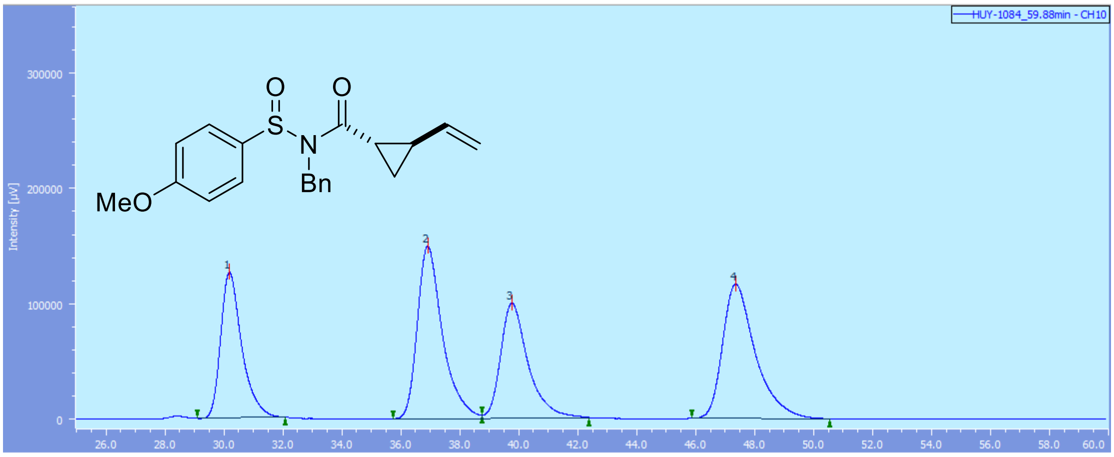

#	Peak Name	CH	tR	Area	Height	Area%	Height%	Quantity	NTP	Resolution	Symmetry Factor	Warning
1	Unknown	10	30.183	6256369	125999	20.537	25.691	N/A	9452	4.964	1.432	
2	Unknown	10	36.900	8821921	149034	28.958	30.387	N/A	10061	1.870	1.458	
3	Unknown	10	39.753	6412334	99316	21.048	20.250	N/A	10021	4.340	1.442	
4	Unknown	10	47.340	8973954	116096	29.457	23.672	N/A	9753	N/A	1.503	

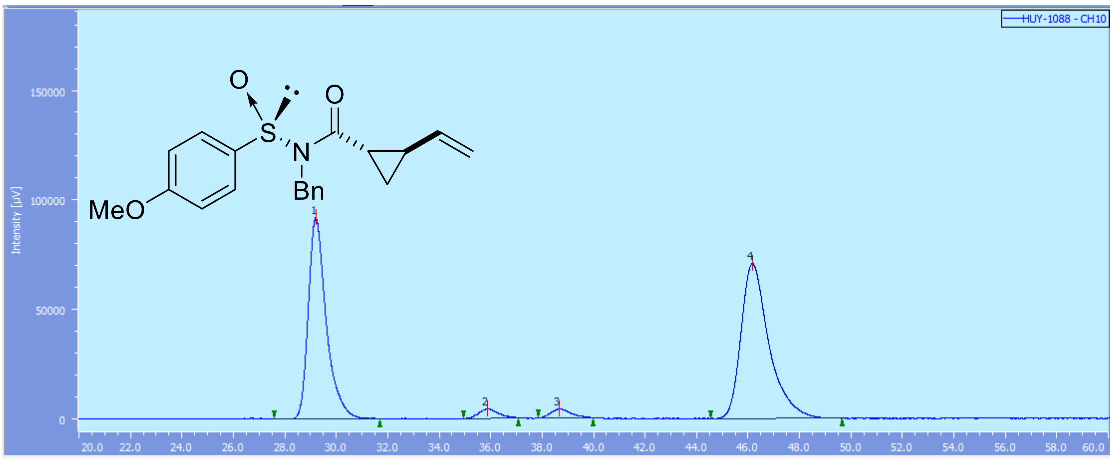

#	Peak Name	CH	tR	Area	Height	Area%	Height%	Quantity	NTP	Resolution	Symmetry Factor	Warning
1	Unknown	10	29.190	4427531	91600	43.453	53.737	N/A	9568	5.307	1.457	
2	Unknown	10	35.850	215091	4145	2.111	2.432	N/A	11751	2.050	1.277	
3	Unknown	10	38.657	230924	4182	2.266	2.454	N/A	11813	4.577	1.298	
4	Unknown	10	46.150	5315790	70533	52.170	41.378	N/A	9848	N/A	1.476	

N-Nenzyl-*N*-((*S*)-(4-bromophenyl)sulfinyl)-2-vinylcyclopropane-1-carboxamide (1.3)

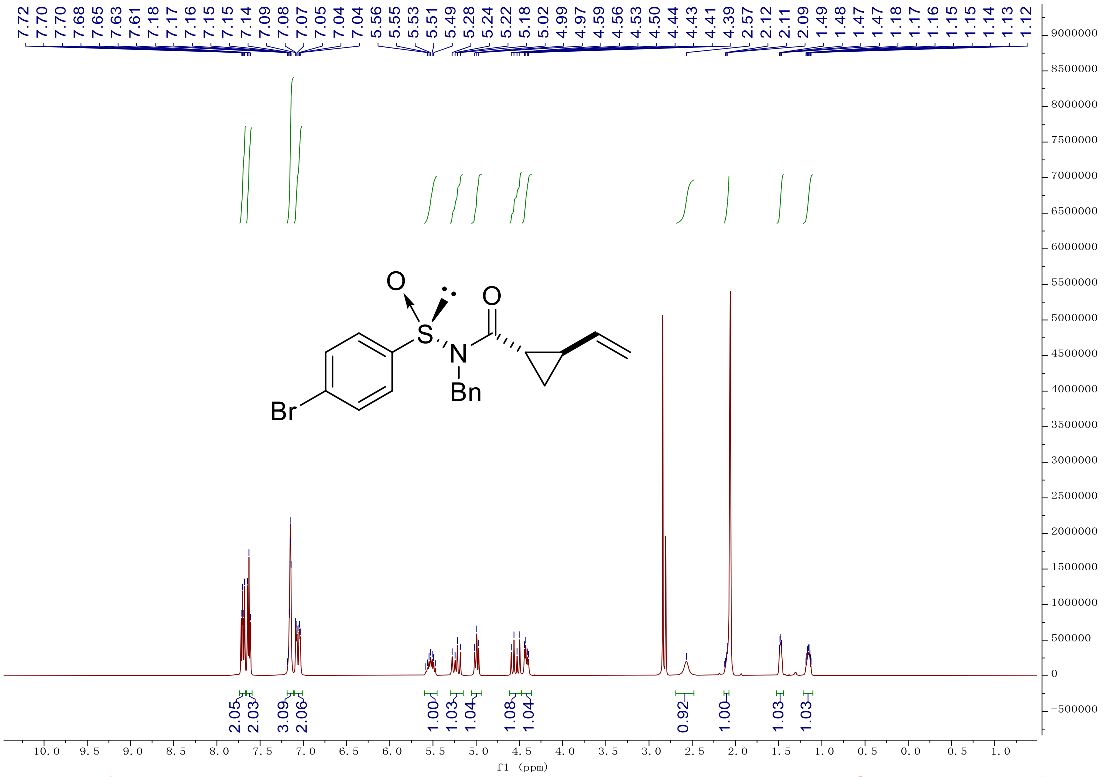

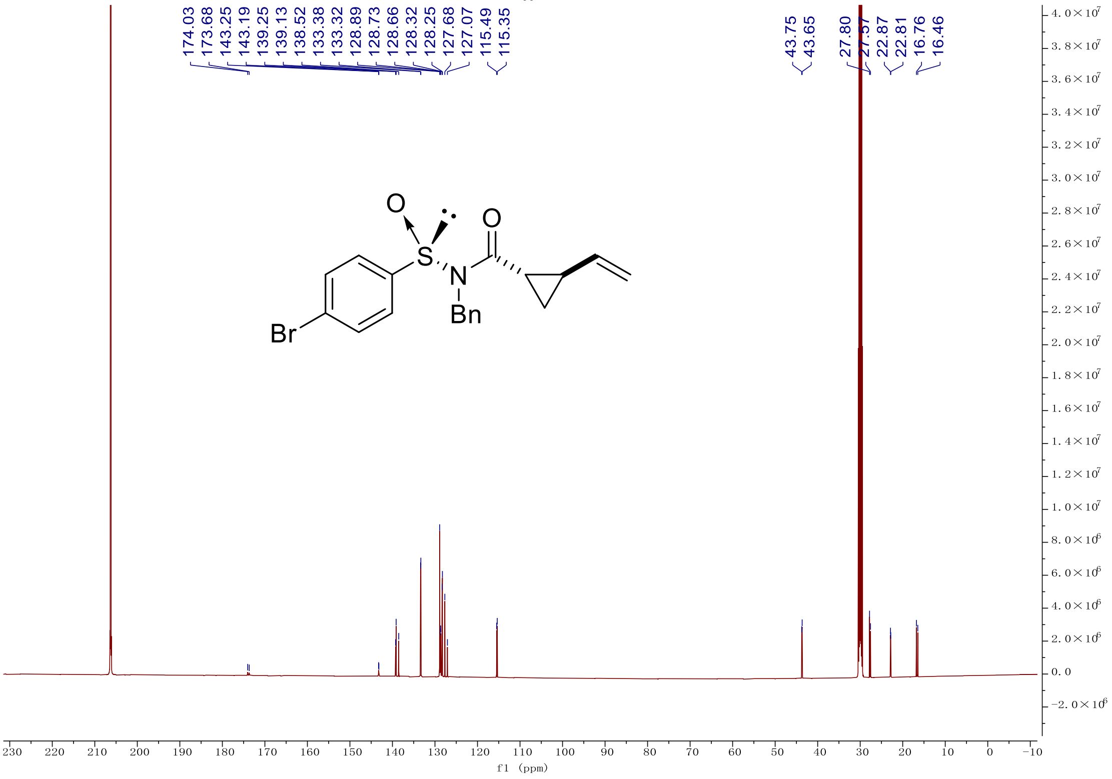

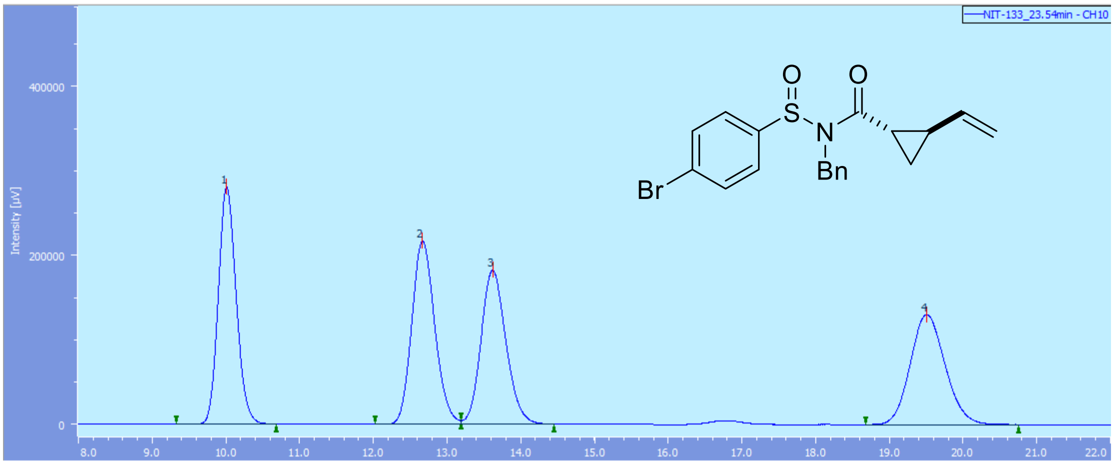

#	Peak Name	CH	tR	Area	Height	Area%	Height%	Quantity	NTP	Resolution	Symmetry Factor	Warning
1	Unknown	10	10.010	4687518	279341	26.189	34.624	N/A	8290	5.268	1.142	
2	Unknown	10	12.663	4686976	216022	26.186	26.776	N/A	7890	1.608	1.151	
3	Unknown	10	13.613	4265709	181553	23.832	22.503	N/A	7859	7.993	1.147	
4	Unknown	10	19.497	4258836	129863	23.794	16.097	N/A	8190	N/A	1.120	

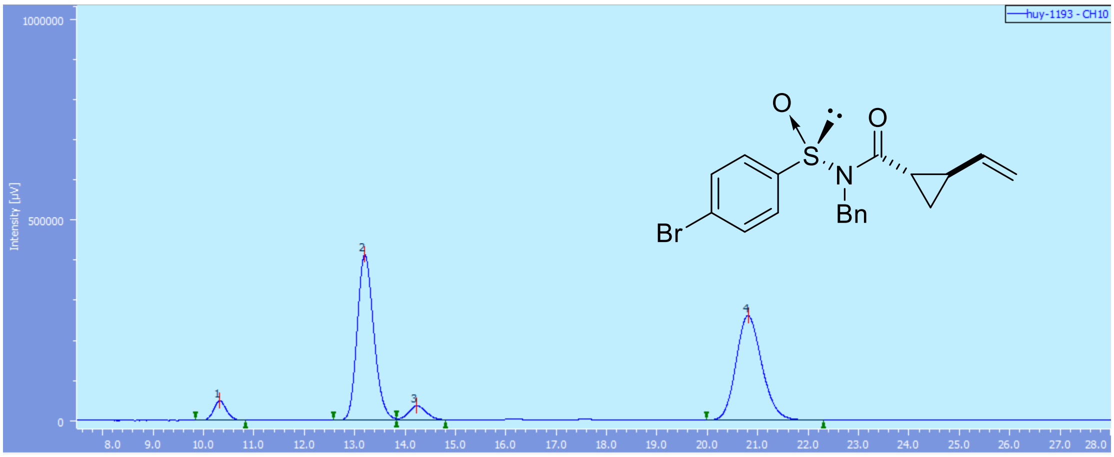

#	Peak Name	CH	tR	Area	Height	Area%	Height%	Quantity	NTP	Resolution	Symmetry Factor	Warning
1	Unknown	10	10.317	857976	48862	4.218	6.478	N/A	8104	5.465	1.122	
2	Unknown	10	13.197	9400224	411199	46.210	54.517	N/A	7784	1.658	1.184	
3	Unknown	10	14.227	863412	34889	4.244	4.626	N/A	7723	8.346	N/A	
4	Unknown	10	20.793	9220812	259310	45.328	34.379	N/A	7994	N/A	1.164	

N-Benzyl-*N*-((*S*)-(4-fluorophenyl)sulfinyl)-2-vinylcyclopropane-1-carboxamide (1.4)

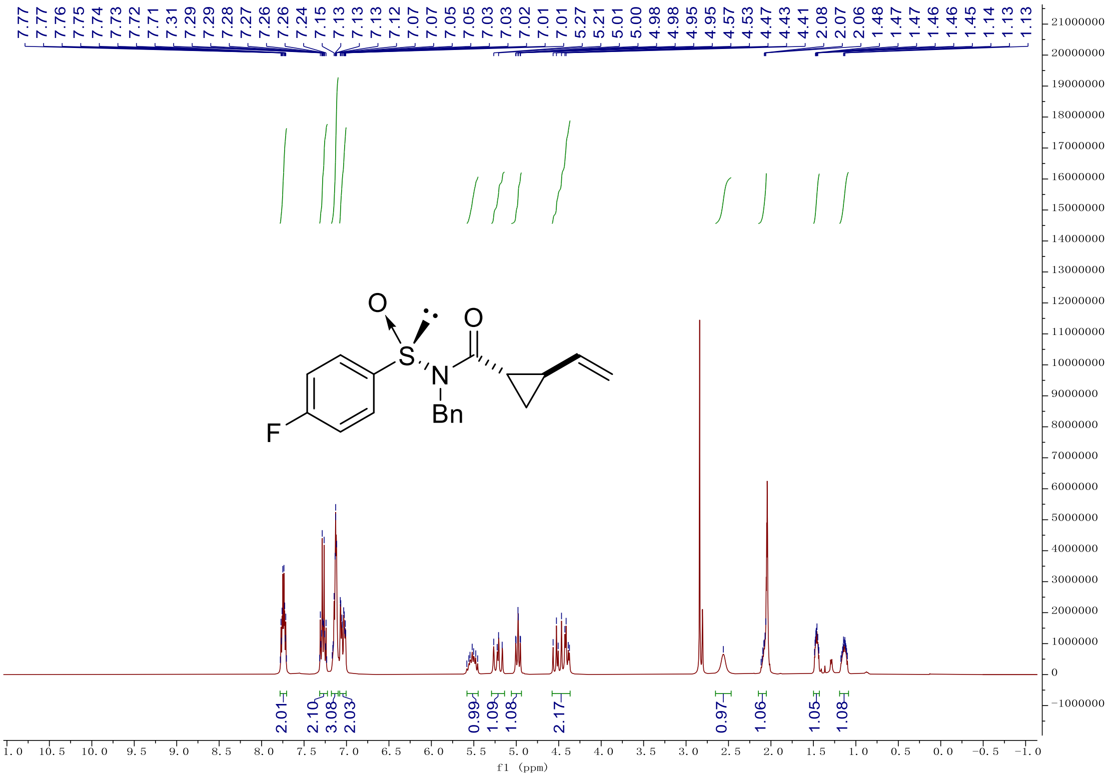

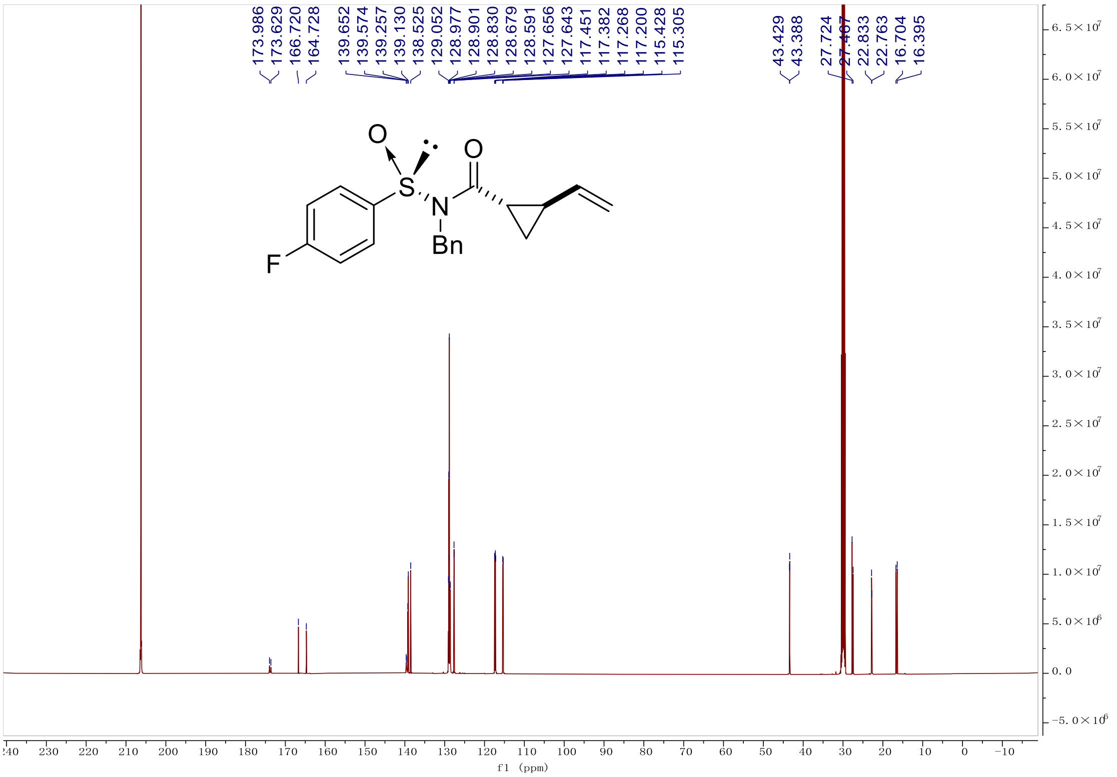

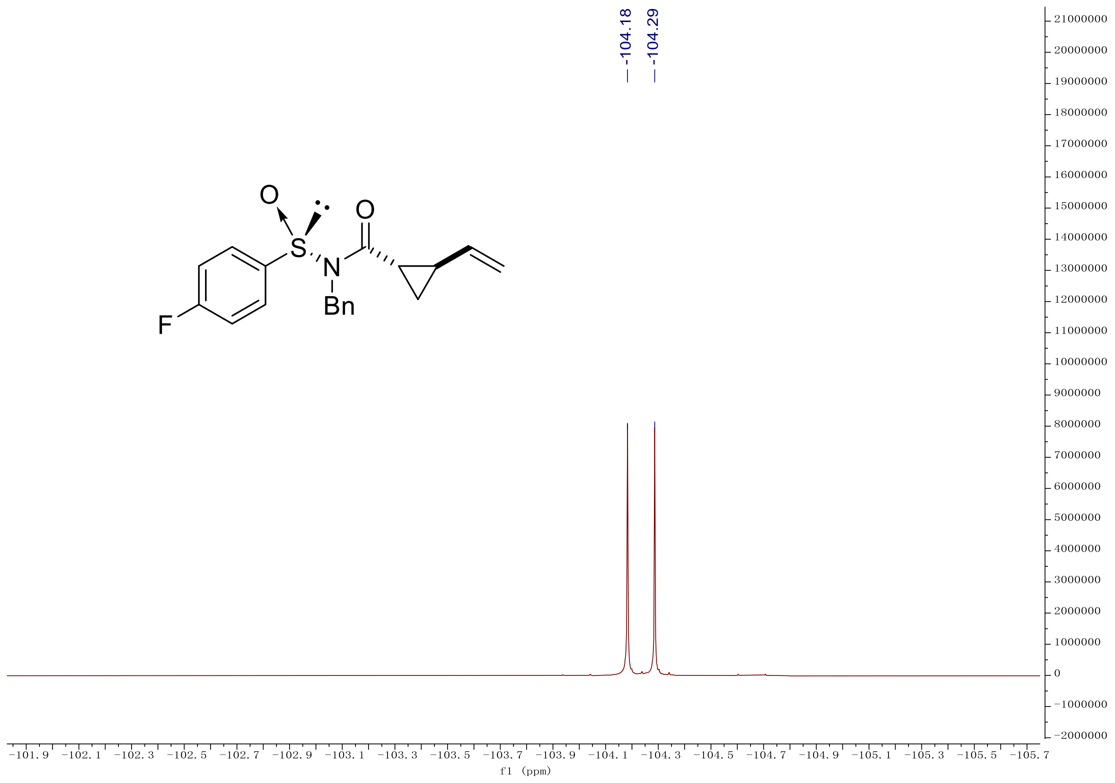

O
S
N
O
Bn
F
-104.18
-104.29
f1 (ppm)

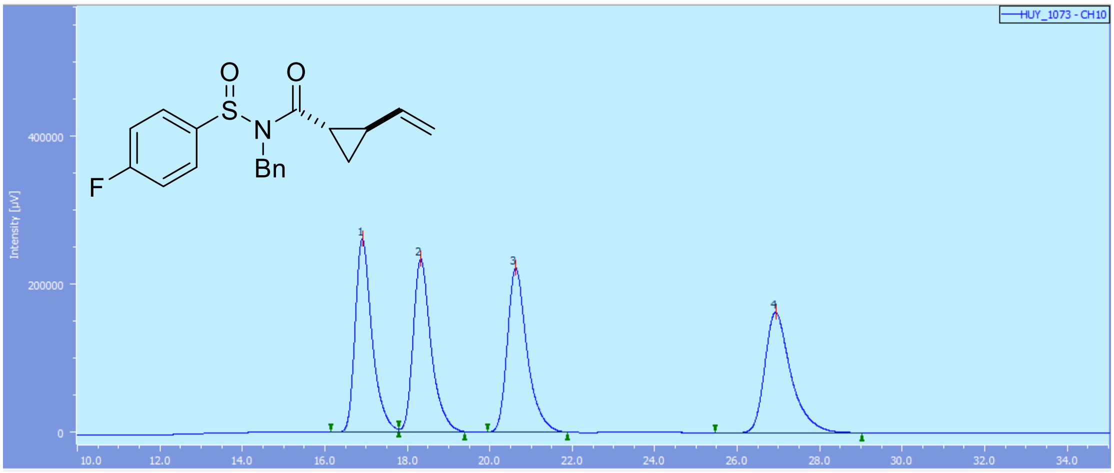

#	Peak Name	CH	tR	Area	Height	Area%	Height%	Quantity	NTP	Resolution	Symmetry Factor	Warning
1	Unknown	10	16.903	7355511	259996	25.464	29.696	N/A	9035	1.928	1.406	
2	Unknown	10	18.317	7040010	232572	24.371	26.564	N/A	9331	2.897	1.390	
3	Unknown	10	20.617	7352283	220775	25.453	25.217	N/A	9787	6.551	1.387	
4	Unknown	10	26.913	7138477	162173	24.712	18.523	N/A	9685	N/A	1.449	

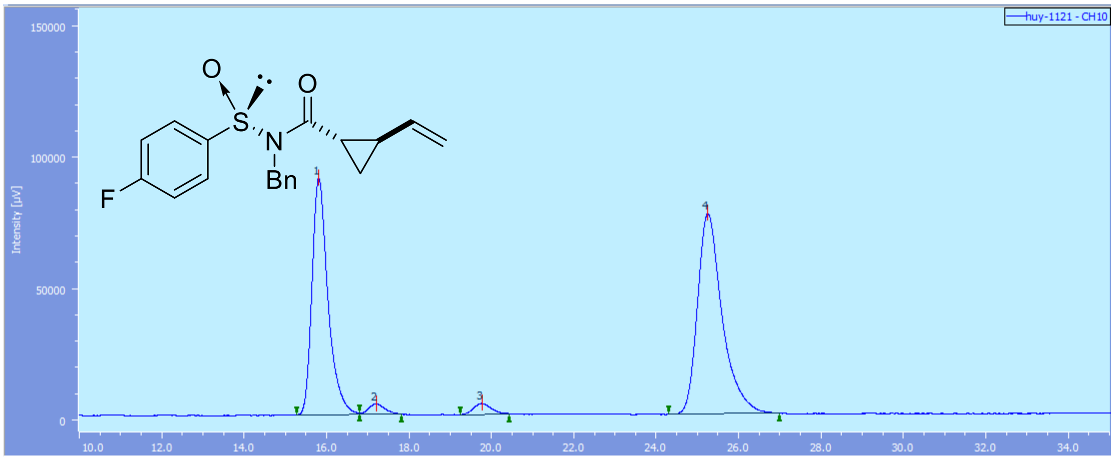

#	Peak Name	CH	tR	Area	Height	Area%	Height%	Quantity	NTP	Resolution	Symmetry Factor	Warning
1	Unknown	10	15.807	2422329	89879	41.672	51.648	N/A	8877	2.019	1.409	
2	Unknown	10	17.193	109140	3979	1.878	2.287	N/A	9486	3.494	N/A	
3	Unknown	10	19.760	117851	4072	2.027	2.340	N/A	10622	6.119	1.142	
4	Unknown	10	25.240	3163552	76094	54.423	43.726	N/A	9624	N/A	1.425	

N-Benzyl-*N*-((*S*)-*o*-tolylsulfinyl)-2-vinylcyclopropane-1-carboxamide (1.5)

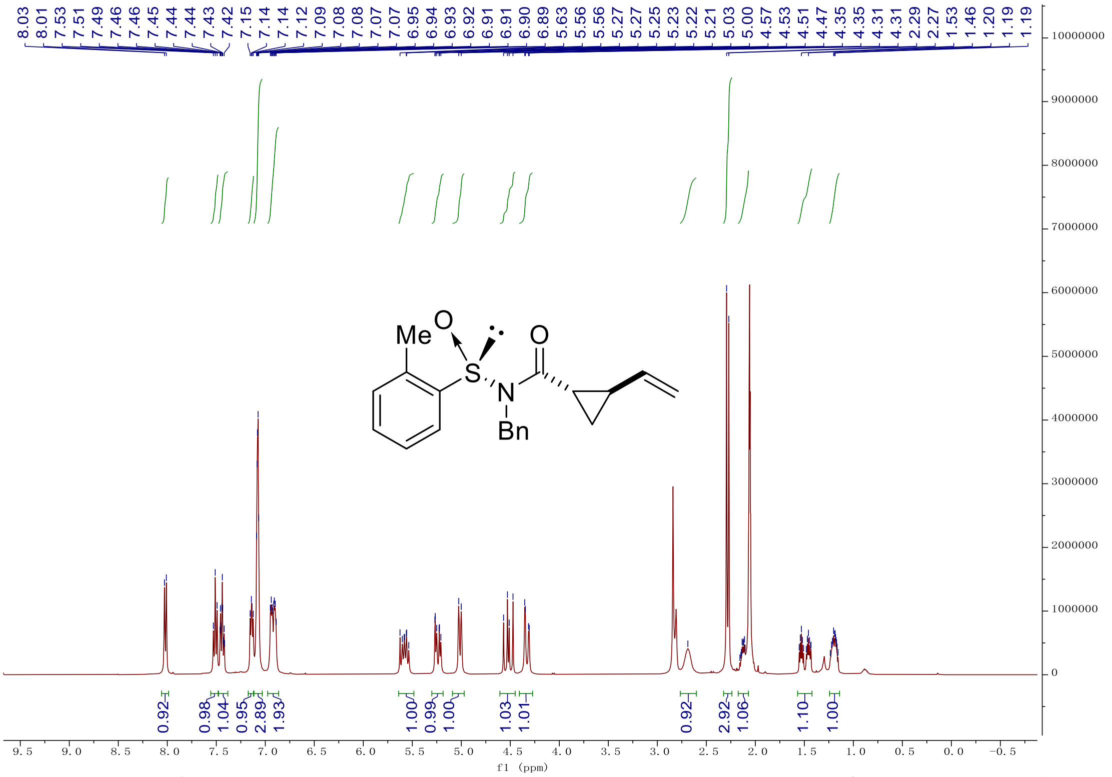

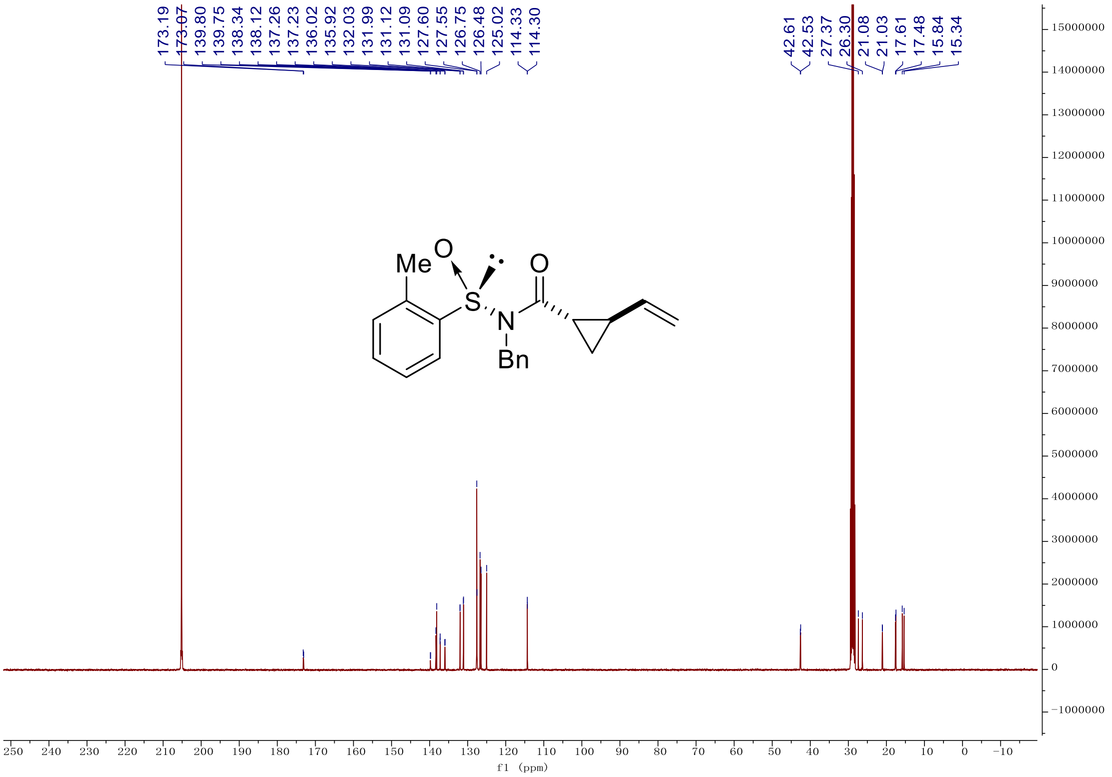

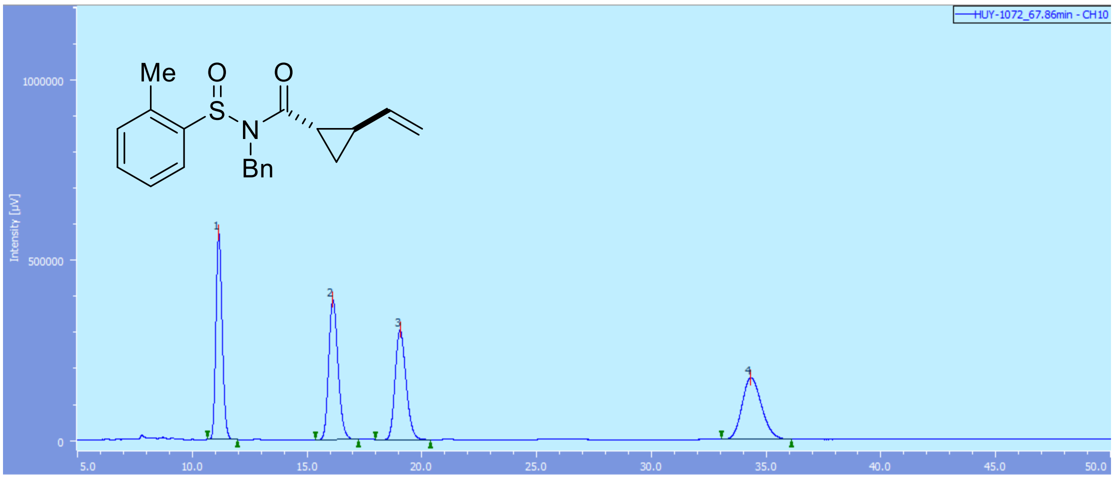

#	Peak Name	CH	tR	Area	Height	Area%	Height%	Quantity	NTP	Resolution	Symmetry Factor	Warning
1	Unknown	10	11.150	10805704	570484	25.824	39.859	N/A	8029	8.028	1.180	
2	Unknown	10	16.117	10979006	387210	26.238	27.054	N/A	7531	3.632	1.207	
3	Unknown	10	19.037	10005573	303017	23.912	21.171	N/A	7669	12.623	1.204	
4	Unknown	10	34.310	10052855	170553	24.025	11.916	N/A	7770	N/A	1.172	

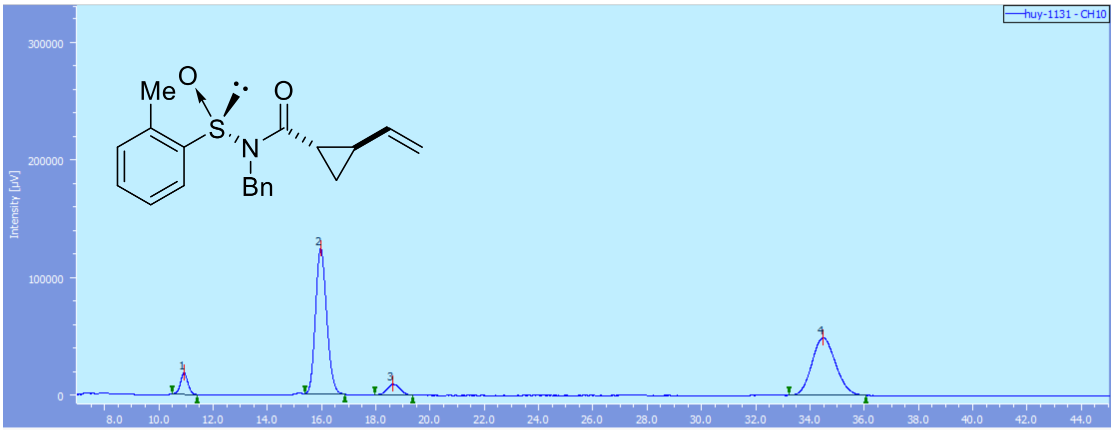

#	Peak Name	CH	tR	Area	Height	Area%	Height%	Quantity	NTP	Resolution	Symmetry Factor	Warning
1	Unknown	10	10.923	351066	18310	4.858	9.148	N/A	7759	8.072	1.028	
2	Unknown	10	15.957	3555268	123592	49.199	61.744	N/A	7159	3.251	1.176	
3	Unknown	10	18.617	311680	9265	4.313	4.628	N/A	7058	12.697	1.133	
4	Unknown	10	34.457	3008222	49000	41.629	24.480	N/A	7280	N/A	1.129	

N-Benzyl-*N*-((*S*)-(2-bromophenyl)sulfinyl)-2-vinylcyclopropane-1-carboxamide (1.6)

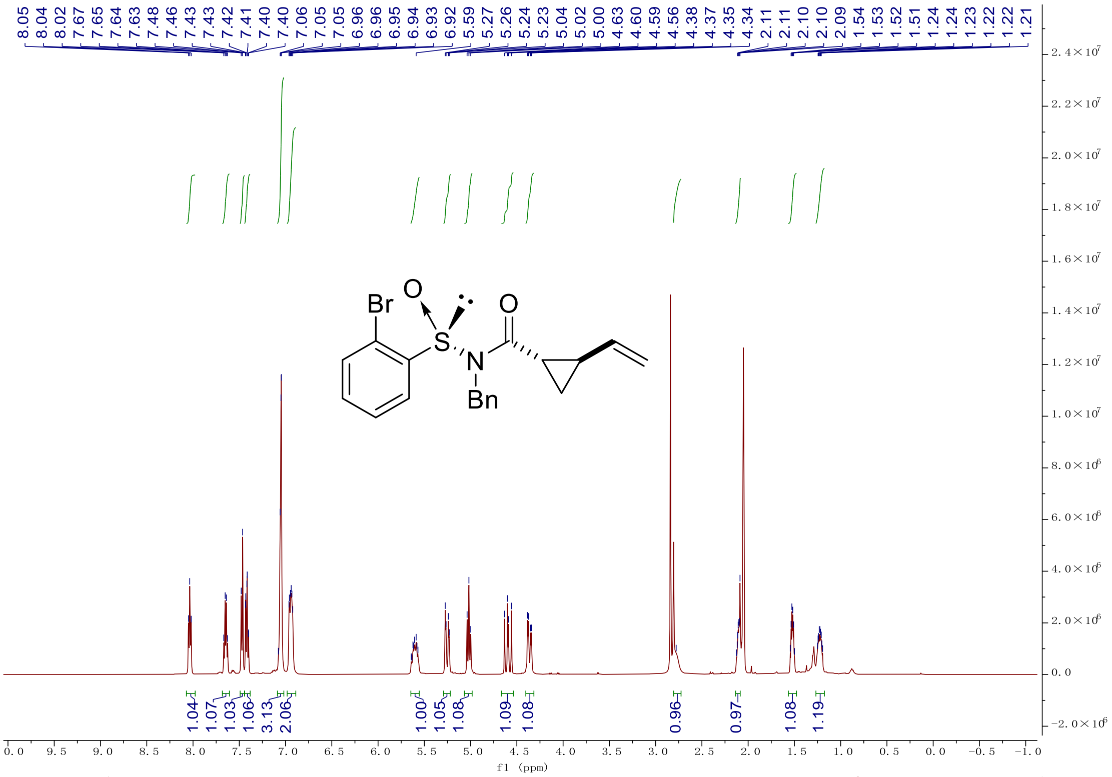

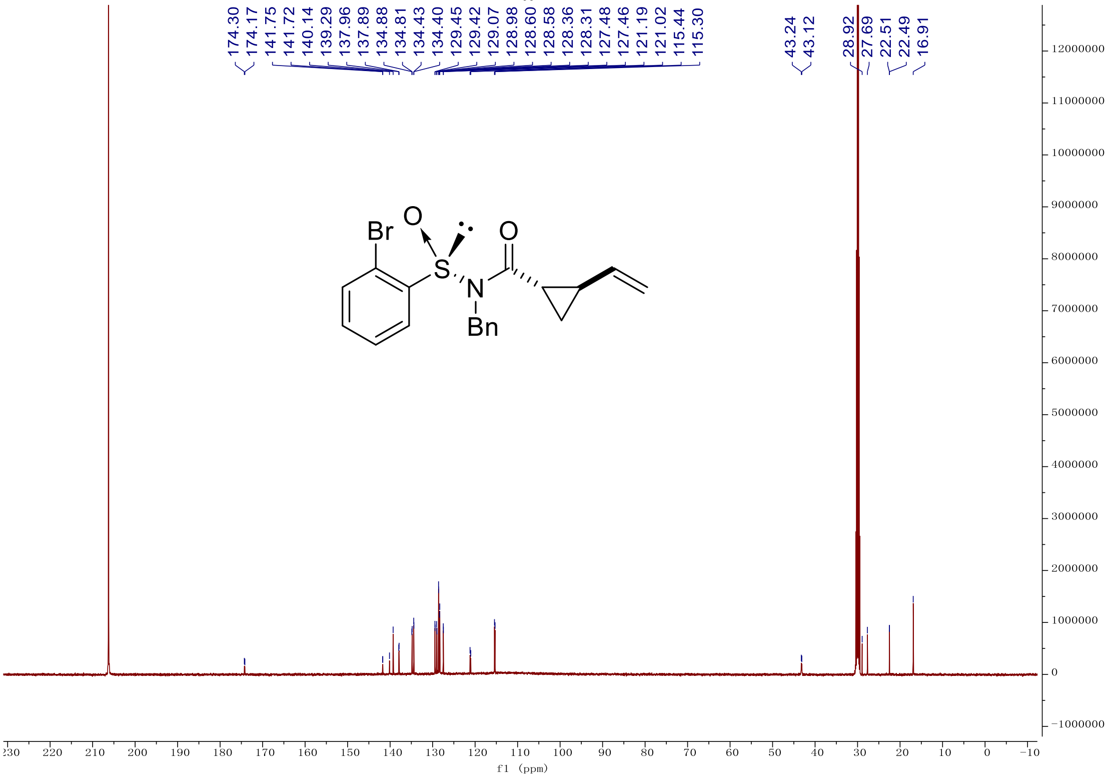

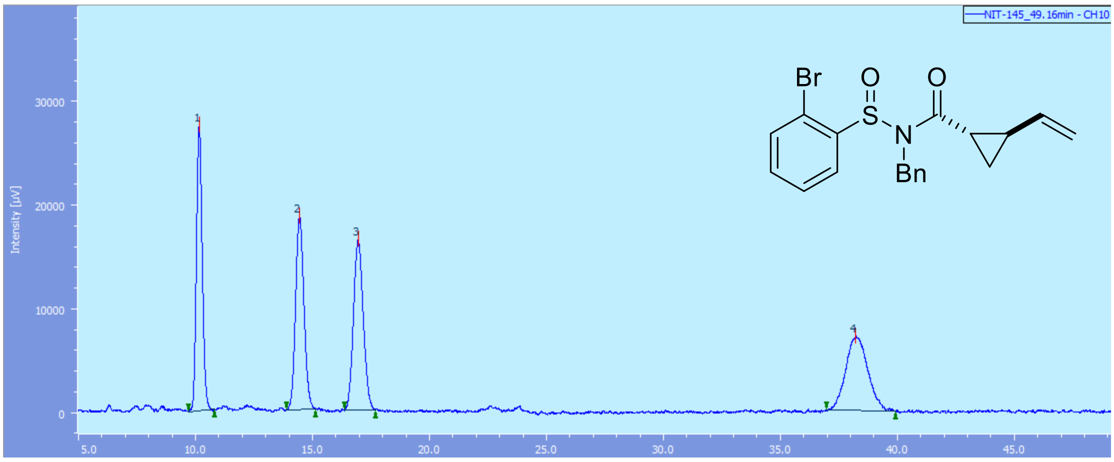

#	Peak Name	CH	tR	Area	Height	Area%	Height%	Quantity	NTP	Resolution	Symmetry Factor	Warning
1	Unknown	10	10.157	474194	27476	25.195	39.448	N/A	8062	7.778	1.123	
2	Unknown	10	14.433	460565	18564	24.471	26.652	N/A	7886	3.546	1.132	
3	Unknown	10	16.943	475013	16410	25.239	23.560	N/A	7760	17.134	1.087	
4	Unknown	10	38.190	472298	7202	25.095	10.340	N/A	7912	N/A	1.138	

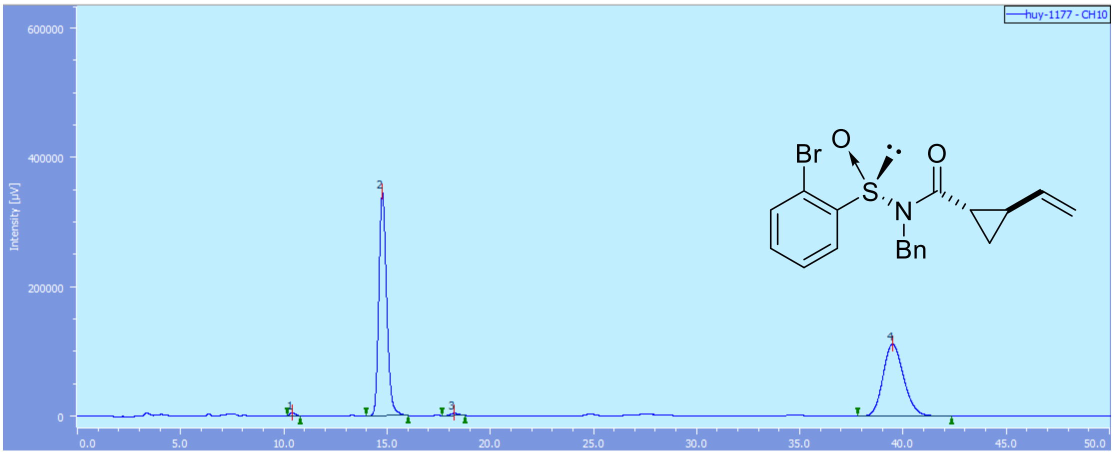

#	Peak Name	CH	tR	Area	Height	Area%	Height%	Quantity	NTP	Resolution	Symmetry Factor	Warning
1	Unknown	10	10.380	83901	4494	0.494	0.969	N/A	6340	7.309	1.312	
2	Unknown	10	14.760	9099751	344943	53.602	74.378	N/A	7531	4.820	1.214	
3	Unknown	10	18.247	93361	3375	0.550	0.728	N/A	8978	16.429	1.117	
4	Unknown	10	39.453	7699387	110961	45.353	23.926	N/A	7533	N/A	1.166	

N-Benzyl-N-((S)-naphthalen-1-ylsulfinyl)-2-vinylcyclopropane-1-carboxamide (1.7)

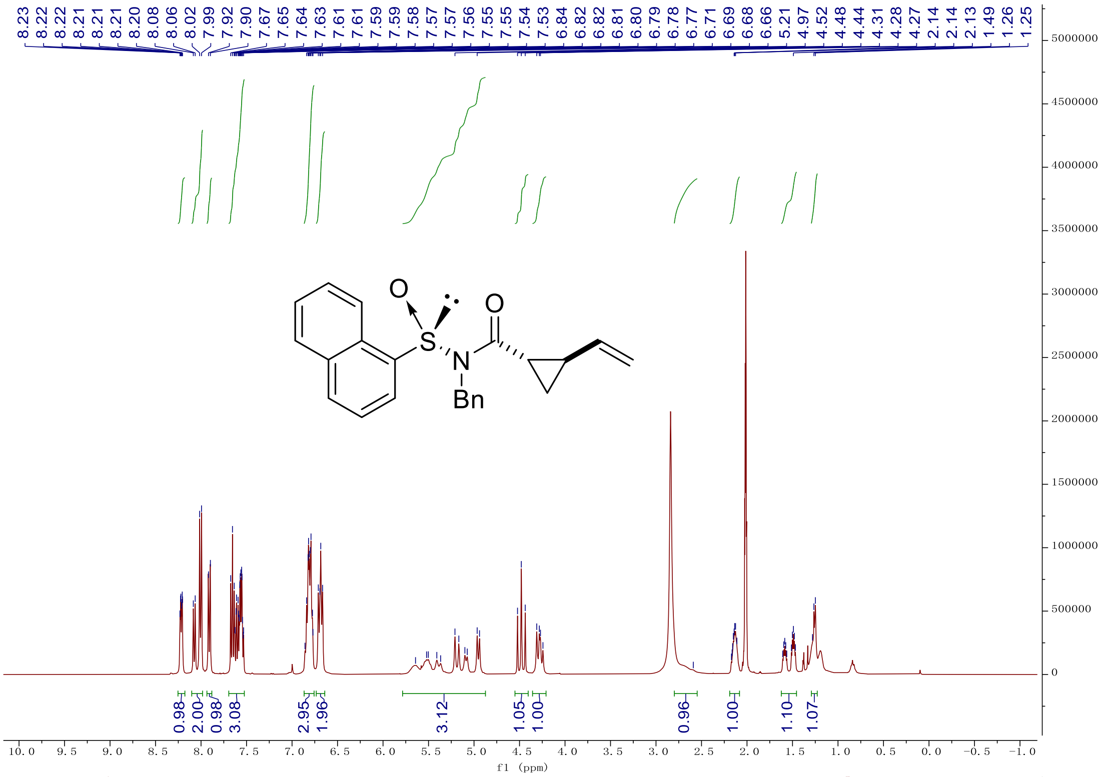

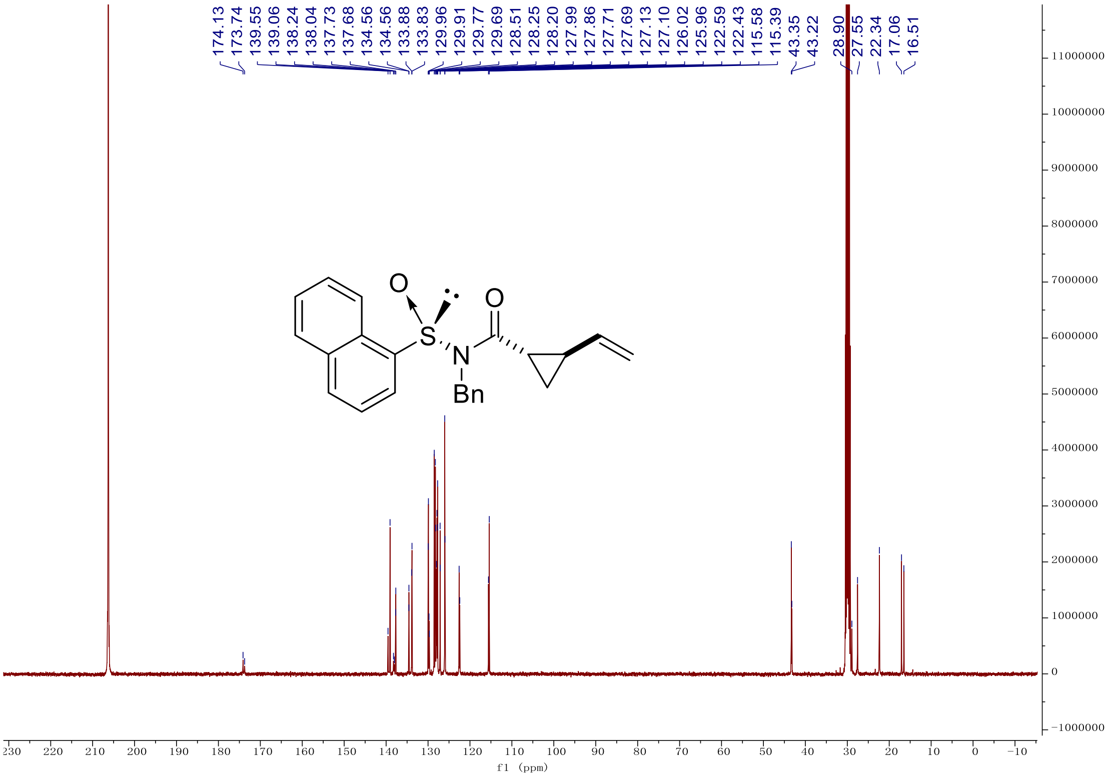

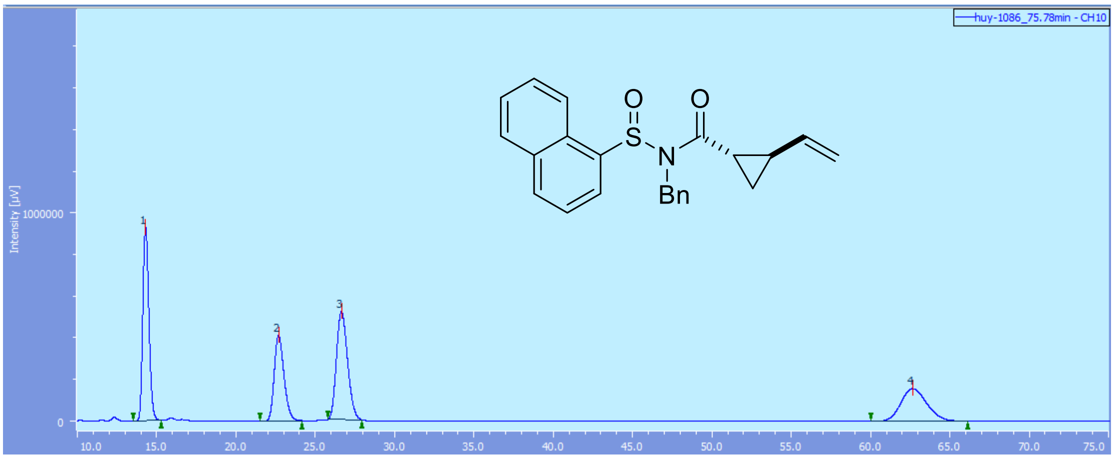

#	Peak Name	CH	tR	Area	Height	Area%	Height%	Quantity	NTP	Resolution	Symmetry Factor	Warning
1	Unknown	10	14.297	24776540	927020	28.949	46.073	N/A	6555	9.185	1.201	
2	Unknown	10	22.670	17538589	409488	20.492	20.352	N/A	6535	3.279	1.253	
3	Unknown	10	26.633	25386419	519075	29.662	25.798	N/A	6696	16.735	1.191	
4	Unknown	10	62.603	17885398	156478	20.897	7.777	N/A	6929	N/A	1.175	

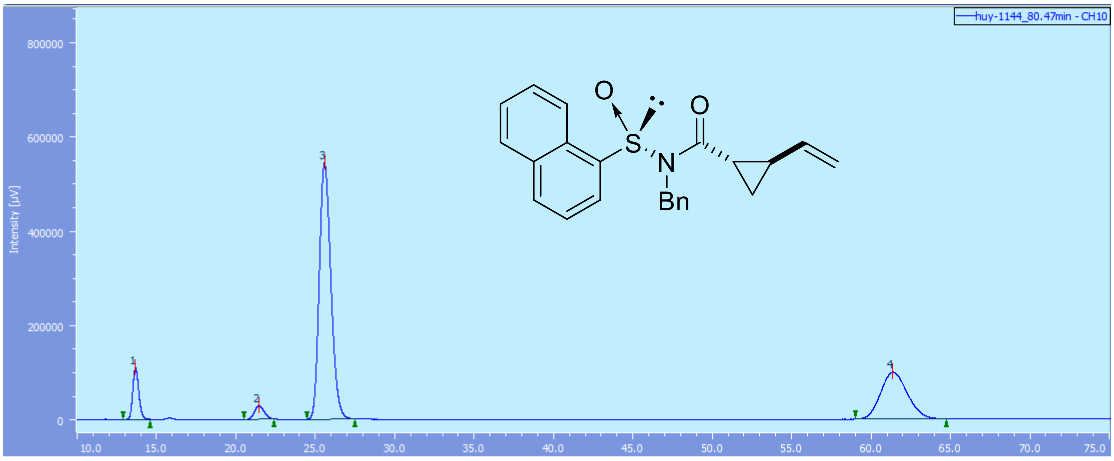

#	Peak Name	CH	tR	Area	Height	Area%	Height%	Quantity	NTP	Resolution	Symmetry Factor	Warning
1	Unknown	10	13.670	2890330	110315	6.684	14.125	N/A	6416	8.624	1.168	
2	Unknown	10	21.457	1199866	28261	2.775	3.619	N/A	5789	3.346	1.120	
3	Unknown	10	25.563	27537190	543330	63.685	69.567	N/A	5883	16.186	1.210	
4	Unknown	10	61.327	11612163	99111	26.855	12.690	N/A	6271	N/A	1.154	

***N*-Benzyl-*N*-((*S*)-naphthalen-2-ylsulfinyl)-2-vinylcyclopropane-1-carboxamide (1.8)**

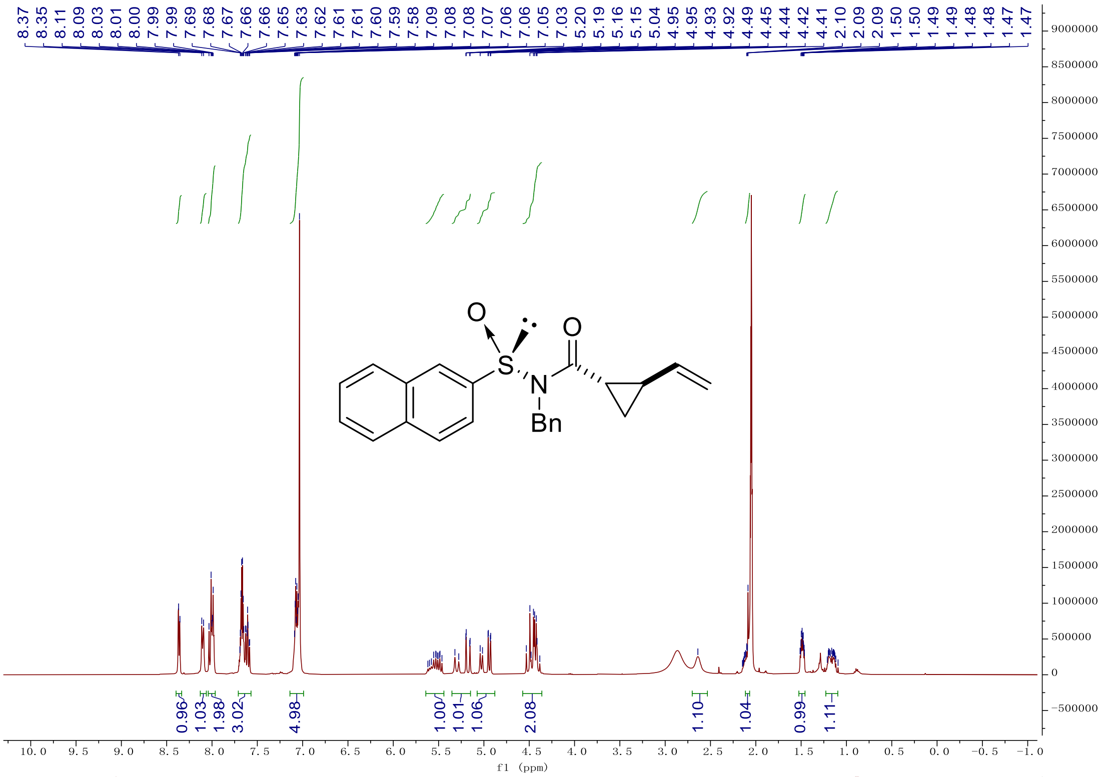

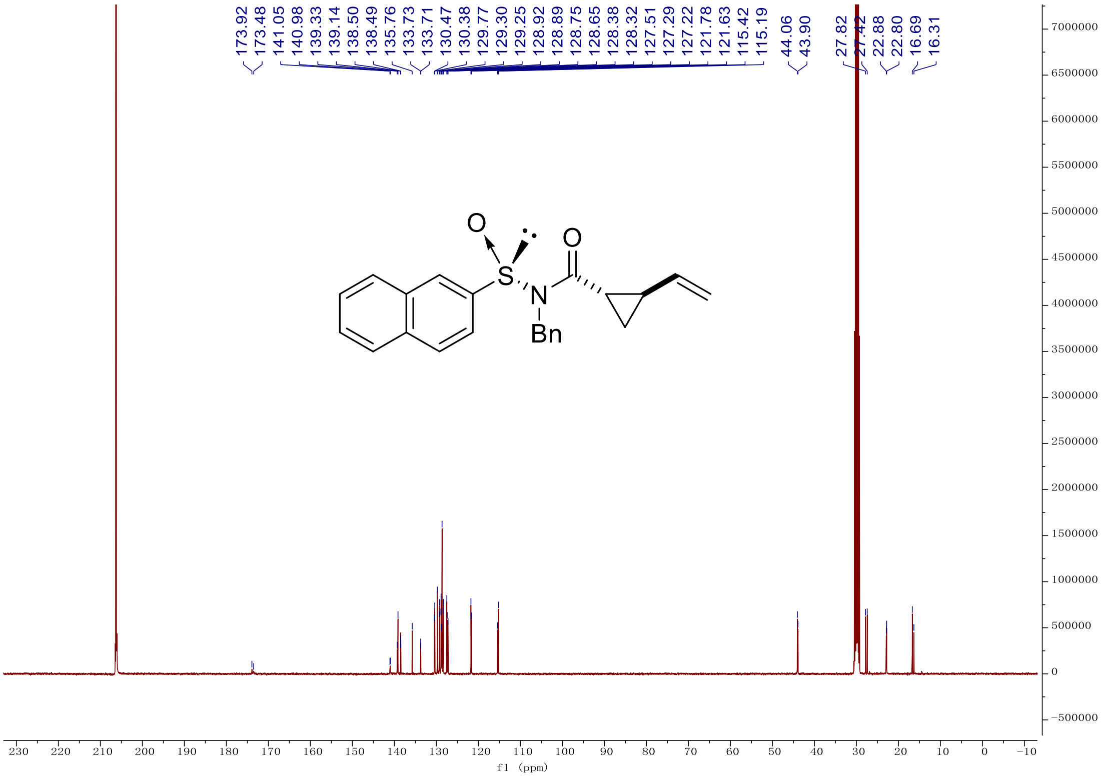

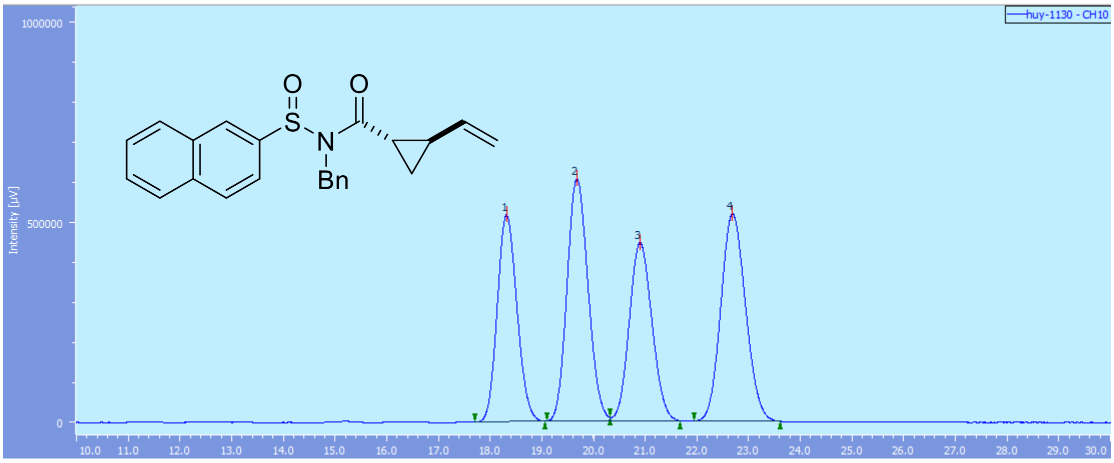

#	Peak Name	CH	tR	Area	Height	Area%	Height%	Quantity	NTP	Resolution	Symmetry Factor	Warning
1	Unknown	10	18.310	13641310	515710	22.186	24.774	N/A	10884	1.875	1.124	
2	Unknown	10	19.670	17074796	604562	27.770	29.042	N/A	10942	1.563	1.125	
3	Unknown	10	20.890	13585137	444345	22.094	21.345	N/A	10562	2.113	1.097	
4	Unknown	10	22.677	17186112	517078	27.951	24.839	N/A	10570	N/A	1.109	

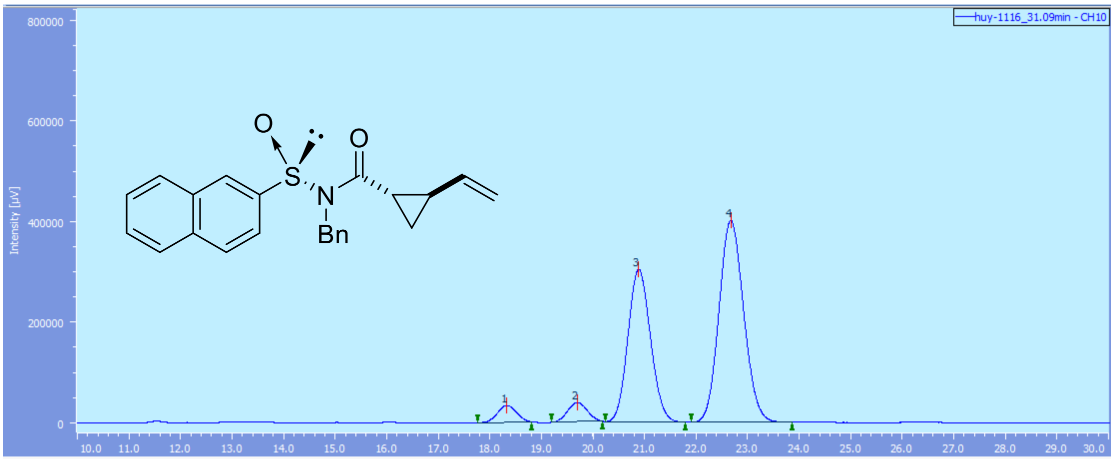

#	Peak Name	CH	tR	Area	Height	Area%	Height%	Quantity	NTP	Resolution	Symmetry Factor	Warning
1	Unknown	10	18.327	859537	33442	3.528	4.320	N/A	11255	1.938	1.017	
2	Unknown	10	19.693	1009452	38040	4.143	4.914	N/A	11867	1.549	1.005	
3	Unknown	10	20.877	9201120	302469	37.763	39.076	N/A	10657	2.118	1.103	
4	Unknown	10	22.660	13295100	400100	54.566	51.689	N/A	10616	N/A	1.104	

N-Benzyl-*N*-((*S*)-thiophen-2-ylsulfinyl)-2-vinylcyclopropane-1-carboxamide (1.9)

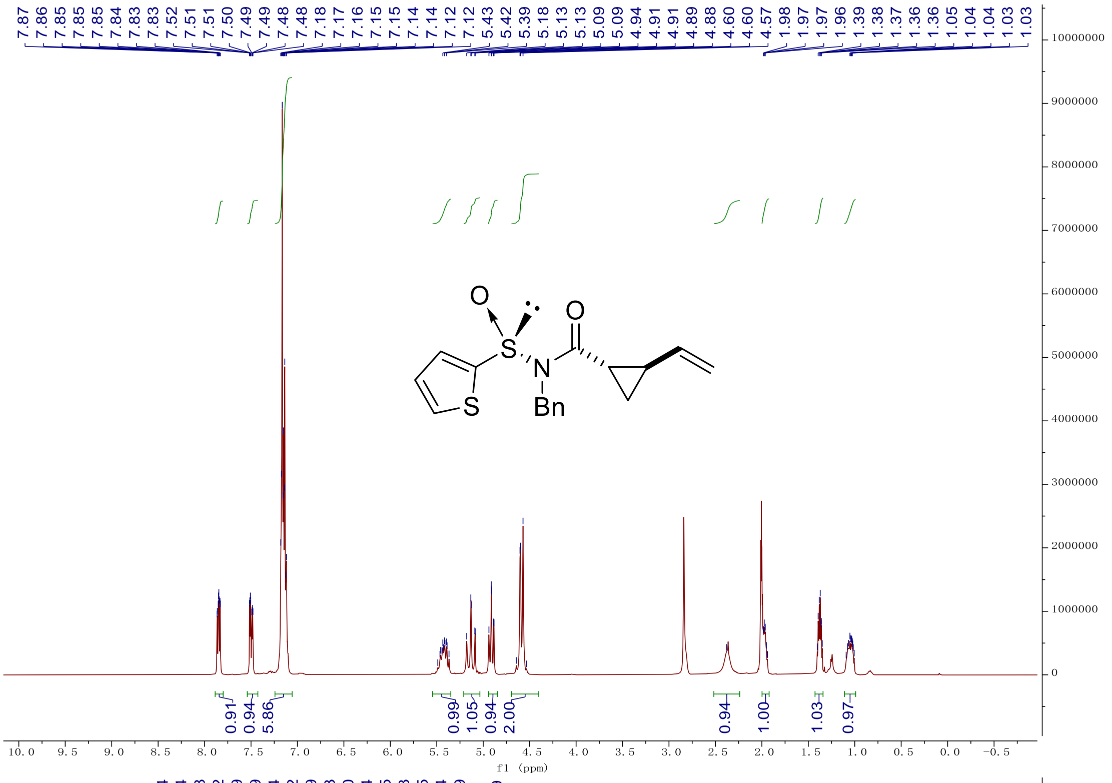

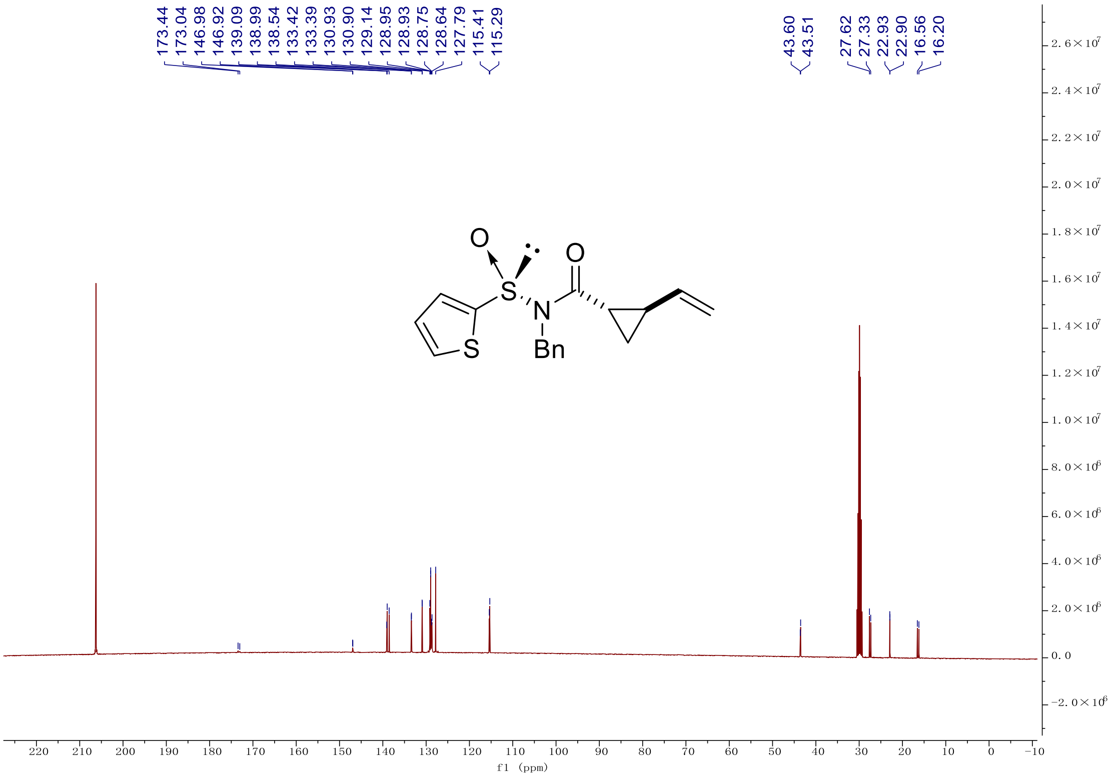

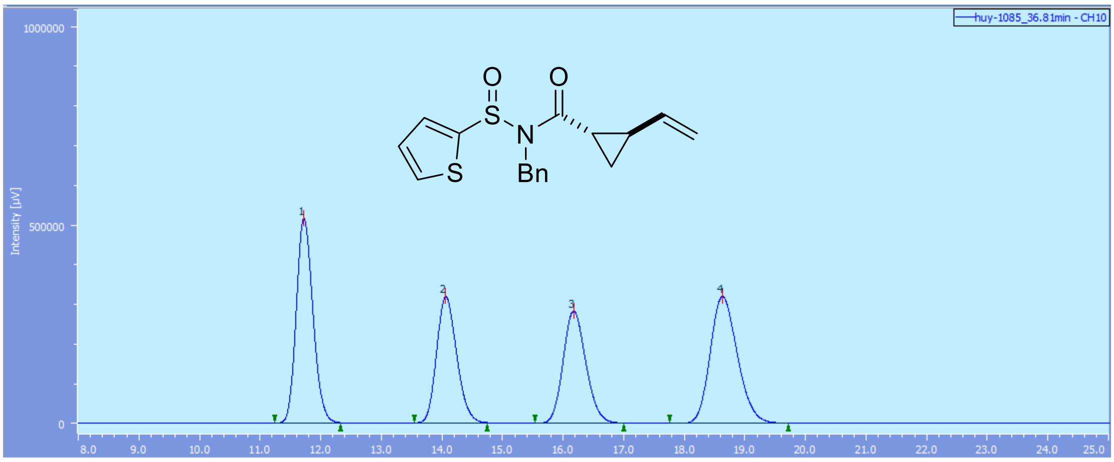

#	Peak Name	CH	tR	Area	Height	Area%	Height%	Quantity	NTP	Resolution	Symmetry Factor	Warning
1	Unknown	10	11.720	9820036	514790	28.582	35.944	N/A	8735	4.235	1.166	
2	Unknown	10	14.057	7274323	317165	21.173	22.145	N/A	8644	3.276	1.183	
3	Unknown	10	16.167	7355317	281748	21.408	19.672	N/A	8865	3.267	1.148	
4	Unknown	10	18.620	9907638	318501	28.837	22.239	N/A	8270	N/A	1.190	

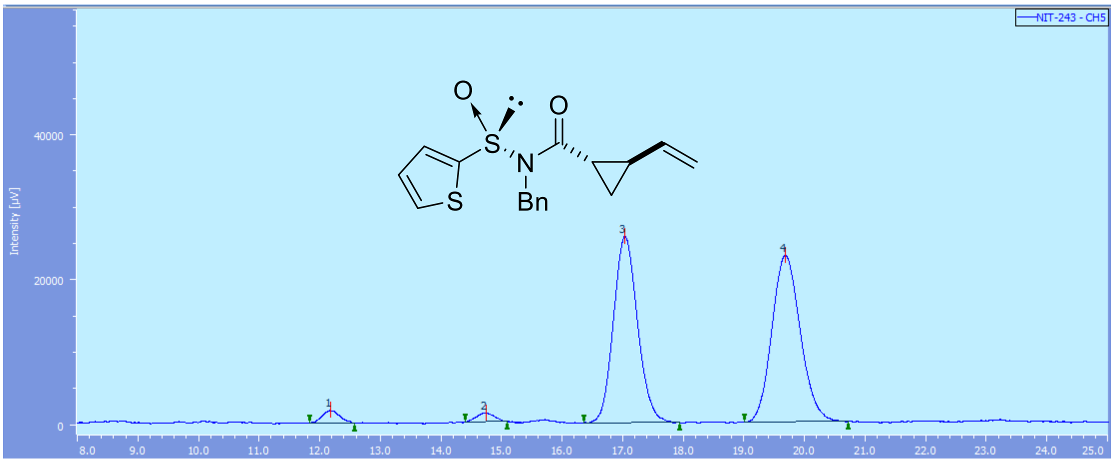

#	Peak Name	CH	tR	Area	Height	Area%	Height%	Quantity	NTP	Resolution	Symmetry Factor	Warning
1	Unknown	5	12.173	34162	1754	2.262	3.402	N/A	8746	4.781	1.123	
2	Unknown	5	14.737	24838	1225	1.645	2.378	N/A	11302	3.604	0.926	
3	Unknown	5	17.030	707298	25651	46.841	49.769	N/A	8912	3.360	1.122	
4	Unknown	5	19.670	743710	22909	49.252	44.451	N/A	8491	N/A	1.140	

N-(4-Methoxybenzyl)-*N*-((*S*)-*p*-tolylsulfinyl)-2-vinylcyclopropane-1-carboxamide (1.10)

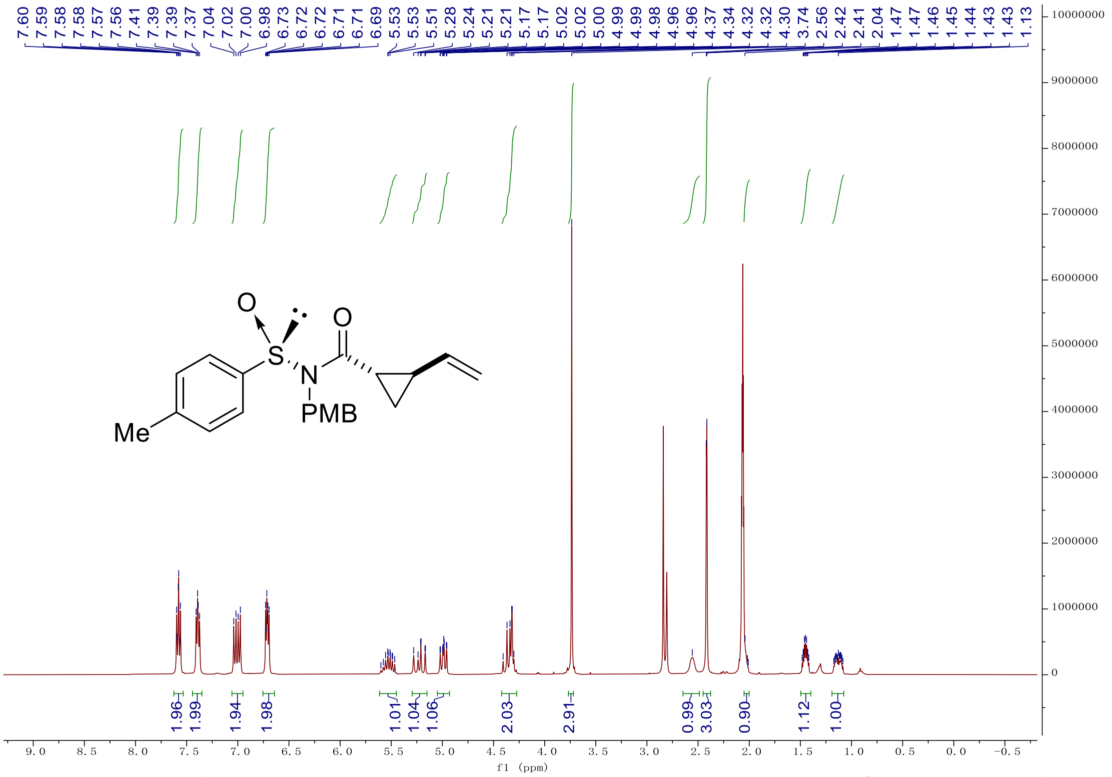

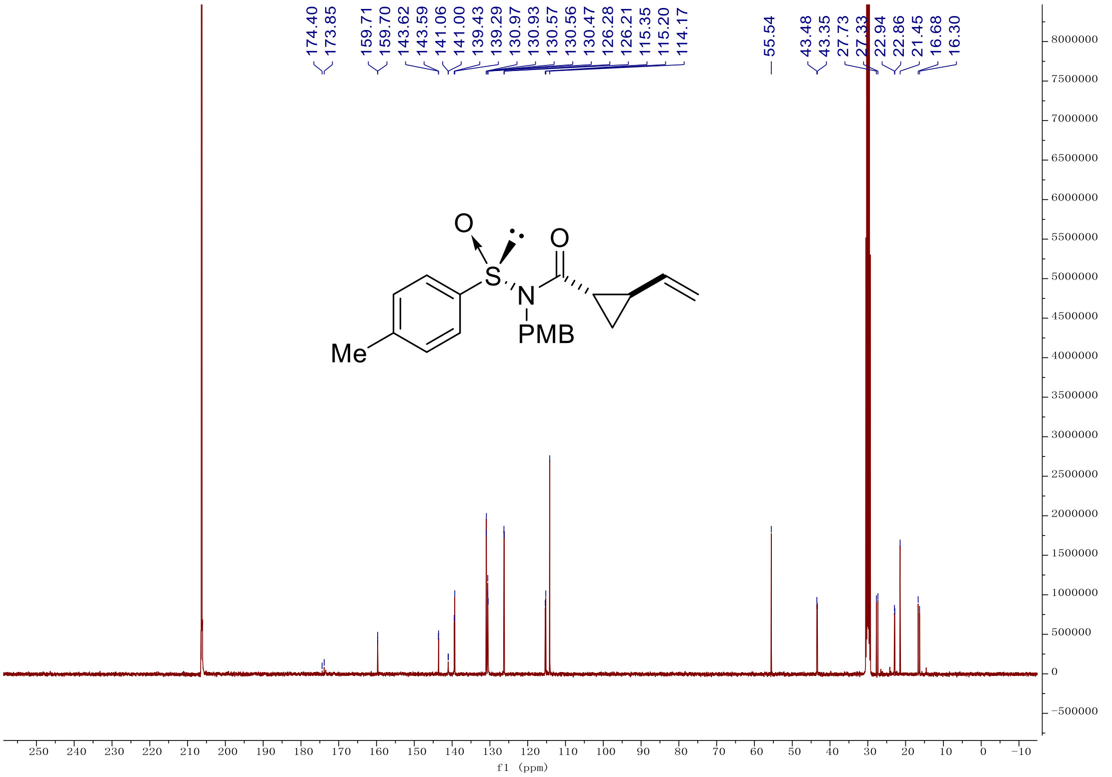

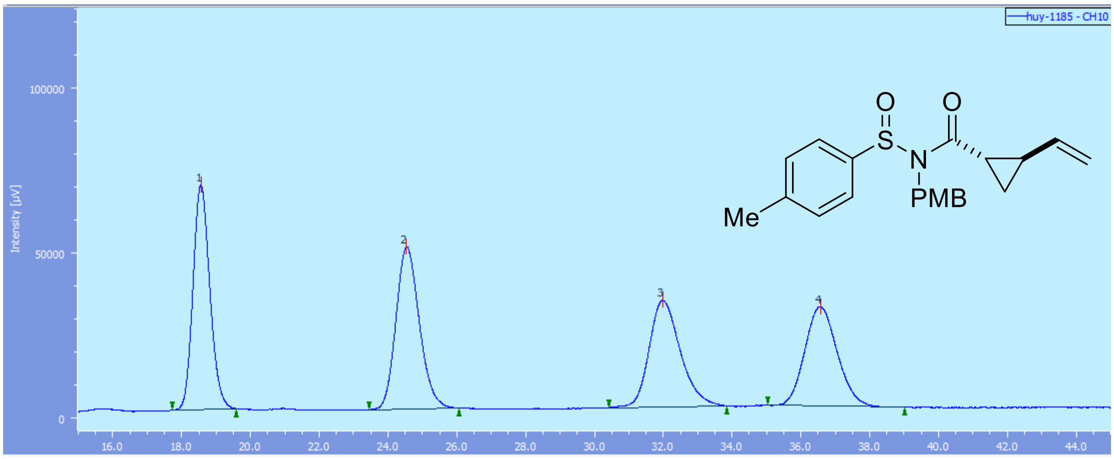

#	Peak Name	CH	tR	Area	Height	Area%	Height%	Quantity	NTP	Resolution	Symmetry Factor	Warning
1	Unknown	10	18.557	2315863	68001	26.847	37.919	N/A	6971	5.644	1.156	
2	Unknown	10	24.530	2322501	49103	26.924	27.381	N/A	6329	5.228	1.177	
3	Unknown	10	31.963	2014696	32150	23.356	17.927	N/A	6247	2.751	1.279	
4	Unknown	10	36.557	1973121	30080	22.874	16.773	N/A	7142	N/A	1.122	

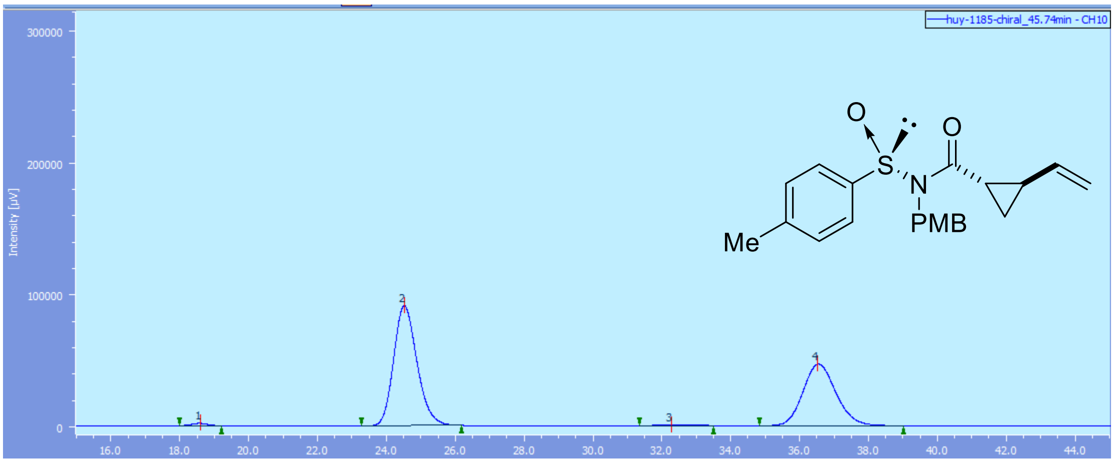

#	Peak Name	CH	tR	Area	Height	Area%	Height%	Quantity	NTP	Resolution	Symmetry Factor	Warning
1	Unknown	10	18.603	66570	1934	0.889	1.381	N/A	6541	5.519	1.004	
2	Unknown	10	24.507	4276468	90889	57.089	64.909	N/A	6402	4.768	1.202	
3	Unknown	10	32.270	41968	653	0.560	0.466	N/A	4004	2.252	1.196	
4	Unknown	10	36.513	3105905	46549	41.462	33.243	N/A	7059	N/A	1.153	

N-Methyl-*N*-((*S*)-*p*-tolylsulfinyl)-2-vinylcyclopropane-1-carboxamide (1.11)

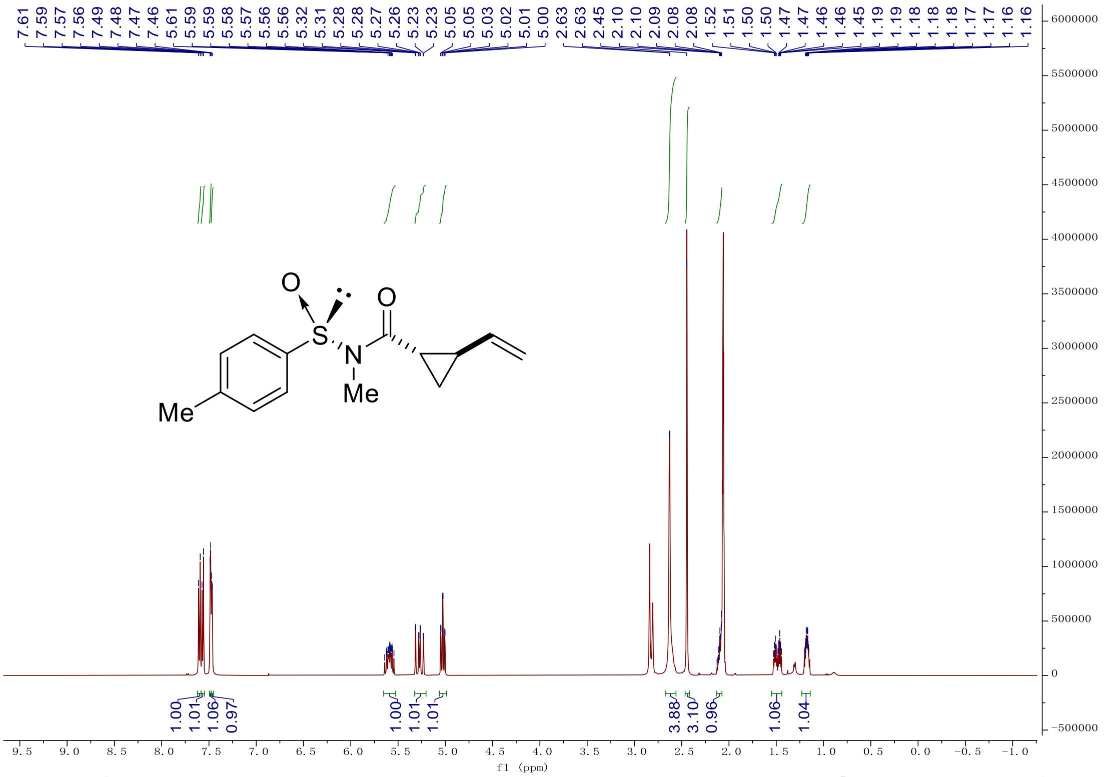

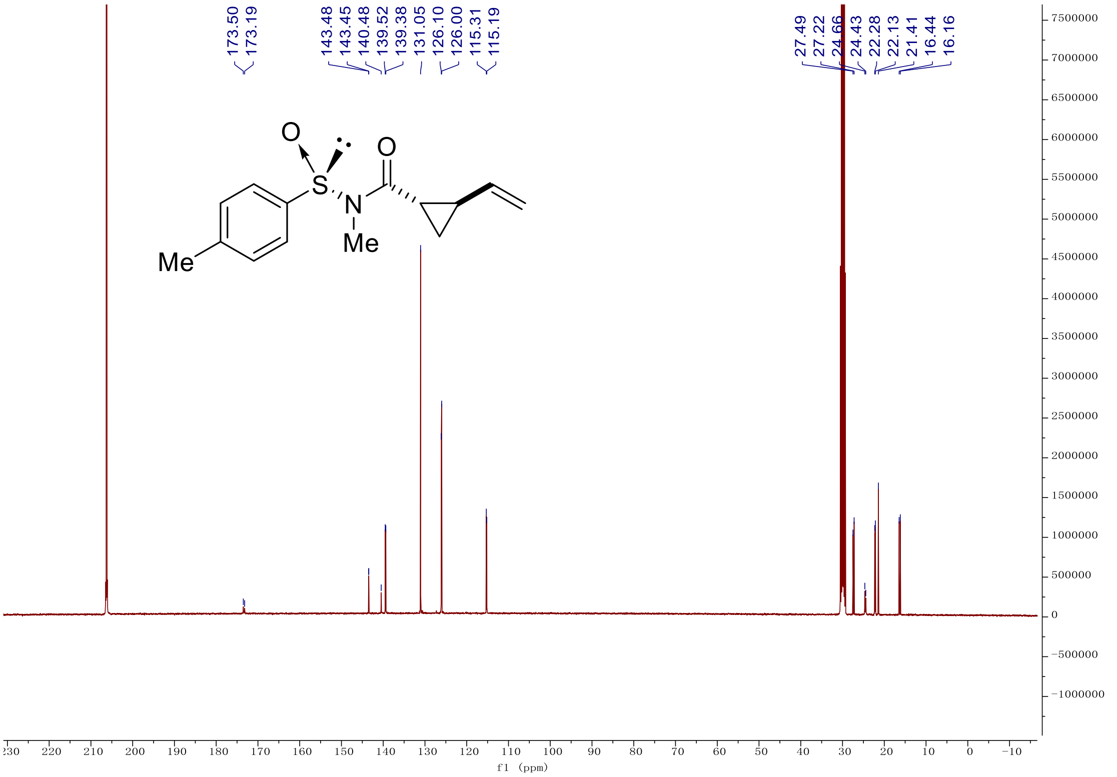

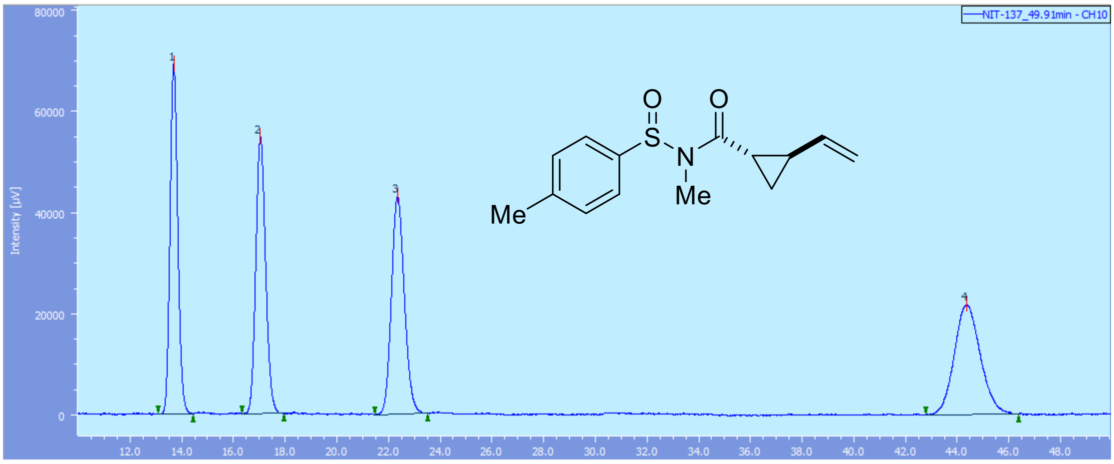

#	Peak Name	CH	tR	Area	Height	Area%	Height%	Quantity	NTP	Resolution	Symmetry Factor	Warning
1	Unknown	10	13.687	1442946	68947	24.537	36.670	N/A	10025	5.422	1.129	
2	Unknown	10	17.033	1434692	54428	24.396	28.948	N/A	9720	6.650	1.137	
3	Unknown	10	22.340	1490570	42942	25.347	22.839	N/A	9662	16.162	1.125	
4	Unknown	10	44.337	1512559	21705	25.720	11.544	N/A	9494	N/A	1.113	

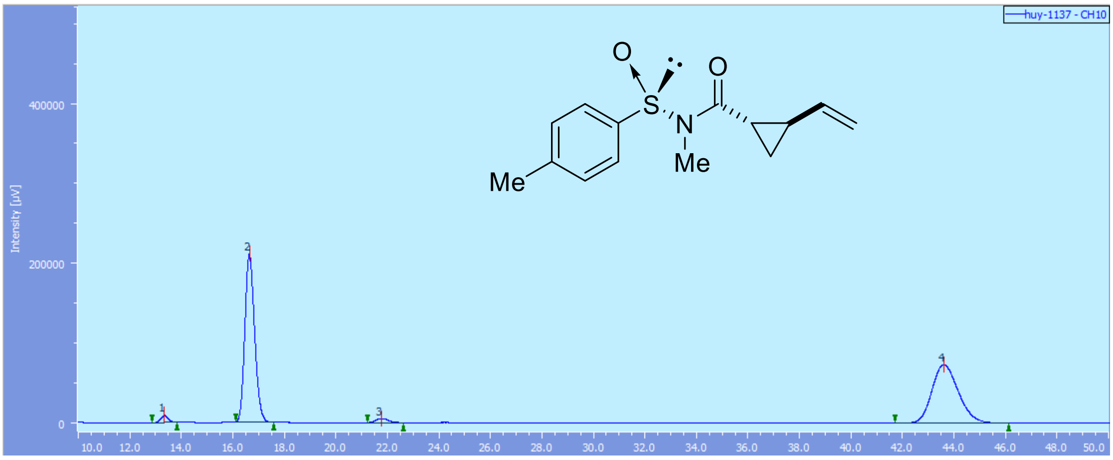

#	Peak Name	CH	tR	Area	Height	Area%	Height%	Quantity	NTP	Resolution	Symmetry Factor	Warning
1	Unknown	10	13.340	183395	8912	1.653	2.998	N/A	9502	5.336	1.152	
2	Unknown	10	16.637	5520411	209890	49.764	70.606	N/A	9262	6.495	1.168	
3	Unknown	10	21.763	176708	5271	1.593	1.773	N/A	9540	15.818	1.233	
4	Unknown	10	43.570	5212729	73196	46.990	24.623	N/A	8656	N/A	1.214	

N-Propyl-*N*-((*S*)-*p*-tolylsulfinyl)-2-vinylcyclopropane-1-carboxamide (1.12)

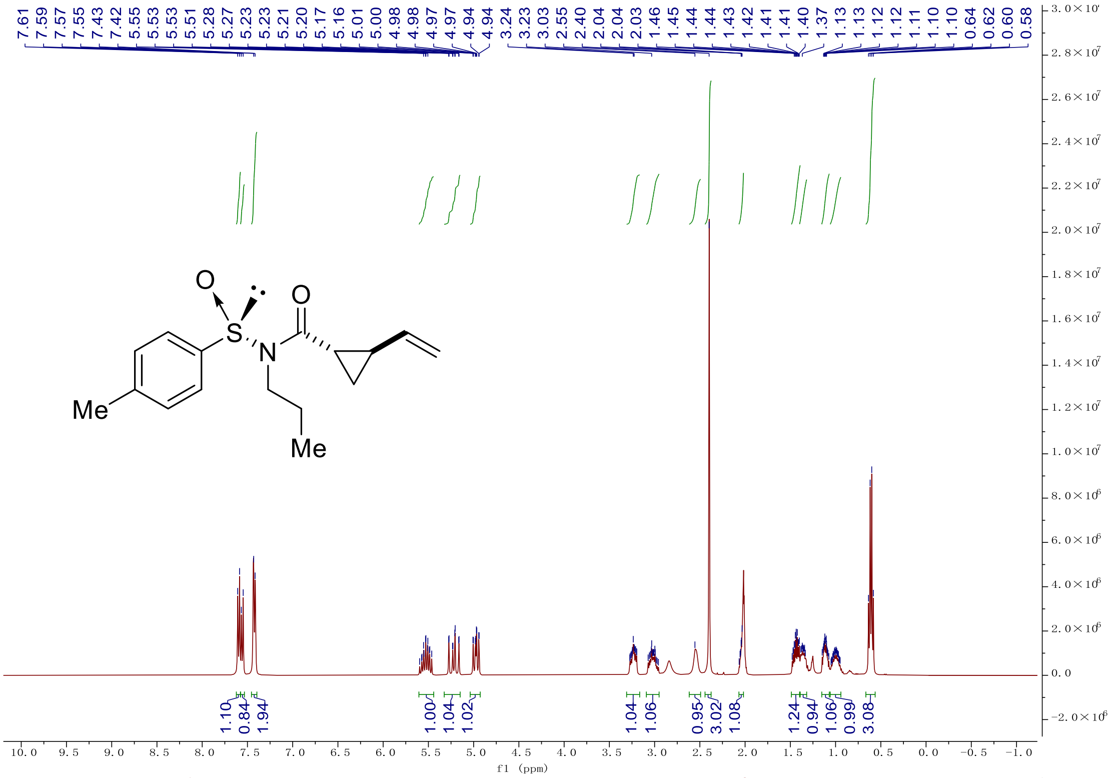

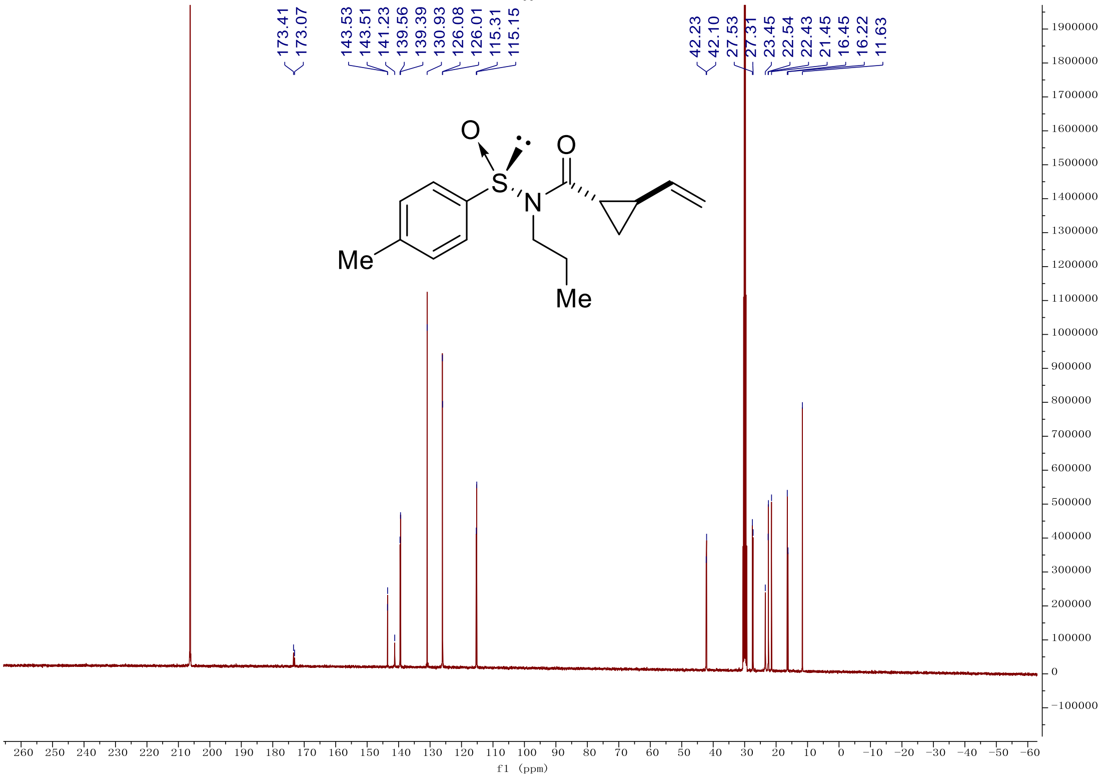

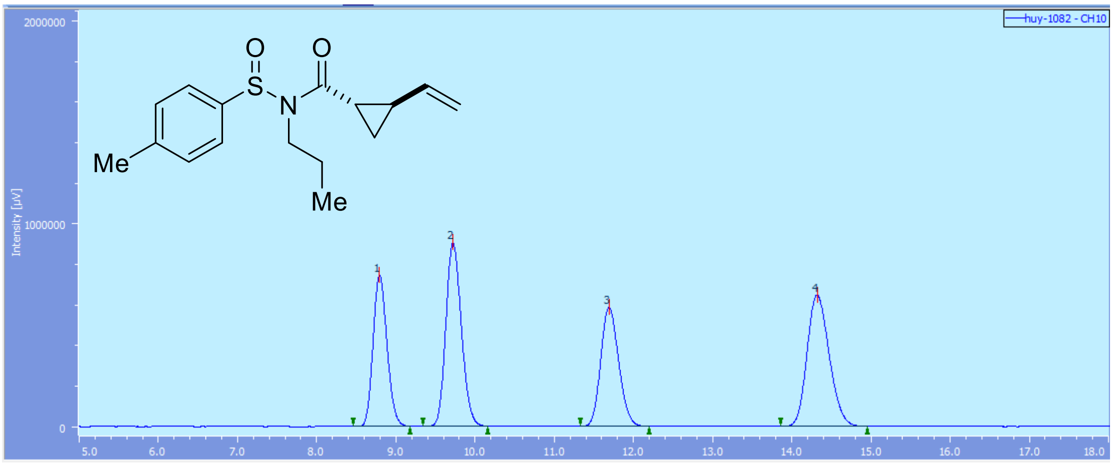

#	Peak Name	CH	tR	Area	Height	Area%	Height%	Quantity	NTP	Resolution	Symmetry Factor	Warning
1	Unknown	10	8.790	9083291	744244	21.048	25.855	N/A	11948	2.730	1.152	
2	Unknown	10	9.717	12284184	902286	28.465	31.345	N/A	11713	5.094	1.159	
3	Unknown	10	11.687	9215965	585580	21.356	20.343	N/A	12603	5.645	1.146	
4	Unknown	10	14.310	12571527	646428	29.131	22.457	N/A	12328	N/A	1.184	

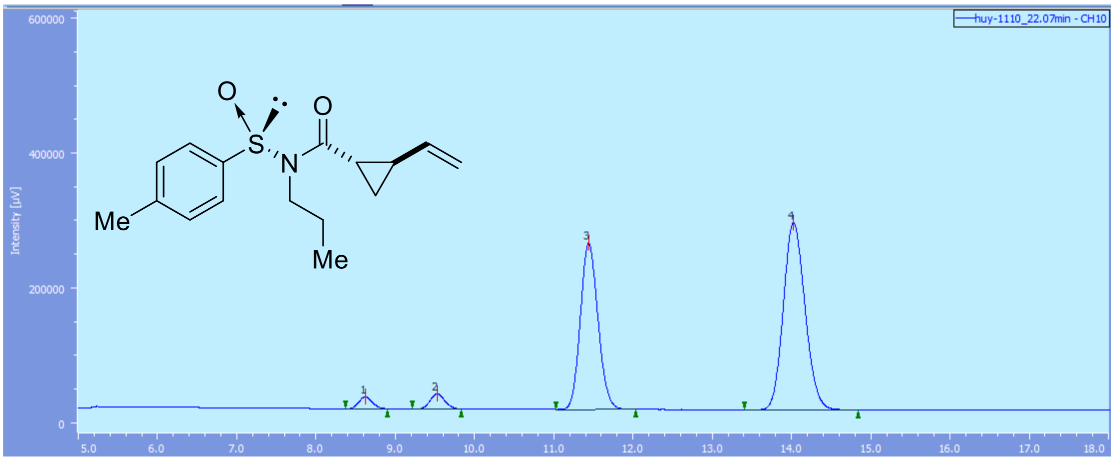

#	Peak Name	CH	tR	Area	Height	Area%	Height%	Quantity	NTP	Resolution	Symmetry Factor	Warning
1	Unknown	10	8.617	208791	17635	2.186	3.131	N/A	12082	2.767	1.117	
2	Unknown	10	9.523	289179	22313	3.027	3.962	N/A	12291	5.107	1.093	
3	Unknown	10	11.433	3803183	246343	39.813	43.741	N/A	12661	5.712	1.130	
4	Unknown	10	14.017	5251392	276890	54.974	49.165	N/A	12548	N/A	1.126	

N-Benzyl-2-(prop-1-en-2-yl)-*N*-((*S*)-*p*-tolylsulfinyl)cyclopropane-1-carboxamide (1.13)

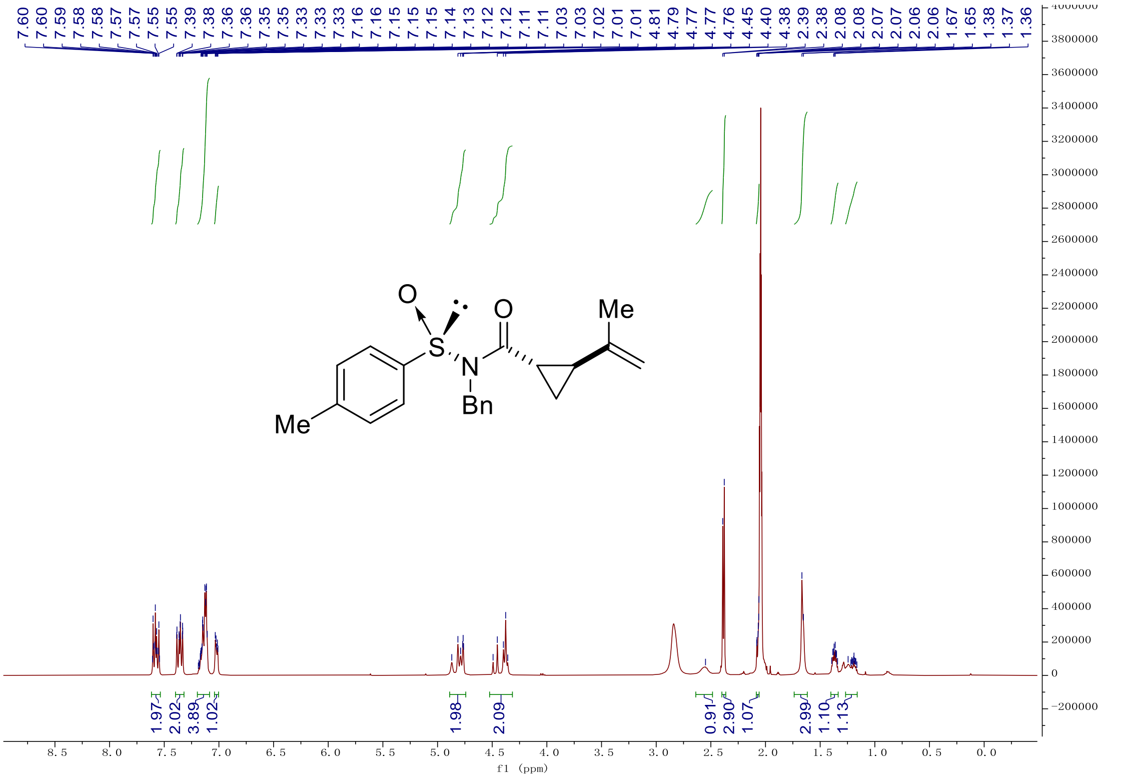

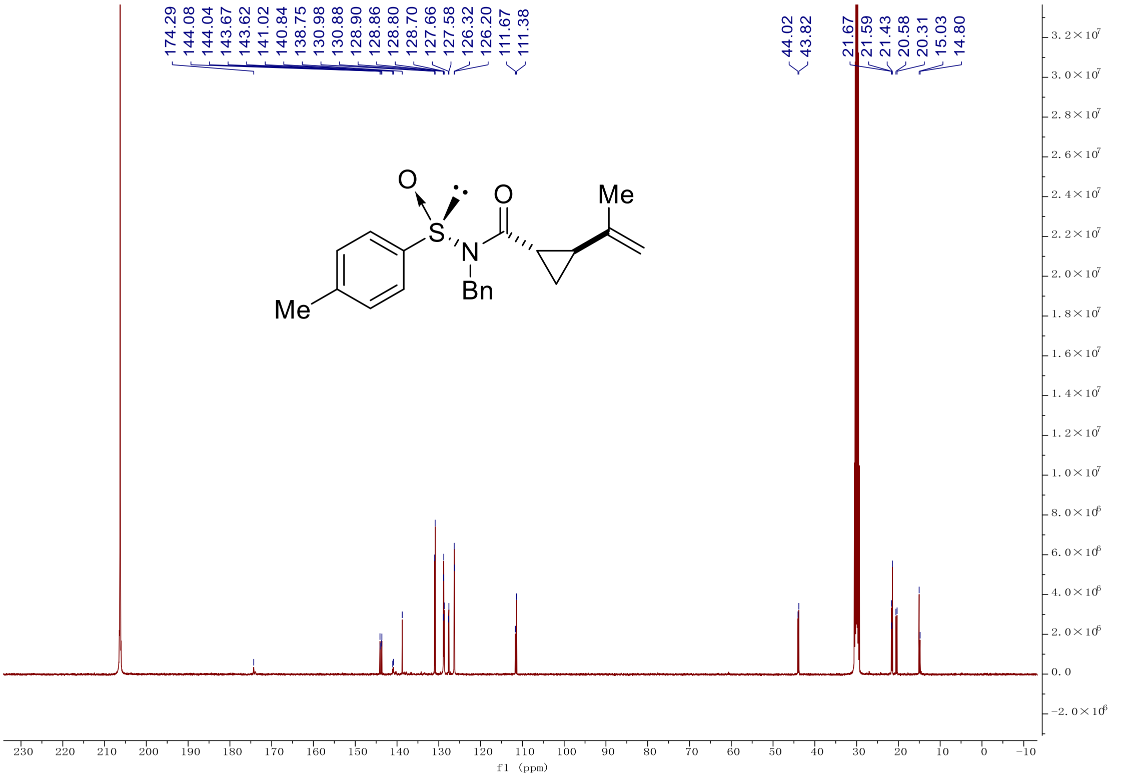

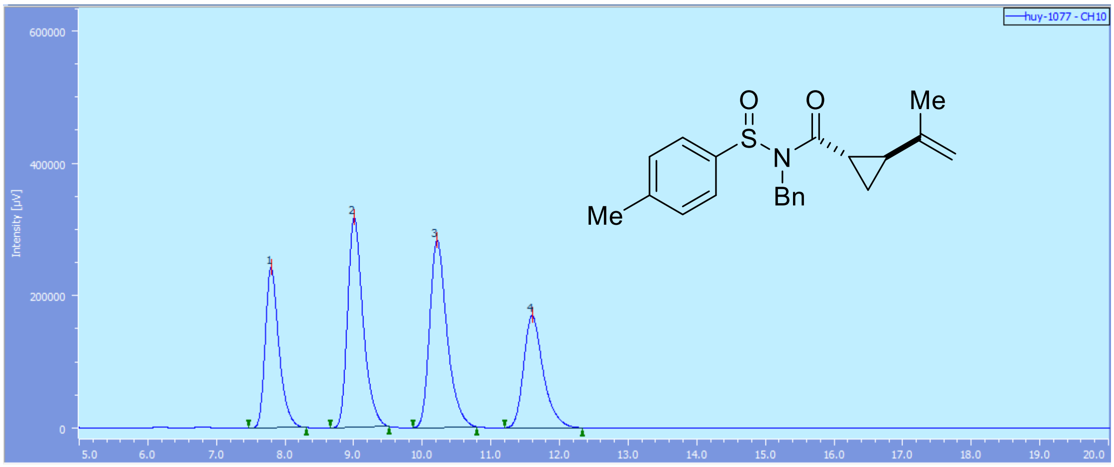

#	Peak Name	CH	tR	Area	Height	Area%	Height%	Quantity	NTP	Resolution	Symmetry Factor	Warning
1	Unknown	10	7.793	3341894	241611	20.114	23.950	N/A	7878	3.234	1.362	
2	Unknown	10	9.003	4952449	316033	29.807	31.327	N/A	8141	2.867	1.354	
3	Unknown	10	10.213	4967338	282198	29.897	27.973	N/A	8348	2.916	1.342	
4	Unknown	10	11.593	3353254	168985	20.182	16.751	N/A	8535	N/A	1.341	

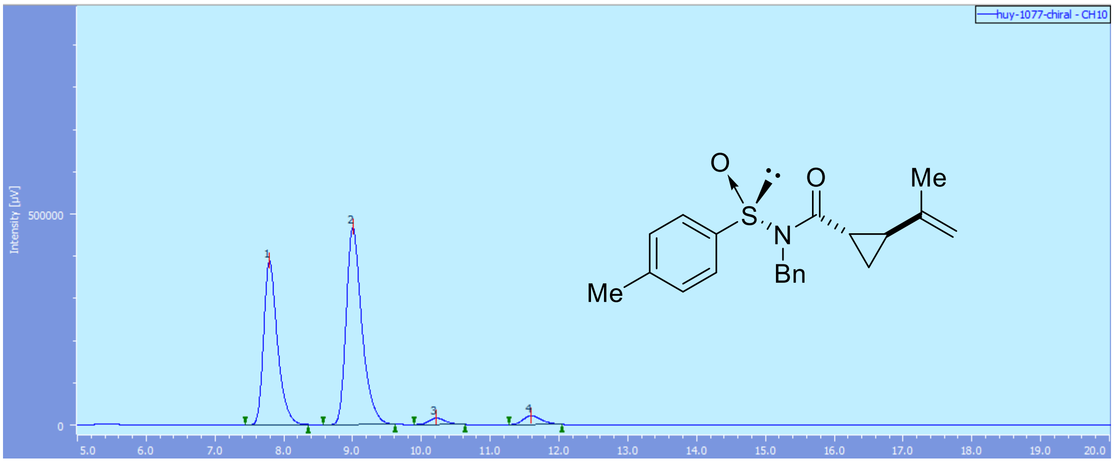

#	Peak Name	CH	tR	Area	Height	Area%	Height%	Quantity	NTP	Resolution	Symmetry Factor	Warning
1	Unknown	10	7.790	5368163	387436	40.081	43.563	N/A	7889	3.226	1.368	
2	Unknown	10	9.000	7388394	466285	55.165	52.429	N/A	8044	2.922	1.373	
3	Unknown	10	10.213	251280	15040	1.876	1.691	N/A	8973	3.006	1.267	
4	Unknown	10	11.590	385378	20600	2.877	2.316	N/A	9054	N/A	1.258	

_N_-Benzyl-2-methyl-_N_-((_S_)-_p_-tolylsulfinyl)-2-vinylcyclopropane-1-carboxamide (1.14)

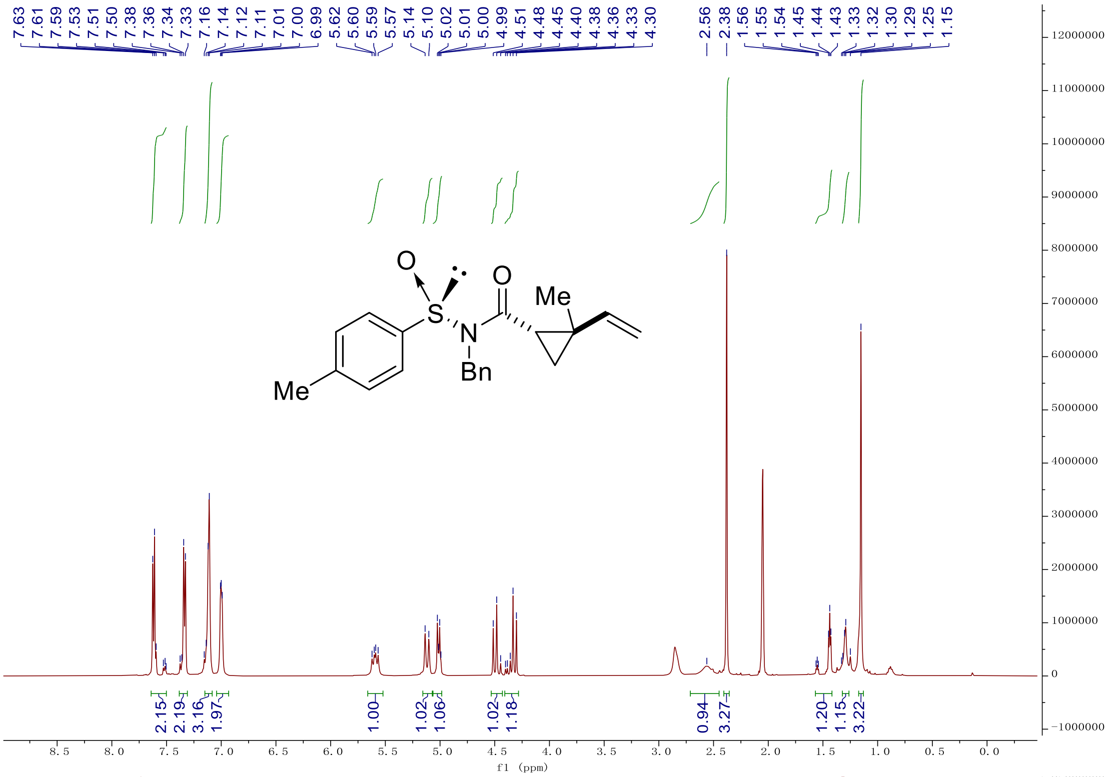

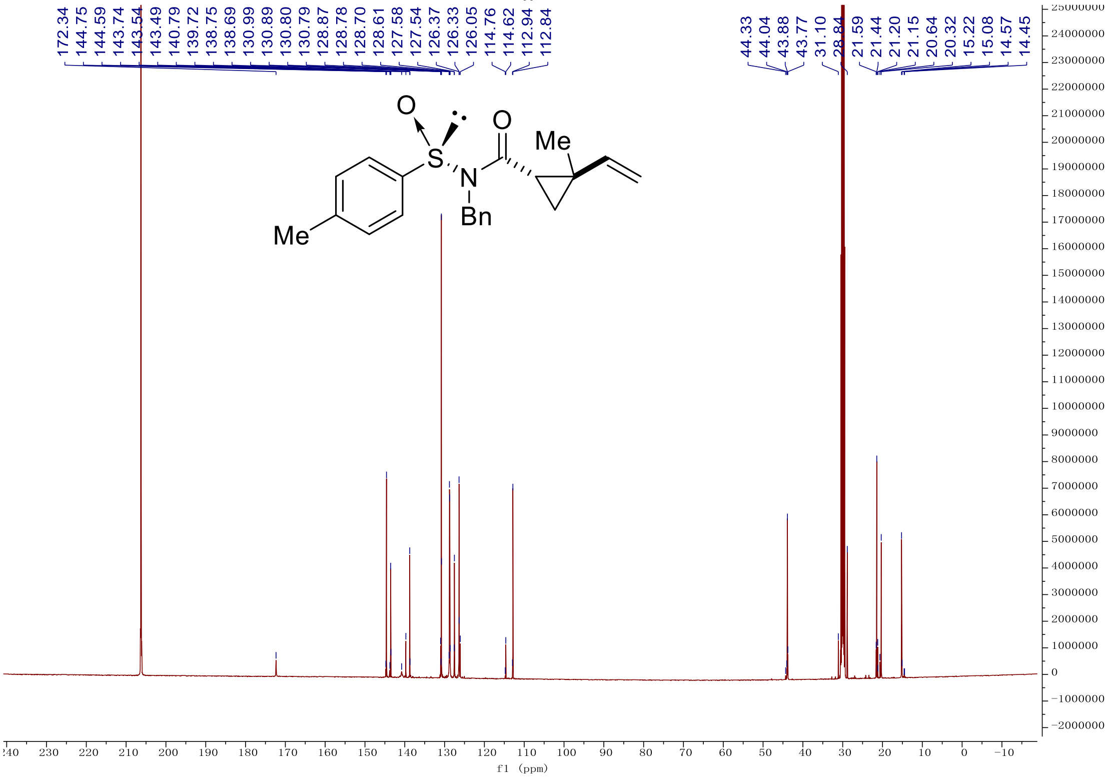

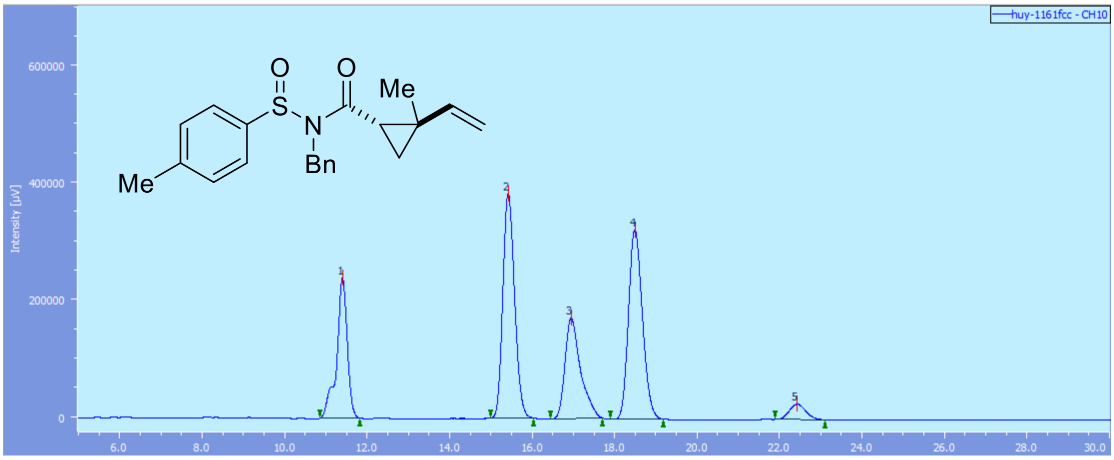

#	Peak Name	CH	tR	Area	Height	Area%	Height%	Quantity	NTP	Resolution	Symmetry Factor	Warning
1	Unknown	10	11.400	4285509	238039	17.442	20.884	N/A	12438	8.656	0.813	
2	Unknown	10	15.413	7524213	381837	30.623	33.501	N/A	14011	2.627	1.163	
3	Unknown	10	16.937	4462474	171161	18.162	15.017	N/A	11131	2.452	1.452	
4	Unknown	10	18.480	7561813	322495	30.776	28.294	N/A	14208	5.772	1.165	
5	Unknown	10	22.413	736229	26261	2.996	2.304	N/A	14432	N/A	1.097	

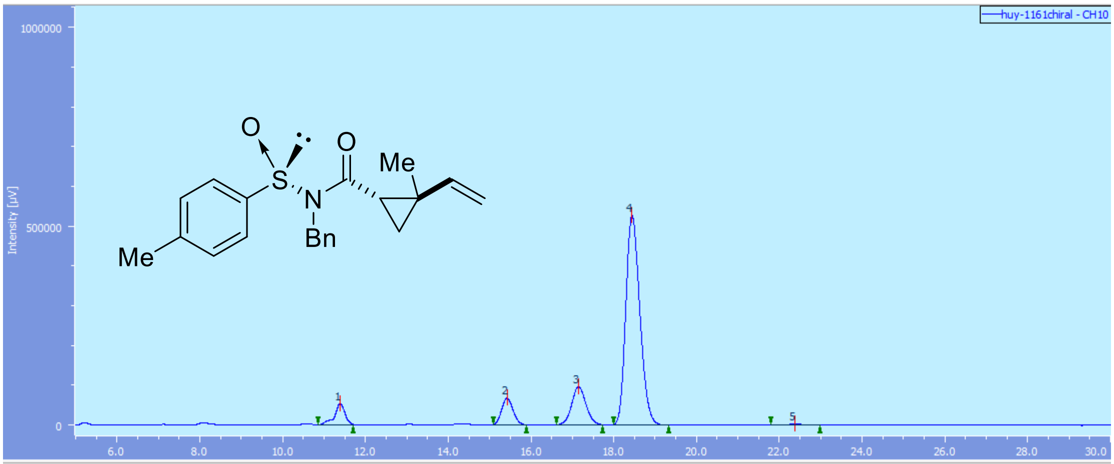

#	Peak Name	CH	tR	Area	Height	Area%	Height%	Quantity	NTP	Resolution	Symmetry Factor	Warning
1	Unknown	10	11.390	919245	52871	5.423	7.115	N/A	12868	8.858	0.793	
2	Unknown	10	15.413	1269831	66579	7.492	8.960	N/A	14658	3.064	1.111	
3	Unknown	10	17.137	2285506	96236	13.484	12.951	N/A	12281	2.084	1.053	
4	Unknown	10	18.430	12400912	524638	73.164	70.605	N/A	13880	5.848	1.212	
5	Unknown	10	22.370	73934	2731	0.436	0.368	N/A	15222	N/A	0.998	

(*S*,*E*)-*N*-Benzyl-2-(*p*-tolyl)-6-tosylhex-4-enamide (2.1)

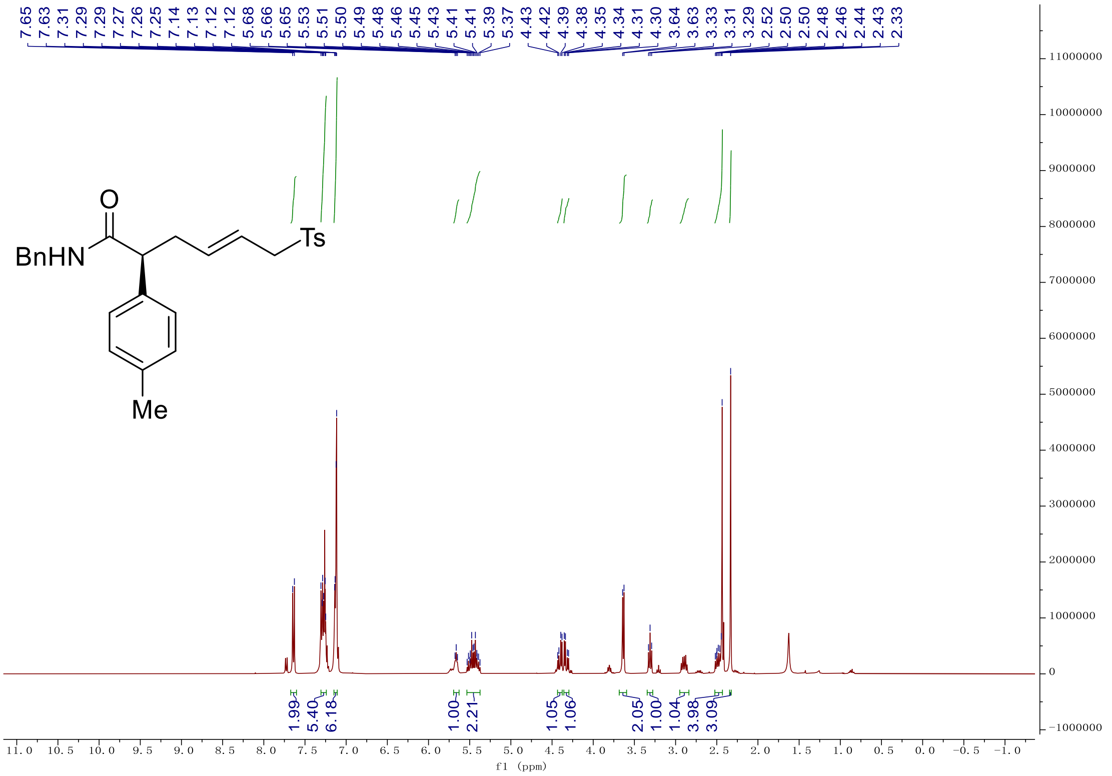

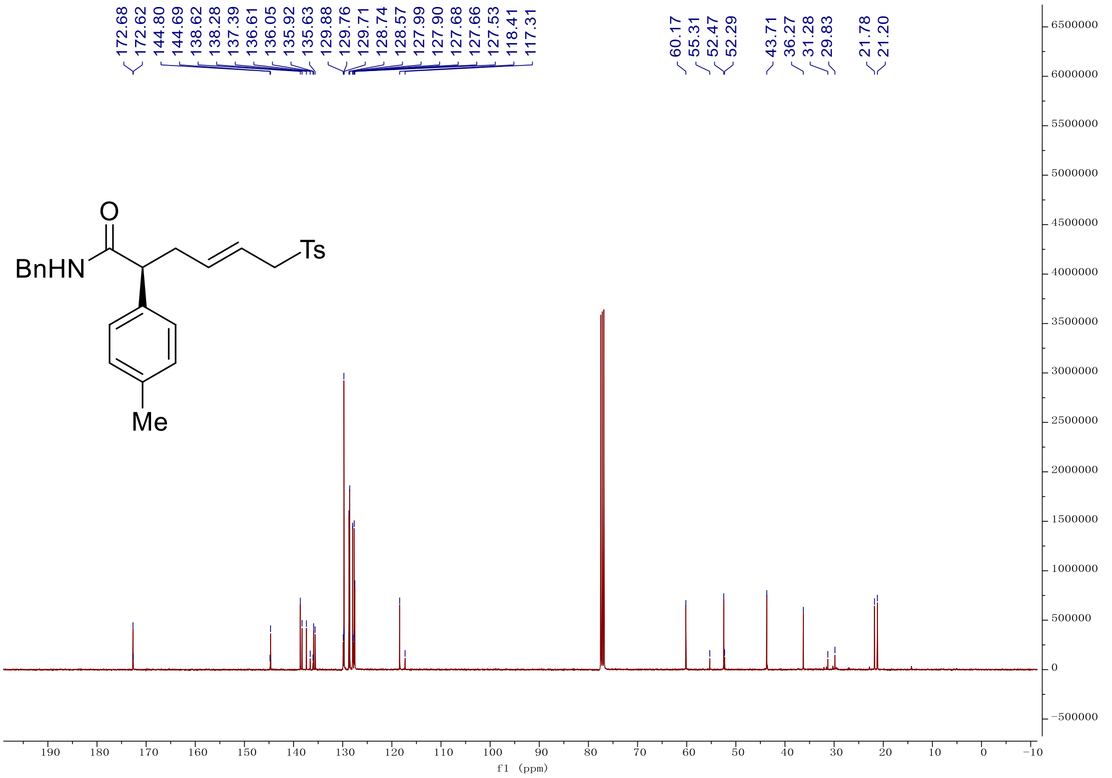

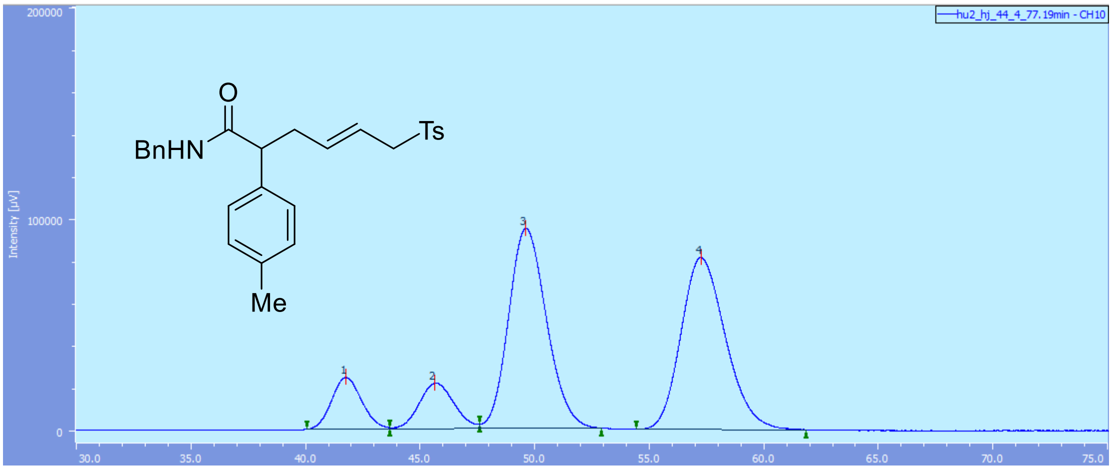

#	Peak Name	CH	tR	Area	Height	Area%	Height%	Quantity	NTP	Resolution	Symmetry Factor	Warning
1	Unknown	10	41.753	2222159	24430	8.430	11.017	N/A	4770	1.492	1.114	
2	Unknown	10	45.637	2277233	21561	8.639	9.723	N/A	4245	1.360	N/A	
3	Unknown	10	49.597	10899298	94557	41.347	42.641	N/A	4264	2.325	1.159	
4	Unknown	10	57.223	10962090	81204	41.585	36.619	N/A	4178	N/A	1.212	

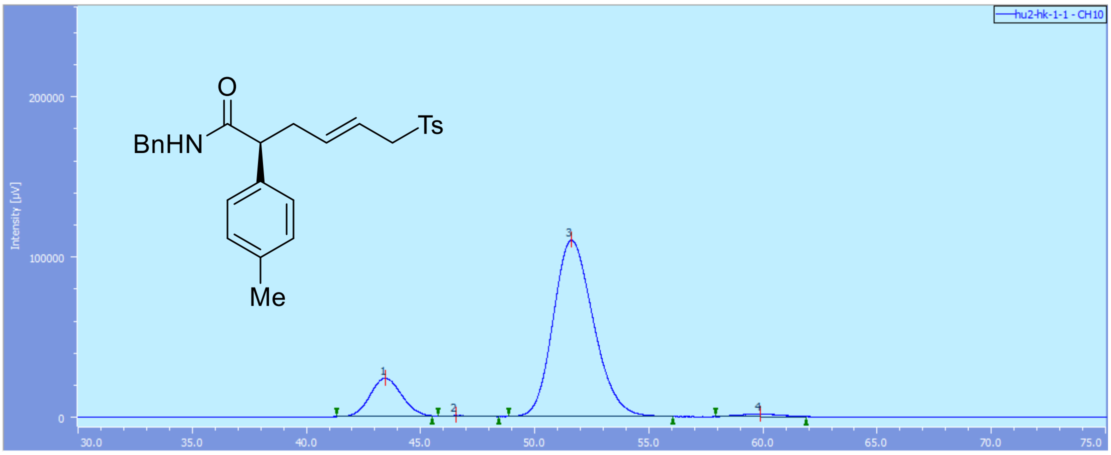

#	Peak Name	CH	tR	Area	Height	Area%	Height%	Quantity	NTP	Resolution	Symmetry Factor	Warning
1	Unknown	10	43.463	2254317	23527	14.194	17.378	N/A	4625	1.227	1.053	
2	Unknown	10	46.527	29022	488	0.183	0.360	N/A	5770	1.792	1.646	
3	Unknown	10	51.583	13399222	109652	84.364	80.994	N/A	4136	2.477	1.196	
4	Unknown	10	59.863	200017	1715	1.259	1.267	N/A	4695	N/A	1.045	

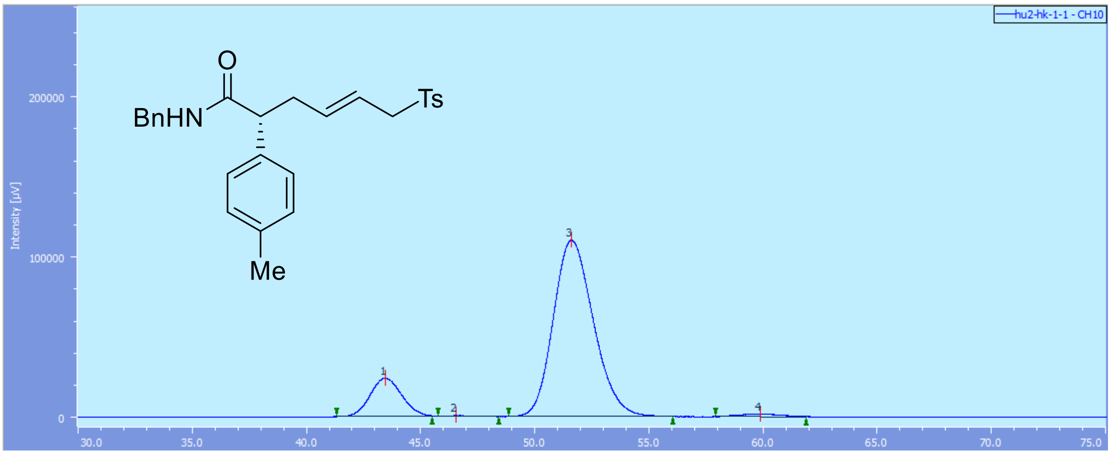

#	Peak Name	CH	tR	Area	Height	Area%	Height%	Quantity	NTP	Resolution	Symmetry Factor	Warning
1	Unknown	10	43.463	2254317	23527	14.194	17.378	N/A	4625	1.227	1.053	
2	Unknown	10	46.527	29022	488	0.183	0.360	N/A	5770	1.792	1.646	
3	Unknown	10	51.583	13399222	109652	84.364	80.994	N/A	4136	2.477	1.196	
4	Unknown	10	59.863	200017	1715	1.259	1.267	N/A	4695	N/A	1.045	

(*S*,*E*)-*N*-Benzyl-2-(4-methoxyphenyl)-6-tosylhex-4-enamide (2.2)

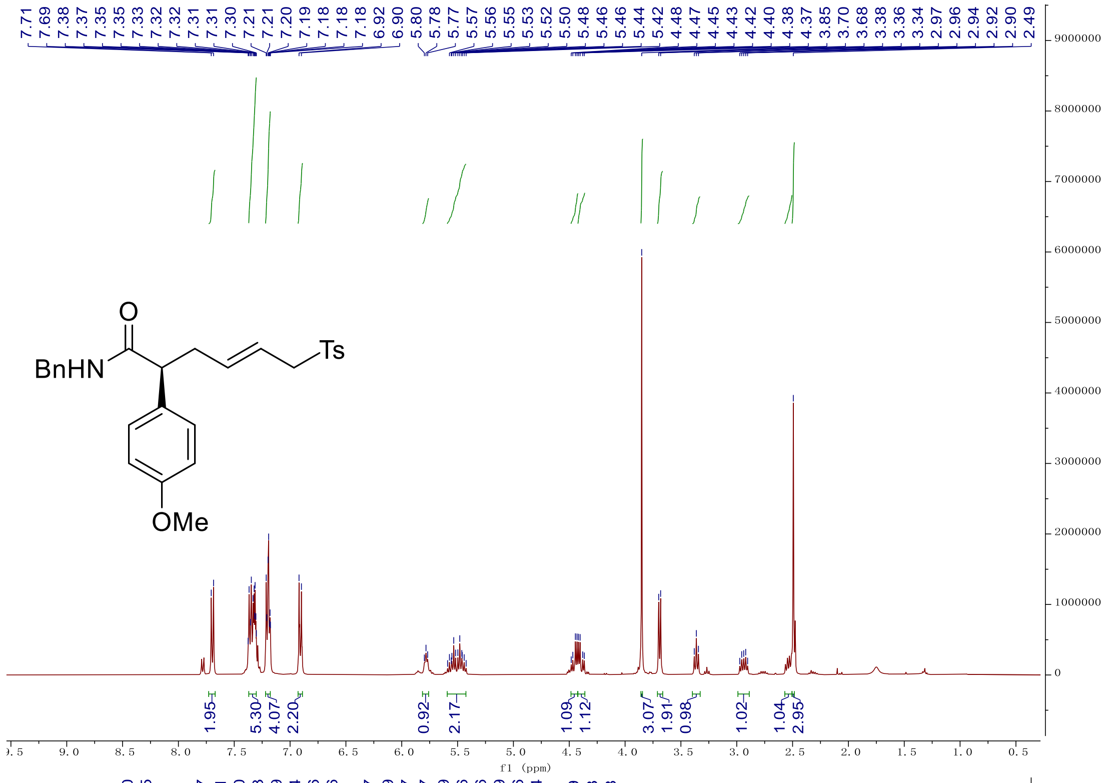

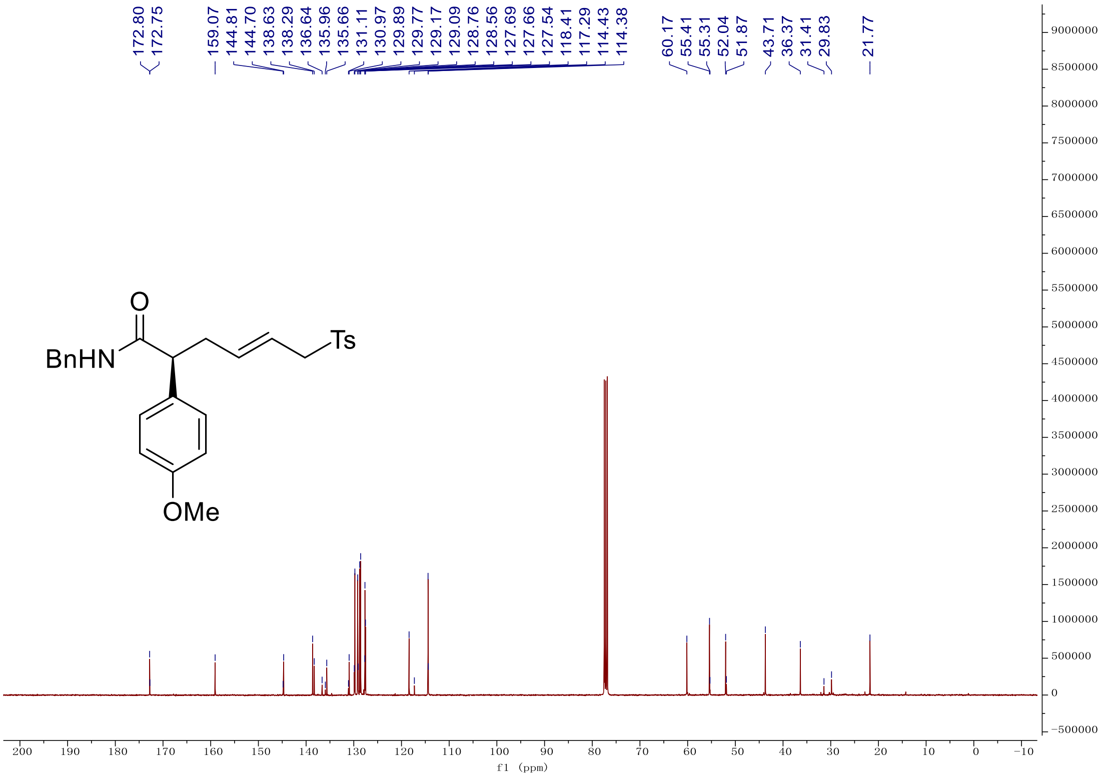

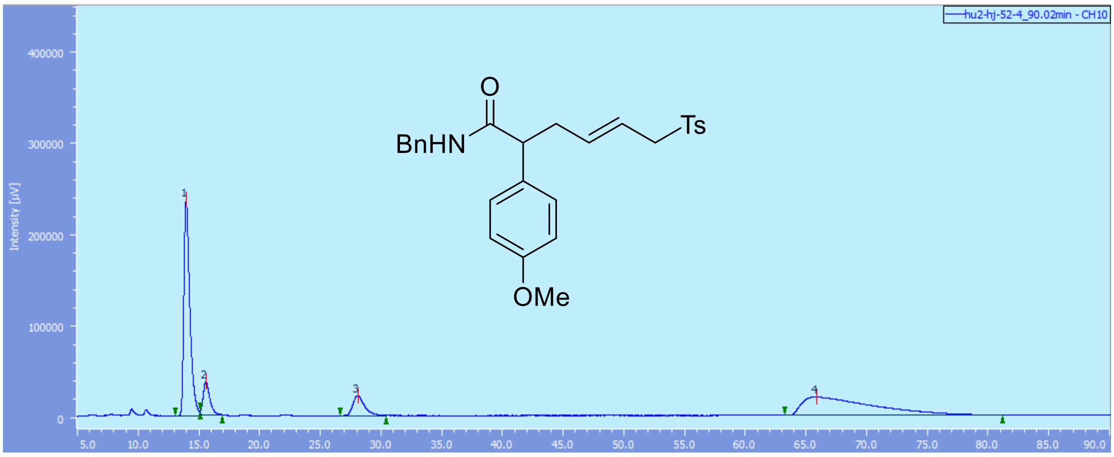

#	Peak Name	CH	tR	Area	Height	Area%	Height%	Quantity	NTP	Resolution	Symmetry Factor	Warning
1	Unknown	10	13.947	8048771	235012	42.469	75.081	N/A	4176	1.799	1.614	
2	Unknown	10	15.577	1369813	36507	7.228	11.663	N/A	4268	9.503	N/A	
3	Unknown	10	28.063	1434366	21510	7.568	6.872	N/A	4458	6.133	1.472	
4	Unknown	10	65.793	8098979	19982	42.734	6.384	N/A	610	N/A	3.629	

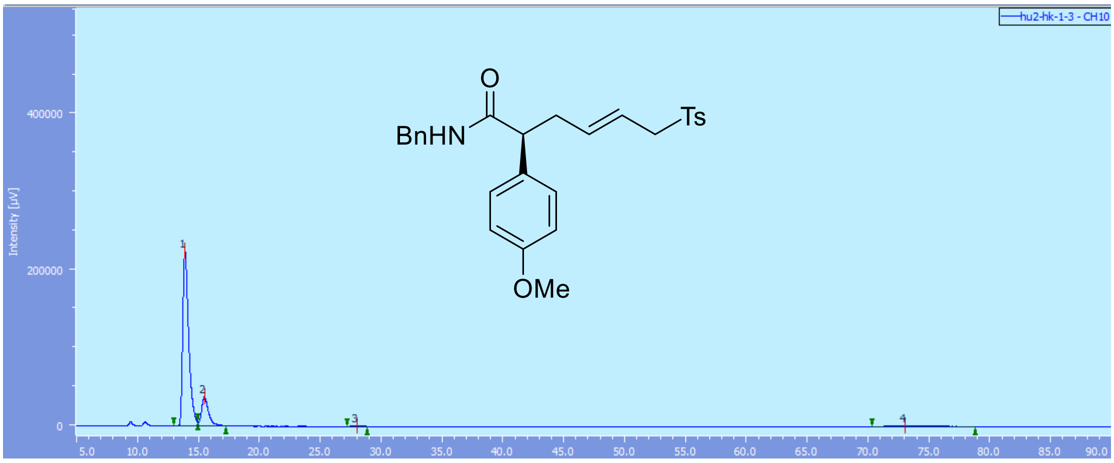

#	Peak Name	CH	tR	Area	Height	Area%	Height%	Quantity	NTP	Resolution	Symmetry Factor	Warning
1	Unknown	10	13.900	7649137	223937	79.690	84.799	N/A	4171	1.754	1.613	
2	Unknown	10	15.493	1477910	37217	15.397	14.093	N/A	4164	10.941	N/A	
3	Unknown	10	28.000	52872	1153	0.551	0.437	N/A	7072	11.467	0.997	
4	Unknown	10	73.000	418651	1772	4.362	0.671	N/A	1995	N/A	1.553	

(*S*,*E*)-*N*-Benzyl-2-(4-bromophenyl)-6-tosylhex-4-enamide (2.3)

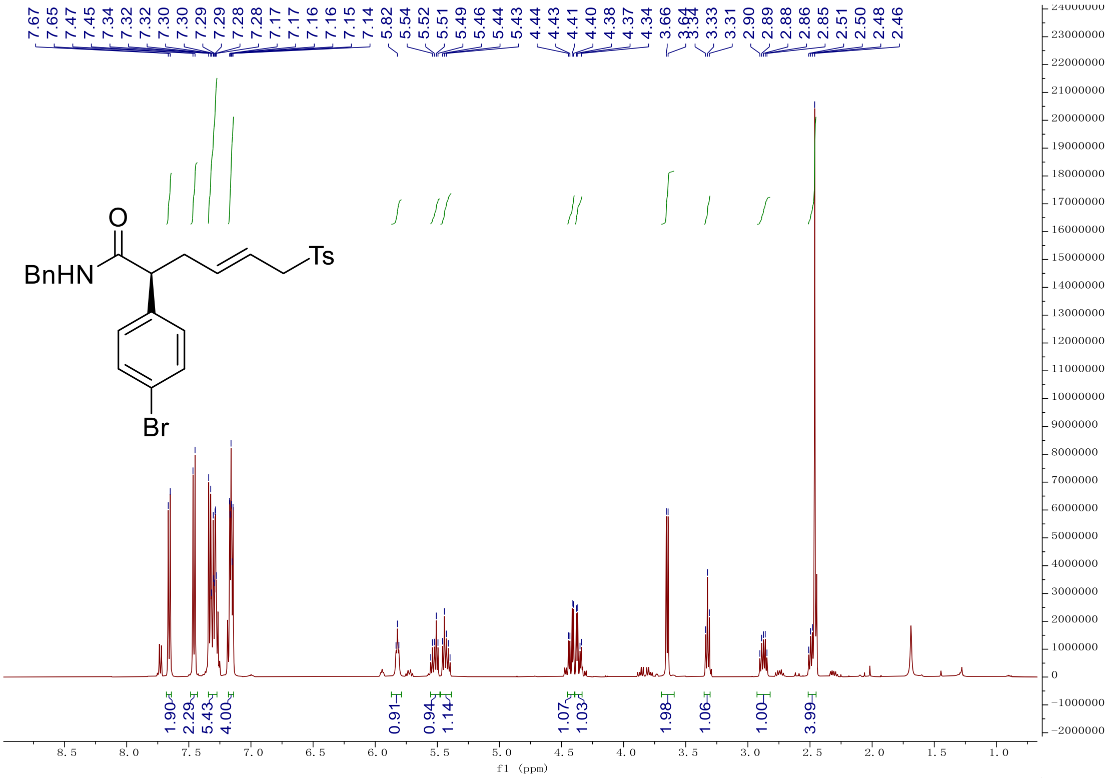

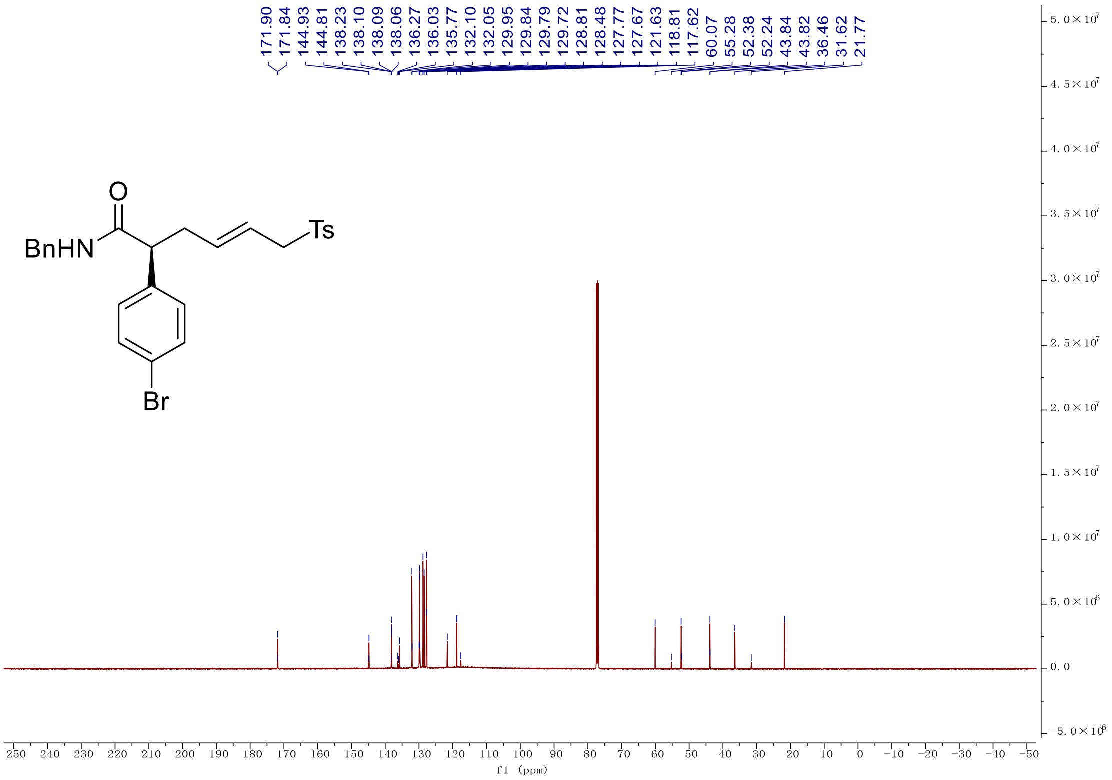

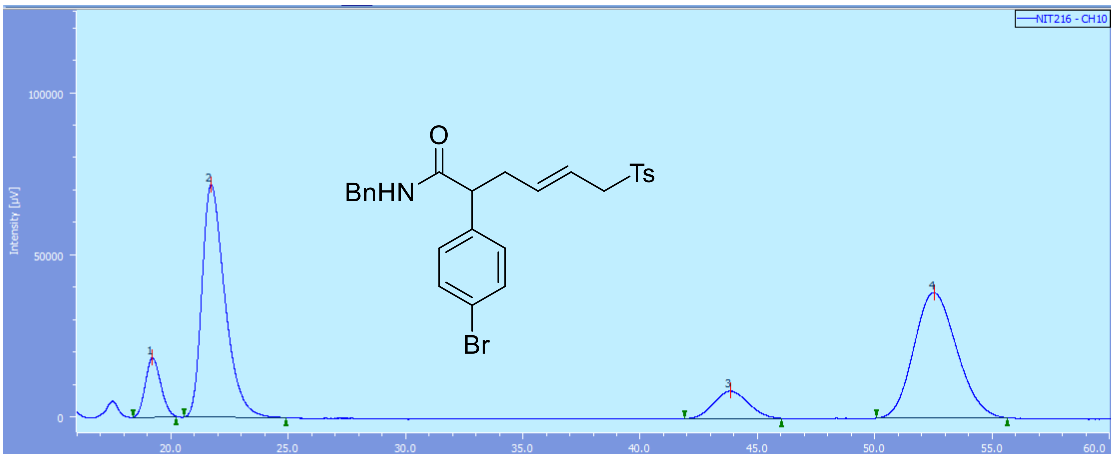

#	Peak Name	CH	tR	Area	Height	Area%	Height%	Quantity	NTP	Resolution	Symmetry Factor	Warning
1	Unknown	10	19.197	826097	18106	7.235	13.254	N/A	3954	1.698	1.151	
2	Unknown	10	21.703	4856476	71571	42.533	52.393	N/A	2493	9.890	1.449	
3	Unknown	10	43.830	857393	8412	7.509	6.158	N/A	4071	2.834	1.077	
4	Unknown	10	52.490	4878266	38516	42.723	28.195	N/A	3856	N/A	1.136	

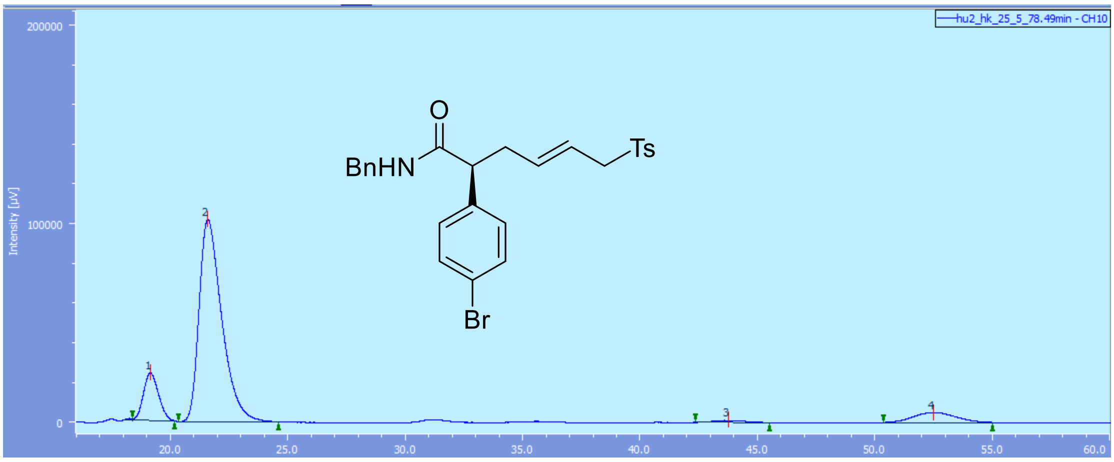

#	Peak Name	CH	tR	Area	Height	Area%	Height%	Quantity	NTP	Resolution	Symmetry Factor	Warning
1	Unknown	10	19.147	1077808	23944	12.524	18.184	N/A	4076	1.681	1.128	
2	Unknown	10	21.590	6814265	101708	79.180	77.240	N/A	2536	10.646	1.506	
3	Unknown	10	43.743	89751	1025	1.043	0.779	N/A	5067	3.004	1.092	
4	Unknown	10	52.460	624228	5001	7.253	3.798	N/A	3897	N/A	1.090	

(*S*,*E*)-*N*-Benzyl-2-(4-fluorophenyl)-6-tosylhex-4-enamide (2.4)

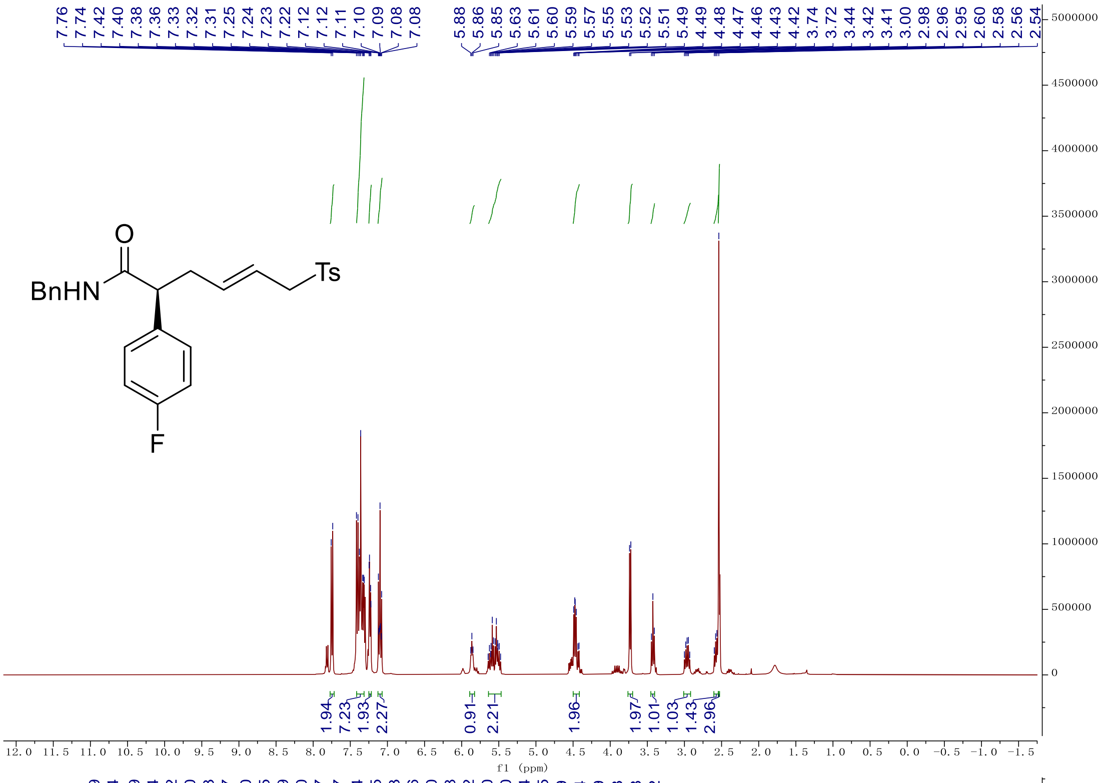

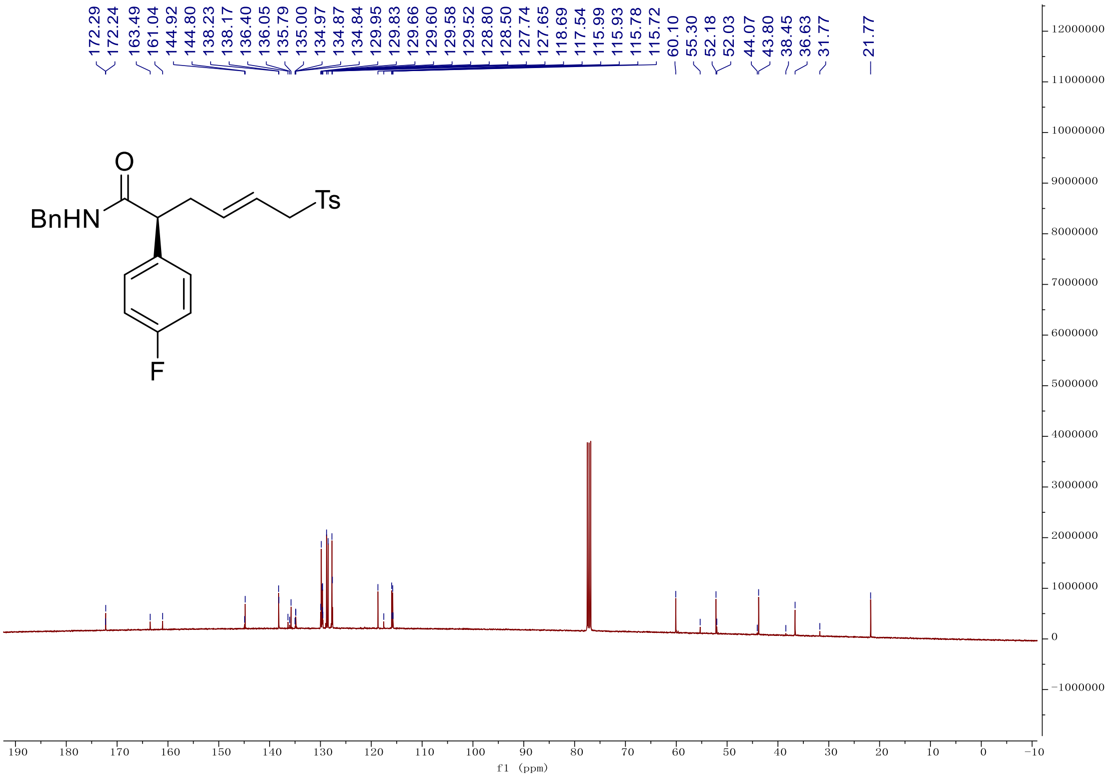

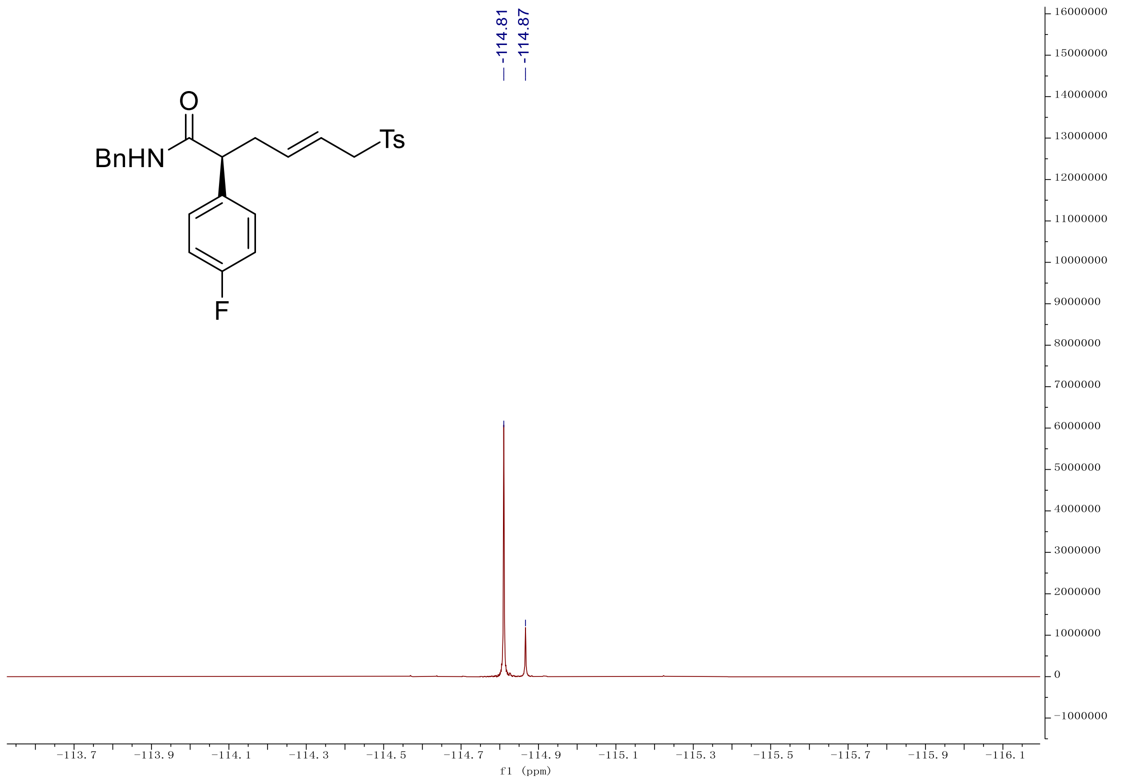
-114.81
-114.87
BnHN
O
Ts
F
f1 (ppm)

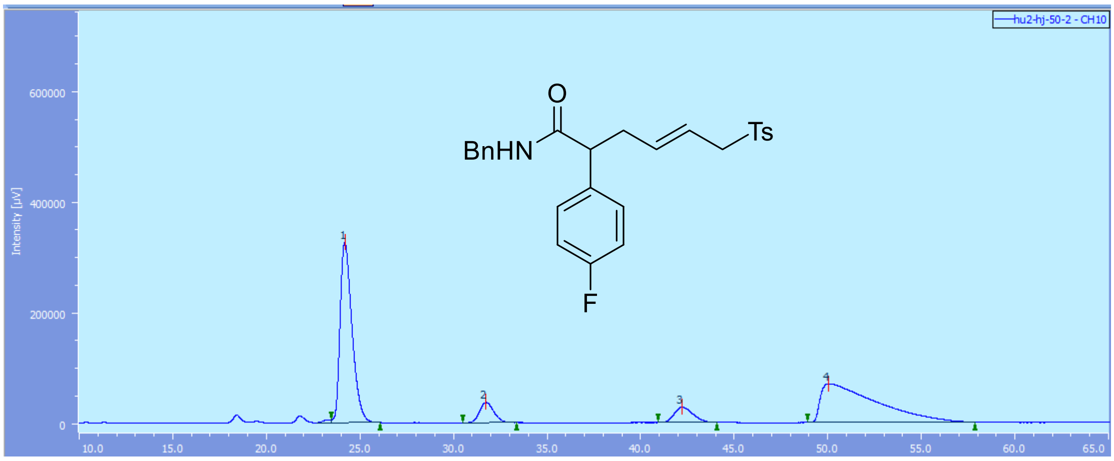

#	Peak Name	CH	tR	Area	Height	Area%	Height%	Quantity	NTP	Resolution	Symmetry Factor	Warning
1	Unknown	10	24.177	14418430	325424	44.097	70.922	N/A	7044	5.940	1.482	
2	Unknown	10	31.717	1931018	36485	5.906	7.952	N/A	8293	6.483	1.185	
3	Unknown	10	42.183	1905109	27534	5.827	6.001	N/A	8368	2.155	1.279	
4	Unknown	10	50.017	14442410	69403	44.170	15.126	N/A	1351	N/A	4.793	

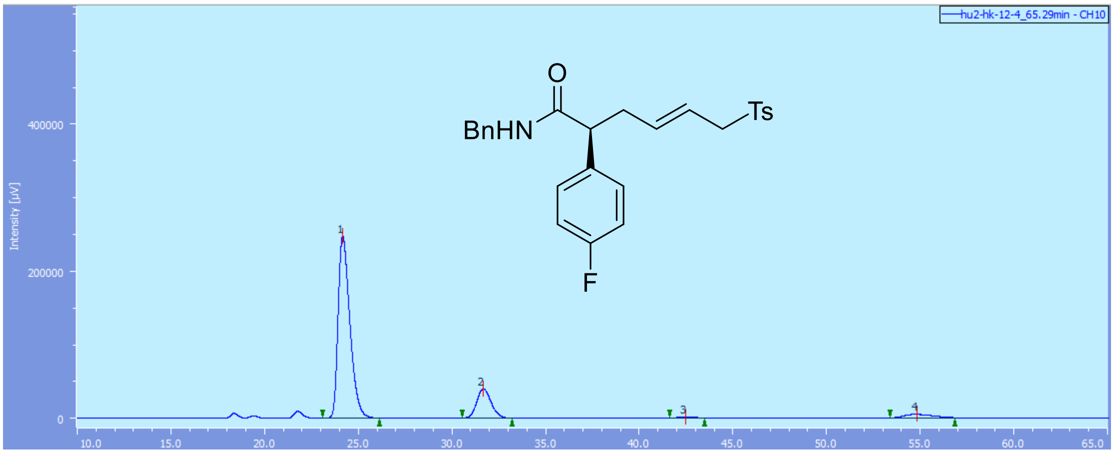

#	Peak Name	CH	tR	Area	Height	Area%	Height%	Quantity	NTP	Resolution	Symmetry Factor	Warning
1	Unknown	10	24.157	10787062	246763	80.433	84.419	N/A	7219	5.932	1.484	
2	Unknown	10	31.657	2088263	39487	15.571	13.509	N/A	8204	7.362	1.166	
3	Unknown	10	42.470	72932	1373	0.544	0.470	N/A	12051	5.747	1.067	
4	Unknown	10	54.767	462930	4683	3.452	1.602	N/A	6376	N/A	1.314	

(*S*,*E*)-*N*-Benzyl-2-(*o*-tolyl)-6-tosylhex-4-enamide (2.5)

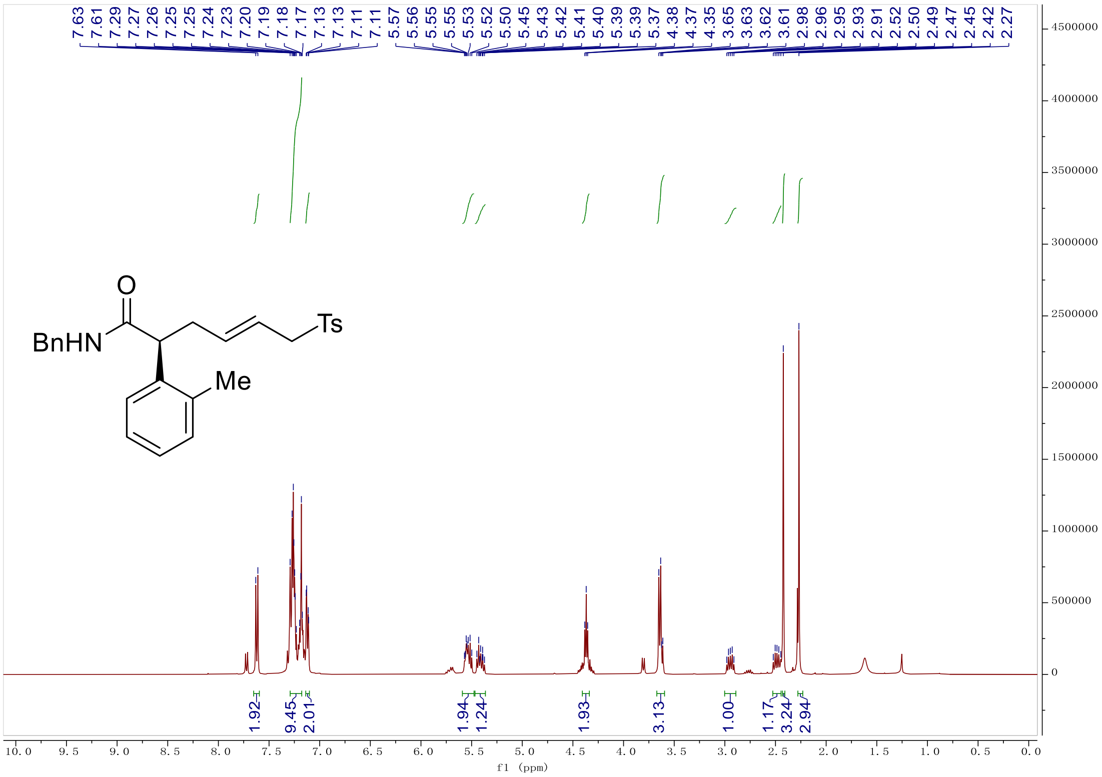

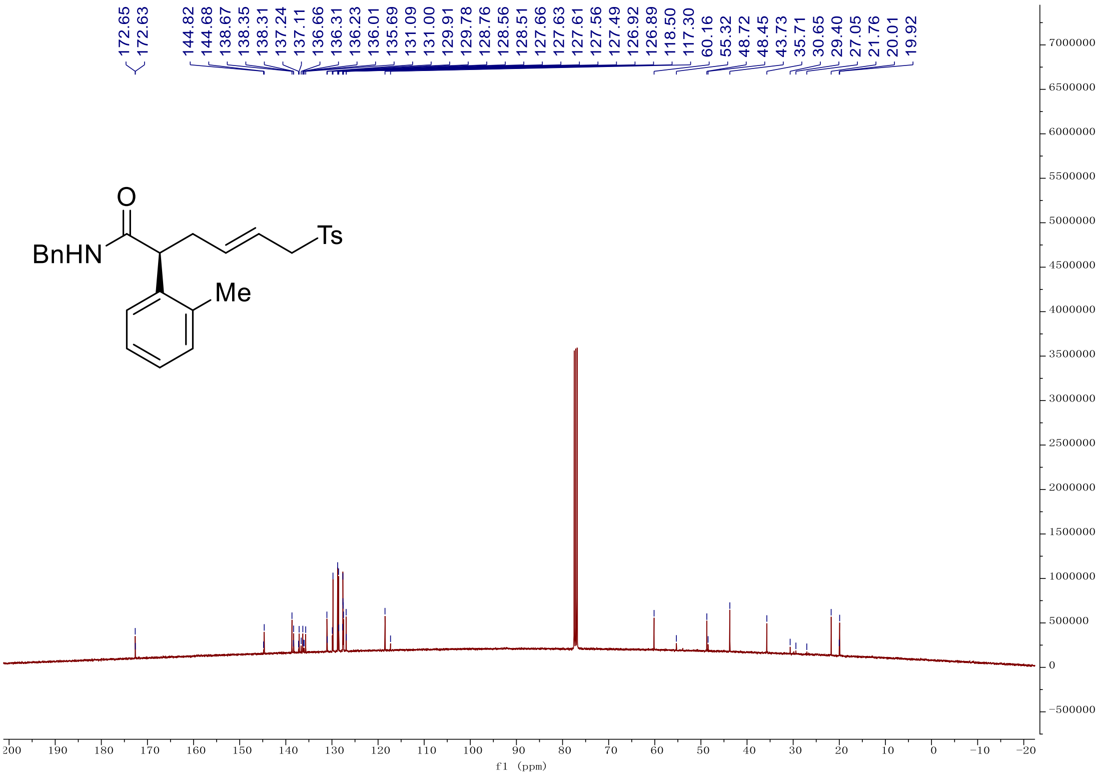

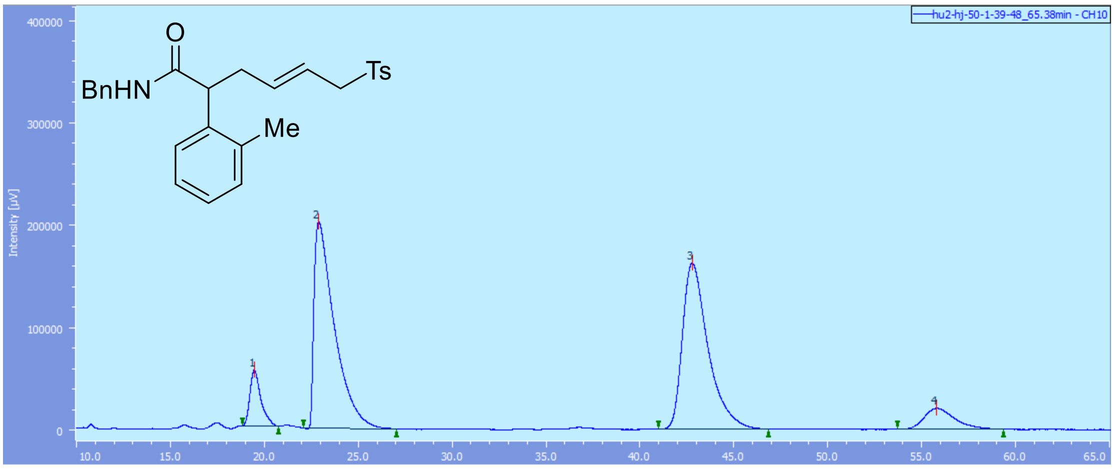

#	Peak Name	CH	tR	Area	Height	Area%	Height%	Quantity	NTP	Resolution	Symmetry Factor	Warning
1	Unknown	10	19.457	2223248	54466	6.350	12.443	N/A	5936	2.378	1.508	
2	Unknown	10	22.890	15166280	201283	43.318	45.982	N/A	2360	9.366	3.194	
3	Unknown	10	42.753	15287417	161563	43.664	36.908	N/A	5214	4.942	1.609	
4	Unknown	10	55.747	2334563	20430	6.668	4.667	N/A	5894	N/A	1.422	

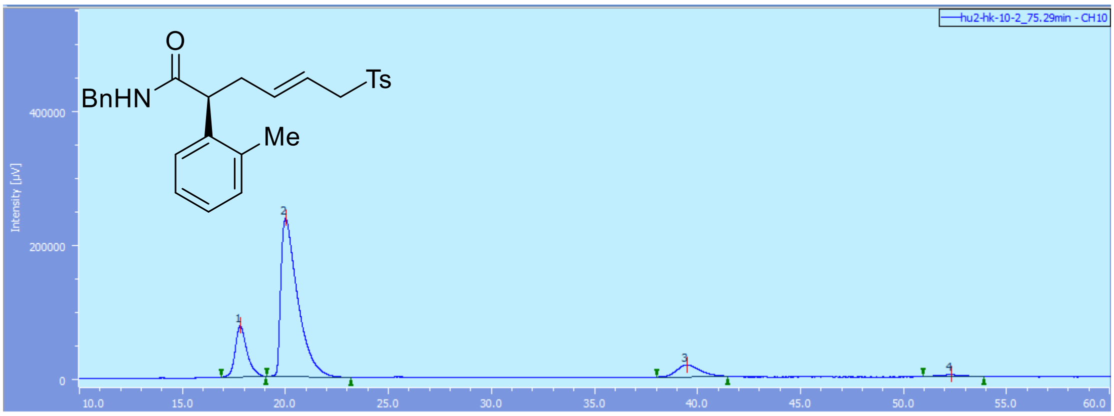

#	Peak Name	CH	tR	Area	Height	Area%	Height%	Quantity	NTP	Resolution	Symmetry Factor	Warning
1	Unknown	10	17.800	3051798	76364	16.833	22.979	N/A	5148	1.818	1.318	
2	Unknown	10	20.000	13430956	235762	74.081	70.943	N/A	3110	11.141	2.467	
3	Unknown	10	39.453	1403153	17446	7.739	5.250	N/A	5829	5.740	1.291	
4	Unknown	10	52.307	244099	2753	1.346	0.828	N/A	7454	N/A	1.022	

(*S*,*E*)-*N*-Benzyl-2-(2-bromo-tolyl)-6-tosylhex-4-enamide (2.6)

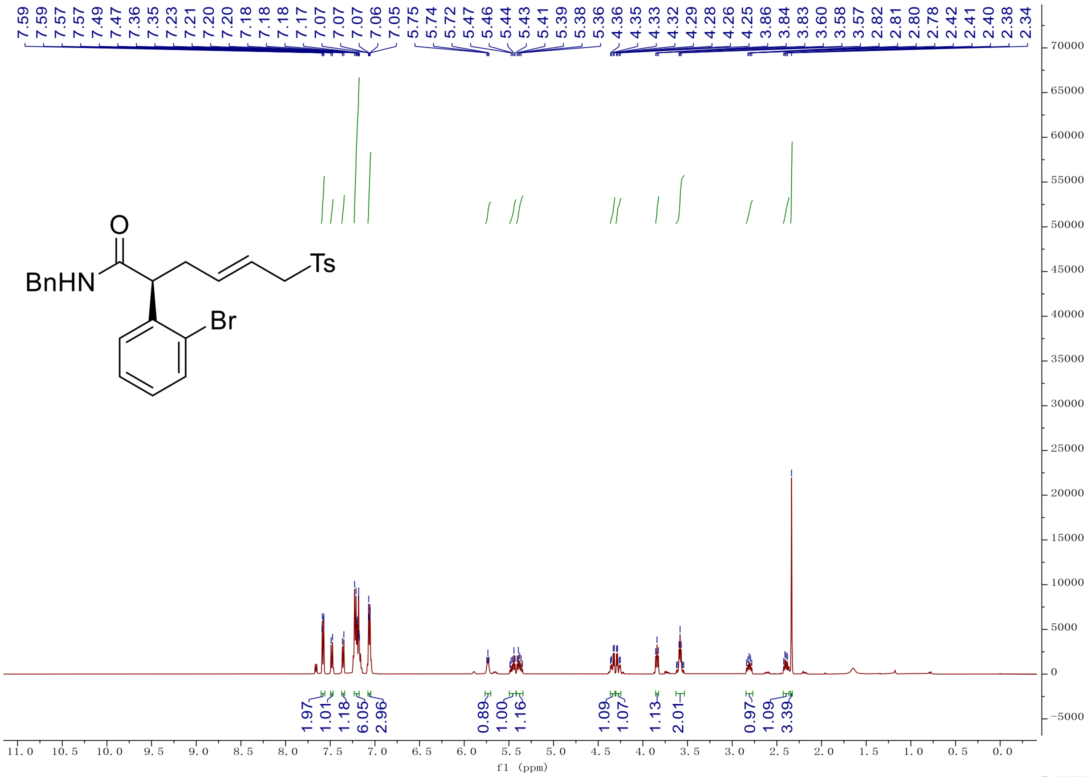

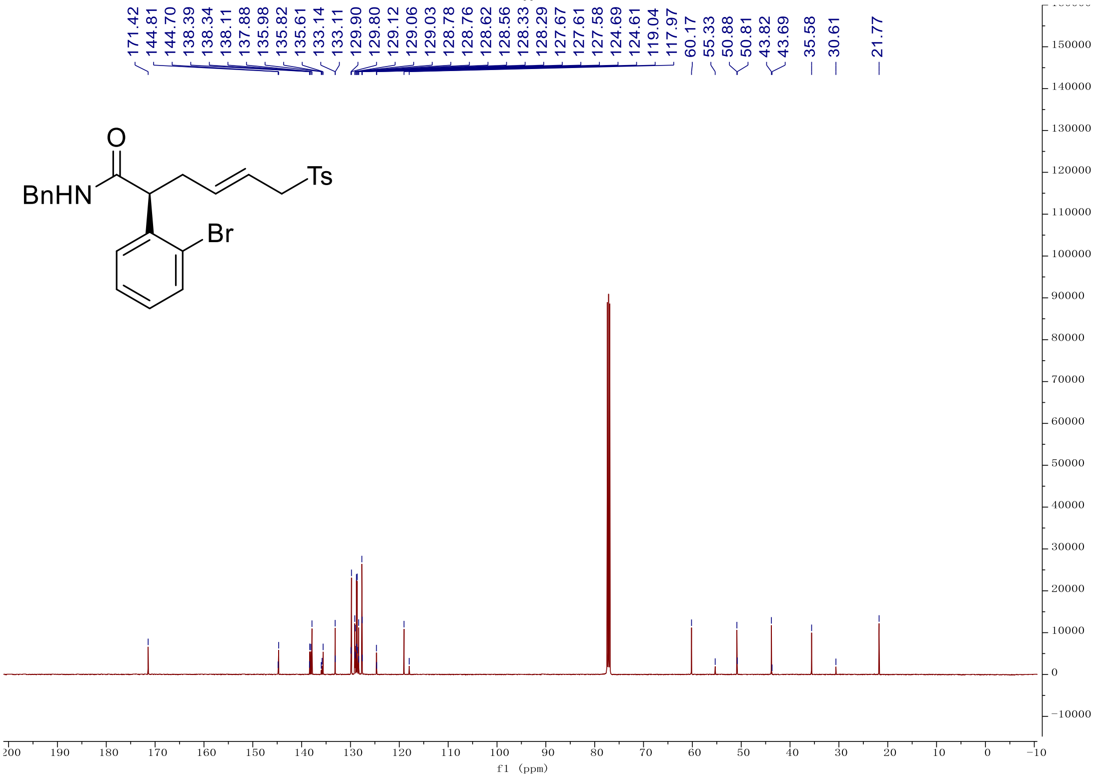

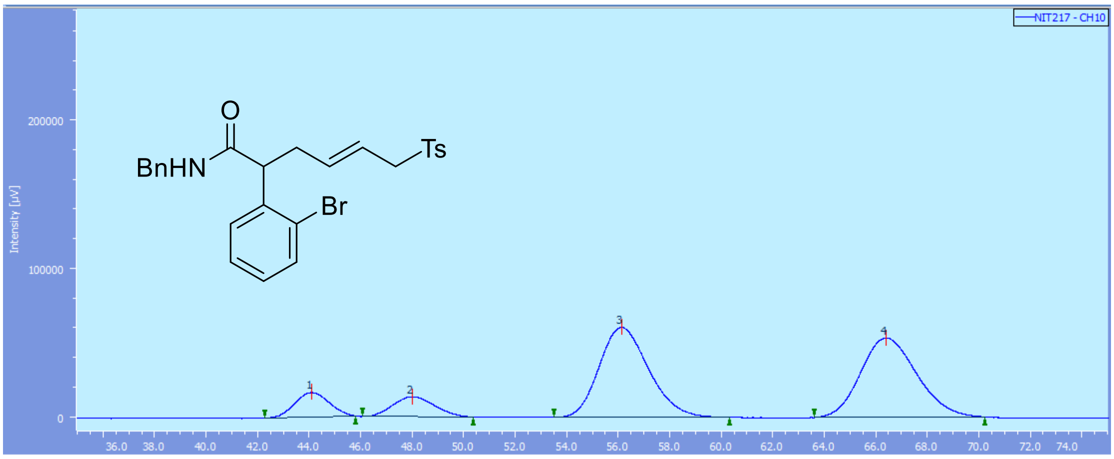

#	Peak Name	CH	tR	Area	Height	Area%	Height%	Quantity	NTP	Resolution	Symmetry Factor	Warning
1	Unknown	10	44.087	1540298	16585	8.024	11.545	N/A	4938	1.408	1.027	
2	Unknown	10	48.017	1513263	13394	7.883	9.324	N/A	3871	2.461	1.130	
3	Unknown	10	56.123	8083316	60399	42.108	42.045	N/A	4071	2.733	1.203	
4	Unknown	10	66.387	8059870	53276	41.986	37.086	N/A	4380	N/A	1.161	

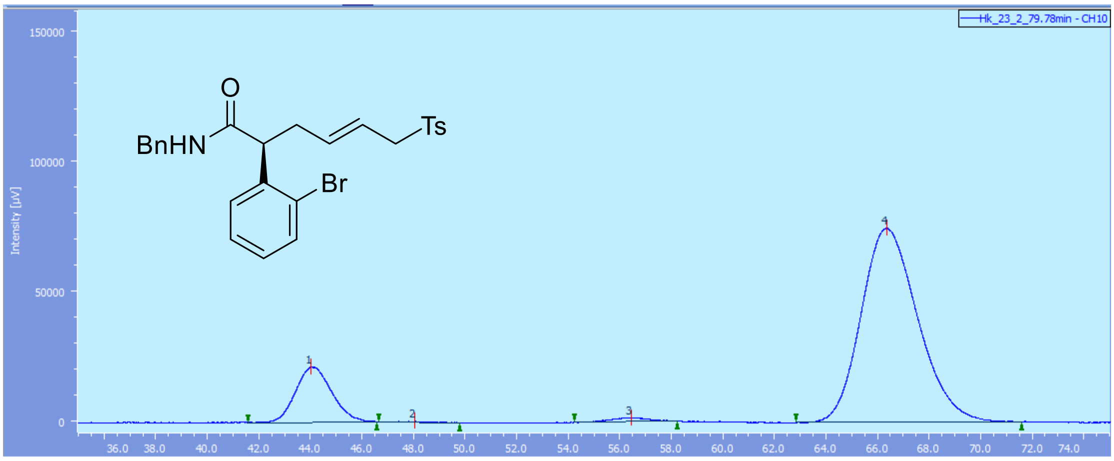

#	Peak Name	CH	tR	Area	Height	Area%	Height%	Quantity	NTP	Resolution	Symmetry Factor	Warning
1	Unknown	10	44.027	2041201	21087	15.065	21.688	N/A	4693	1.439	1.167	
2	Unknown	10	48.047	13119	294	0.097	0.302	N/A	4018	2.810	1.261	
3	Unknown	10	56.417	148482	1454	1.096	1.495	N/A	5884	2.864	0.966	
4	Unknown	10	66.340	11346649	74394	83.743	76.515	N/A	4389	N/A	1.193	

(*S*,*E*)-*N*-Benzyl-2-(naphthalen-1-yl)-6-tosylhex-4-enamide (*E*-2.7)

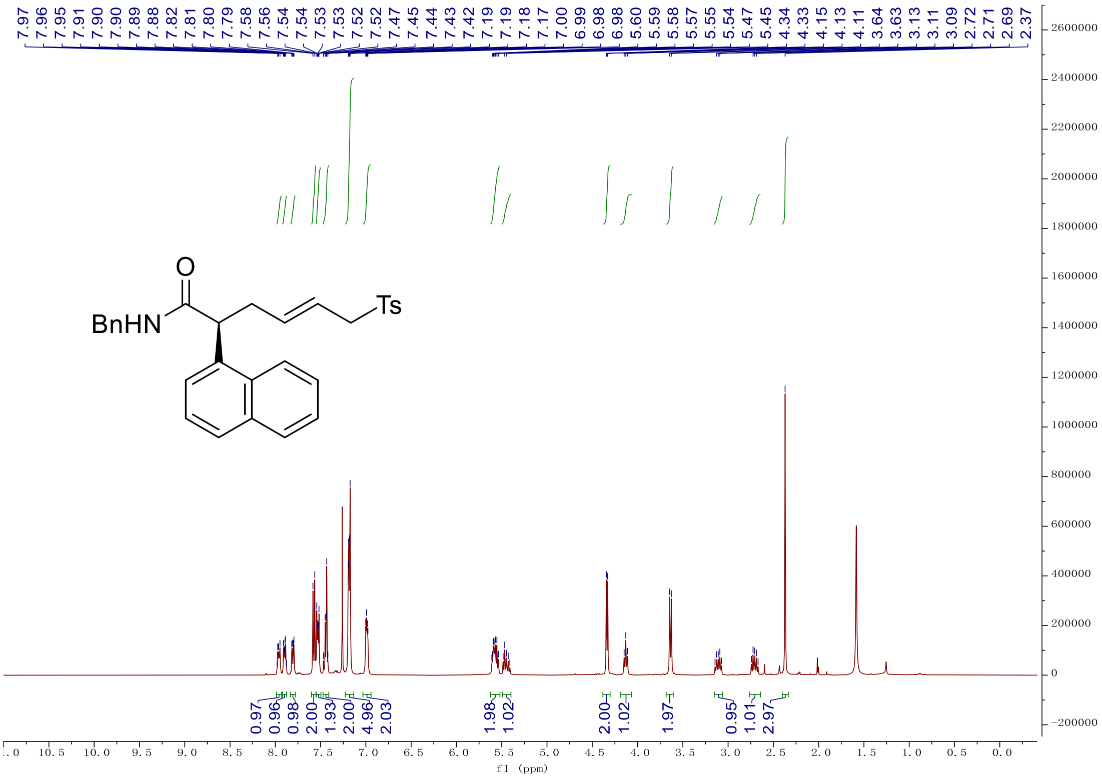

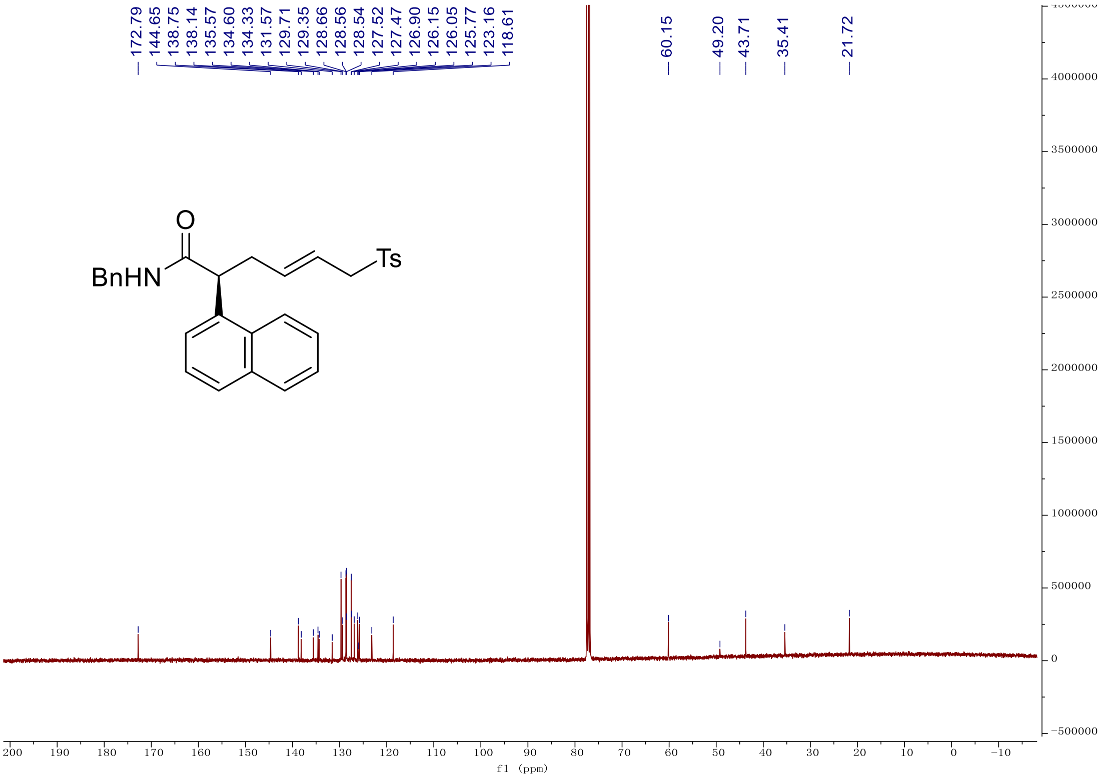

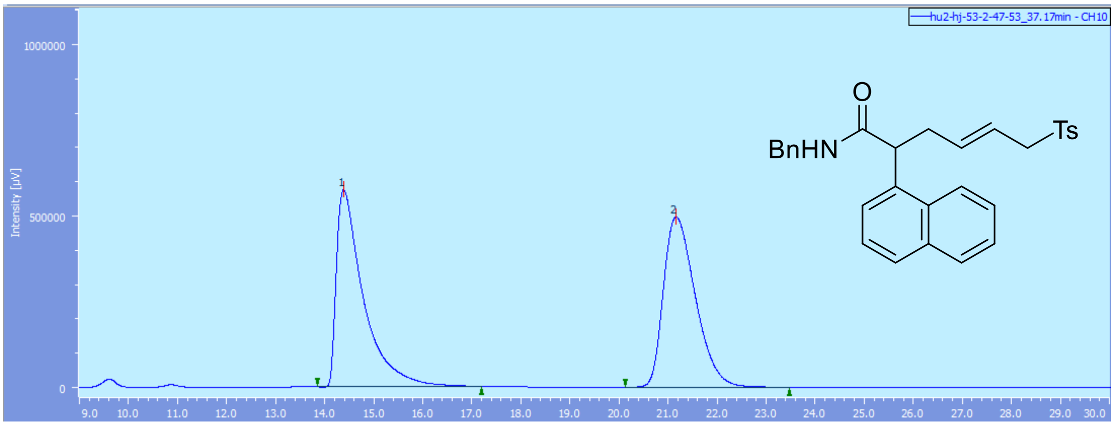

#	Peak Name	CH	tR	Area	Height	Area%	Height%	Quantity	NTP	Resolution	Symmetry Factor	Warning
1	Unknown	10	13.553	10439997	399445	50.823	63.736	N/A	6128	9.088	1.136	
2	Unknown	10	22.060	10101985	227273	49.177	36.264	N/A	5549	N/A	1.190	

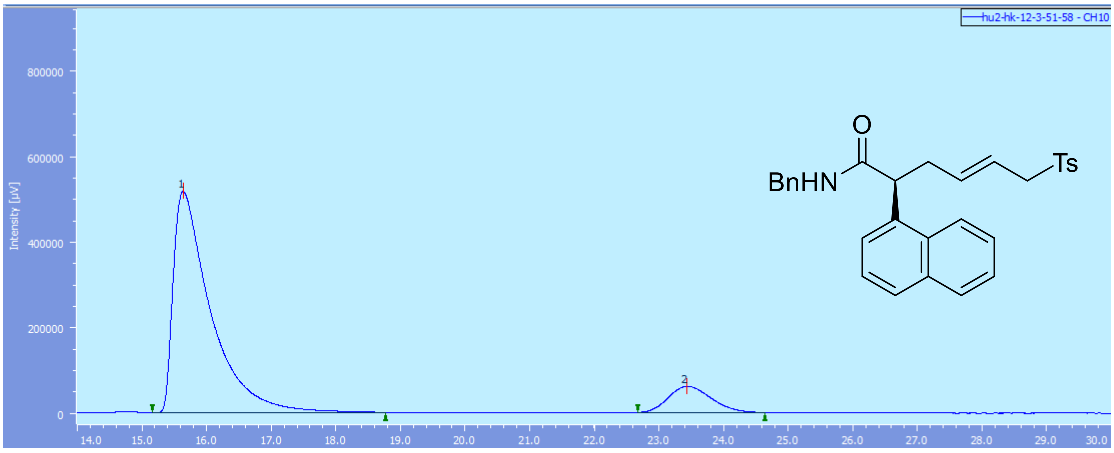

#	Peak Name	CH	tR	Area	Height	Area%	Height%	Quantity	NTP	Resolution	Symmetry Factor	Warning
1	Unknown	10	15.637	20850241	516705	88.122	89.428	N/A	4293	7.170	2.797	
2	Unknown	10	23.427	2810323	61082	11.878	10.572	N/A	5859	N/A	1.223	

(*S*,*Z*)-*N*-Benzyl-2-(naphthalen-1-yl)-6-tosylhex-4-enamide (*Z*-2.7)

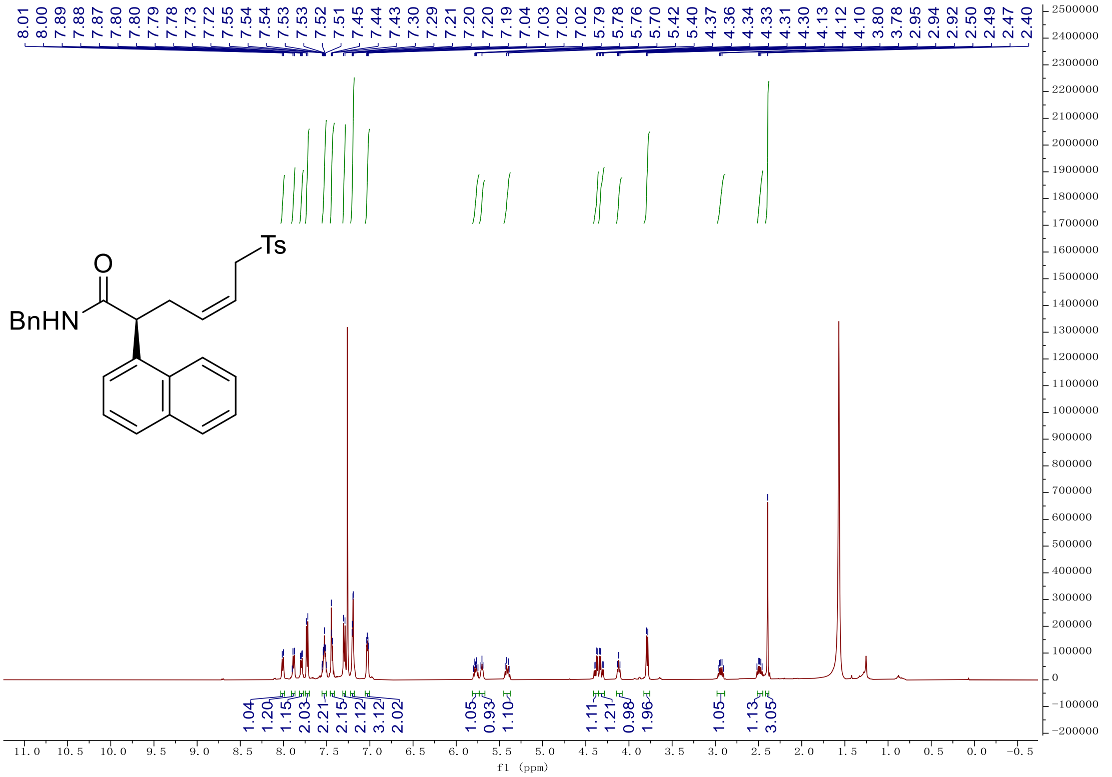

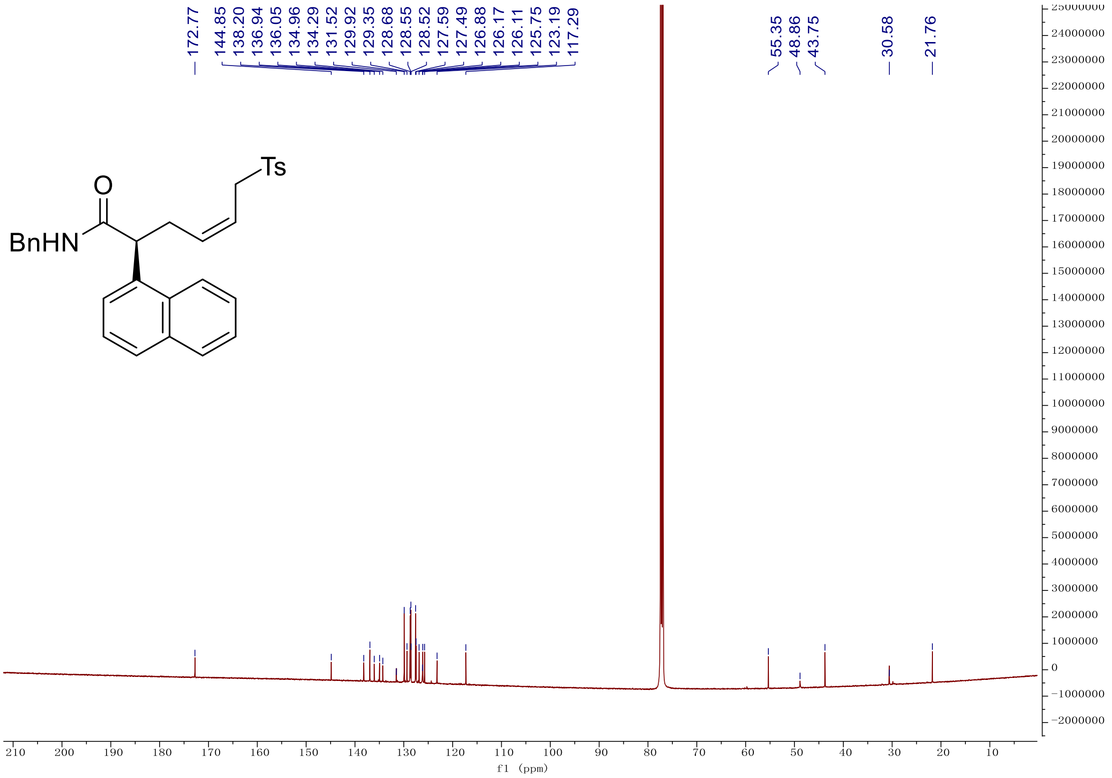

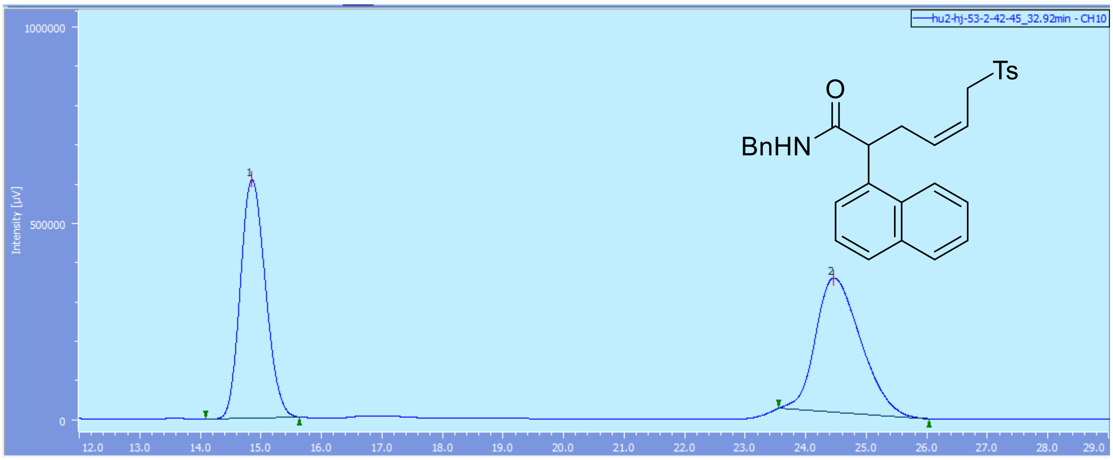

#	Peak Name	CH	tR	Area	Height	Area%	Height%	Quantity	NTP	Resolution	Symmetry Factor	Warning
1	Unknown	10	14.850	17395634	605847	49.605	63.999	N/A	5998	9.010	1.157	
2	Unknown	10	24.453	17672650	340799	50.395	36.001	N/A	5094	N/A	1.252	

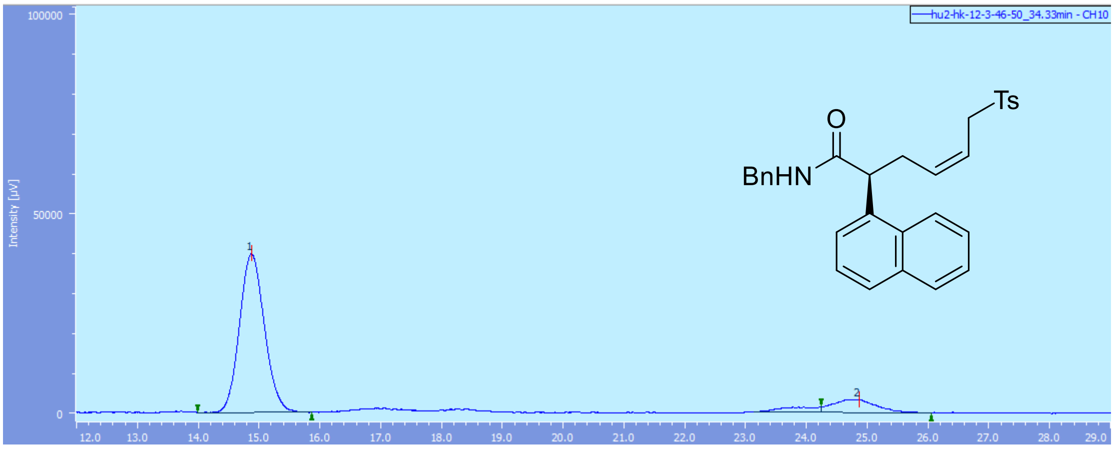

#	Peak Name	CH	tR	Area	Height	Area%	Height%	Quantity	NTP	Resolution	Symmetry Factor	Warning
1	Unknown	10	14.877	1129945	39650	87.841	92.509	N/A	6315	9.254	1.097	
2	Unknown	10	24.860	156402	3210	12.159	7.491	N/A	4941	N/A	N/A	

(*S*,*E*)-*N*-Benzyl-2-(naphthalen-2-yl)-6-tosylhex-4-enamide (2.8)

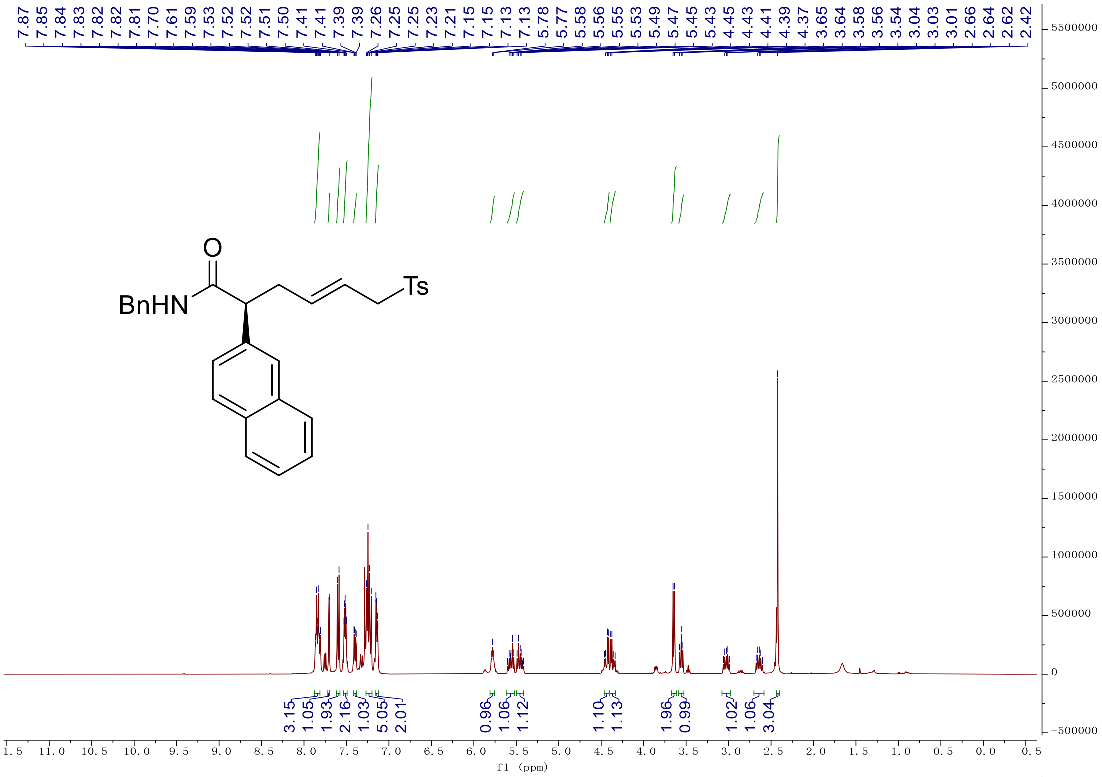

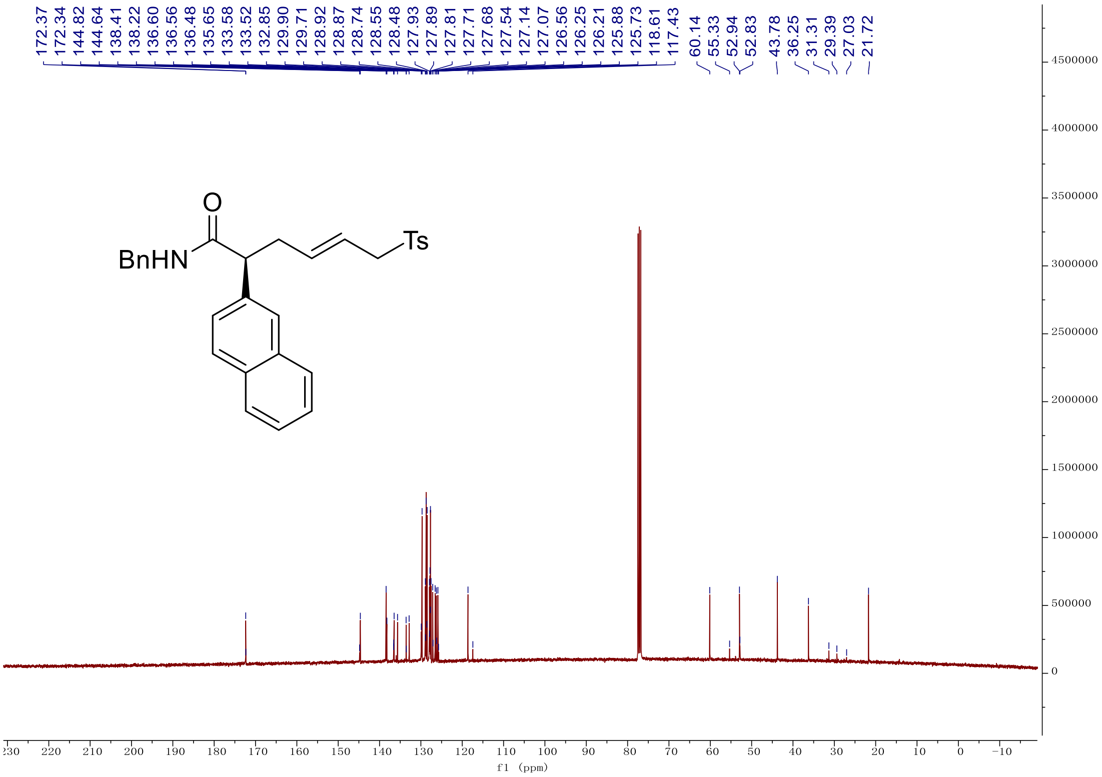

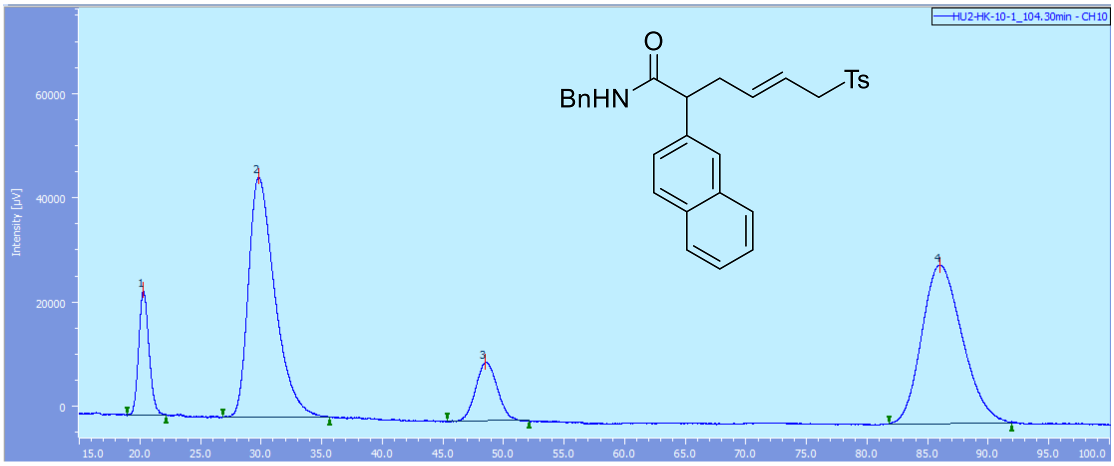

#	Peak Name	CH	tR	Area	Height	Area%	Height%	Quantity	NTP	Resolution	Symmetry Factor	Warning
1	Unknown	10	20.287	1401276	23789	8.346	21.363	N/A	2749	3.526	1.155	
2	Unknown	10	29.813	6966036	45958	41.492	41.272	N/A	949	5.228	1.488	
3	Unknown	10	48.473	1387628	11281	8.265	10.130	N/A	3480	7.971	1.132	
4	Unknown	10	85.983	7033941	30327	41.896	27.235	N/A	3127	N/A	1.185	

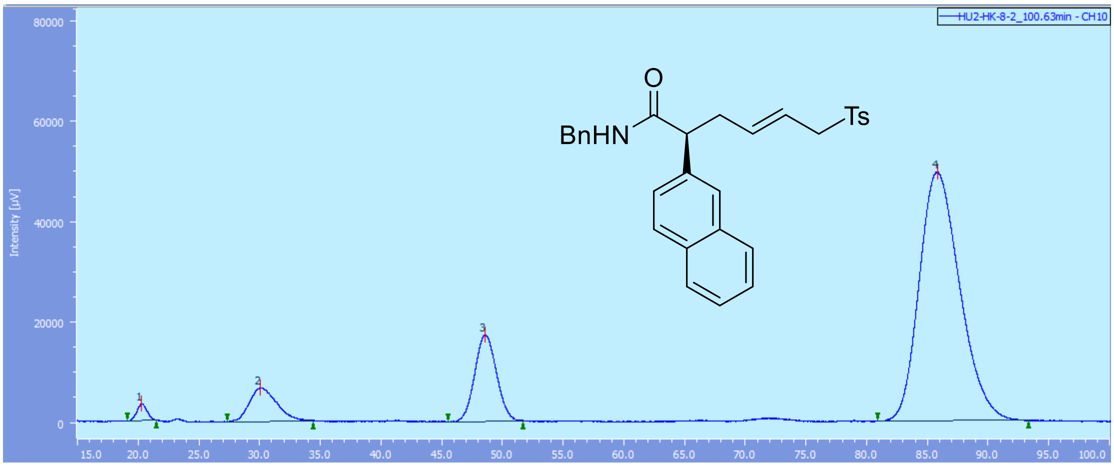

#	Peak Name	CH	tR	Area	Height	Area%	Height%	Quantity	NTP	Resolution	Symmetry Factor	Warning
1	Unknown	10	20.300	190821	3302	1.287	4.303	N/A	2675	3.434	1.103	
2	Unknown	10	30.083	1047228	6760	7.063	8.810	N/A	844	4.986	1.247	
3	Unknown	10	48.560	2128949	17191	14.359	22.403	N/A	3491	7.946	1.057	
4	Unknown	10	85.777	11459657	49482	77.291	64.484	N/A	3159	N/A	1.222	

(*R*,*E*)-*N*-Benzyl-2-(thiophen-2-yl)-6-tosylhex-4-enamide (2.9)

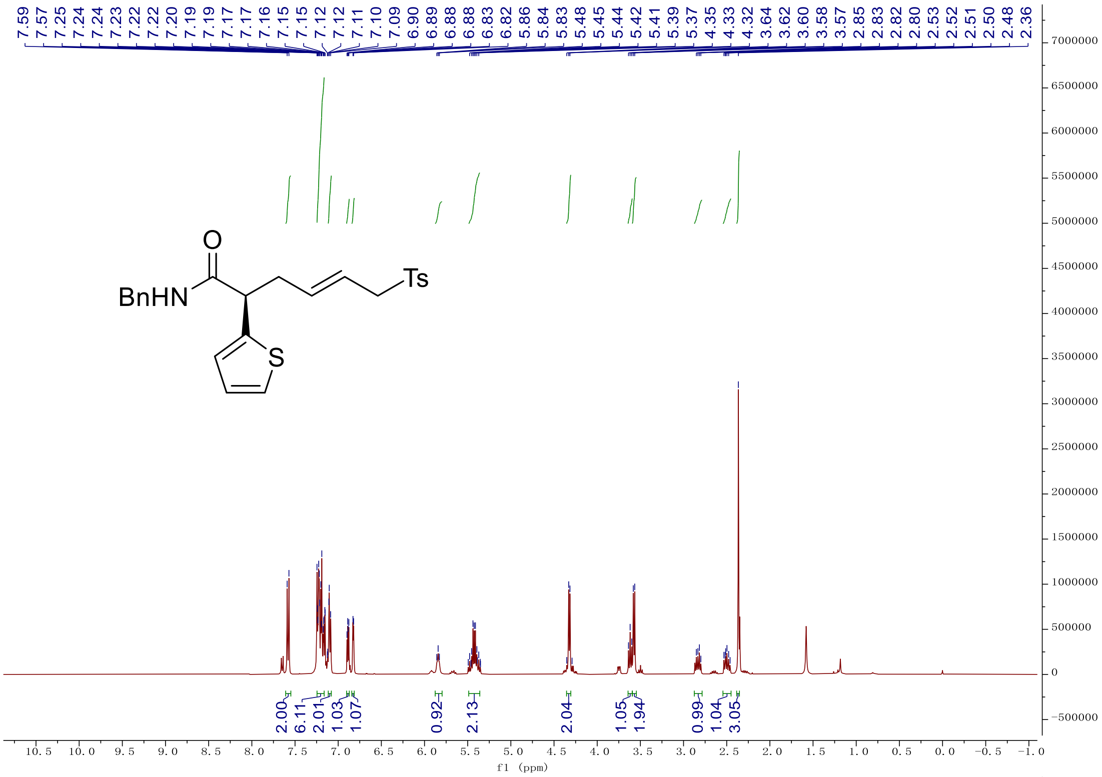

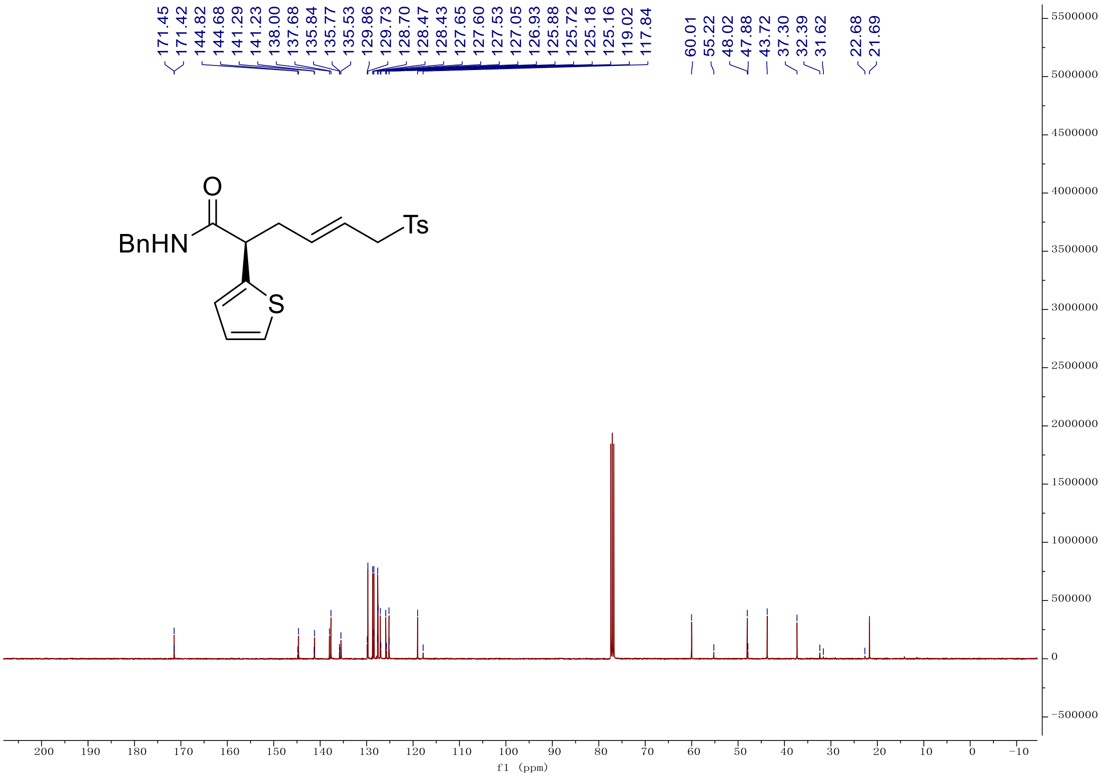

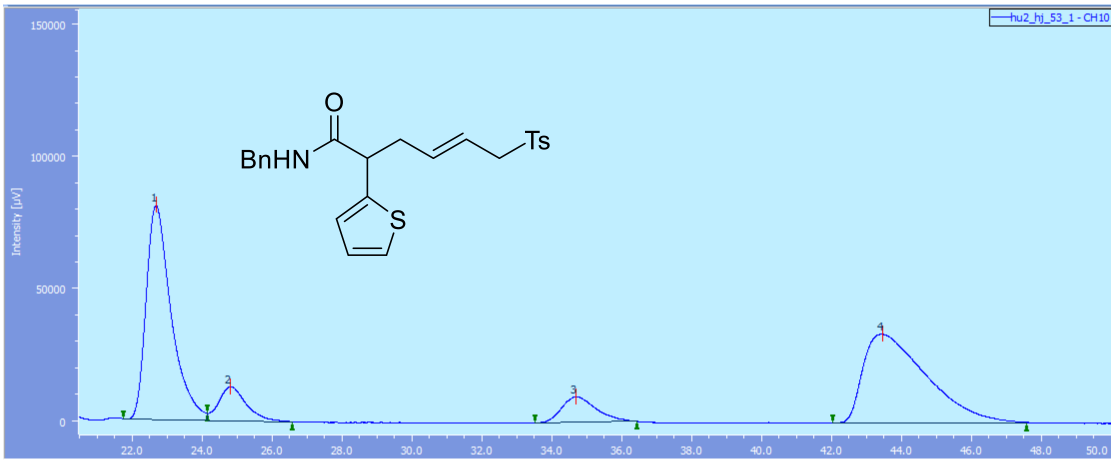

#	Peak Name	CH	tR	Area	Height	Area%	Height%	Quantity	NTP	Resolution	Symmetry Factor	Warning
1	Unknown	10	22.690	4010571	80713	41.969	59.134	N/A	5314	1.630	1.581	
2	Unknown	10	24.807	699979	12834	7.325	9.402	N/A	5331	6.306	N/A	
3	Unknown	10	34.690	651333	9465	6.816	6.934	N/A	6050	3.421	1.373	
4	Unknown	10	43.427	4194185	33481	43.890	24.530	N/A	2710	N/A	2.103	

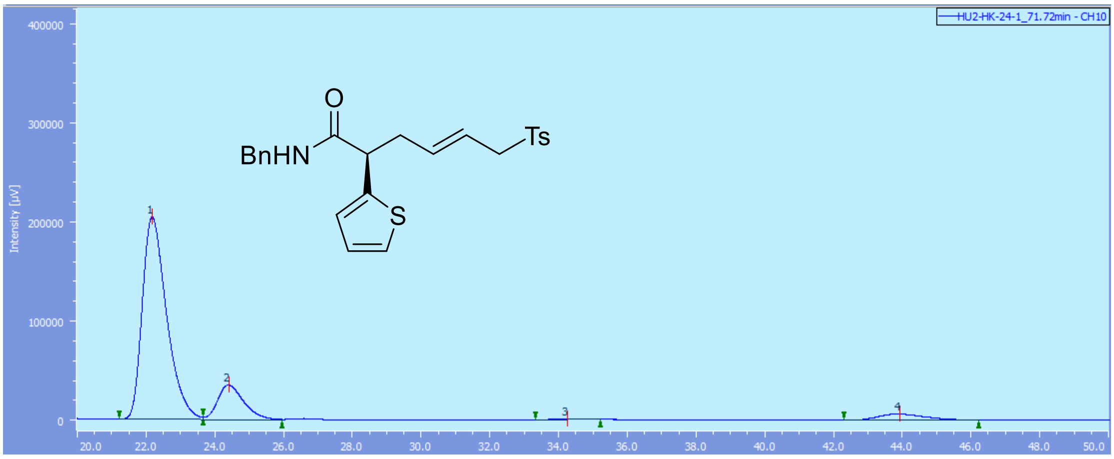

#	Peak Name	CH	tR	Area	Height	Area%	Height%	Quantity	NTP	Resolution	Symmetry Factor	Warning
1	Unknown	10	22.173	9992421	203735	80.427	82.948	N/A	4893	1.699	1.437	
2	Unknown	10	24.397	1815241	34663	14.611	14.113	N/A	5174	7.113	N/A	
3	Unknown	10	34.253	54659	1127	0.440	0.459	N/A	9283	5.019	0.954	
4	Unknown	10	43.913	561854	6092	4.522	2.480	N/A	5193	N/A	1.251	

(*S*,*E*)-*N*-(4-Methoxybenzyl)-2-(*p*-tolyl)-6-tosylhex-4-enamide (2.10)

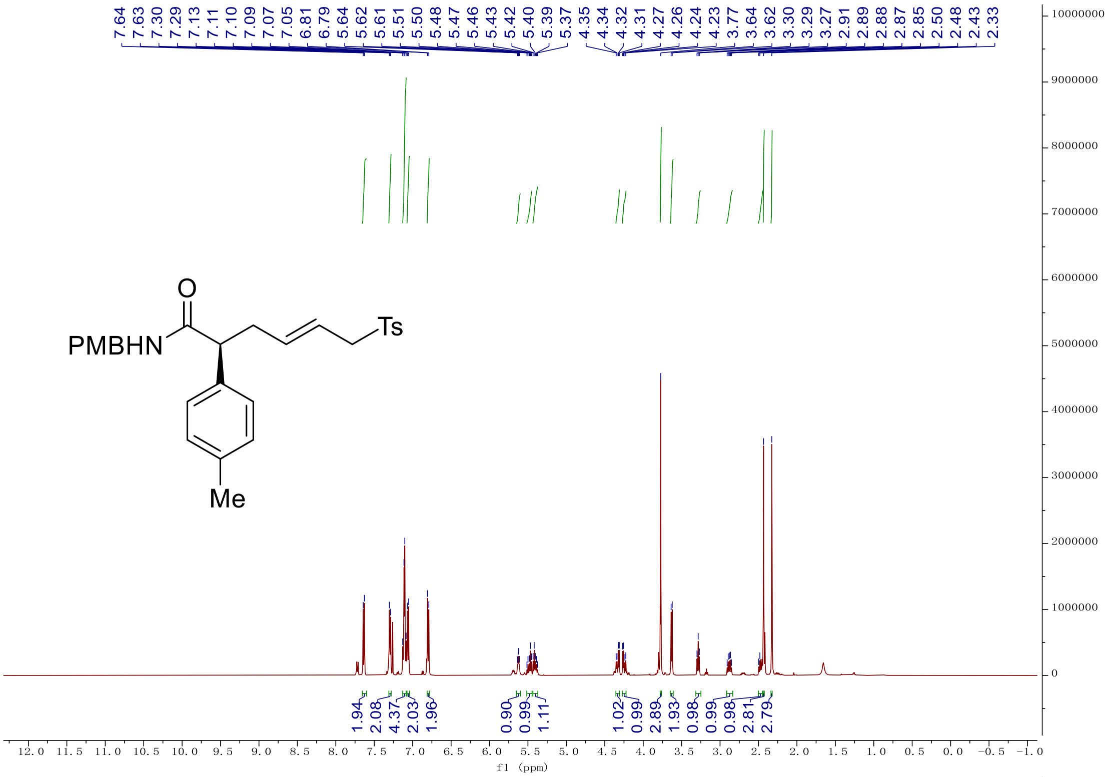

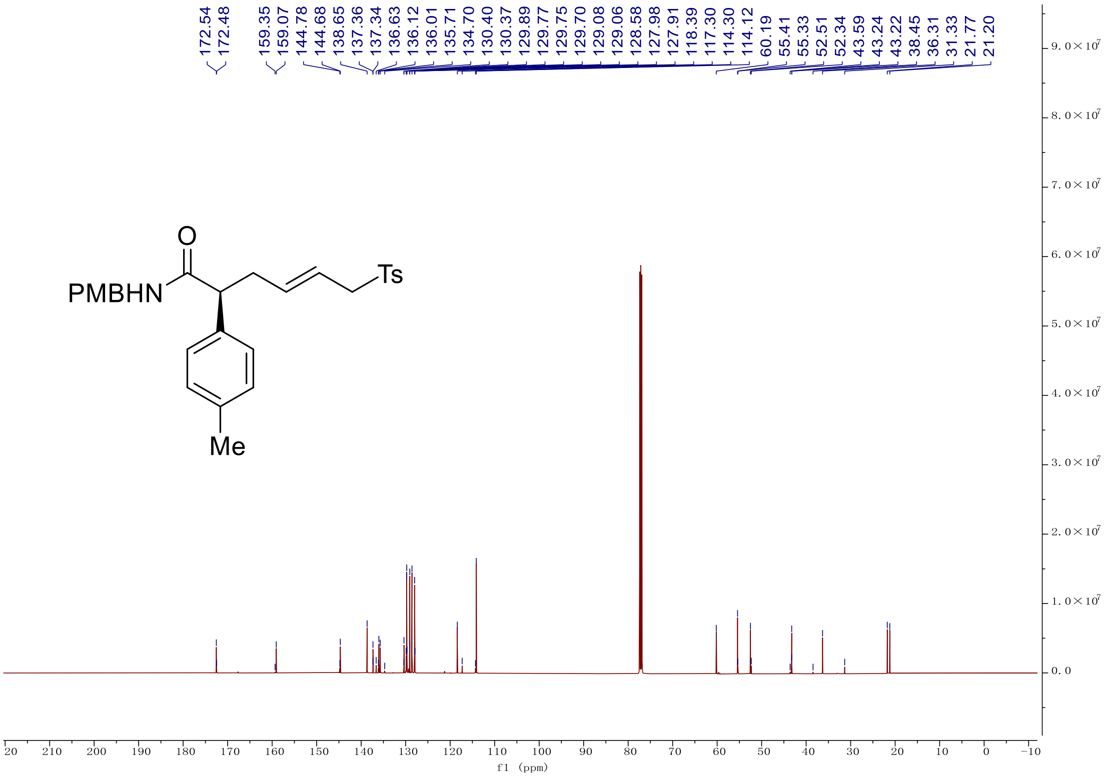

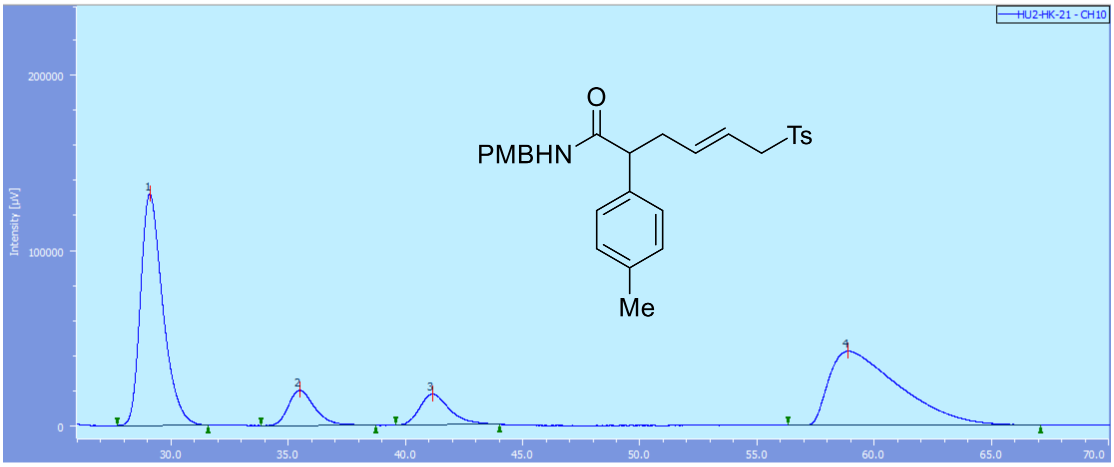

#	Peak Name	CH	tR	Area	Height	Area%	Height%	Quantity	NTP	Resolution	Symmetry Factor	Warning
1	Unknown	10	29.093	8690884	131591	42.435	62.349	N/A	4706	3.465	1.372	
2	Unknown	10	35.487	1566414	20001	7.648	9.477	N/A	5021	2.671	1.336	
3	Unknown	10	41.170	1558389	17370	7.609	8.230	N/A	5290	4.579	1.371	
4	Unknown	10	58.867	8664773	42092	42.308	19.944	N/A	1842	N/A	2.307	

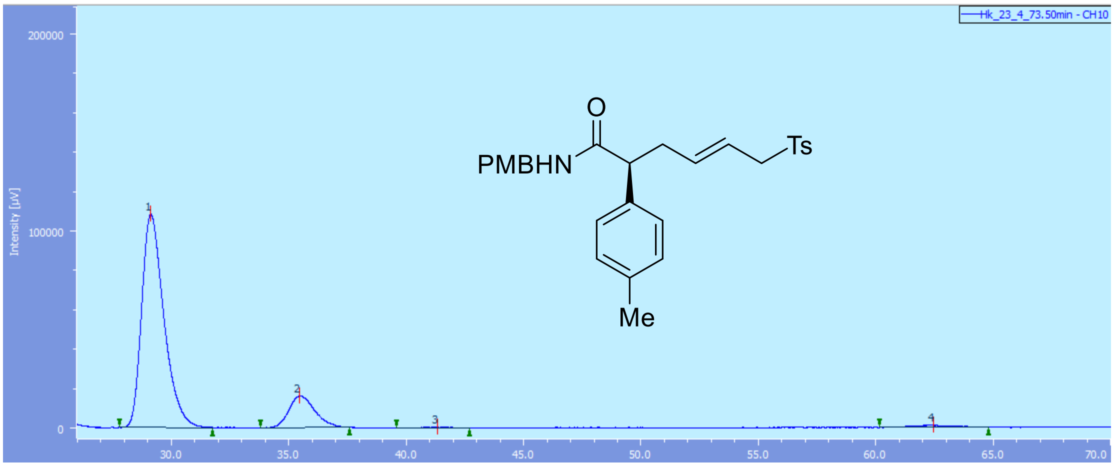

#	Peak Name	CH	tR	Area	Height	Area%	Height%	Quantity	NTP	Resolution	Symmetry Factor	Warning
1	Unknown	10	29.127	7093303	108050	83.976	86.190	N/A	4750	3.432	1.394	
2	Unknown	10	35.450	1220628	15926	14.451	12.704	N/A	5007	2.416	1.324	
3	Unknown	10	41.330	28220	418	0.334	0.333	N/A	3305	6.855	0.934	
4	Unknown	10	62.410	104665	968	1.239	0.772	N/A	5755	N/A	0.921	

(*S*,*E*)-*N*-Methyl-2-(*p*-tolyl)-6-tosylhex-4-enamide (2.11)

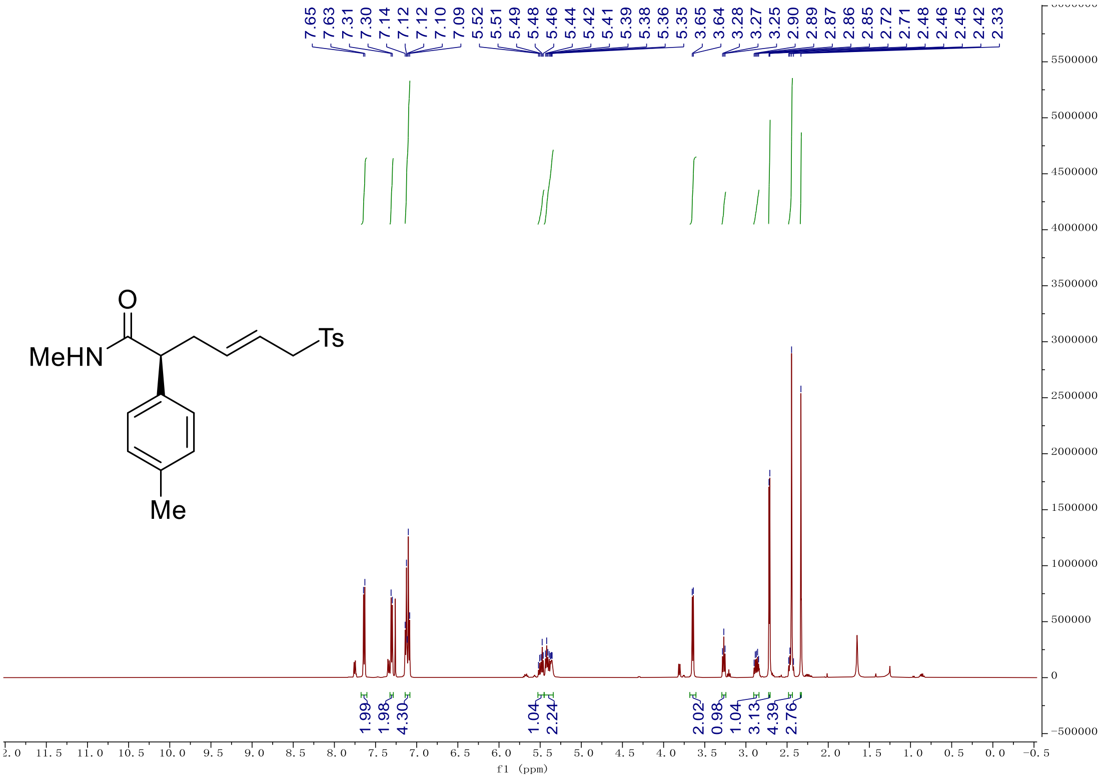

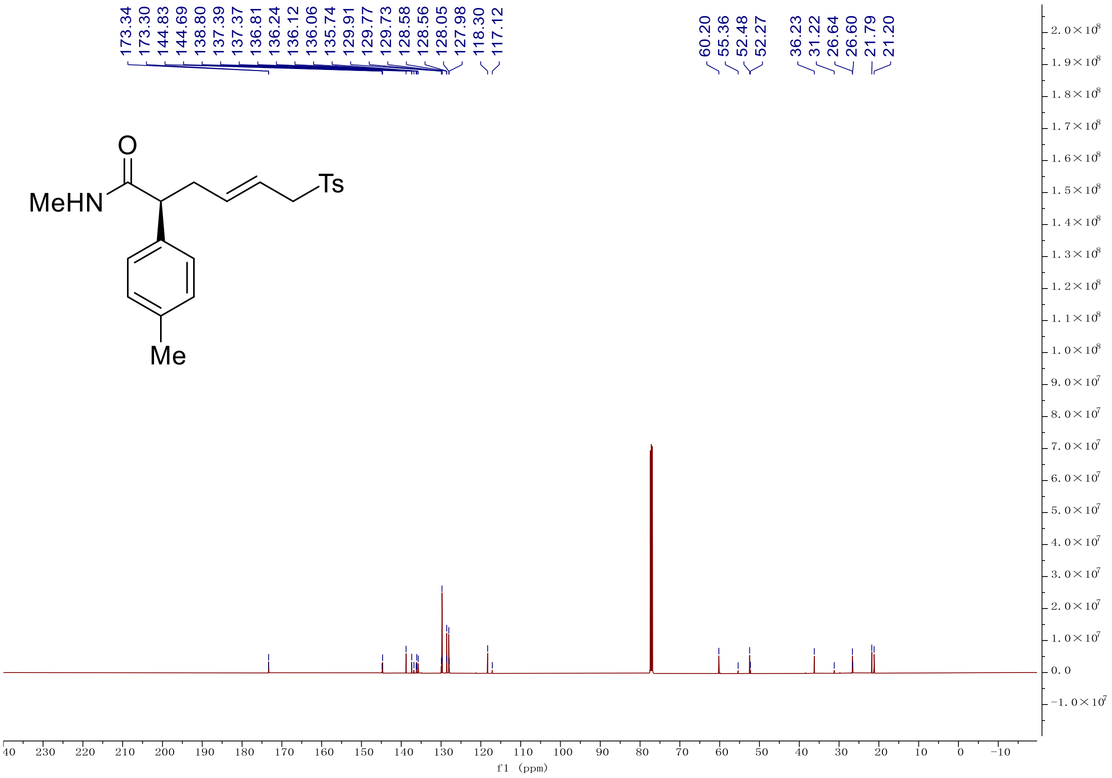

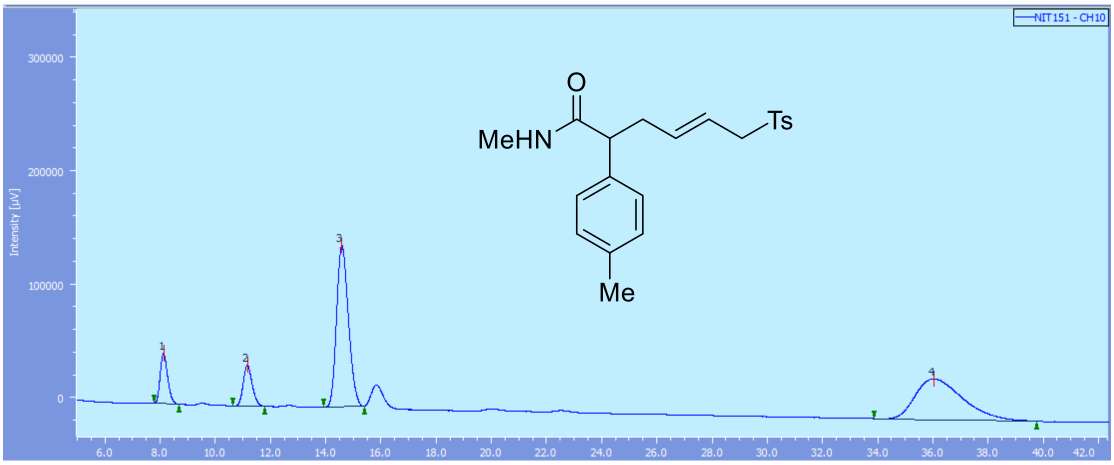

#	Peak Name	CH	tR	Area	Height	Area%	Height%	Quantity	NTP	Resolution	Symmetry Factor	Warning
1	Unknown	10	8.123	836656	44203	8.234	17.154	N/A	4241	5.566	1.220	
2	Unknown	10	11.157	810918	35854	7.981	13.914	N/A	5647	4.958	1.190	
3	Unknown	10	14.577	4220607	141751	41.538	55.011	N/A	5454	10.927	1.199	
4	Unknown	10	36.023	4292721	35869	42.247	13.920	N/A	2097	N/A	1.440	

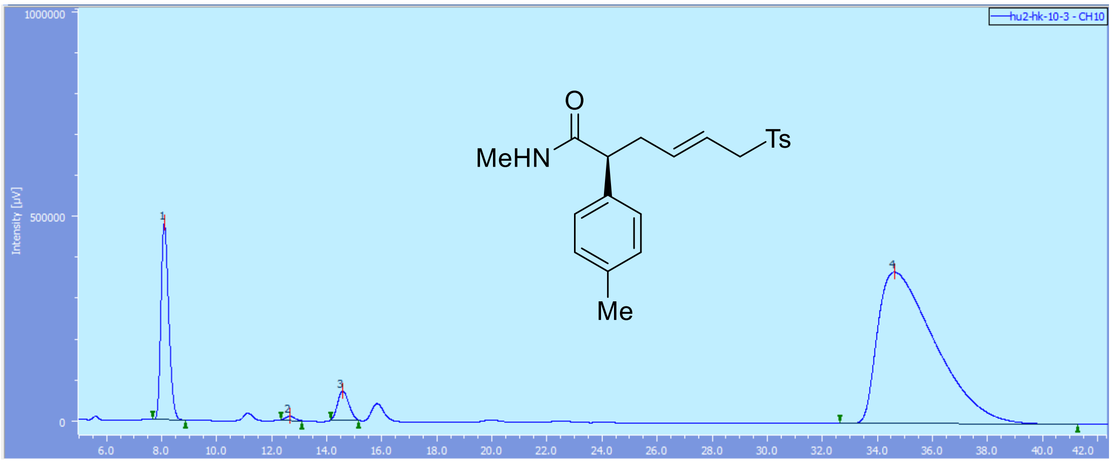

#	Peak Name	CH	tR	Area	Height	Area%	Height%	Quantity	NTP	Resolution	Symmetry Factor	Warning
1	Unknown	10	8.093	9366298	478365	14.410	51.516	N/A	3897	7.990	1.276	
2	Unknown	10	12.640	230875	10124	0.355	1.090	N/A	6597	2.794	1.170	
3	Unknown	10	14.573	2020367	71427	3.108	7.692	N/A	5802	8.697	1.142	
4	Unknown	10	34.593	53381782	368656	82.127	39.701	N/A	1291	N/A	2.162	

(*S*,*E*)-*N*-Propyl-2-(*p*-tolyl)-6-tosylhex-4-enamide (2.12)

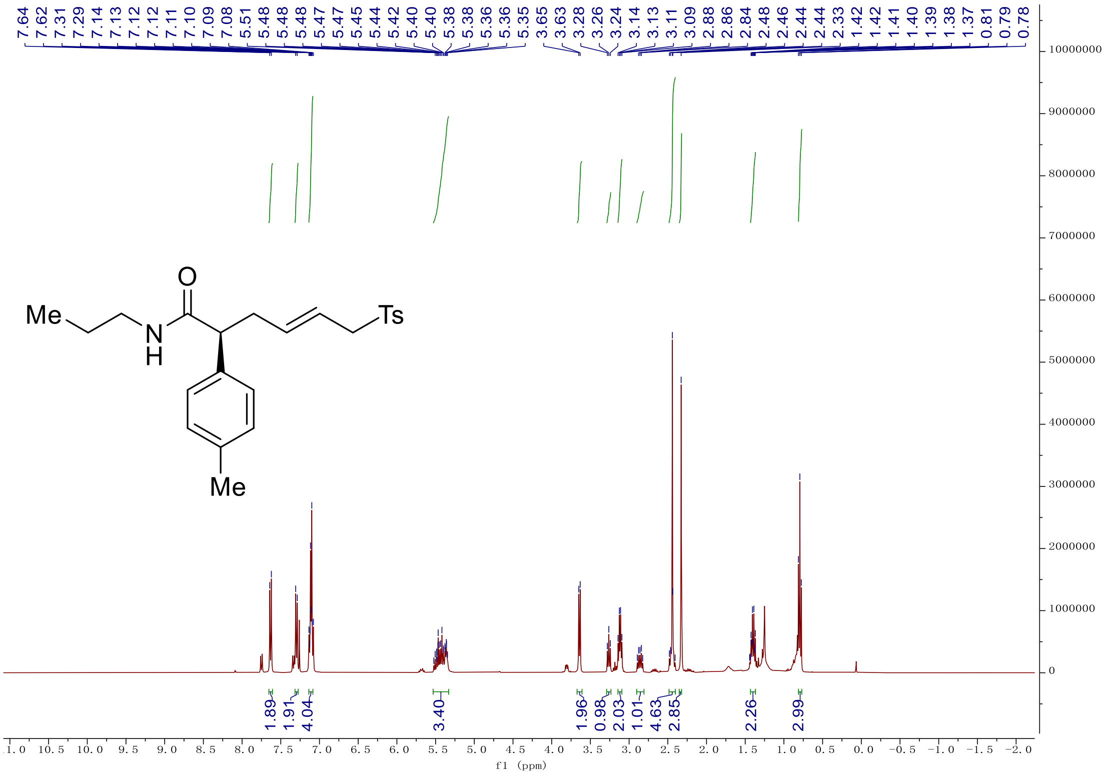

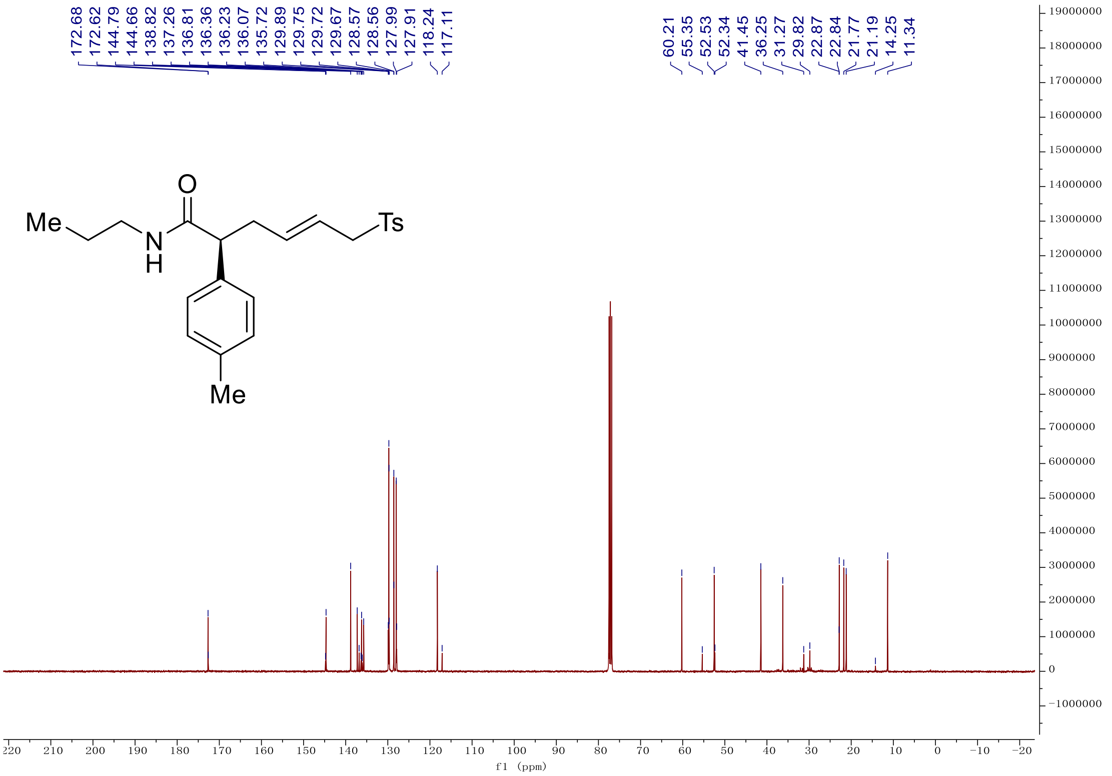

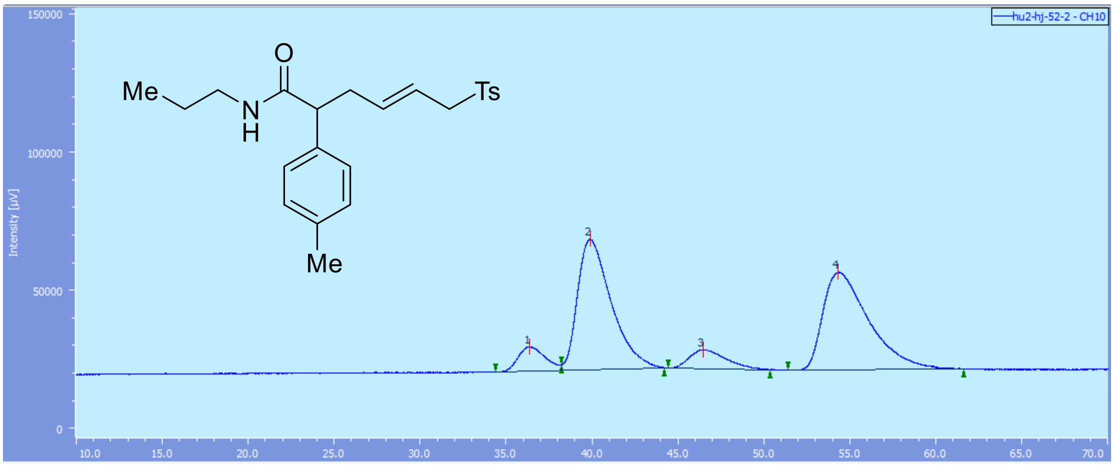

#	Peak Name	CH	tR	Area	Height	Area%	Height%	Quantity	NTP	Resolution	Symmetry Factor	Warning
1	Unknown	10	36.363	965462	8767	6.480	8.946	N/A	2364	1.081	N/A	
2	Unknown	10	39.870	6399397	47111	42.951	48.072	N/A	2059	1.751	1.548	
3	Unknown	10	46.423	1024563	6920	6.877	7.061	N/A	2165	1.802	1.486	
4	Unknown	10	54.263	6509874	35204	43.692	35.921	N/A	2101	N/A	1.908	

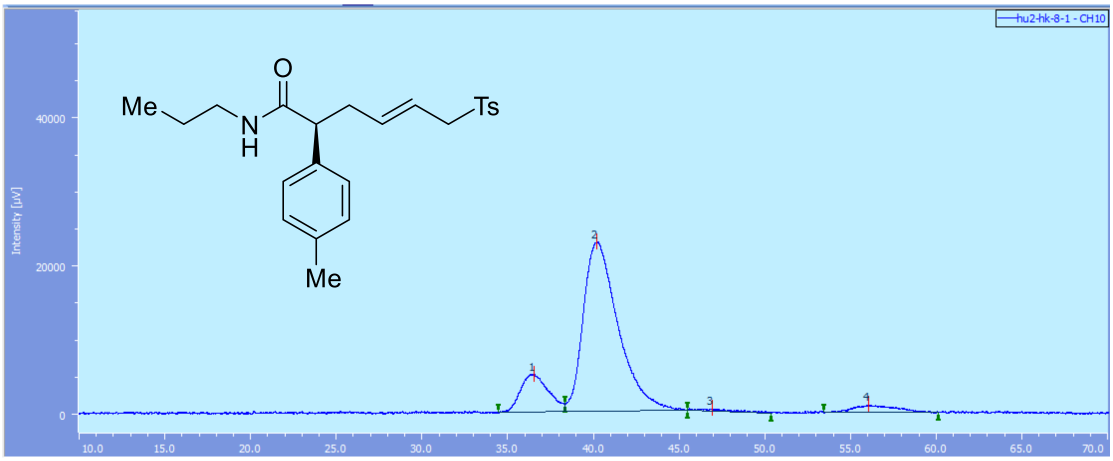

#	Peak Name	CH	tR	Area	Height	Area%	Height%	Quantity	NTP	Resolution	Symmetry Factor	Warning
1	Unknown	10	36.493	568067	5054	14.284	17.316	N/A	2204	1.110	N/A	
2	Unknown	10	40.197	3214541	22800	80.831	78.119	N/A	2018	1.203	1.480	
3	Unknown	10	46.887	26246	288	0.660	0.987	N/A	613	1.418	1.690	
4	Unknown	10	56.020	167990	1044	4.224	3.579	N/A	1759	N/A	1.336	

(*S*,*E*)-*N*-Benzyl-5-methyl-2-(*p*-tolyl)-6-tosylhex-4-enamide (*E*-2.13)

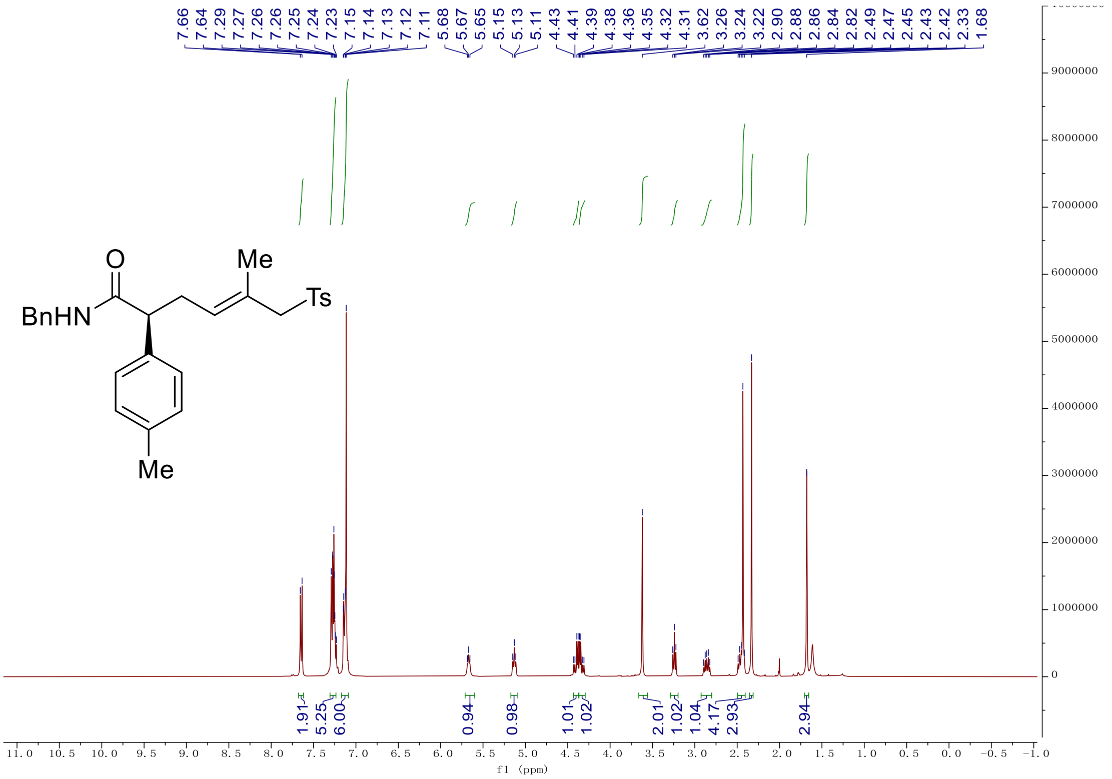

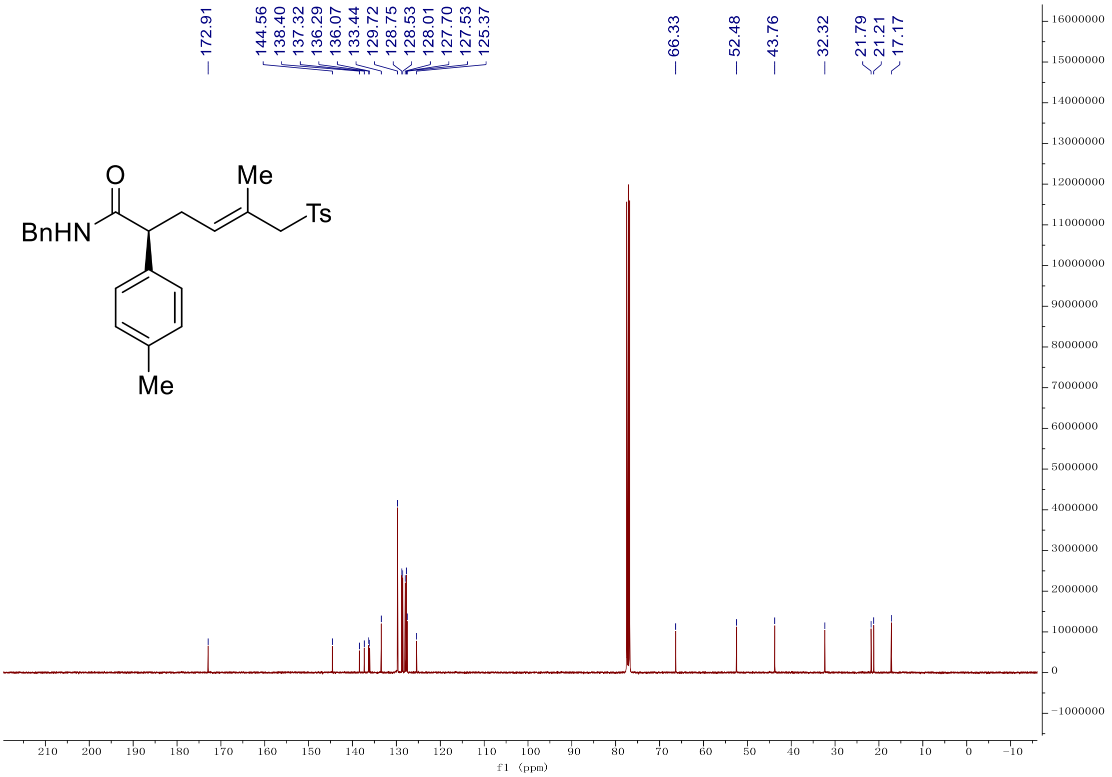

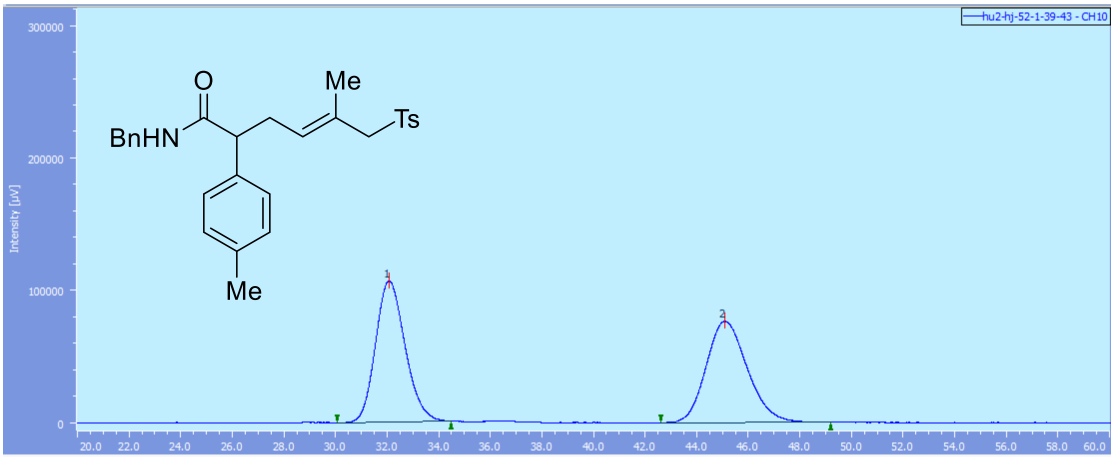

#	Peak Name	CH	tR	Area	Height	Area%	Height%	Quantity	NTP	Resolution	Symmetry Factor	Warning
1	Unknown	10	32.063	8444464	106305	50.023	58.237	N/A	3790	5.234	1.183	
2	Unknown	10	45.067	8436660	76234	49.977	41.763	N/A	3866	N/A	1.181	

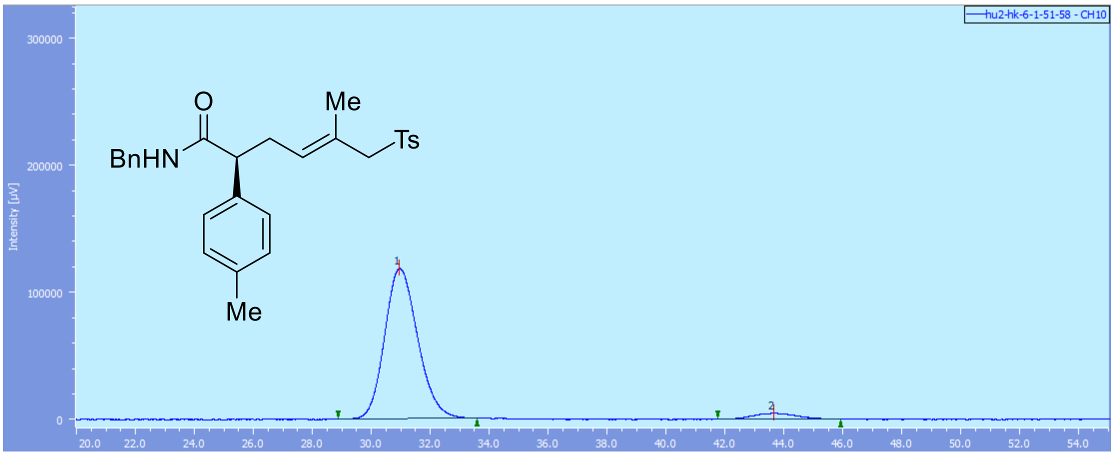

#	Peak Name	CH	tR	Area	Height	Area%	Height%	Quantity	NTP	Resolution	Symmetry Factor	Warning
1	Unknown	10	30.947	9166201	117953	94.849	96.019	N/A	3702	5.222	1.185	
2	Unknown	10	43.633	497750	4890	5.151	3.981	N/A	3784	N/A	1.036	

(*S*,*Z*)-*N*-Benzyl-5-methyl-2-(*p*-tolyl)-6-tosylhex-4-enamide (*Z*-2.13)

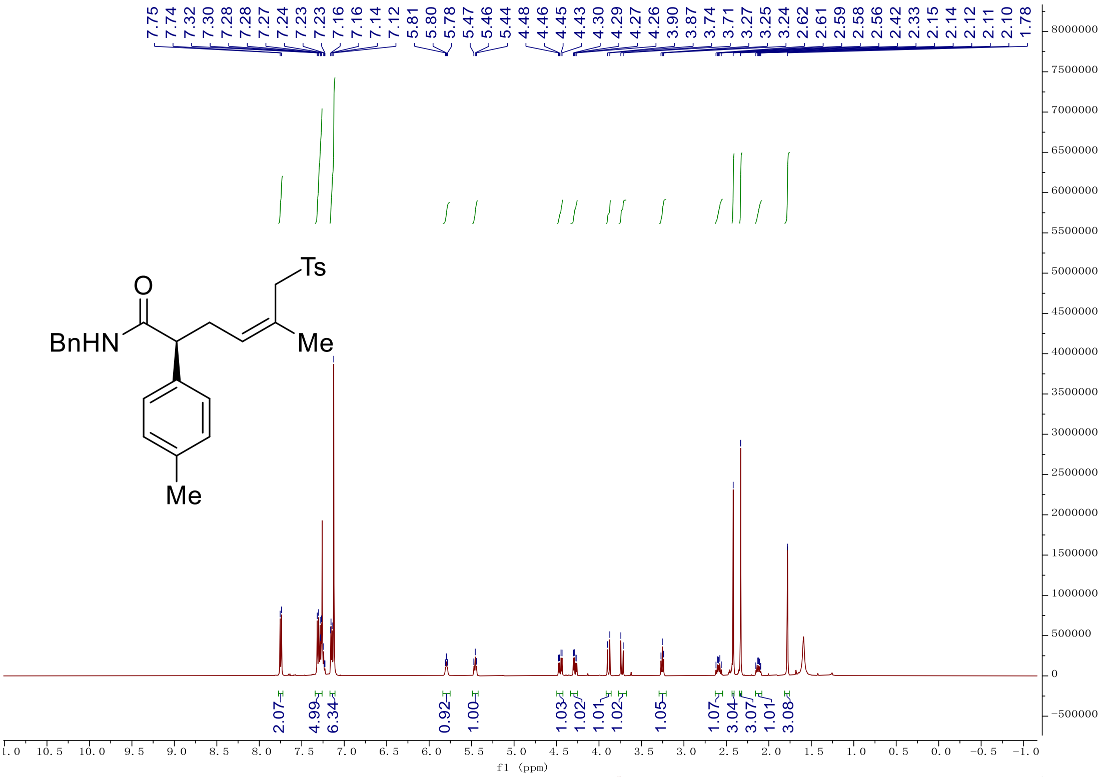

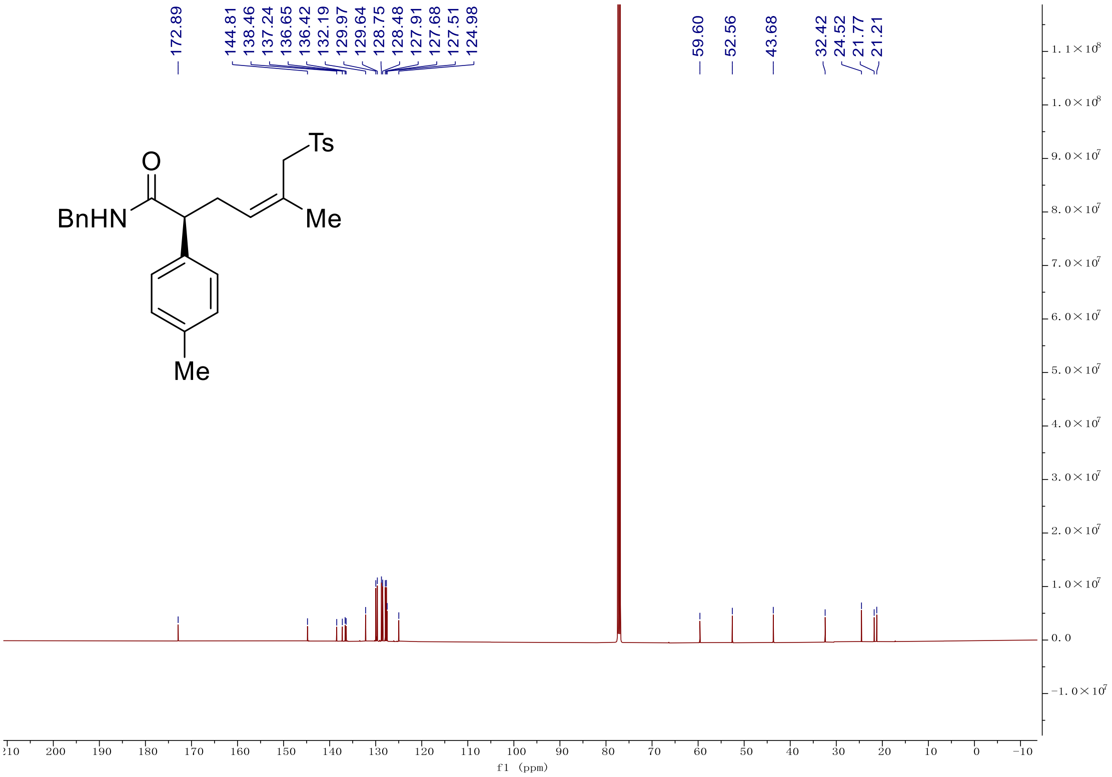

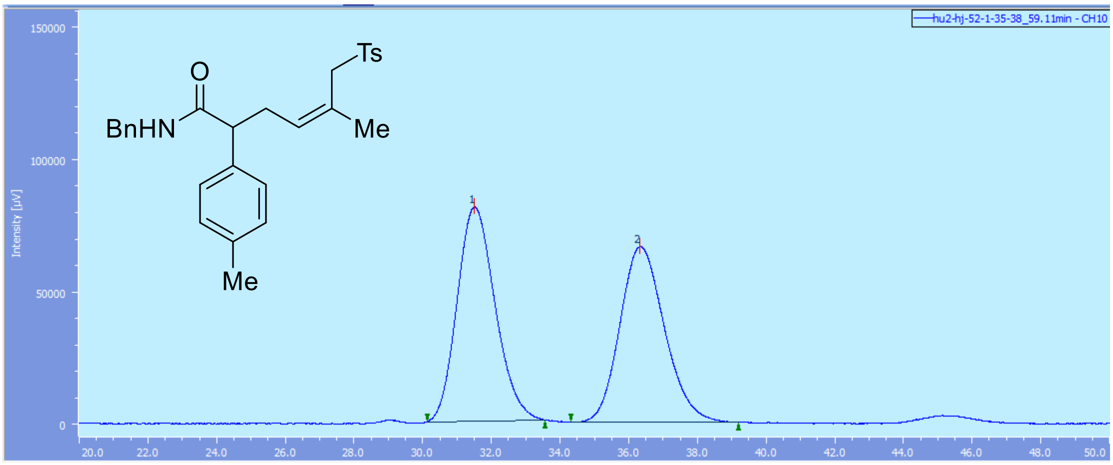

#	Peak Name	CH	tR	Area	Height	Area%	Height%	Quantity	NTP	Resolution	Symmetry Factor	Warning
1	Unknown	10	31.503	6209663	80723	51.044	55.008	N/A	3860	2.199	1.180	
2	Unknown	10	36.317	5955607	66026	48.956	44.992	N/A	3785	N/A	1.183	

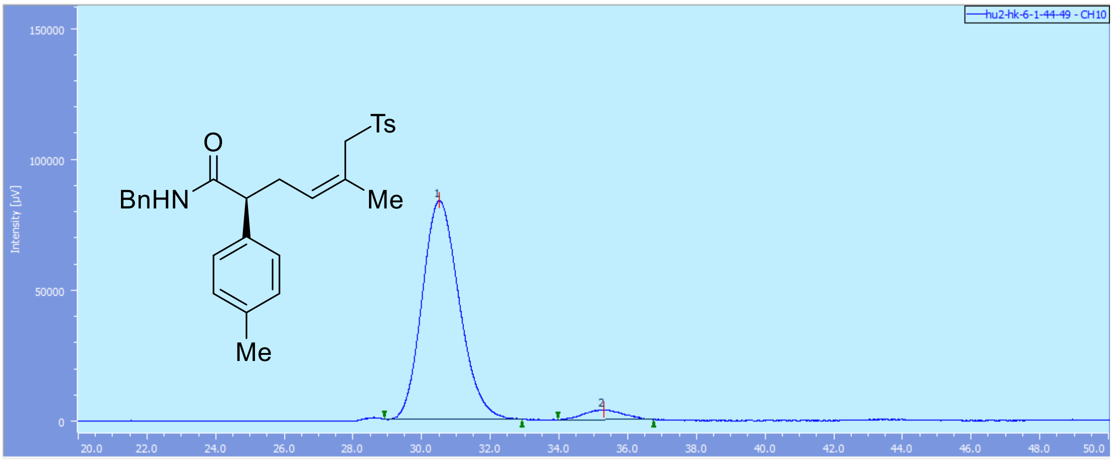

#	Peak Name	CH	tR	Area	Height	Area%	Height%	Quantity	NTP	Resolution	Symmetry Factor	Warning
1	Unknown	10	30.507	6238578	83281	95.413	95.631	N/A	3826	2.329	1.169	
2	Unknown	10	35.293	299904	3804	4.587	4.369	N/A	4318	N/A	1.014	

(*S*,*E*)-*N*-Benzyl-4-methyl-2-(*p*-tolyl)-6-tosylhex-4-enamide (*E*-2.14)

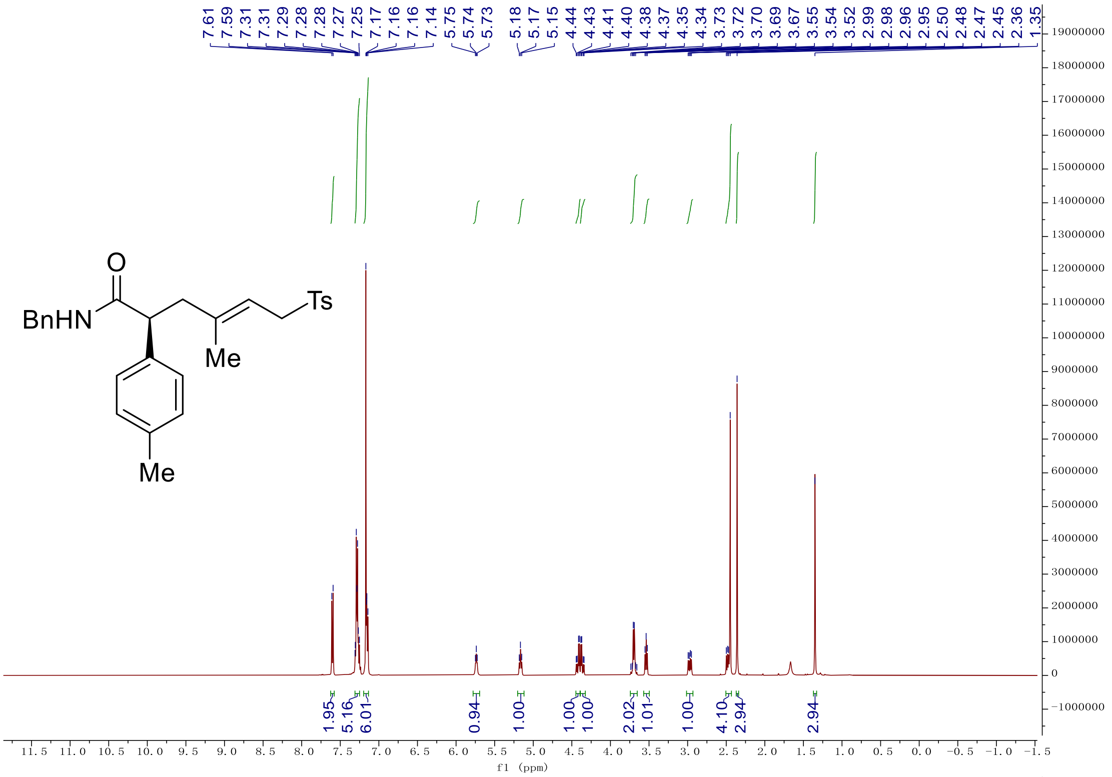

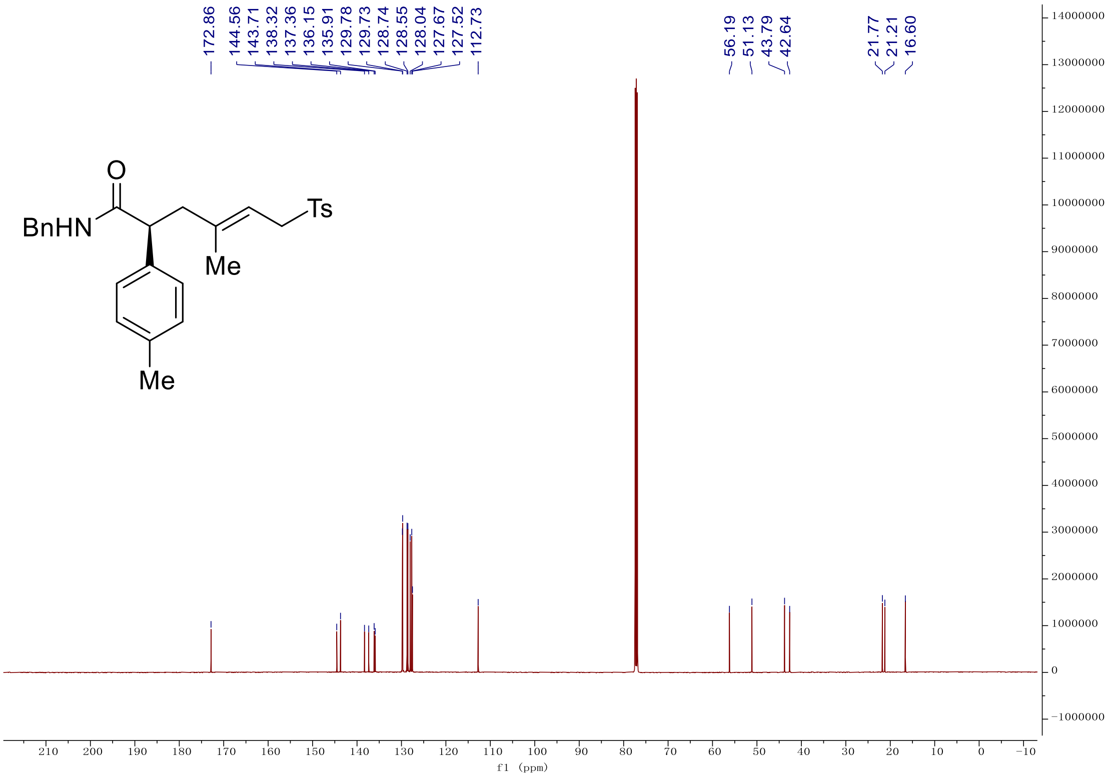

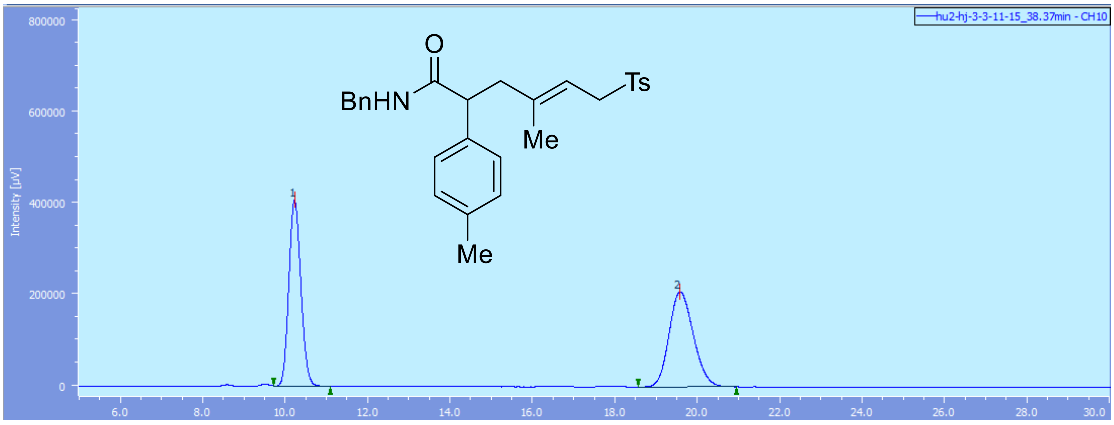

#	Peak Name	CH	tR	Area	Height	Area%	Height%	Quantity	NTP	Resolution	Symmetry Factor	Warning
1	Unknown	10	10.220	8234234	405704	49.223	66.131	N/A	5787	11.579	1.129	
2	Unknown	10	19.570	8494051	207780	50.777	33.869	N/A	5235	N/A	1.172	

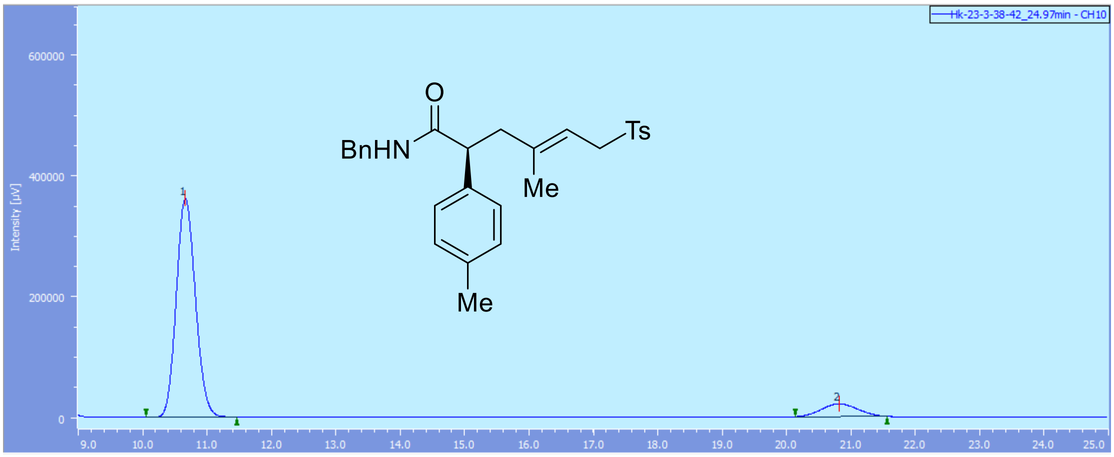

#	Peak Name	CH	tR	Area	Height	Area%	Height%	Quantity	NTP	Resolution	Symmetry Factor	Warning
1	Unknown	10	10.663	7438067	360199	90.079	94.523	N/A	6088	12.602	1.111	
2	Unknown	10	20.810	819239	20871	9.921	5.477	N/A	6076	N/A	1.039	

(*S*,*Z*)-*N*-Benzyl-4-methyl-2-(*p*-tolyl)-6-tosylhex-4-enamide (*Z*-2.14)

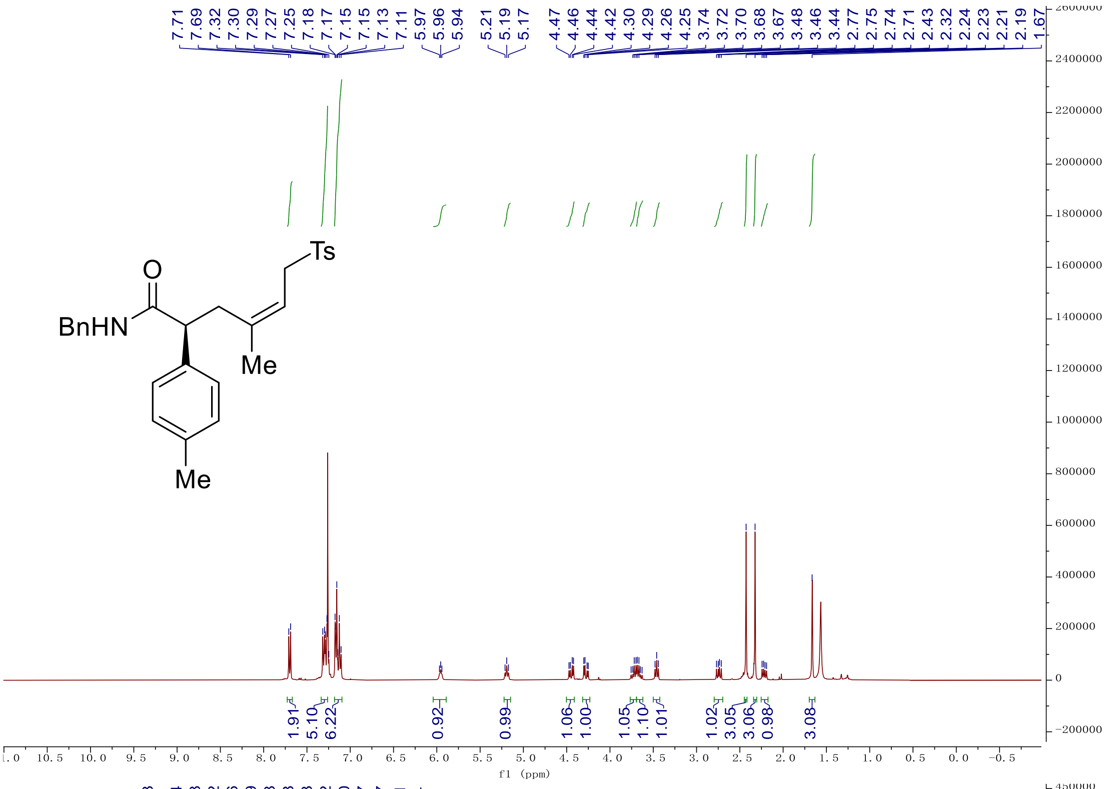

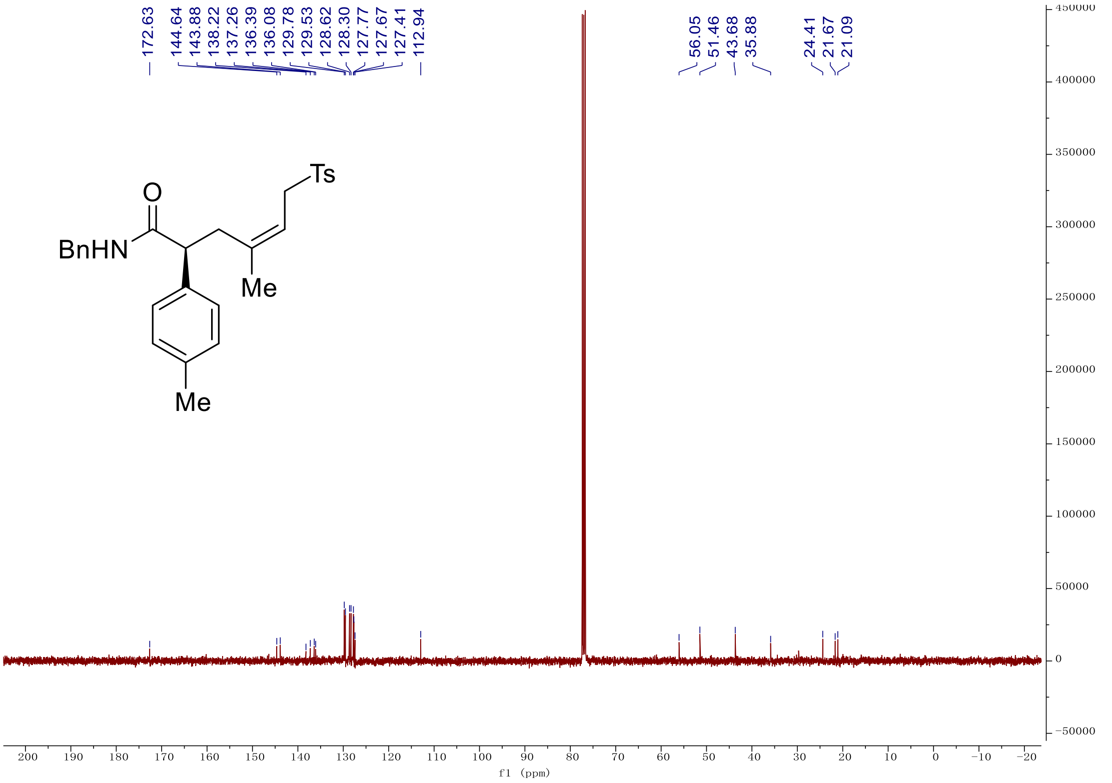

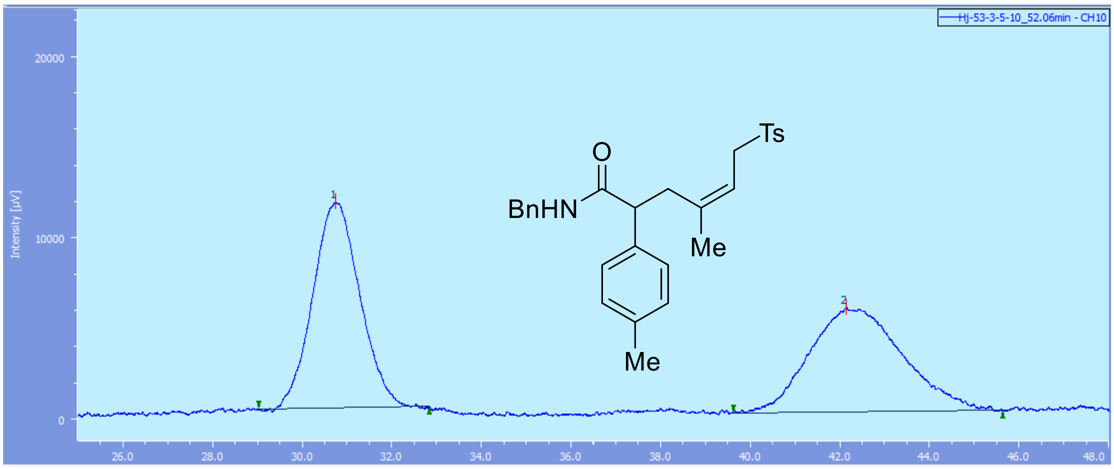

#	Peak Name	CH	tR	Area	Height	Area%	Height%	Quantity	NTP	Resolution	Symmetry Factor	Warning
1	Unknown	10	30.733	821562	11318	50.151	66.341	N/A	4109	3.987	1.064	
2	Unknown	10	42.127	816613	5742	49.849	33.659	N/A	1954	N/A	1.266	

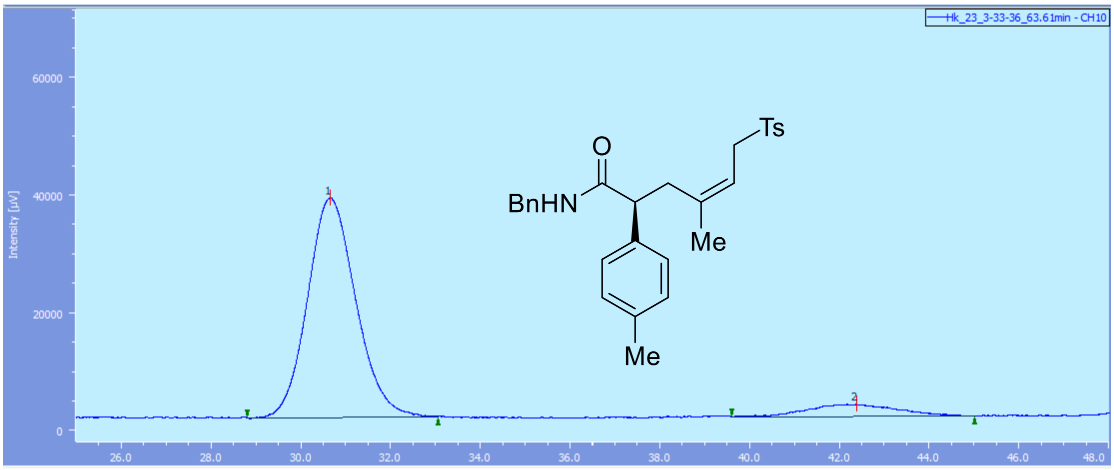

#	Peak Name	CH	tR	Area	Height	Area%	Height%	Quantity	NTP	Resolution	Symmetry Factor	Warning
1	Unknown	10	30.660	2777014	37285	90.552	94.700	N/A	4010	4.083	1.122	
2	Unknown	10	42.367	289761	2087	9.448	5.300	N/A	1975	N/A	0.982	

(*S*,*E*)-*N*-Benzyl-6-((4-(*tert*-butyl)phenyl)sulfonyl)-2-(*p*-tolyl)hex-4-enamide (2.15)

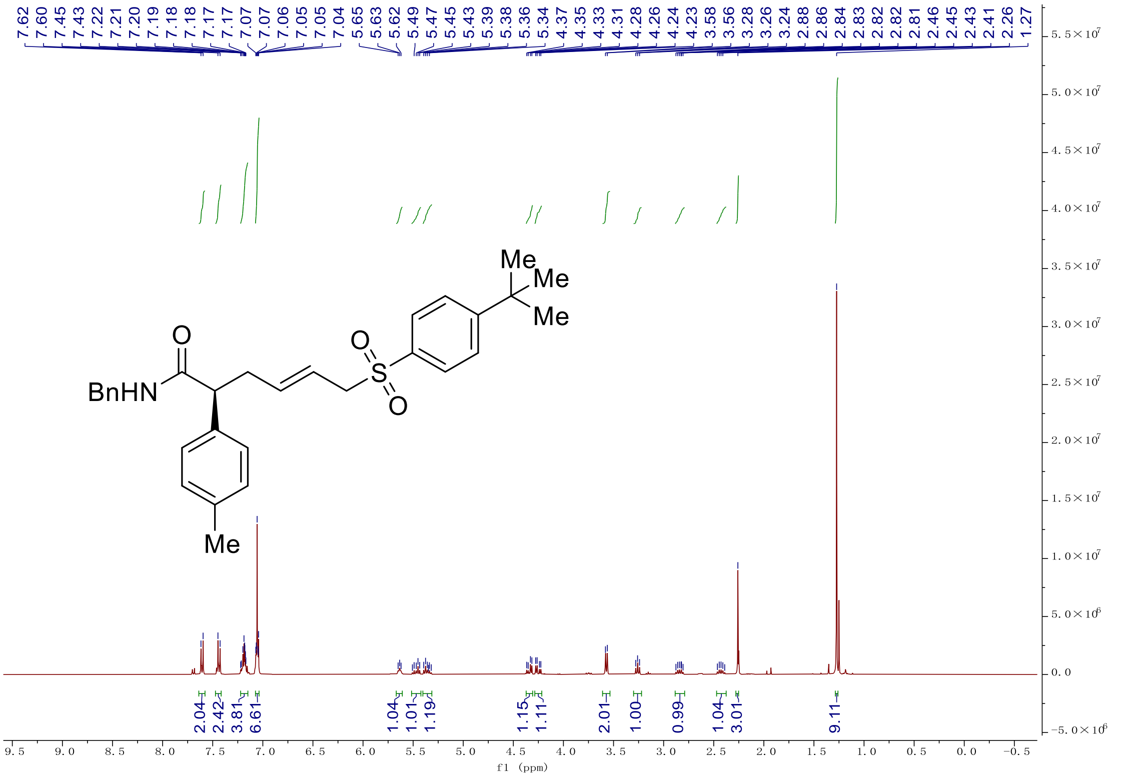

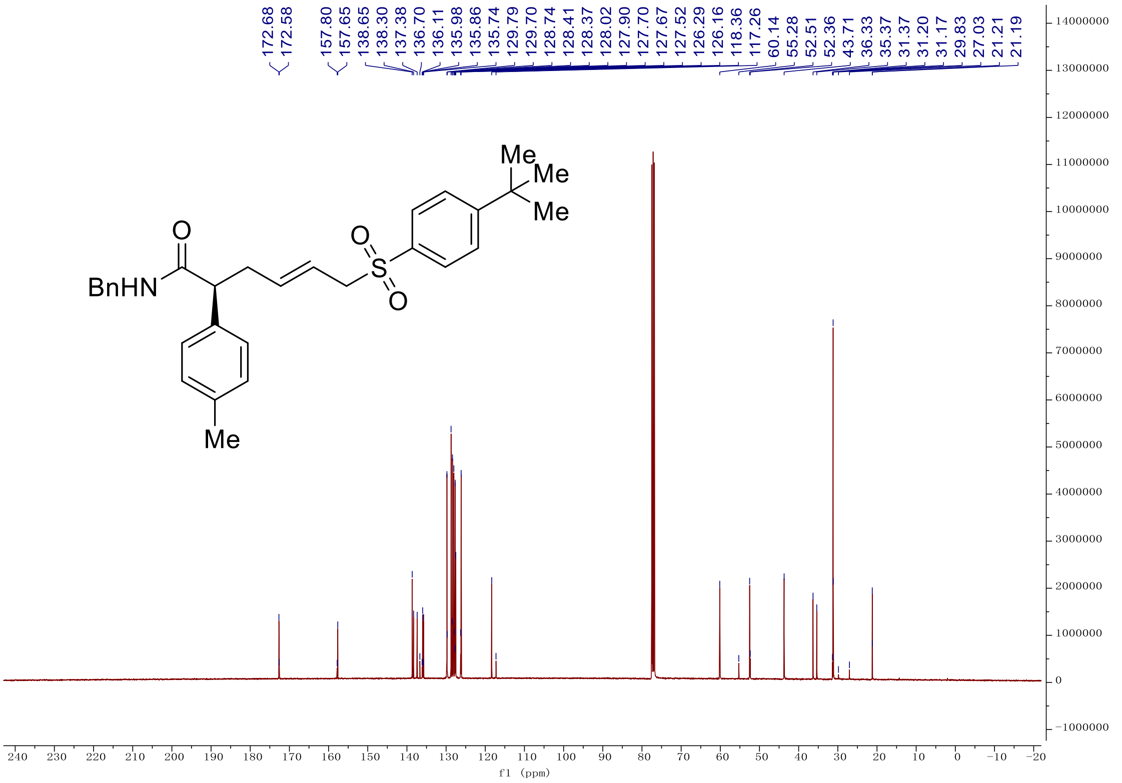

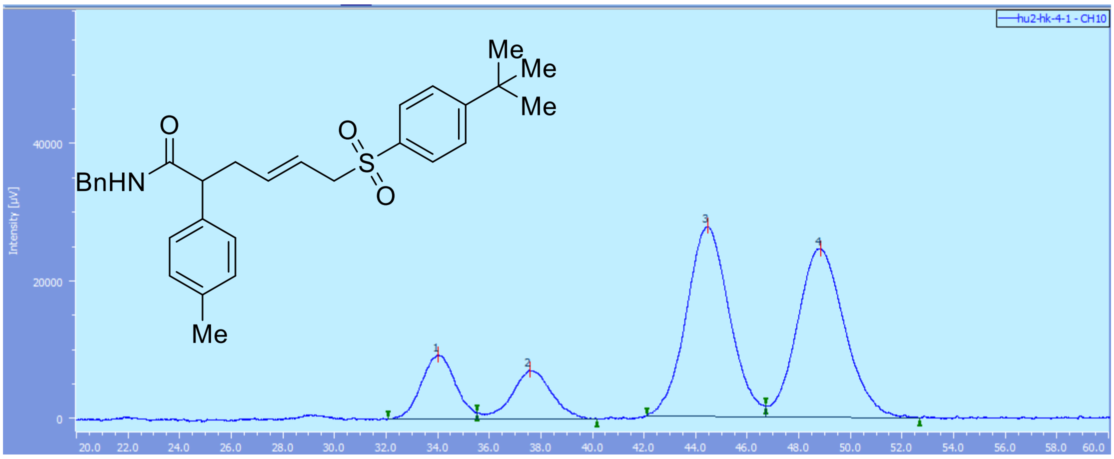

#	Peak Name	CH	tR	Area	Height	Area%	Height%	Quantity	NTP	Resolution	Symmetry Factor	Warning
1	Unknown	10	34.013	847671	9309	10.722	13.624	N/A	3106	1.384	N/A	
2	Unknown	10	37.560	753083	7102	9.525	10.395	N/A	3103	2.422	N/A	
3	Unknown	10	44.453	3183213	27513	40.262	40.266	N/A	3487	1.379	N/A	
4	Unknown	10	48.823	3122227	24403	39.491	35.715	N/A	3409	N/A	N/A	

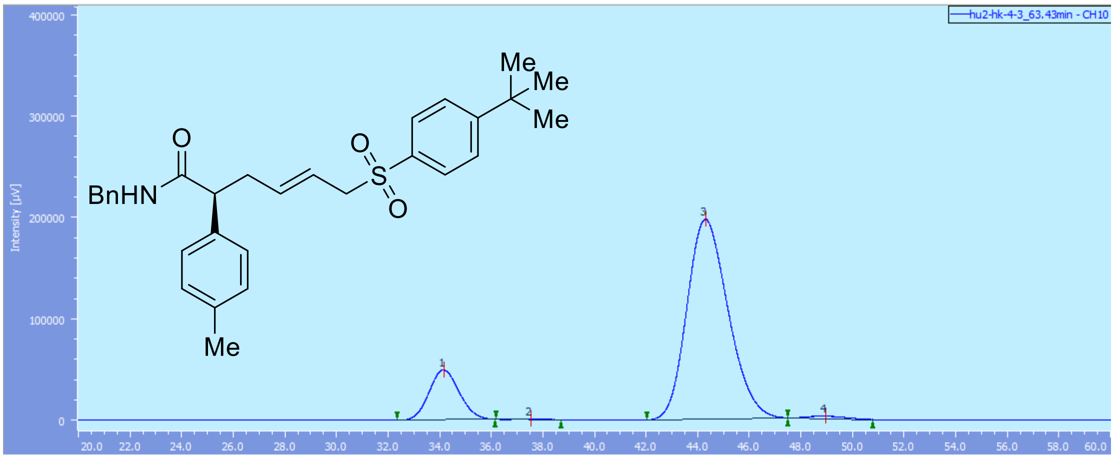

#	Peak Name	CH	tR	Area	Height	Area%	Height%	Quantity	NTP	Resolution	Symmetry Factor	Warning
1	Unknown	10	34.157	3988844	49048	15.318	19.715	N/A	4011	1.806	1.088	
2	Unknown	10	37.543	29926	582	0.115	0.234	N/A	8773	2.982	0.914	
3	Unknown	10	44.287	21786220	196636	83.665	79.038	N/A	3652	1.633	1.193	
4	Unknown	10	48.933	234928	2521	0.902	1.013	N/A	4973	N/A	1.188	

(*S*,*E*)-*N*-Benzyl-6-((4-methoxyphenyl)sulfonyl)-2-(*p*-tolyl)hex-4-enamide (2.16)

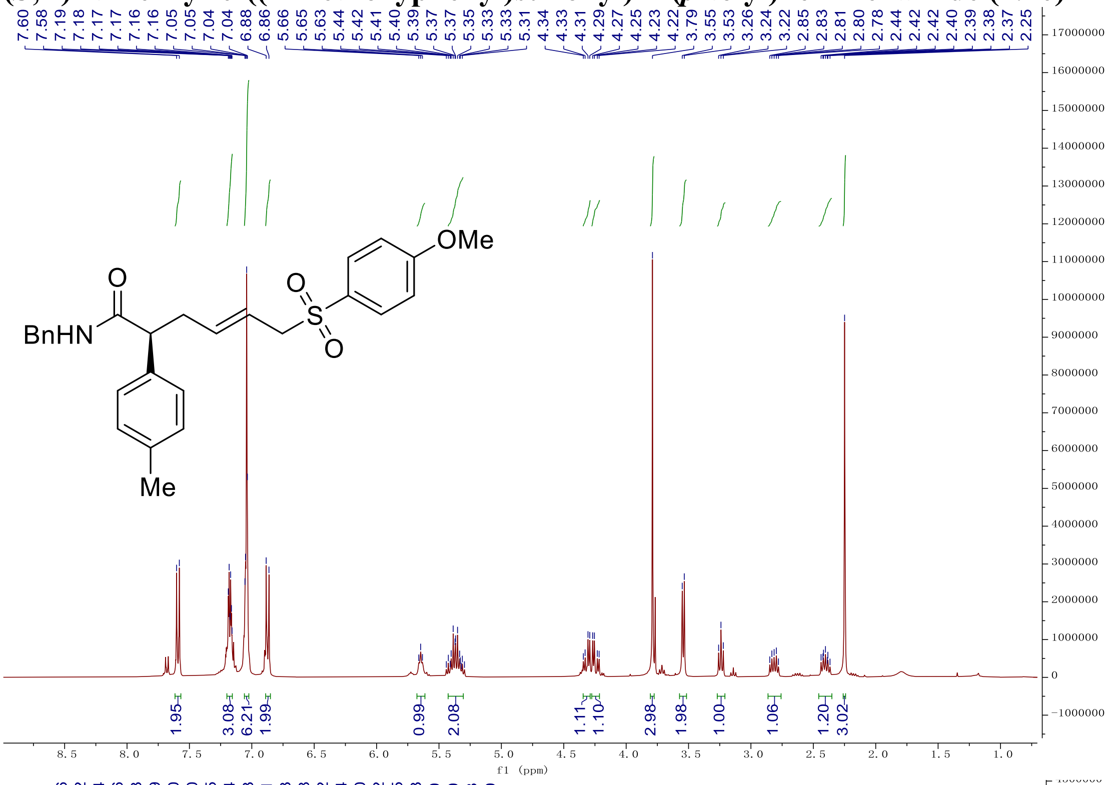

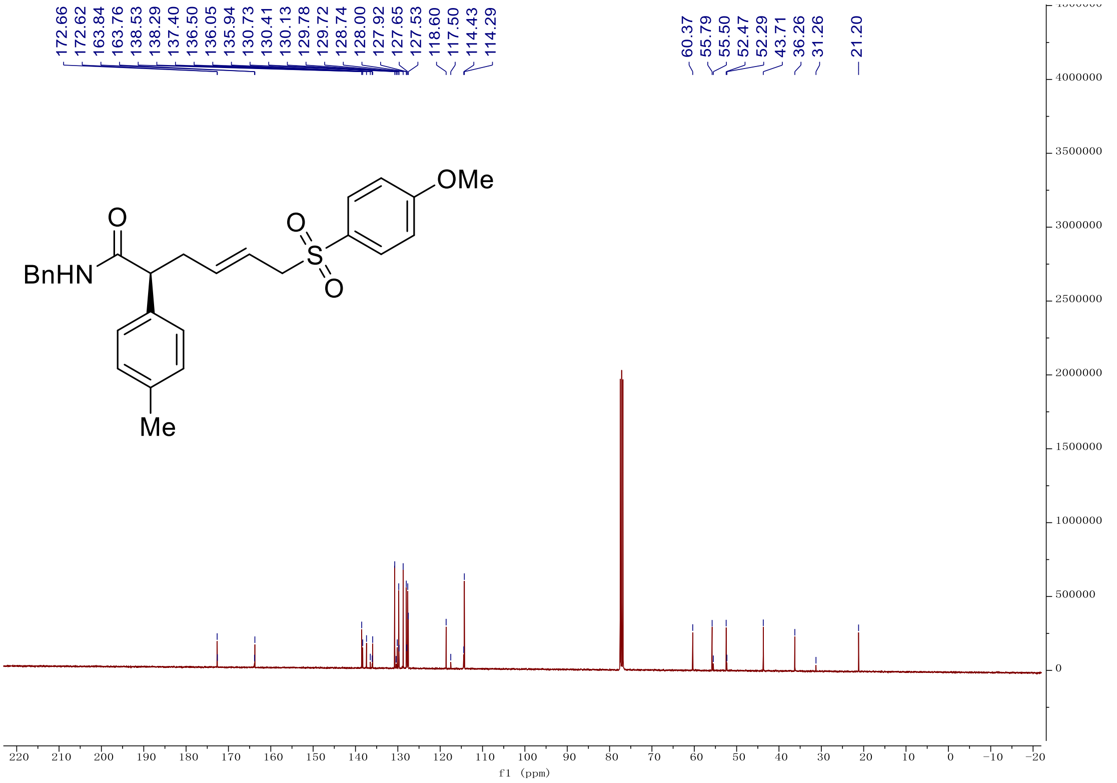

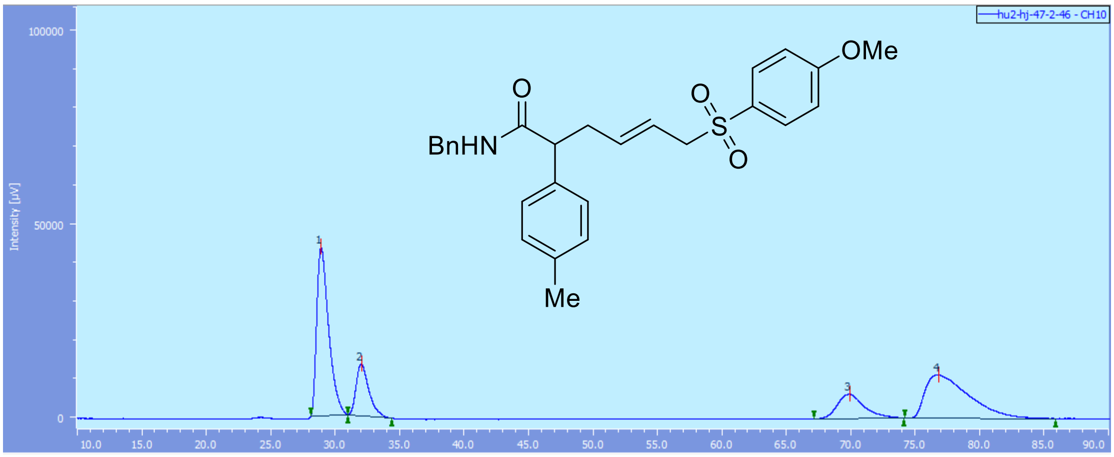

#	Peak Name	CH	tR	Area	Height	Area%	Height%	Quantity	NTP	Resolution	Symmetry Factor	Warning
1	Unknown	10	28.900	2800326	43388	38.565	58.497	N/A	4788	1.829	1.639	
2	Unknown	10	32.020	915739	13369	12.611	18.025	N/A	5353	14.087	1.484	
3	Unknown	10	69.867	913948	6283	12.587	8.471	N/A	5904	1.402	1.374	
4	Unknown	10	76.787	2631238	11132	36.237	15.008	N/A	2408	N/A	2.111	

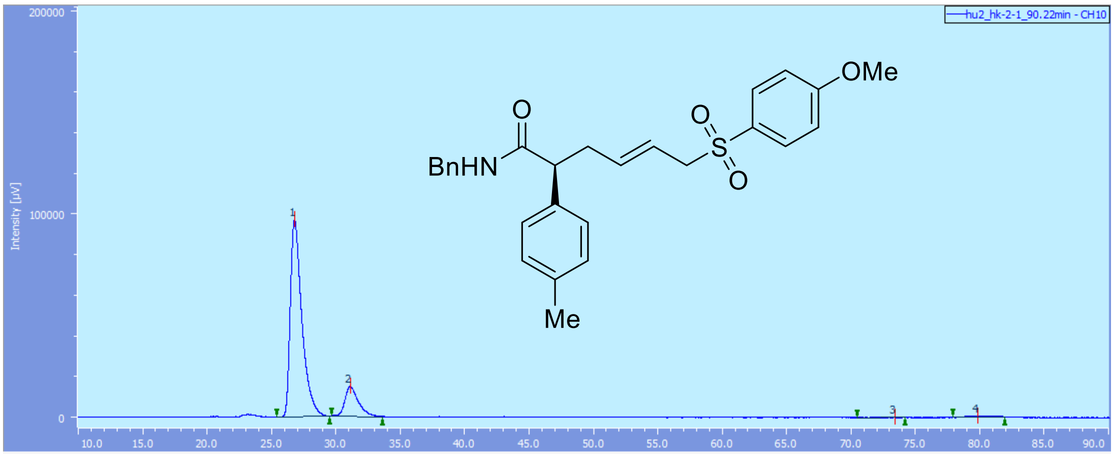

#	Peak Name	CH	tR	Area	Height	Area%	Height%	Quantity	NTP	Resolution	Symmetry Factor	Warning
1	Unknown	10	26.783	6032321	96727	84.040	86.293	N/A	4719	2.580	1.708	
2	Unknown	10	31.110	1043534	14468	14.538	12.907	N/A	4764	12.348	1.513	
3	Unknown	10	73.383	6976	101	0.097	0.090	N/A	3362	1.361	0.612	
4	Unknown	10	79.780	95107	796	1.325	0.710	N/A	5351	N/A	1.152	

(*S*,*E*)-*N*-Benzyl-6-(phenylsulfonyl)-2-(*p*-tolyl)hex-4-enamide (2.17)

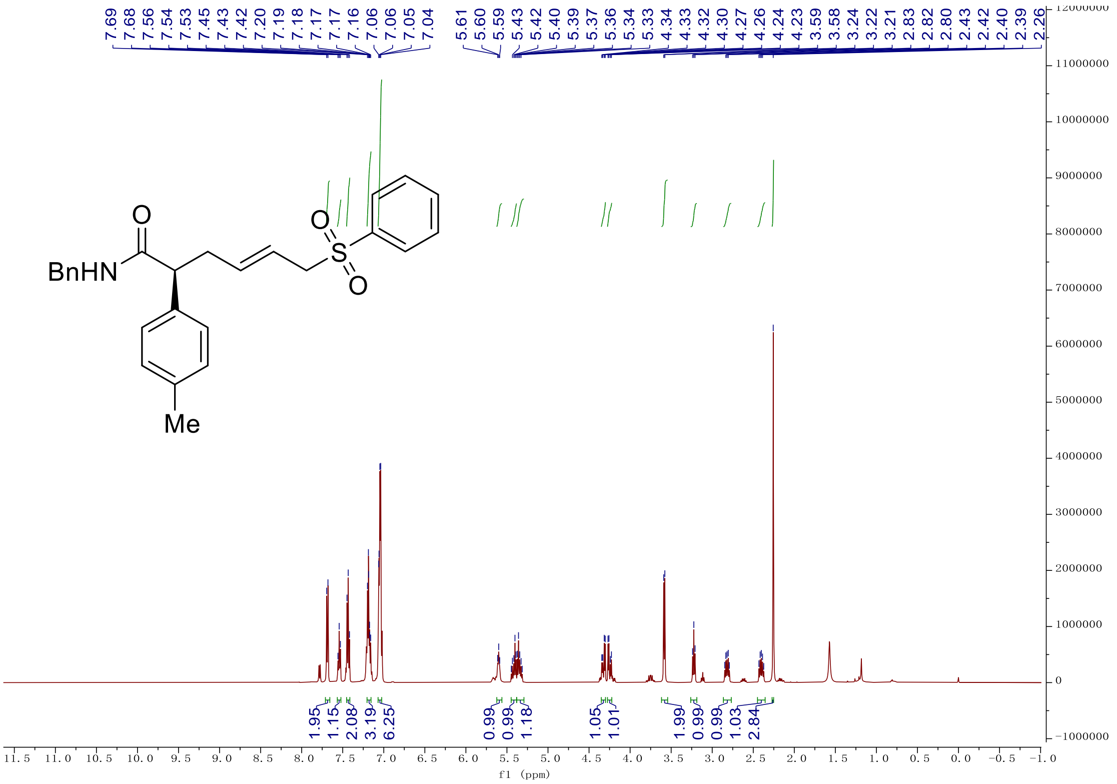

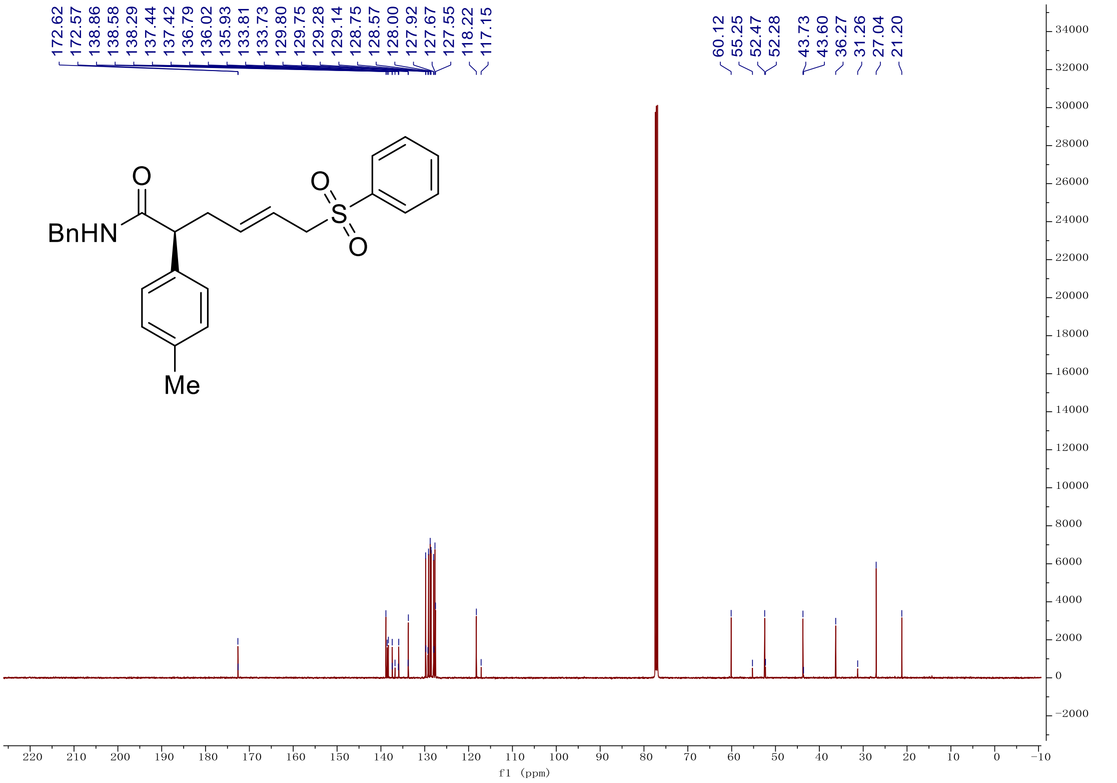

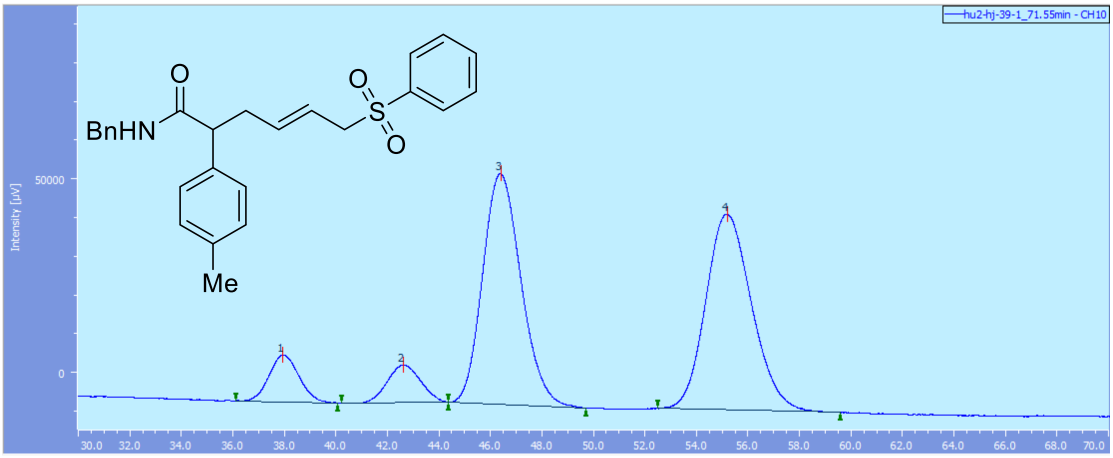

#	Peak Name	CH	tR	Area	Height	Area%	Height%	Quantity	NTP	Resolution	Symmetry Factor	Warning
1	Unknown	10	37.930	967450	12034	6.791	9.136	N/A	5032	2.043	1.103	
2	Unknown	10	42.597	872069	9676	6.122	7.346	N/A	4866	1.468	1.041	
3	Unknown	10	46.383	6157770	59525	43.227	45.189	N/A	4617	2.953	1.163	
4	Unknown	10	55.180	6247933	50488	43.860	38.329	N/A	4630	N/A	1.141	

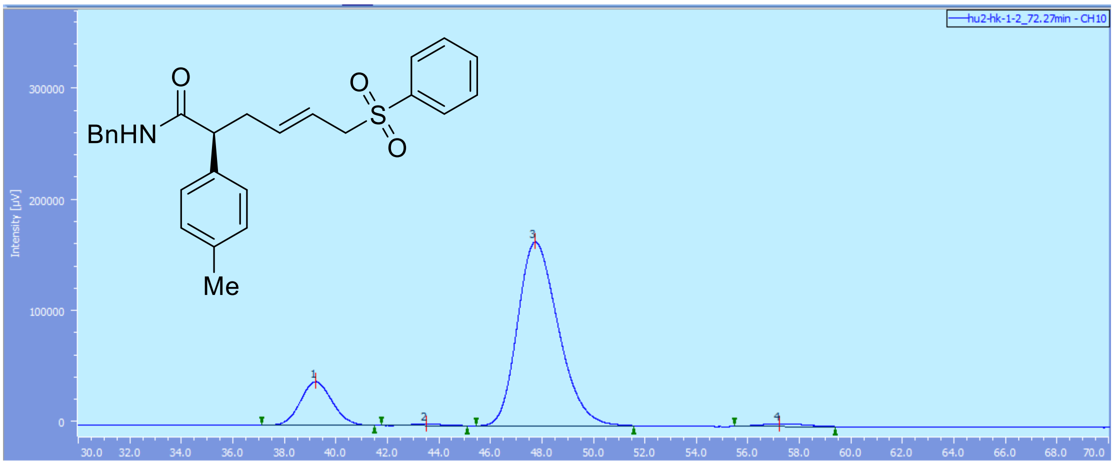

#	Peak Name	CH	tR	Area	Height	Area%	Height%	Quantity	NTP	Resolution	Symmetry Factor	Warning
1	Unknown	10	39.220	3366639	39305	15.334	18.855	N/A	4822	1.863	1.070	
2	Unknown	10	43.503	127842	1480	0.582	0.710	N/A	5476	1.619	1.123	
3	Unknown	10	47.733	18154773	165118	82.689	79.208	N/A	4371	3.076	1.253	
4	Unknown	10	57.177	306196	2557	1.395	1.227	N/A	4897	N/A	1.127	

(*S*,*E*)-*N*-Benzyl-6-((4-fluorophenyl)sulfonyl)-2-(*p*-tolyl)hex-4-enamide (2.18)

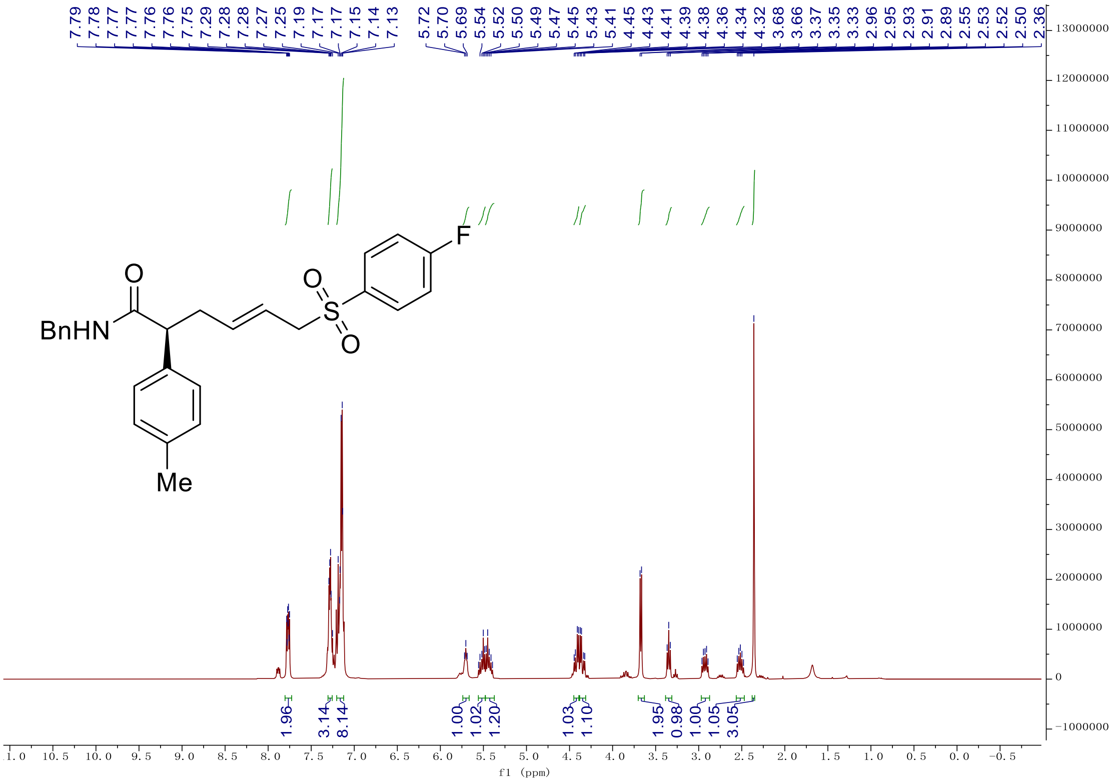

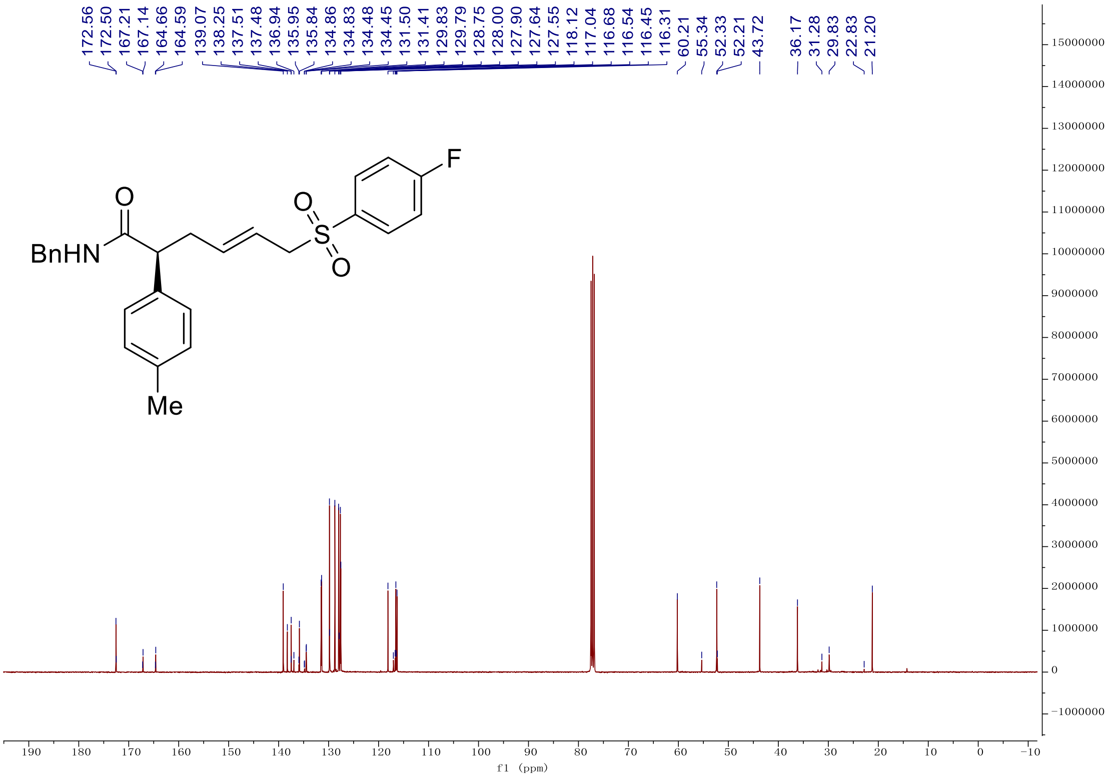

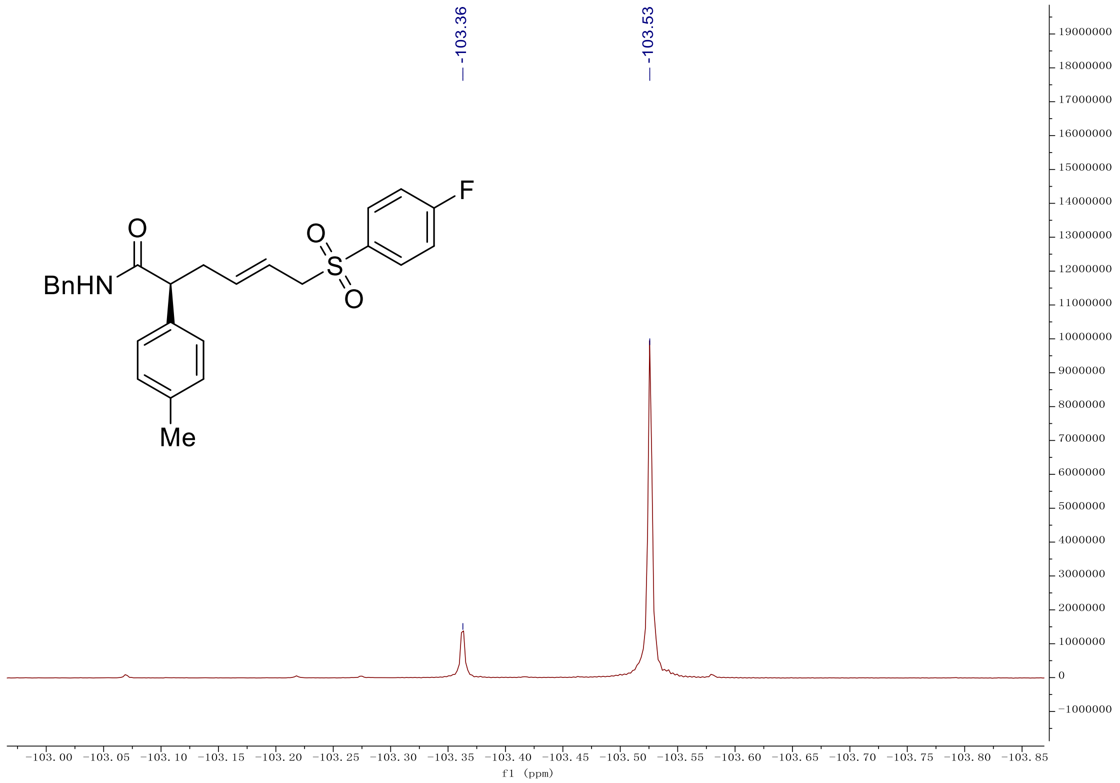

-103.36
-103.53
BnHN
O
O
S
O
F
Me
f1 (ppm)

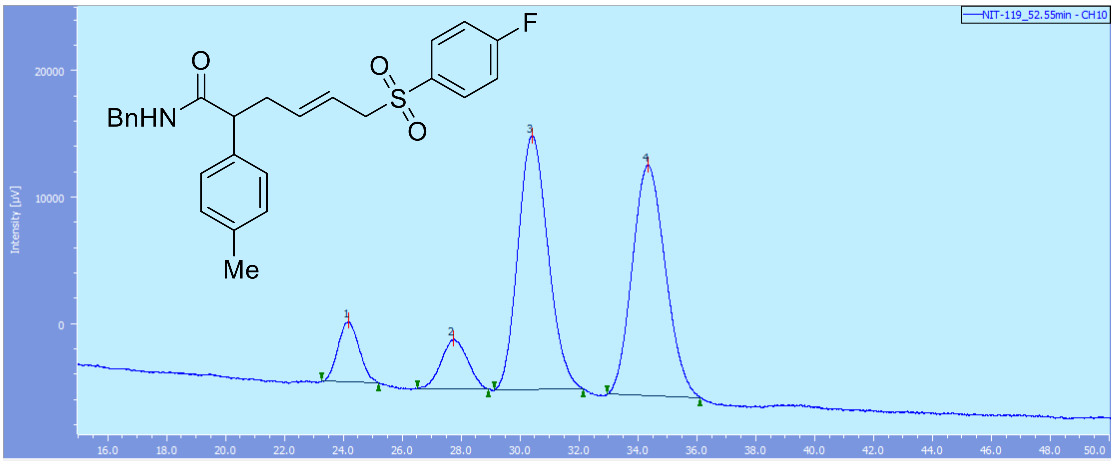

#	Peak Name	CH	tR	Area	Height	Area%	Height%	Quantity	NTP	Resolution	Symmetry Factor	Warning
1	Unknown	10	24.170	237098	4788	7.140	10.207	N/A	5234	2.360	1.071	
2	Unknown	10	27.730	245921	3926	7.405	8.370	N/A	4317	1.493	0.985	
3	Unknown	10	30.387	1416457	20007	42.654	42.650	N/A	4184	1.989	1.163	
4	Unknown	10	34.323	1421360	18189	42.801	38.773	N/A	4312	N/A	1.126	

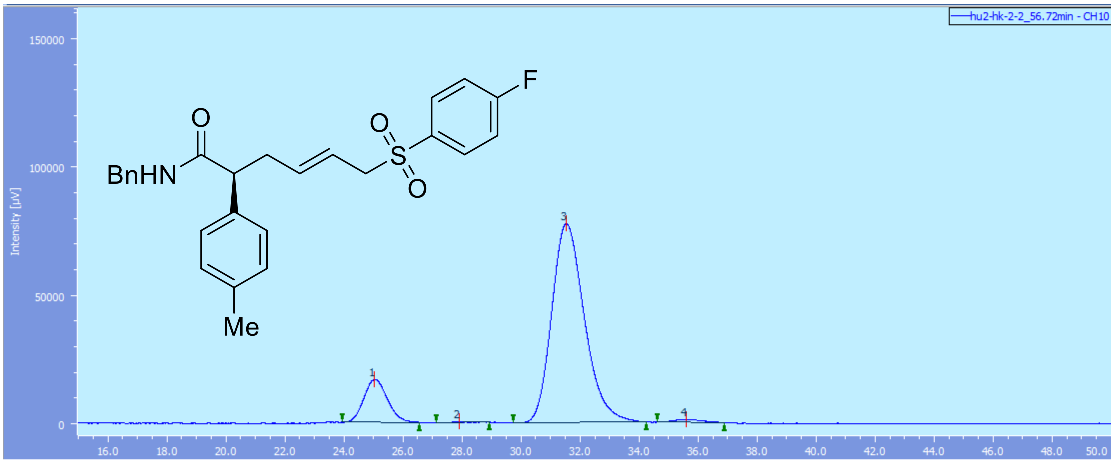

#	Peak Name	CH	tR	Area	Height	Area%	Height%	Quantity	NTP	Resolution	Symmetry Factor	Warning
1	Unknown	10	25.027	921444	16701	13.213	17.444	N/A	4670	2.866	1.157	
2	Unknown	10	27.900	14536	535	0.208	0.559	N/A	41796	2.860	1.039	
3	Unknown	10	31.530	5958771	77297	85.447	80.737	N/A	3980	2.133	1.239	
4	Unknown	10	35.583	78921	1205	1.132	1.259	N/A	6168	N/A	1.143	

(*S*,*E*)-*N*-Benzyl-2-(*p*-tolyl)-6-(*m*-tolylsulfonyl)hex-4-enamide (2.19)

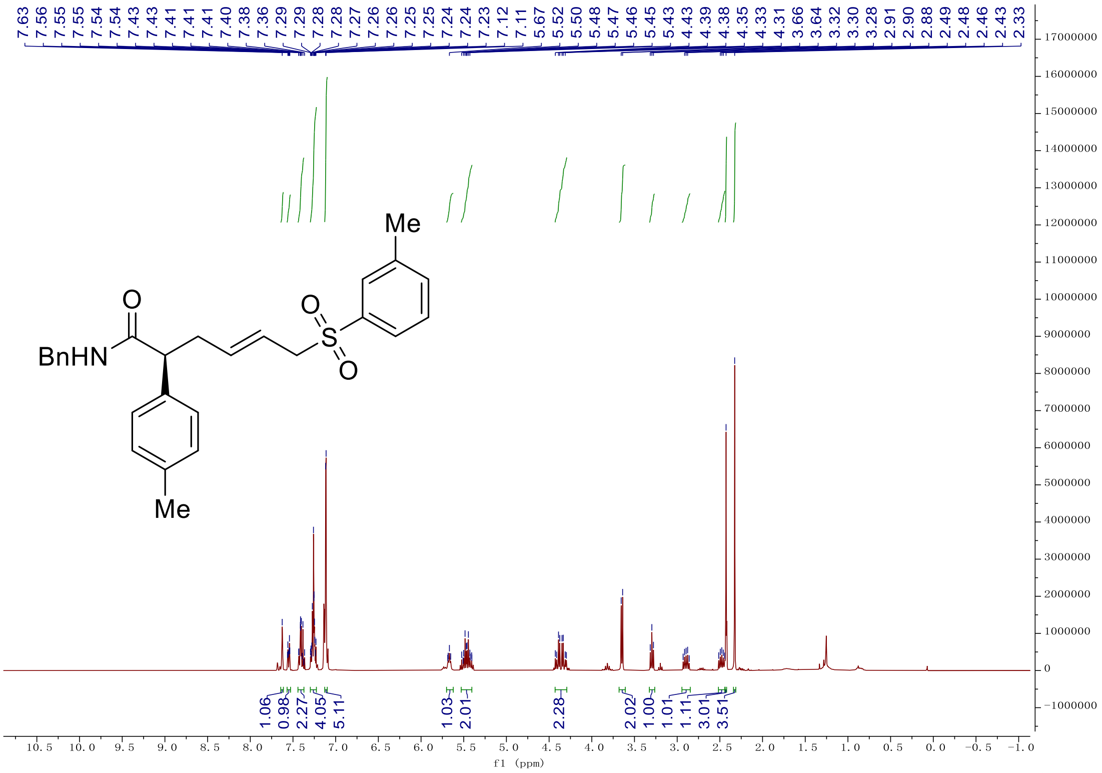

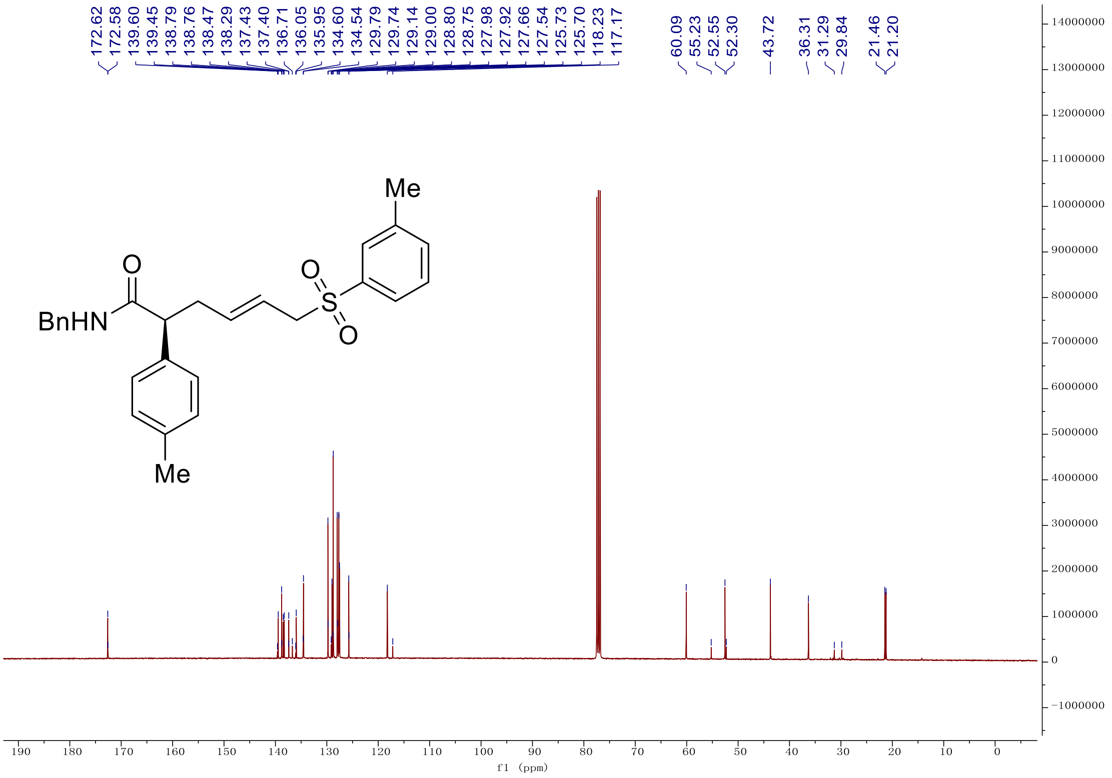

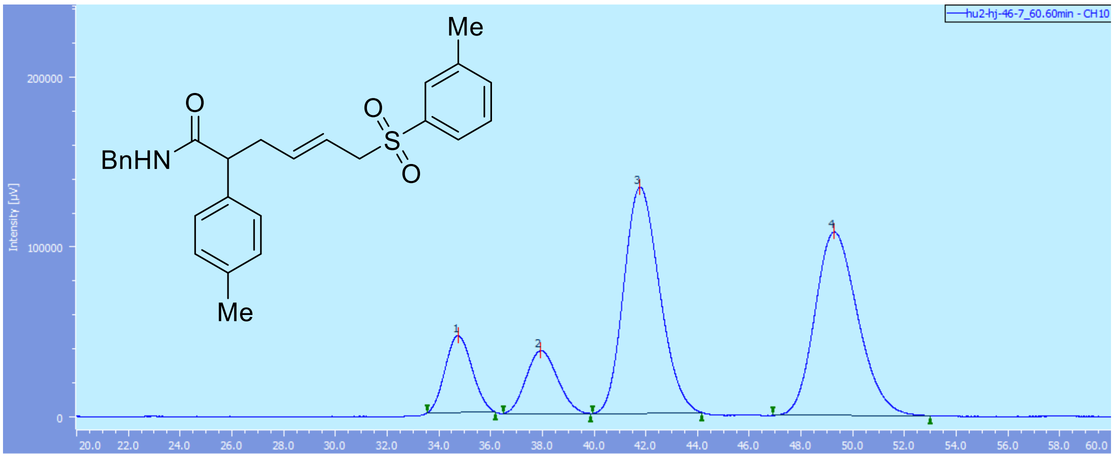

#	Peak Name	CH	tR	Area	Height	Area%	Height%	Quantity	NTP	Resolution	Symmetry Factor	Warning
1	Unknown	10	34.723	3272282	45183	10.379	14.014	N/A	4995	1.513	1.103	
2	Unknown	10	37.917	3103137	36914	9.842	11.450	N/A	4471	1.599	1.132	
3	Unknown	10	41.760	12728348	132608	40.370	41.130	N/A	4282	2.692	1.177	
4	Unknown	10	49.257	12425500	107703	39.409	33.406	N/A	4223	N/A	1.200	

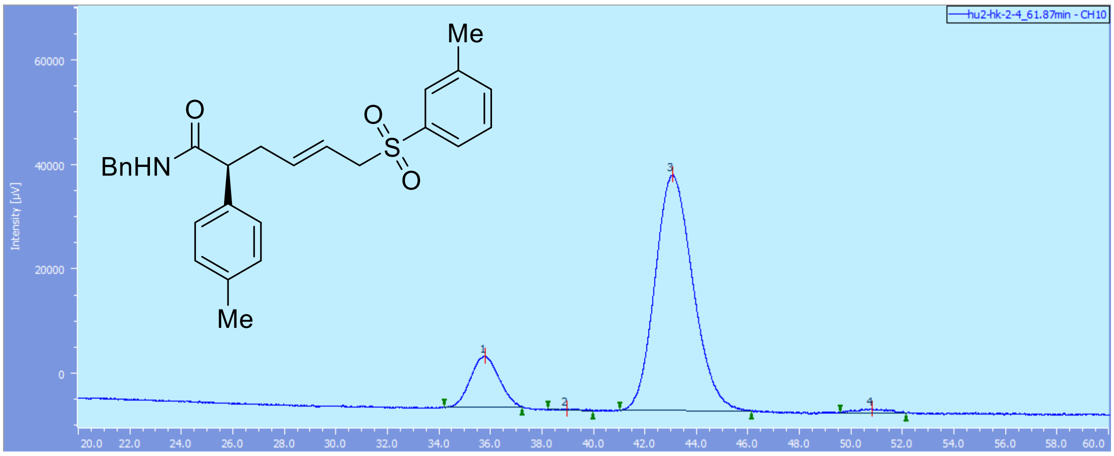

#	Peak Name	CH	tR	Area	Height	Area%	Height%	Quantity	NTP	Resolution	Symmetry Factor	Warning
1	Unknown	10	35.783	744915	9761	13.762	17.513	N/A	4763	1.633	1.034	
2	Unknown	10	38.970	6752	185	0.125	0.333	N/A	7177	1.803	1.380	
3	Unknown	10	43.040	4589714	44960	84.790	80.665	N/A	4109	2.740	1.161	
4	Unknown	10	50.780	71627	830	1.323	1.489	N/A	4649	N/A	1.045	

(*S*,*E*)-*N*-Benzyl-2-(*p*-tolyl)-6-(*o*-tolylsulfonyl)hex-4-enamide (2.20)

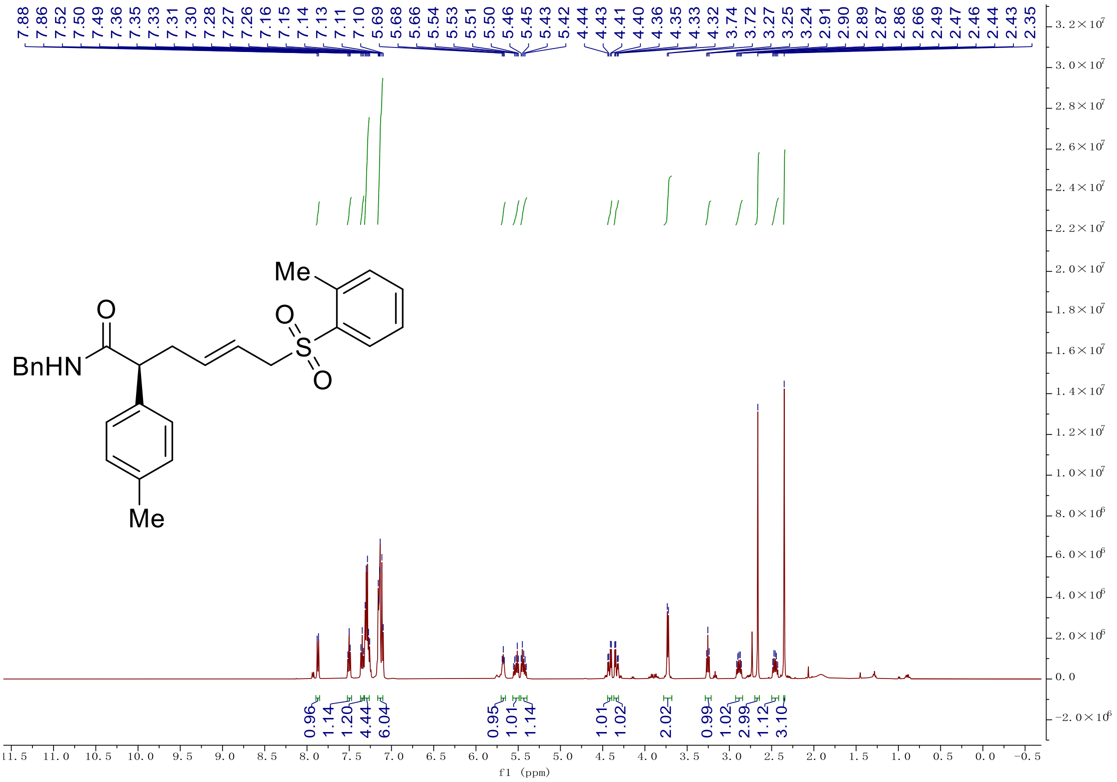

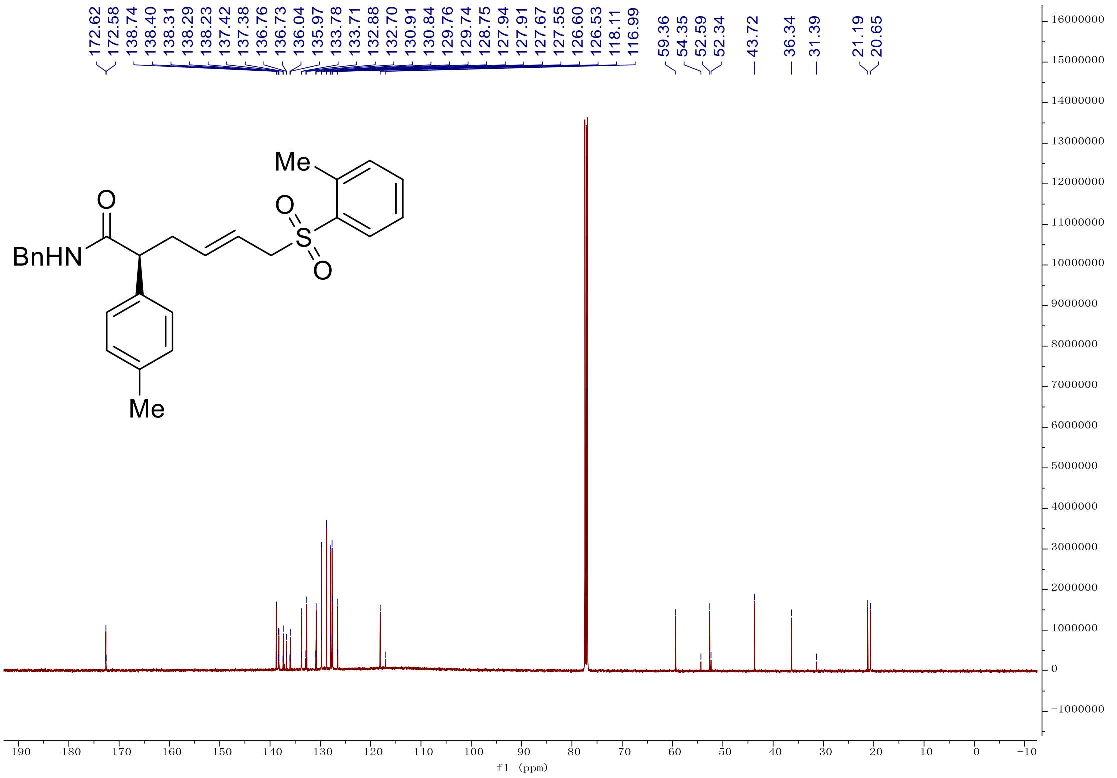

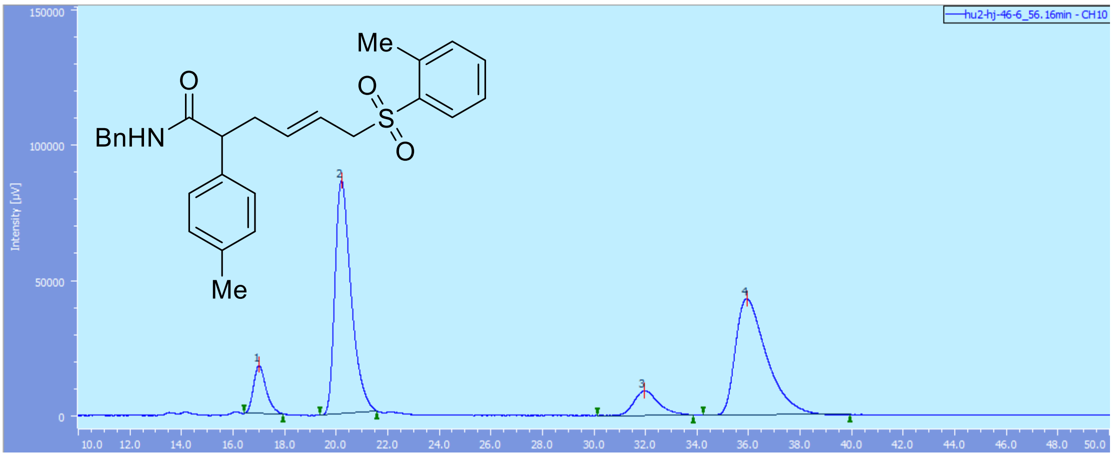

#	Peak Name	CH	tR	Area	Height	Area%	Height%	Quantity	NTP	Resolution	Symmetry Factor	Warning
1	Unknown	10	17.010	608833	17809	7.115	11.439	N/A	5990	3.204	1.307	
2	Unknown	10	20.197	3744888	85926	43.767	55.193	N/A	5249	8.479	1.491	
3	Unknown	10	31.943	594321	9050	6.946	5.813	N/A	5903	2.088	1.316	
4	Unknown	10	35.913	3608435	42897	42.172	27.555	N/A	4462	N/A	1.692	

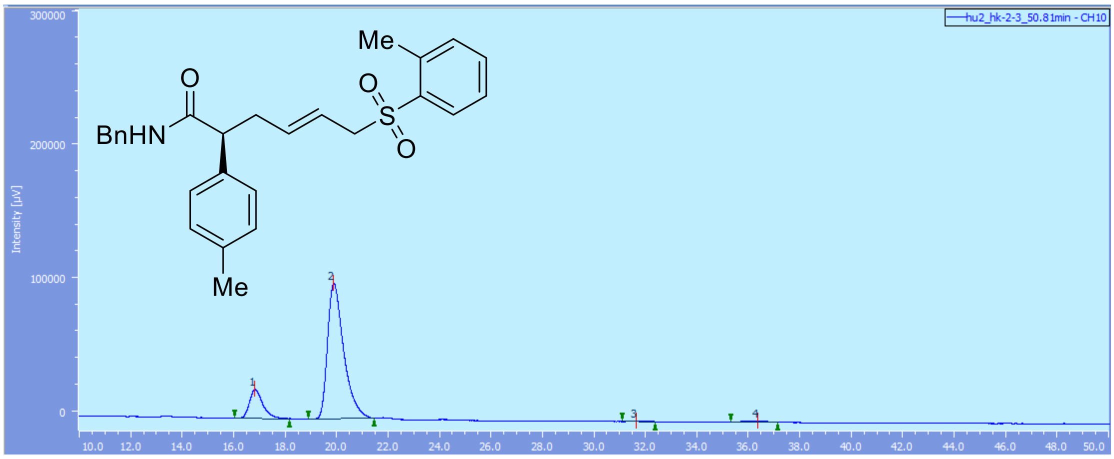

#	Peak Name	CH	tR	Area	Height	Area%	Height%	Quantity	NTP	Resolution	Symmetry Factor	Warning
1	Unknown	10	16.827	763725	21475	14.524	17.319	N/A	5609	3.056	1.380	
2	Unknown	10	19.883	4422513	101105	84.105	81.538	N/A	5159	9.437	1.545	
3	Unknown	10	31.627	11792	337	0.224	0.272	N/A	8307	3.015	1.160	
4	Unknown	10	36.347	60284	1080	1.146	0.871	N/A	6893	N/A	0.897	

(*S*,*E*)-*N*-Benzyl-6-(thiophen-2-ylsulfonyl)-2-(*p*-tolyl)hex-4-enamide (2.21)

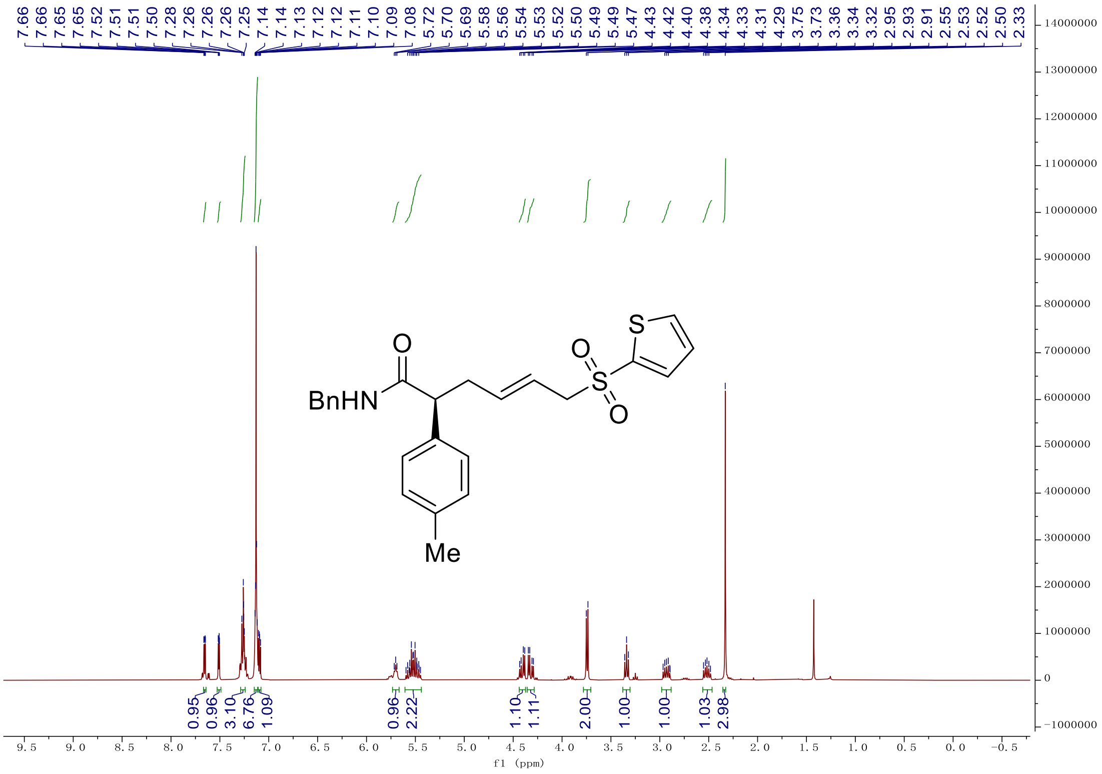

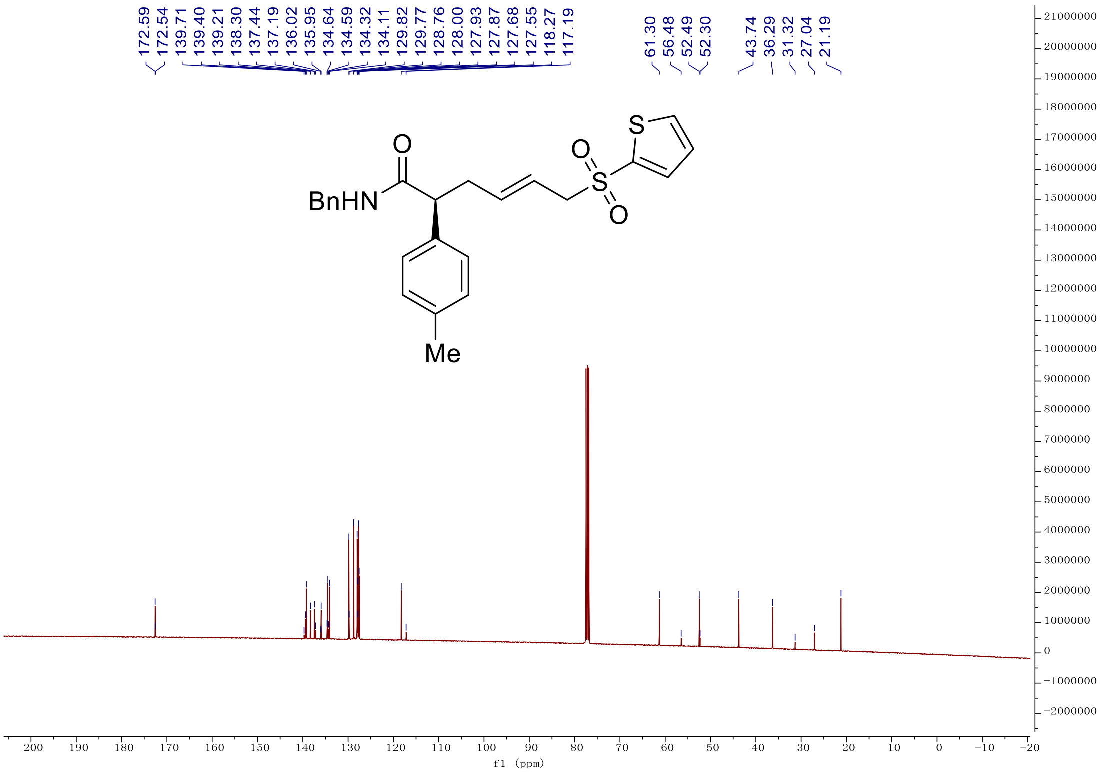

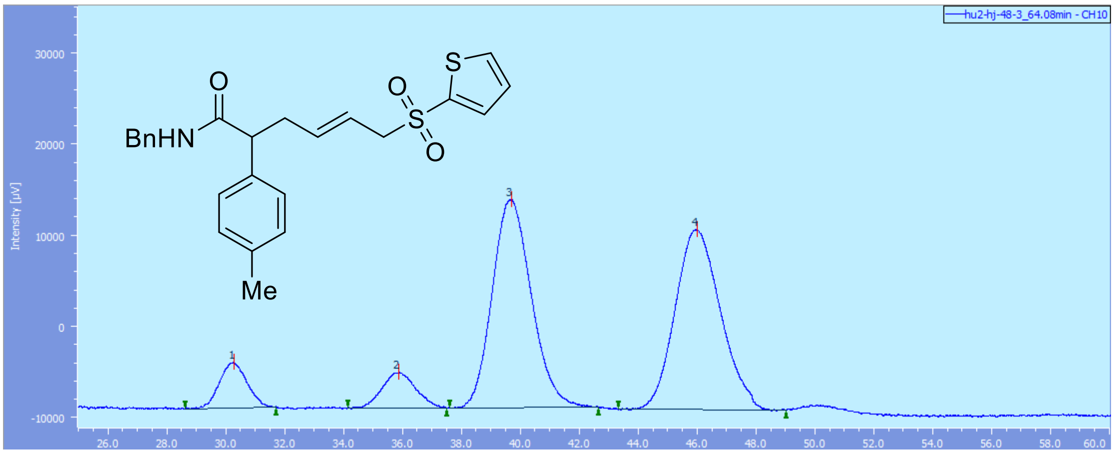

#	Peak Name	CH	tR	Area	Height	Area%	Height%	Quantity	NTP	Resolution	Symmetry Factor	Warning
1	Unknown	10	30.270	323930	4987	6.668	9.702	N/A	4834	2.912	1.044	
2	Unknown	10	35.860	306275	3901	6.304	7.590	N/A	4631	1.696	1.100	
3	Unknown	10	39.697	2105483	22788	43.339	44.336	N/A	4271	2.394	1.124	
4	Unknown	10	45.987	2122488	19722	43.689	38.372	N/A	4199	N/A	1.093	

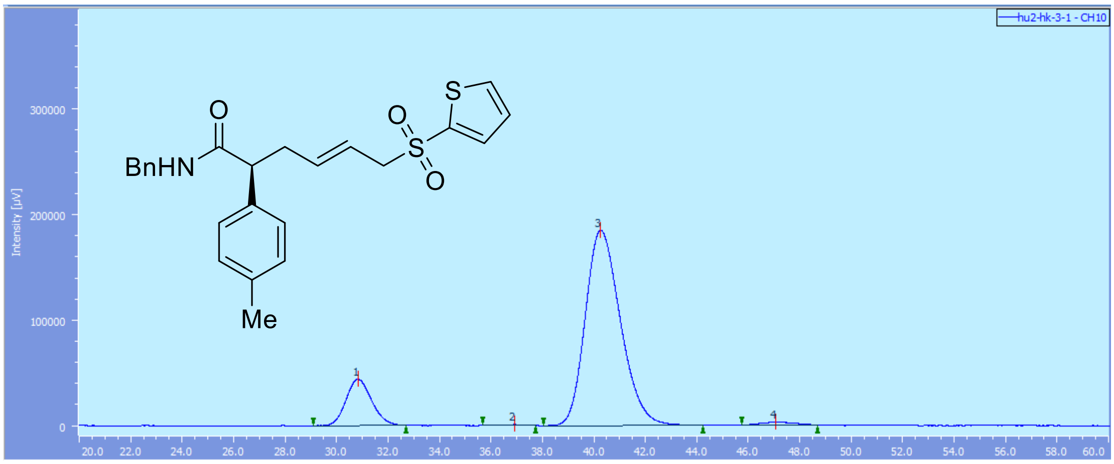

#	Peak Name	CH	tR	Area	Height	Area%	Height%	Quantity	NTP	Resolution	Symmetry Factor	Warning
1	Unknown	10	30.833	3020225	43826	14.504	18.885	N/A	4639	3.314	1.133	
2	Unknown	10	36.910	53303	929	0.256	0.400	N/A	6255	1.536	0.782	
3	Unknown	10	40.243	17491296	184574	83.999	79.534	N/A	4199	2.622	1.245	
4	Unknown	10	47.023	258377	2740	1.241	1.181	N/A	4852	N/A	1.164	

(*S*,*E*)-*N*-Benzyl-6-(((*E*)-styryl)sulfonyl)-2-(*p*-tolyl)hex-4-enamide (2.22)

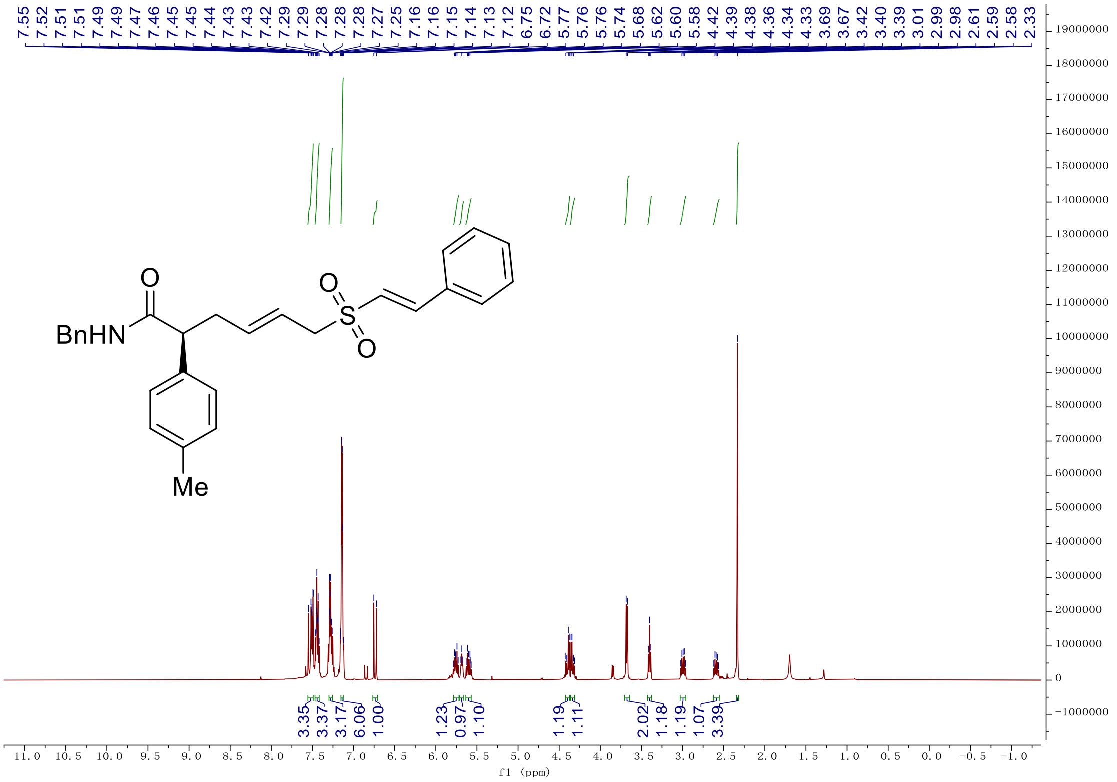

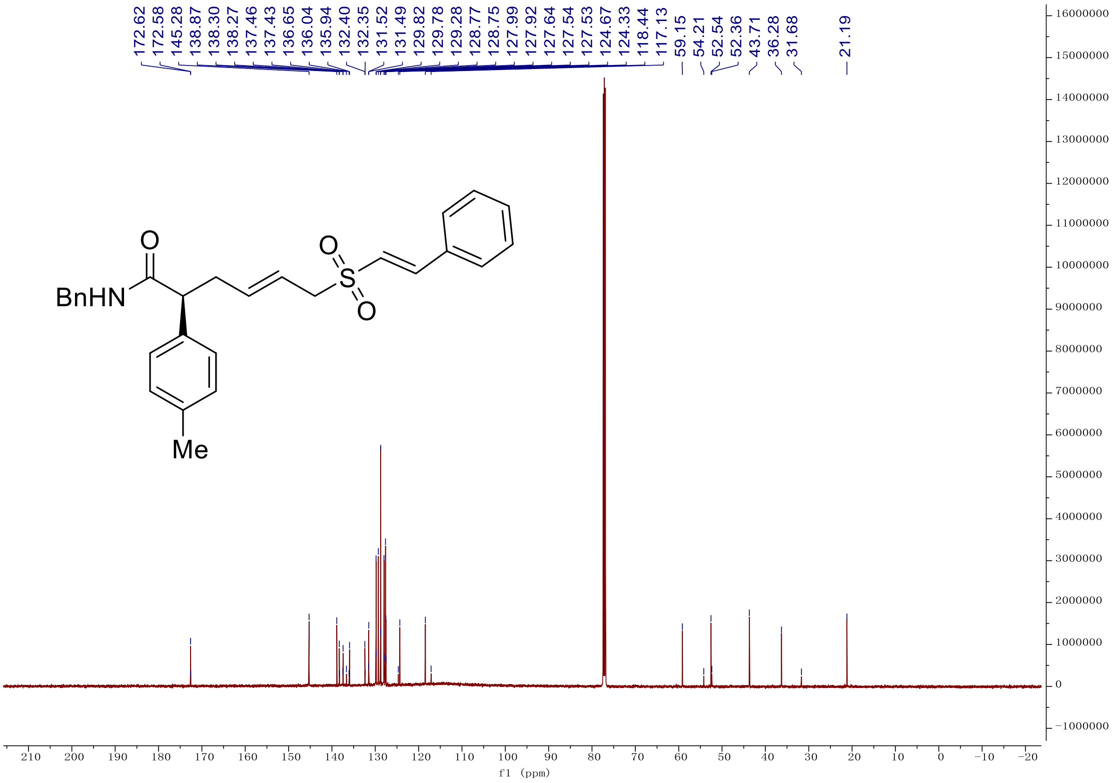

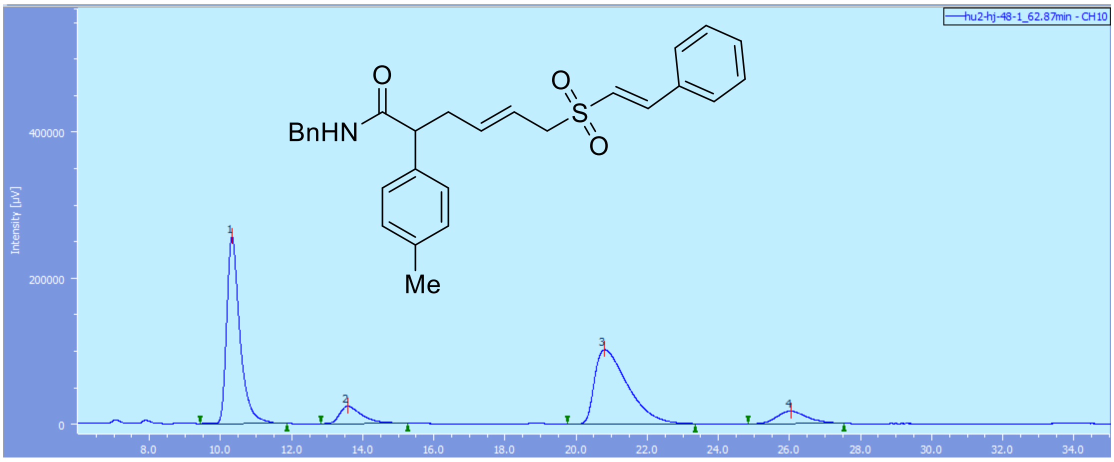

#	Peak Name	CH	tR	Area	Height	Area%	Height%	Quantity	NTP	Resolution	Symmetry Factor	Warning
1	Unknown	10	10.317	6566795	254984	43.445	64.230	N/A	4233	3.790	1.526	
2	Unknown	10	13.573	1027191	23645	6.796	5.956	N/A	2487	5.225	1.662	
3	Unknown	10	20.793	6513378	101393	43.091	25.541	N/A	2445	3.263	2.049	
4	Unknown	10	26.037	1007966	16967	6.669	4.274	N/A	4573	N/A	1.224	

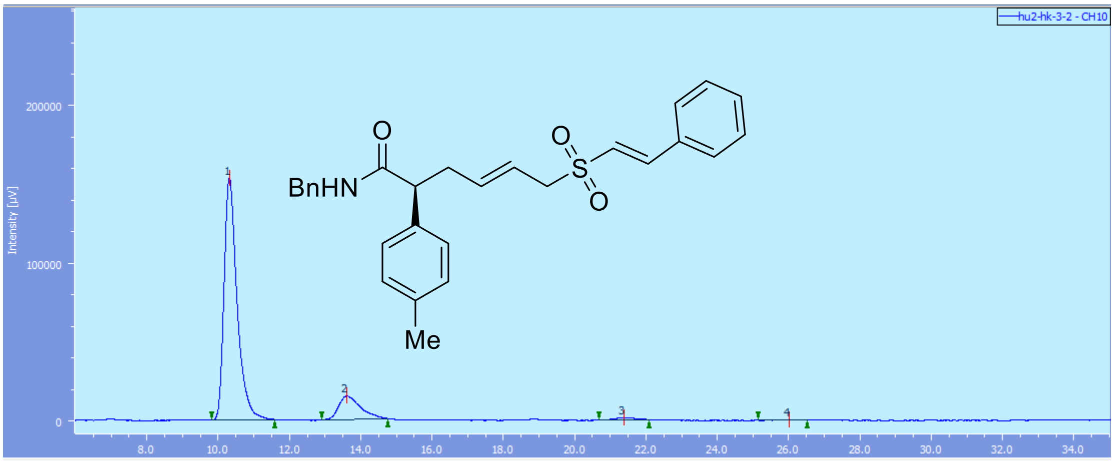

#	Peak Name	CH	tR	Area	Height	Area%	Height%	Quantity	NTP	Resolution	Symmetry Factor	Warning
1	Unknown	10	10.310	3889280	153024	84.700	90.226	N/A	4269	3.828	1.477	
2	Unknown	10	13.610	635730	14790	13.845	8.720	N/A	2460	6.827	1.454	
3	Unknown	10	21.367	57177	1405	1.245	0.828	N/A	5239	4.242	1.130	
4	Unknown	10	26.003	9660	383	0.210	0.226	N/A	10577	N/A	0.855	

(*S*,*E*)-*N*-Benzyl-6-(cyclopropylsulfonyl)-2-(*p*-tolyl)hex-4-enamide (2.23)

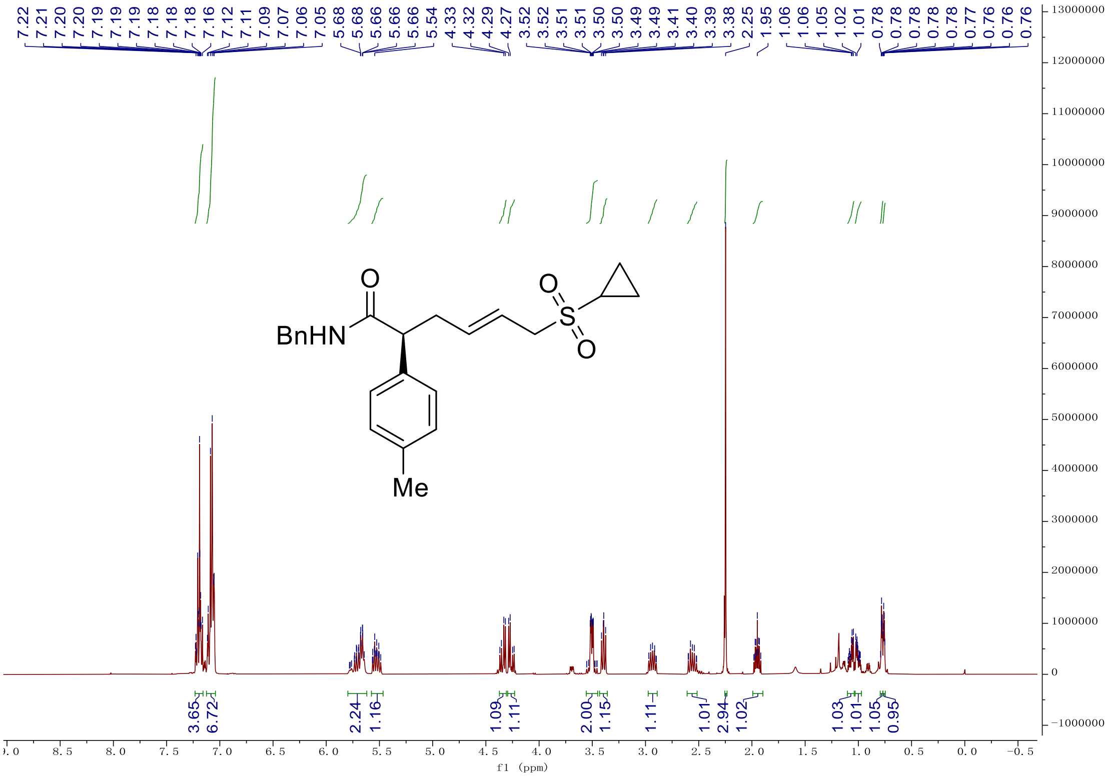

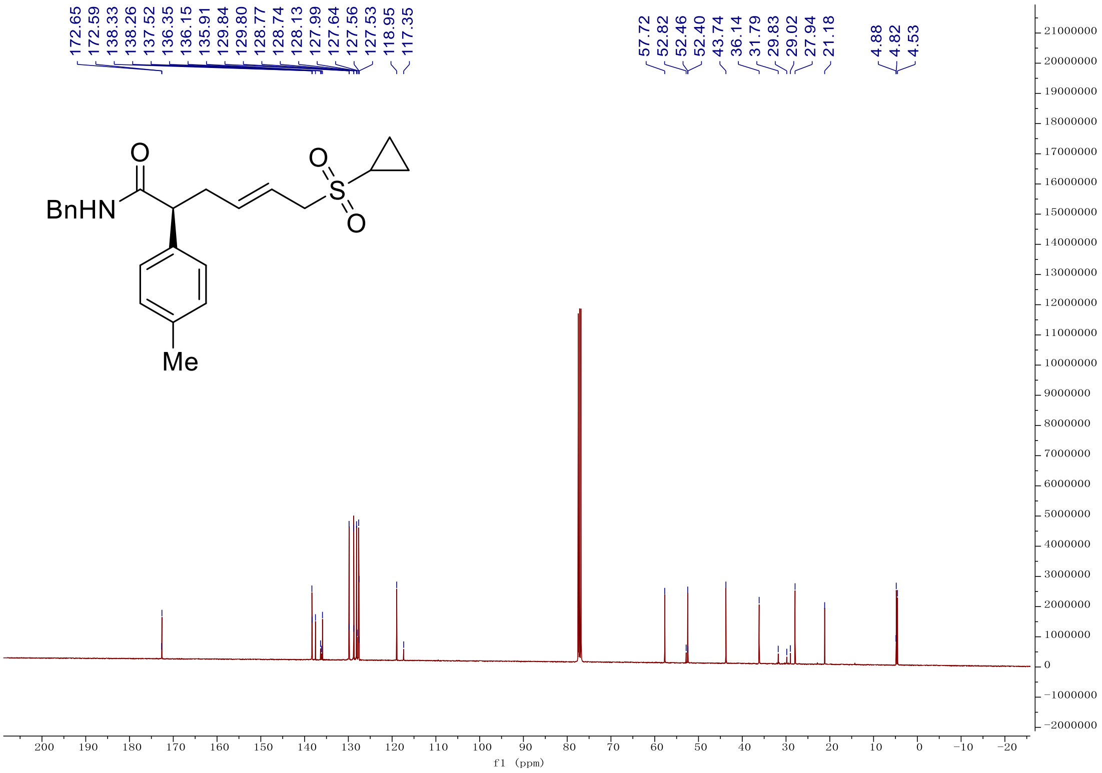

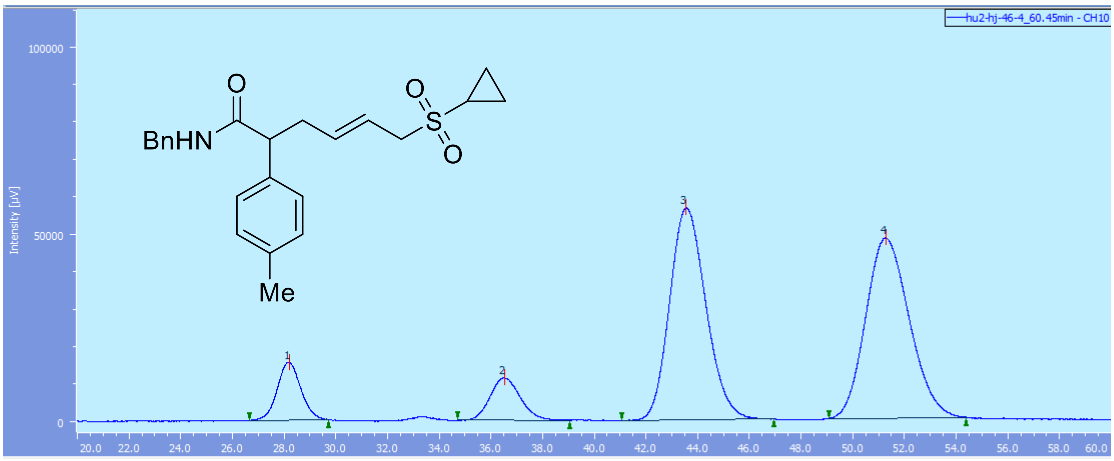

#	Peak Name	CH	tR	Area	Height	Area%	Height%	Quantity	NTP	Resolution	Symmetry Factor	Warning
1	Unknown	10	28.190	984337	15486	7.533	11.783	N/A	4697	4.419	1.047	
2	Unknown	10	36.500	913677	11306	6.992	8.603	N/A	4718	3.002	1.138	
3	Unknown	10	43.530	5532613	56575	42.342	43.049	N/A	4587	2.721	1.170	
4	Unknown	10	51.240	5635958	48053	43.133	36.564	N/A	4340	N/A	1.161	

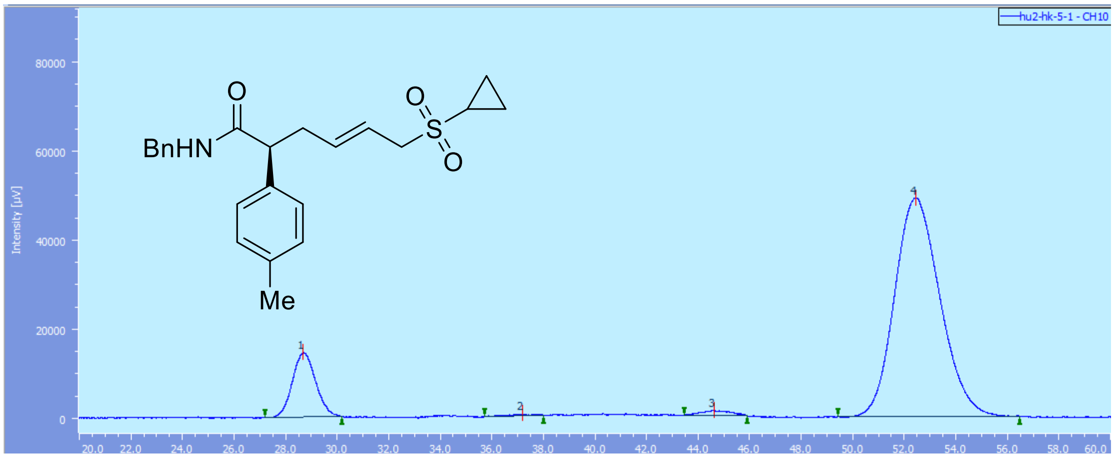

#	Peak Name	CH	tR	Area	Height	Area%	Height%	Quantity	NTP	Resolution	Symmetry Factor	Warning
1	Unknown	10	28.673	895963	14340	12.854	22.153	N/A	4733	4.567	1.112	
2	Unknown	10	37.177	16429	381	0.236	0.589	N/A	5178	3.338	0.800	
3	Unknown	10	44.587	88157	1111	1.265	1.716	N/A	5595	2.798	1.060	
4	Unknown	10	52.407	5969991	48902	85.646	75.543	N/A	4240	N/A	1.158	

(*S*,*E*)-*N*-Benzyl-6-(propylsulfonyl)-2-(*p*-tolyl)hex-4-enamide (2.24)

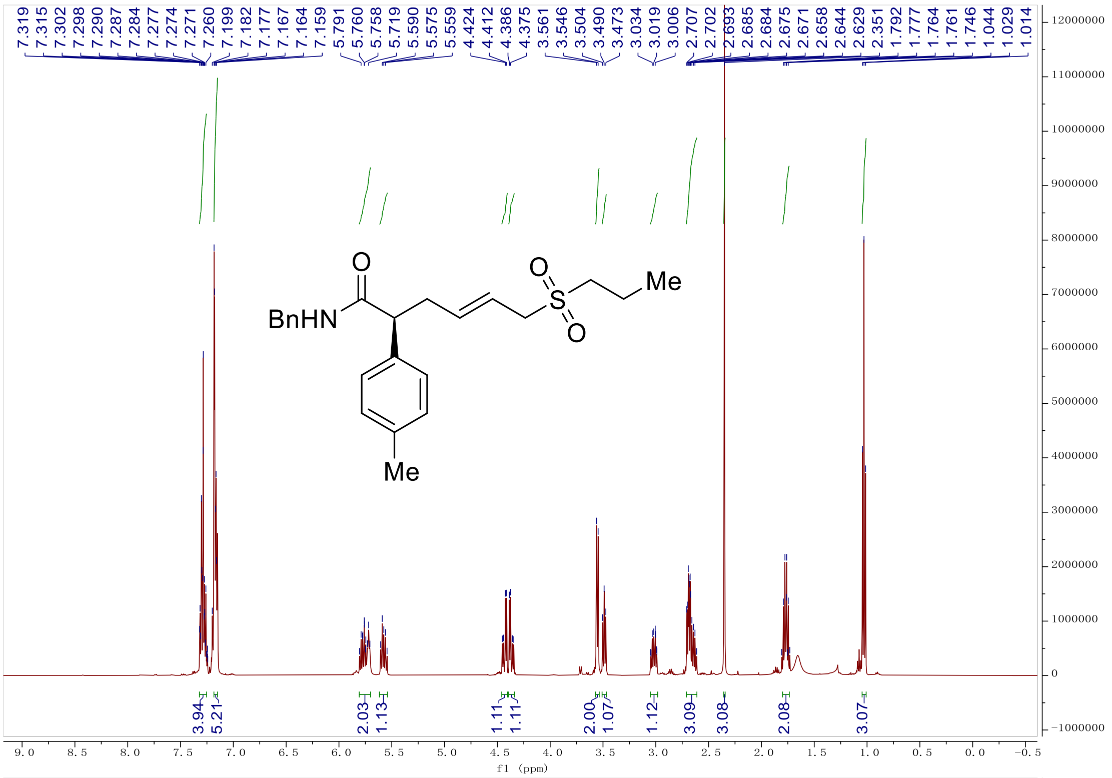

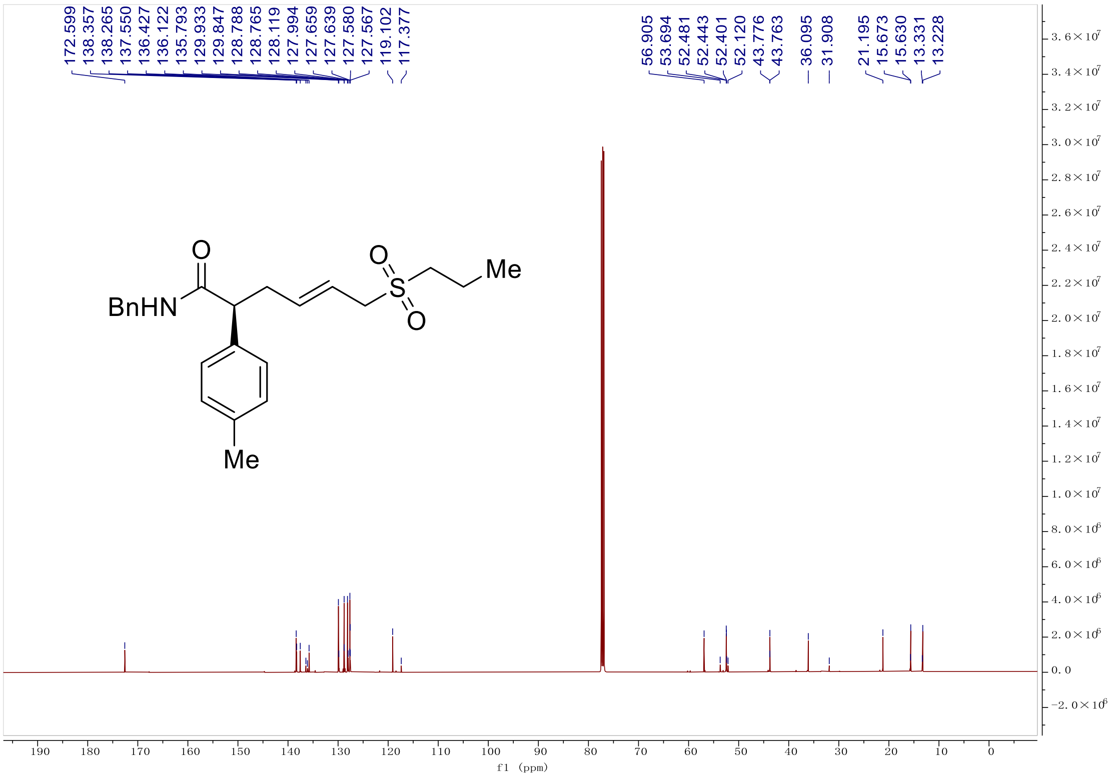

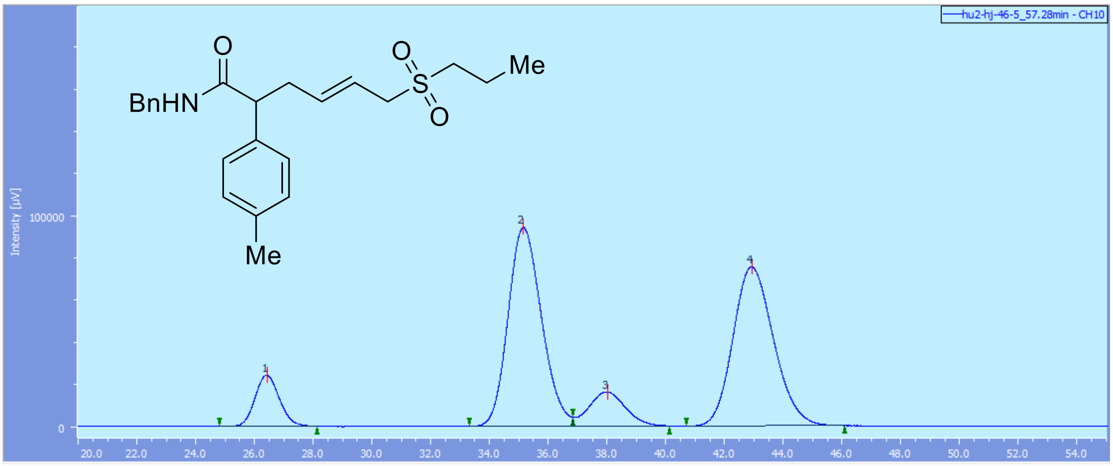

#	Peak Name	CH	tR	Area	Height	Area%	Height%	Quantity	NTP	Resolution	Symmetry Factor	Warning
1	Unknown	10	26.423	1373644	24012	7.878	11.464	N/A	4871	4.846	1.101	
2	Unknown	10	35.133	7450111	94012	42.725	44.883	N/A	4522	1.316	1.213	
3	Unknown	10	37.997	1390227	16174	7.973	7.722	N/A	4469	2.051	N/A	
4	Unknown	10	42.907	7223244	75261	41.424	35.931	N/A	4609	N/A	1.160	

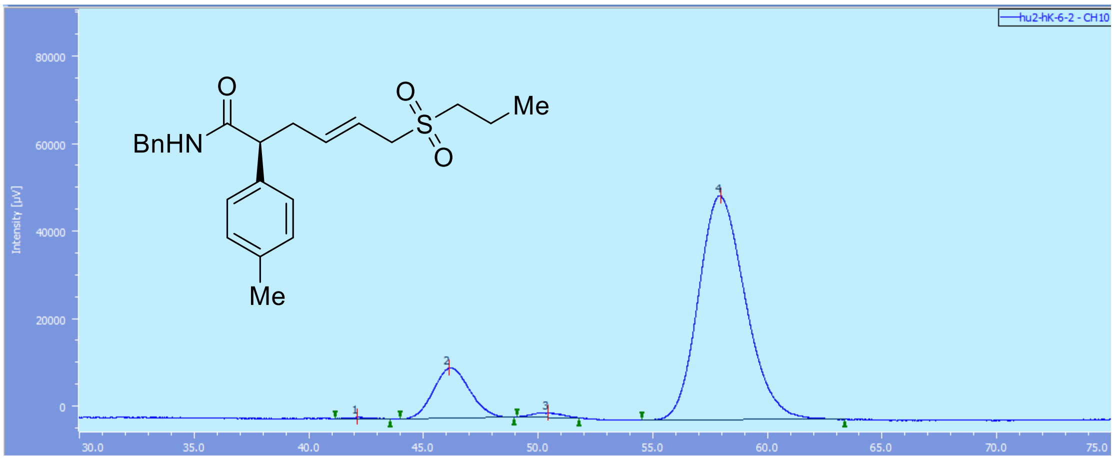

#	Peak Name	CH	tR	Area	Height	Area%	Height%	Quantity	NTP	Resolution	Symmetry Factor	Warning
1	Unknown	10	42.117	18679	339	0.217	0.530	N/A	7242	1.643	1.117	
2	Unknown	10	46.120	1250099	11496	14.514	17.981	N/A	4032	1.538	1.155	
3	Unknown	10	50.397	89565	1006	1.040	1.574	N/A	5692	2.362	0.999	
4	Unknown	10	57.927	7254576	51097	84.229	79.915	N/A	3879	N/A	1.169	

Ethyl (*S*,*E*)-8-(benzylamino)-2,2-difluoro-8-oxo-7-(*p*-tolyl)oct-4-enoate (2.25)

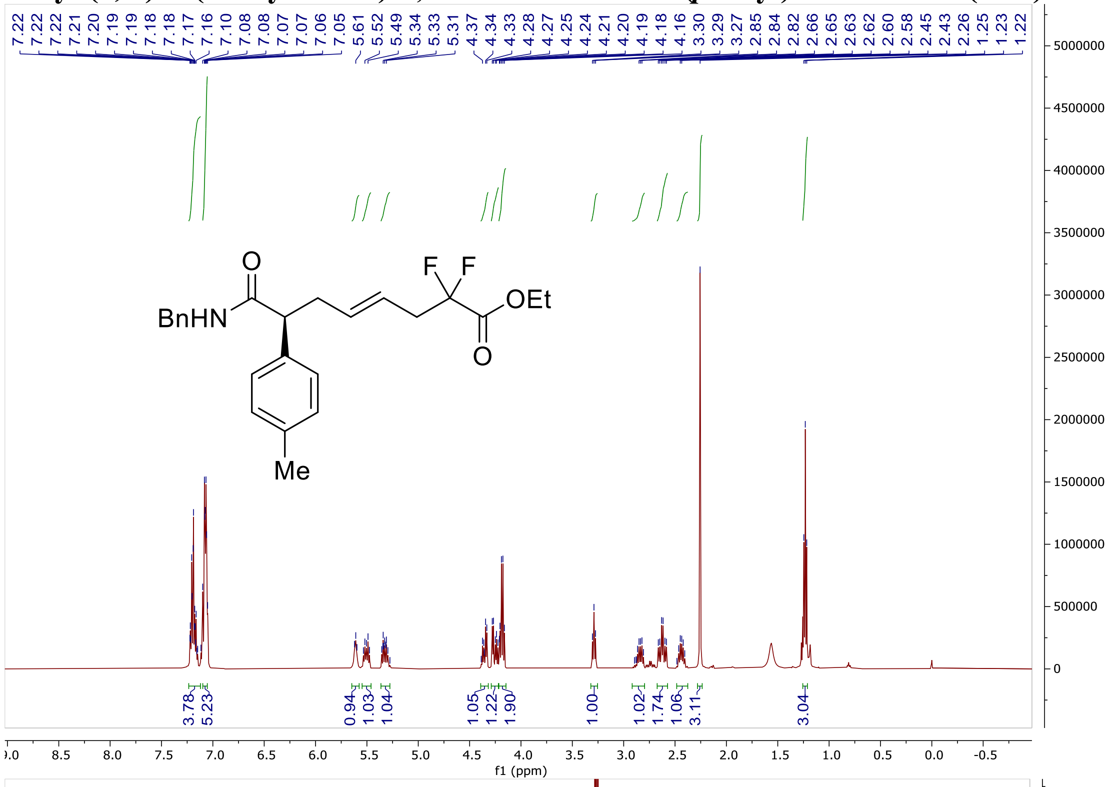

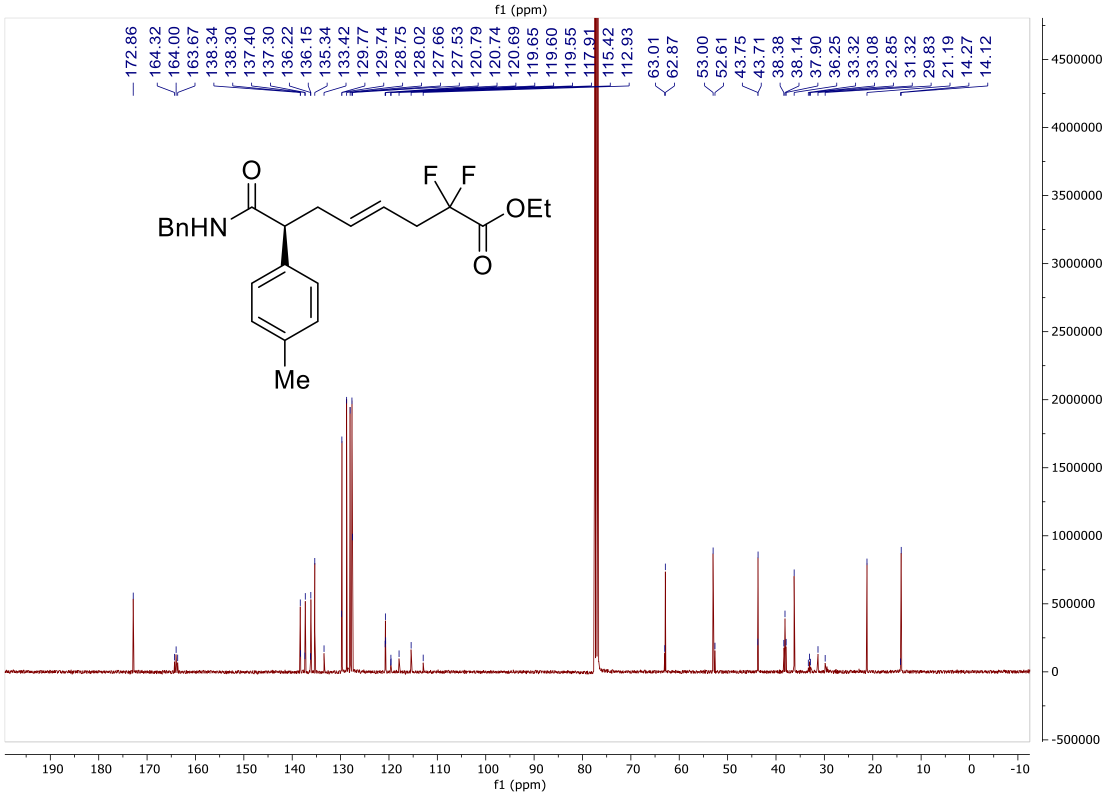

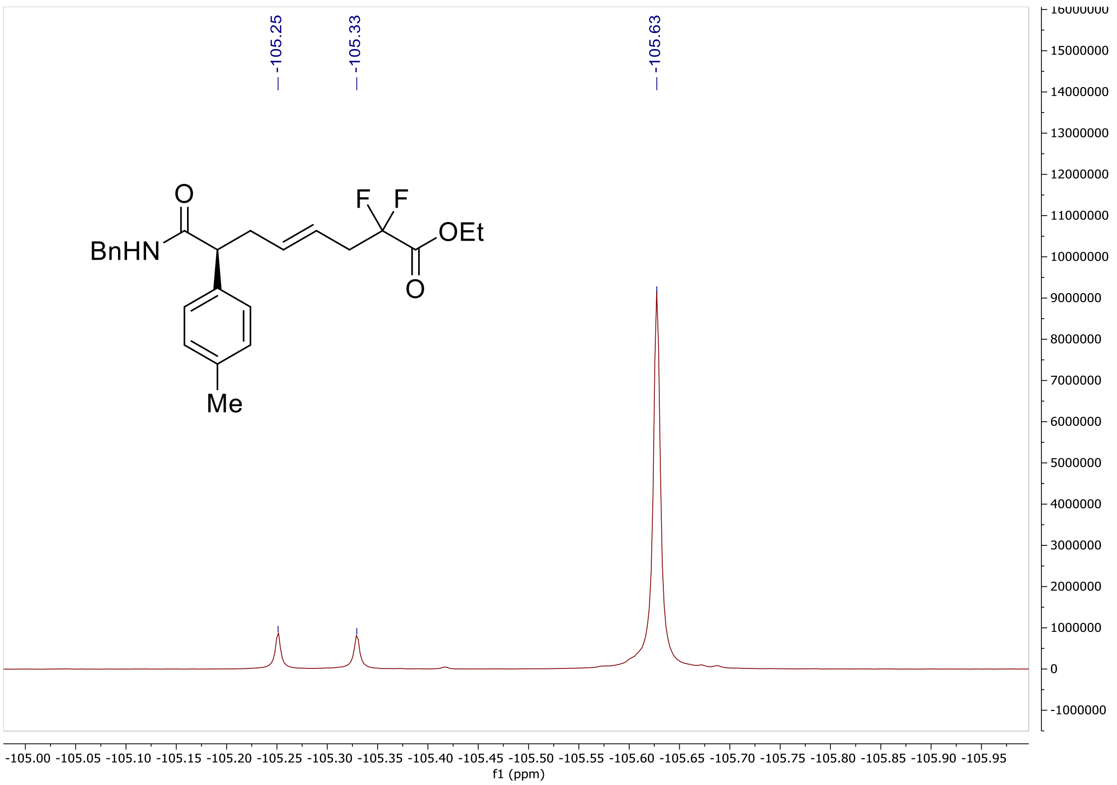

-105.25
-105.33
-105.63
BnHN
O
F
F
OEt
O
Me
16000000
15000000
14000000
13000000
12000000
11000000
10000000
9000000
8000000
7000000
6000000
5000000
4000000
3000000
2000000
1000000
0
-1000000
-105.00 -105.05 -105.10 -105.15 -105.20 -105.25 -105.30 -105.35 -105.40 -105.45 -105.50 -105.55 -105.60 -105.65 -105.70 -105.75 -105.80 -105.85 -105.90 -105.95
f1 (ppm)

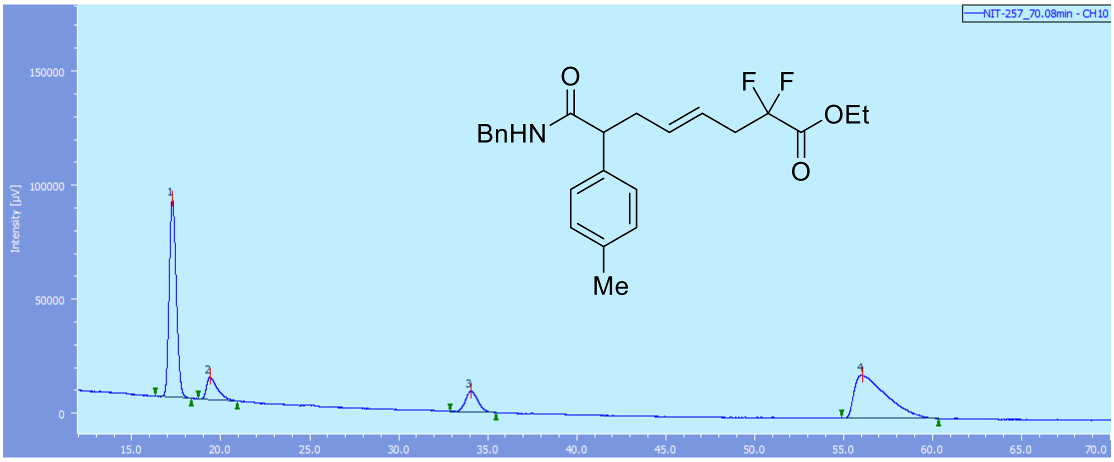

#	Peak Name	CH	tR	Area	Height	Area%	Height%	Quantity	NTP	Resolution	Symmetry Factor	Warning
1	Unknown	10	17.280	2386977	86630	42.651	69.743	N/A	8921	2.343	1.174	
2	Unknown	10	19.403	419584	9763	7.497	7.860	N/A	5111	12.382	1.846	
3	Unknown	10	34.023	439398	9050	7.851	7.286	N/A	11265	9.542	1.214	
4	Unknown	10	56.027	2350633	18769	42.001	15.110	N/A	4497	N/A	2.559	

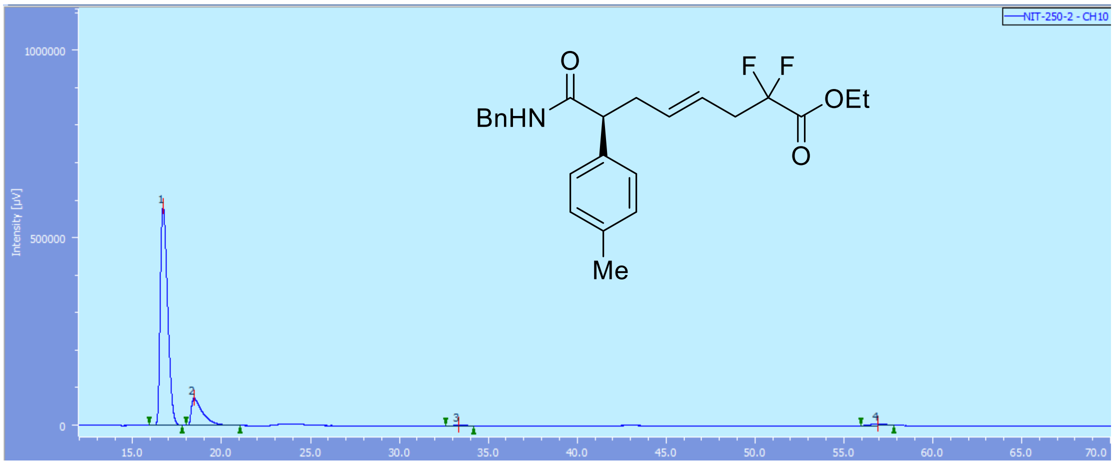

#	Peak Name	CH	tR	Area	Height	Area%	Height%	Quantity	NTP	Resolution	Symmetry Factor	Warning
1	Unknown	10	16.707	16672106	581357	82.504	88.224	N/A	7540	1.891	1.489	
2	Unknown	10	18.457	3234311	72157	16.005	10.950	N/A	4624	12.957	2.998	
3	Unknown	10	33.317	81291	1883	0.402	0.286	N/A	12047	15.738	1.019	
4	Unknown	10	56.887	219945	3556	1.088	0.540	N/A	16175	N/A	0.989	

Cyclohexyl (*S*,*E*)-8-(benzylamino)-2,2-difluoro-8-oxo-7-(*p*-tolyl)oct-4-enoate (2.26)

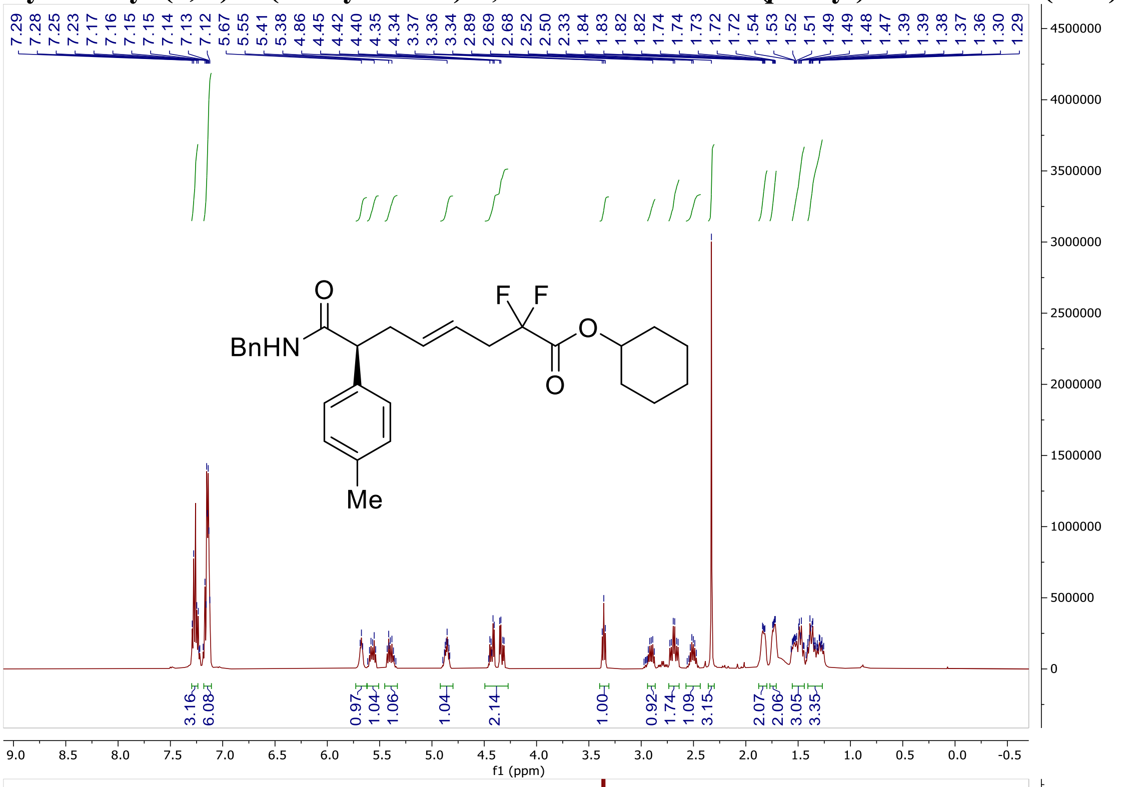

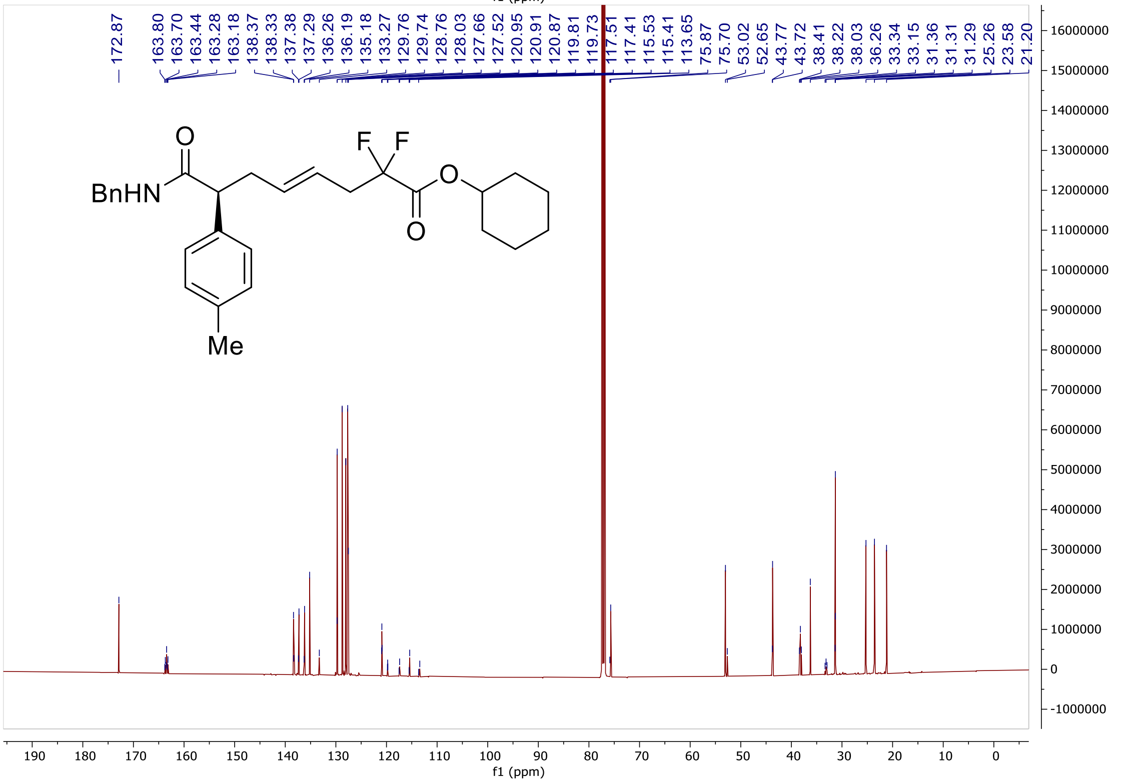

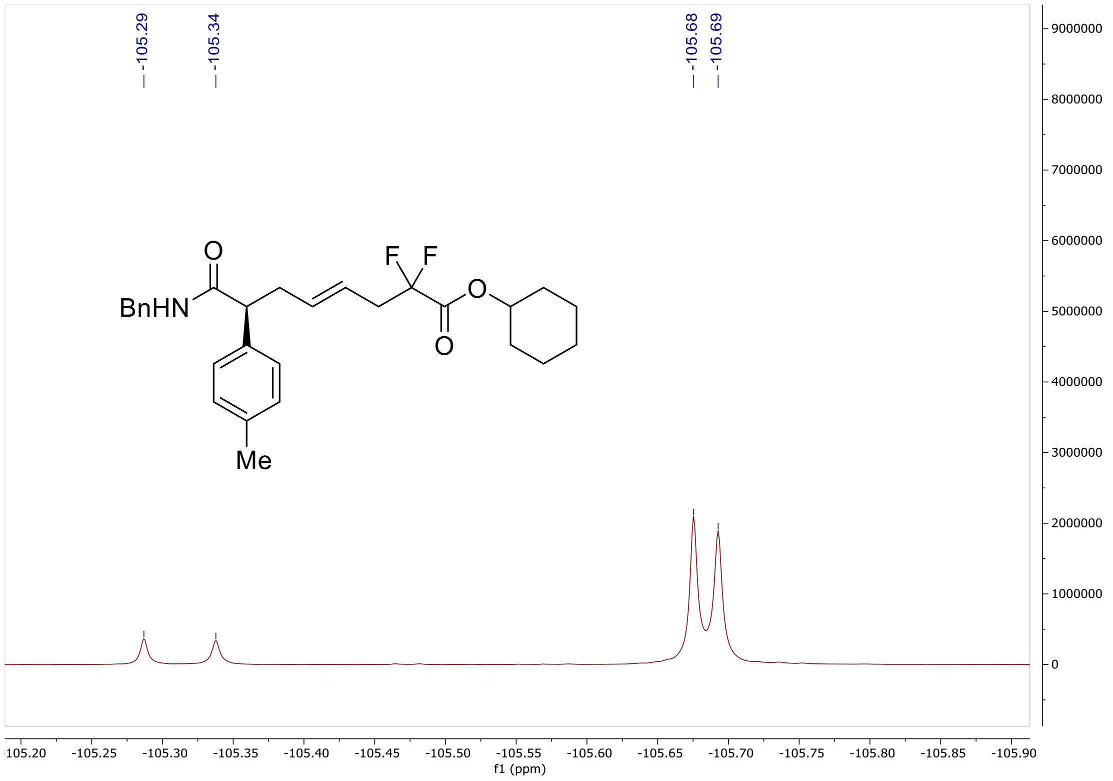
-105.29
-105.34
-105.68
-105.69
BnHN
O
F
F
O
O
Me
9000000
8000000
7000000
6000000
5000000
4000000
3000000
2000000
1000000
0
105.20
-105.25
-105.30
-105.35
-105.40
-105.45
-105.50
-105.55
-105.60
-105.65
-105.70
-105.75
-105.80
-105.85
-105.90
f1 (ppm)

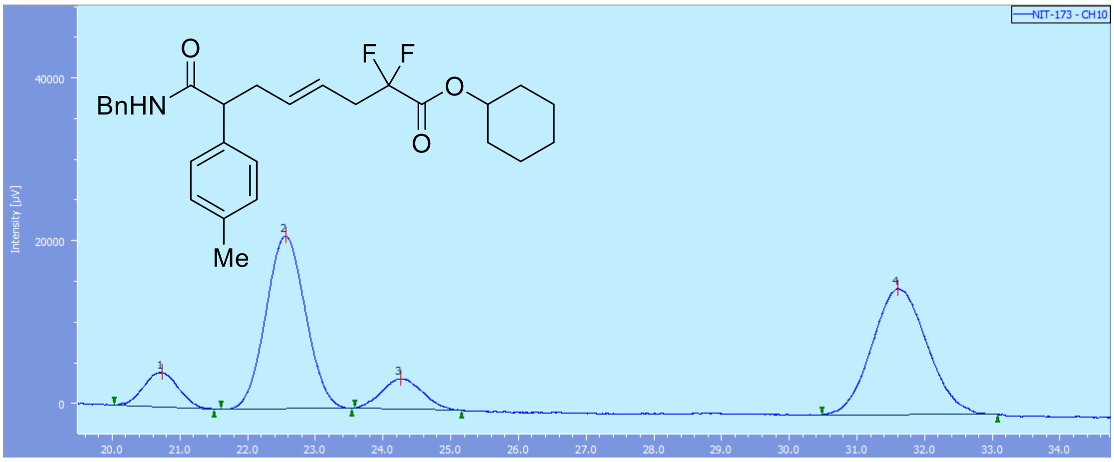

#	Peak Name	CH	tR	Area	Height	Area%	Height%	Quantity	NTP	Resolution	Symmetry Factor	Warning
1	Unknown	10	20.740	150751	4204	7.411	9.441	N/A	7363	1.778	0.950	
2	Unknown	10	22.557	858453	21166	42.200	47.529	N/A	6949	1.541	1.045	
3	Unknown	10	24.260	156073	3724	7.672	8.362	N/A	7312	5.578	1.086	
4	Unknown	10	31.593	868953	15438	42.717	34.668	N/A	7082	N/A	1.107	

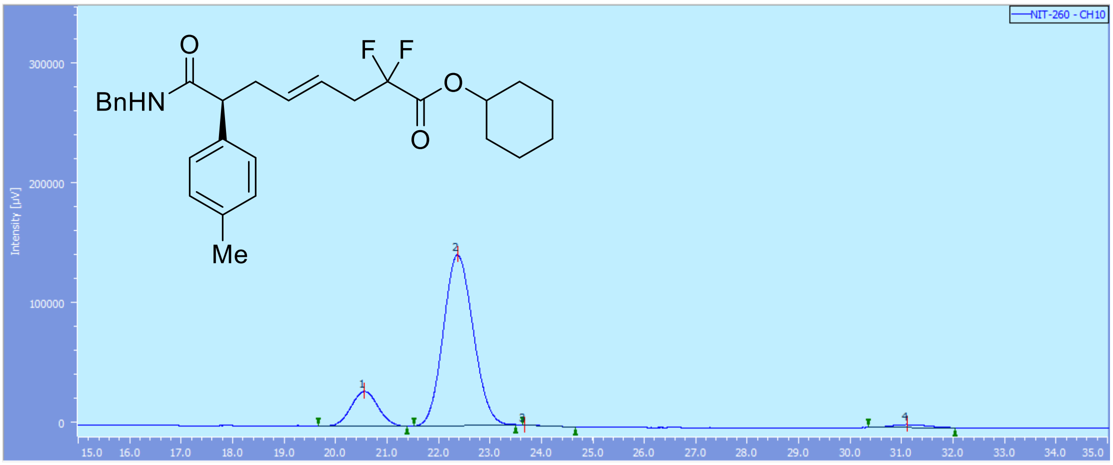

#	Peak Name	CH	tR	Area	Height	Area%	Height%	Quantity	NTP	Resolution	Symmetry Factor	Warning
1	Unknown	10	20.557	1064743	28919	15.380	16.634	N/A	7031	1.763	1.046	
2	Unknown	10	22.363	5733632	142322	82.822	81.864	N/A	6931	1.967	1.096	
3	Unknown	10	23.660	343	72	0.005	0.042	N/A	146094	9.145	14.595	
4	Unknown	10	31.083	124095	2539	1.793	1.461	N/A	8116	N/A	1.172	

Benzyl (*S*,*E*)-8-(benzylamino)-2,2-difluoro-8-oxo-7-(*p*-tolyl)oct-4-enoate (2.27)

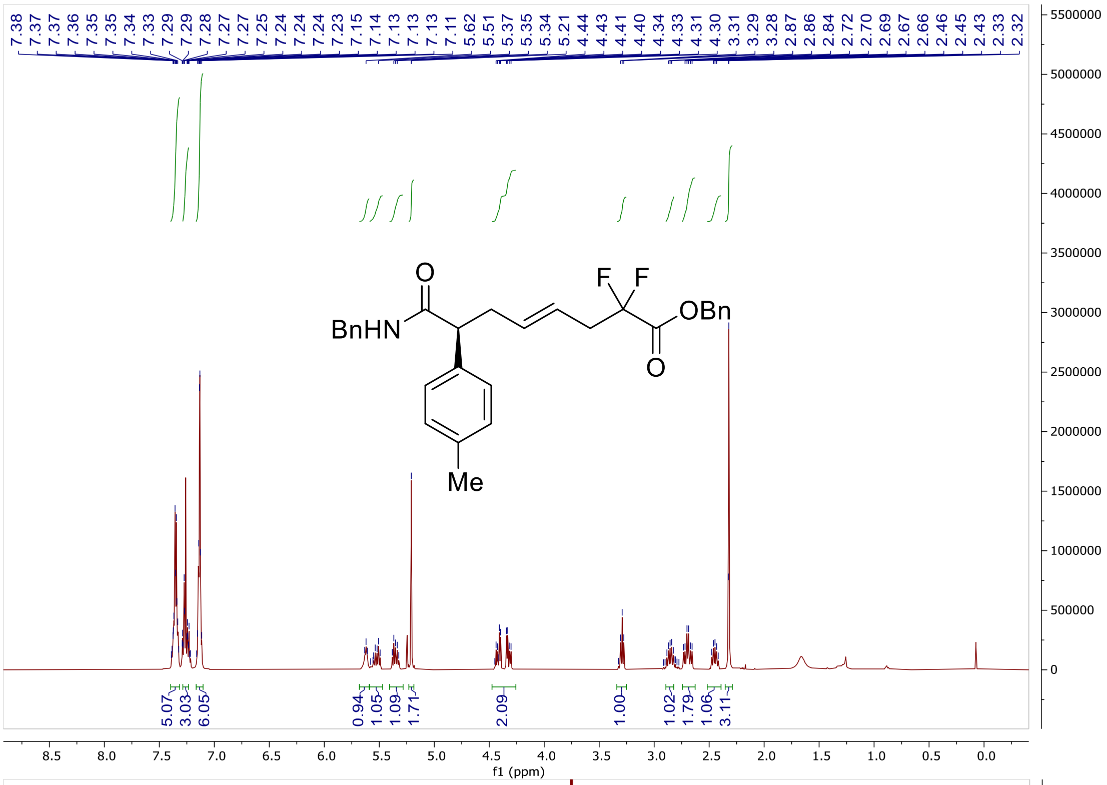

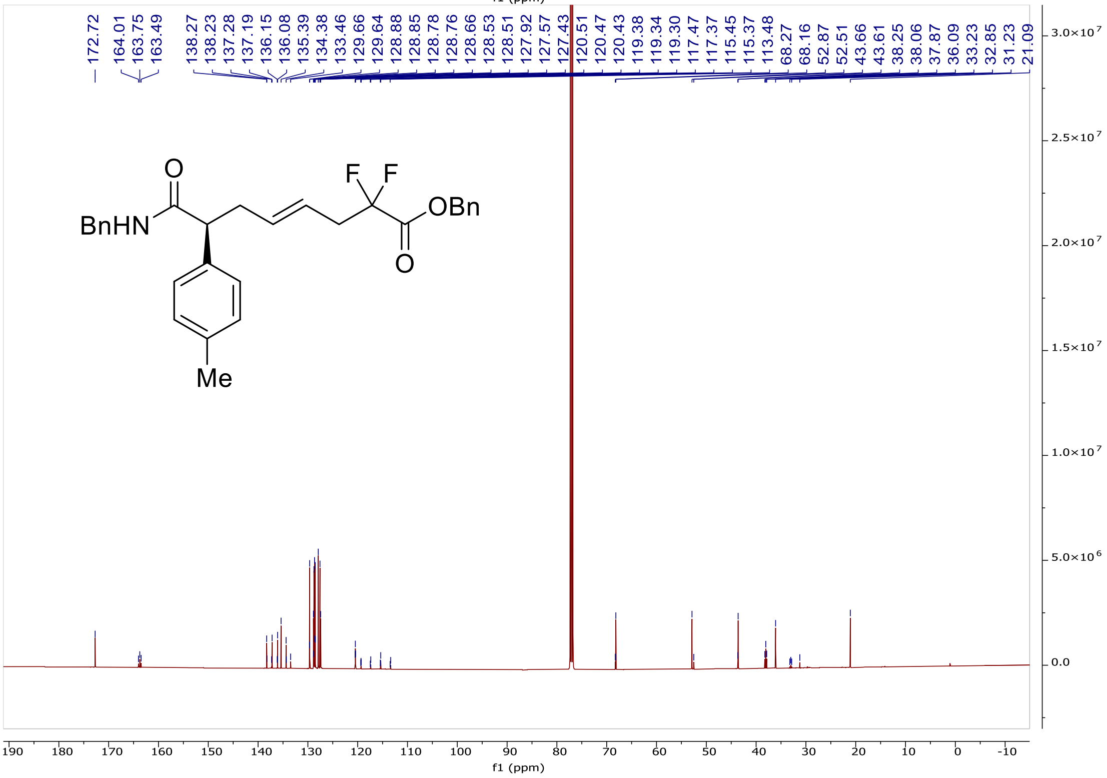

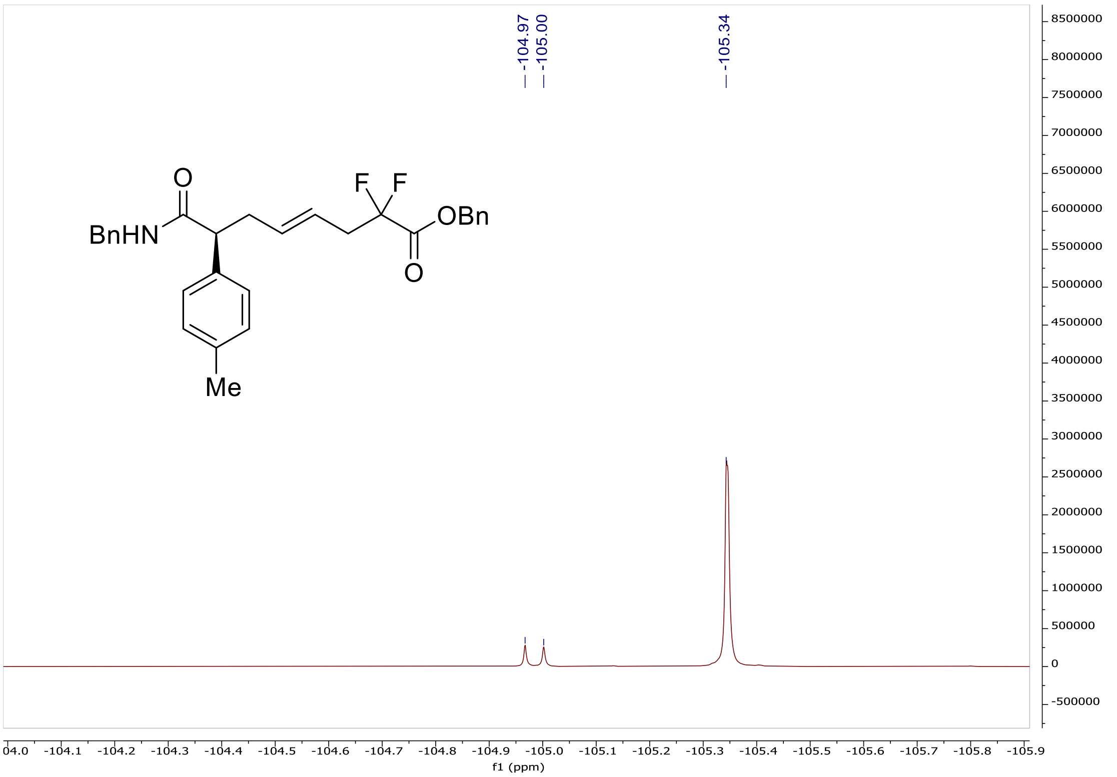
-104.97
-105.00
-105.34
O
F F
OBn
BnHN
O
Me
8500000
8000000
7500000
7000000
6500000
6000000
5500000
5000000
4500000
4000000
3500000
3000000
2500000
2000000
1500000
1000000
500000
0
-500000
04.0 -104.1 -104.2 -104.3 -104.4 -104.5 -104.6 -104.7 -104.8 -104.9 -105.0 -105.1 -105.2 -105.3 -105.4 -105.5 -105.6 -105.7 -105.8 -105.9
f1 (ppm)

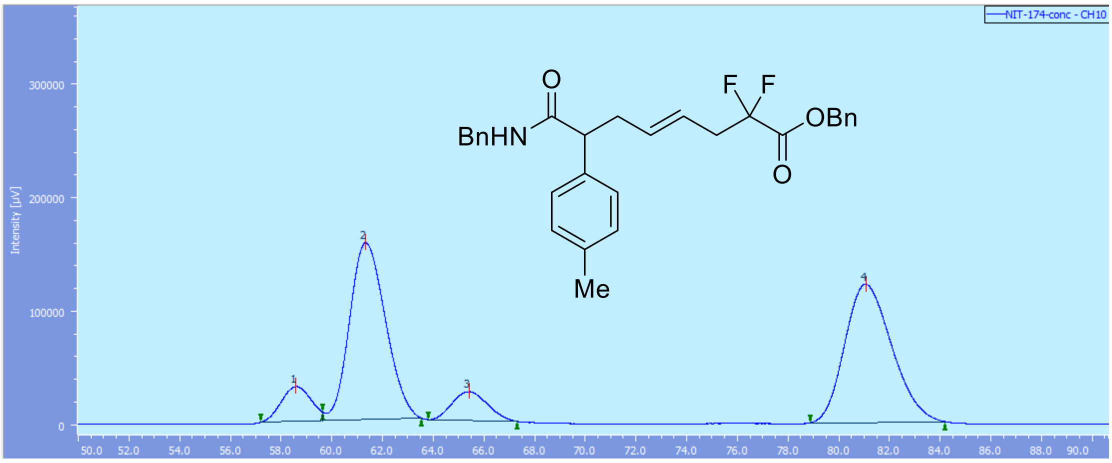

#	Peak Name	CH	tR	Area	Height	Area%	Height%	Quantity	NTP	Resolution	Symmetry Factor	Warning
1	Unknown	10	58.563	2505170	30706	6.959	9.213	N/A	10447	1.124	N/A	
2	Unknown	10	61.327	15392835	155677	42.762	46.708	N/A	8658	1.549	1.142	
3	Unknown	10	65.410	2452771	25437	6.814	7.632	N/A	9750	5.143	1.107	
4	Unknown	10	81.033	15645771	121477	43.465	36.447	N/A	8868	N/A	1.207	

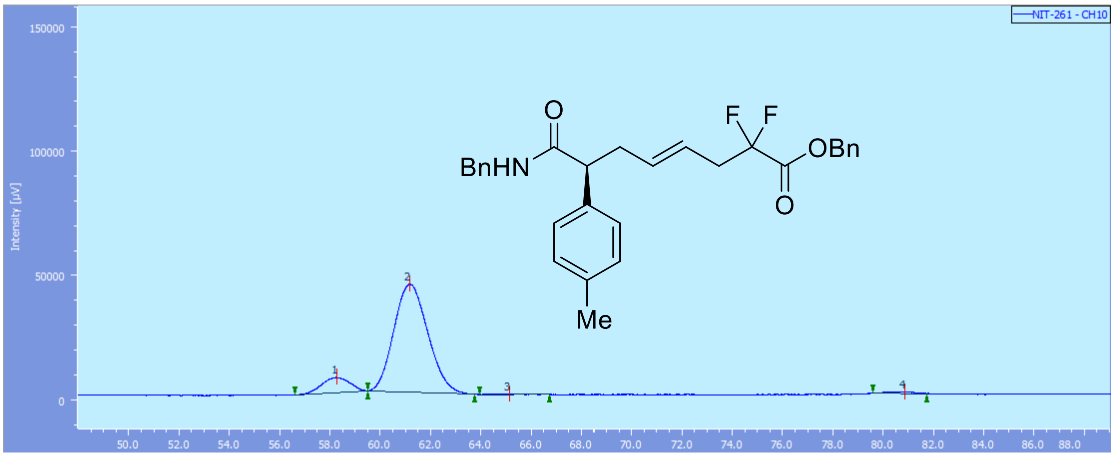

#	Peak Name	CH	tR	Area	Height	Area%	Height%	Quantity	NTP	Resolution	Symmetry Factor	Warning
1	Unknown	10	58.250	458212	6010	10.021	11.967	N/A	11910	1.260	0.948	
2	Unknown	10	61.173	4060389	43331	88.796	86.278	N/A	9445	1.591	1.137	
3	Unknown	10	65.140	8140	167	0.178	0.333	N/A	11010	6.591	1.148	
4	Unknown	10	80.823	45975	714	1.005	1.421	N/A	19952	N/A	0.839	

(*S*,*E*)-*N*[8]-Benzyl-2,2-difluoro-*N*[1]-phenyl-7-(*p*-tolyl)oct-4-enediamide (2.28)

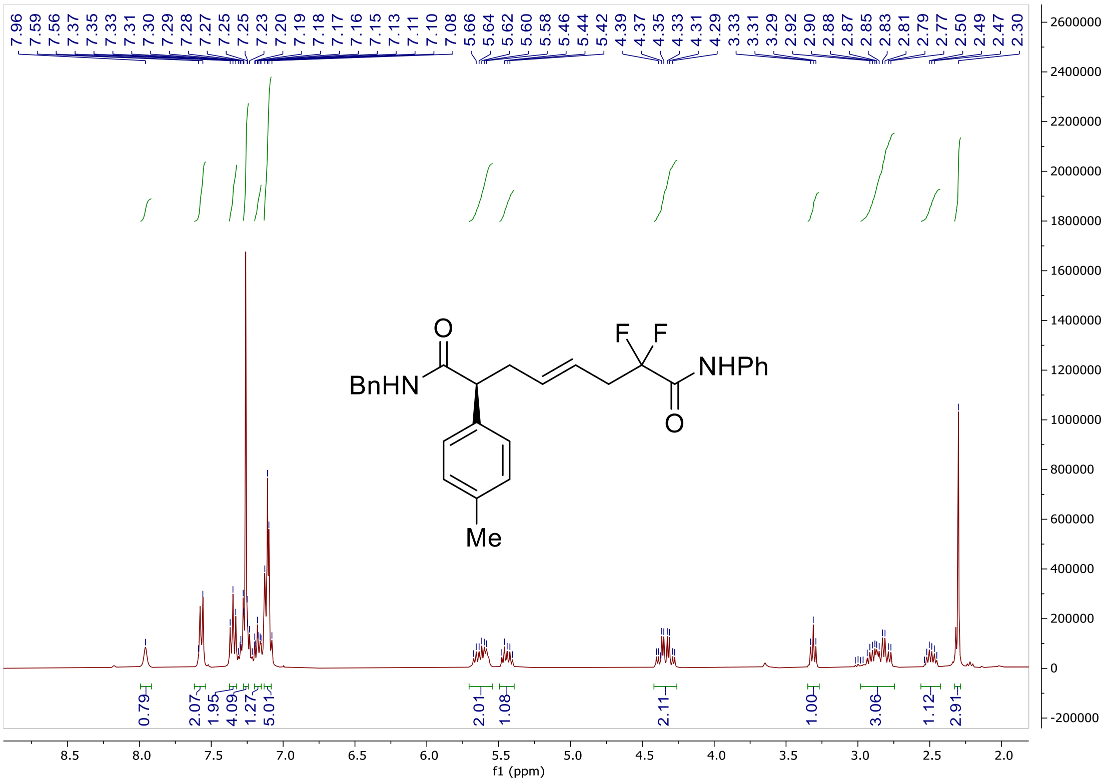

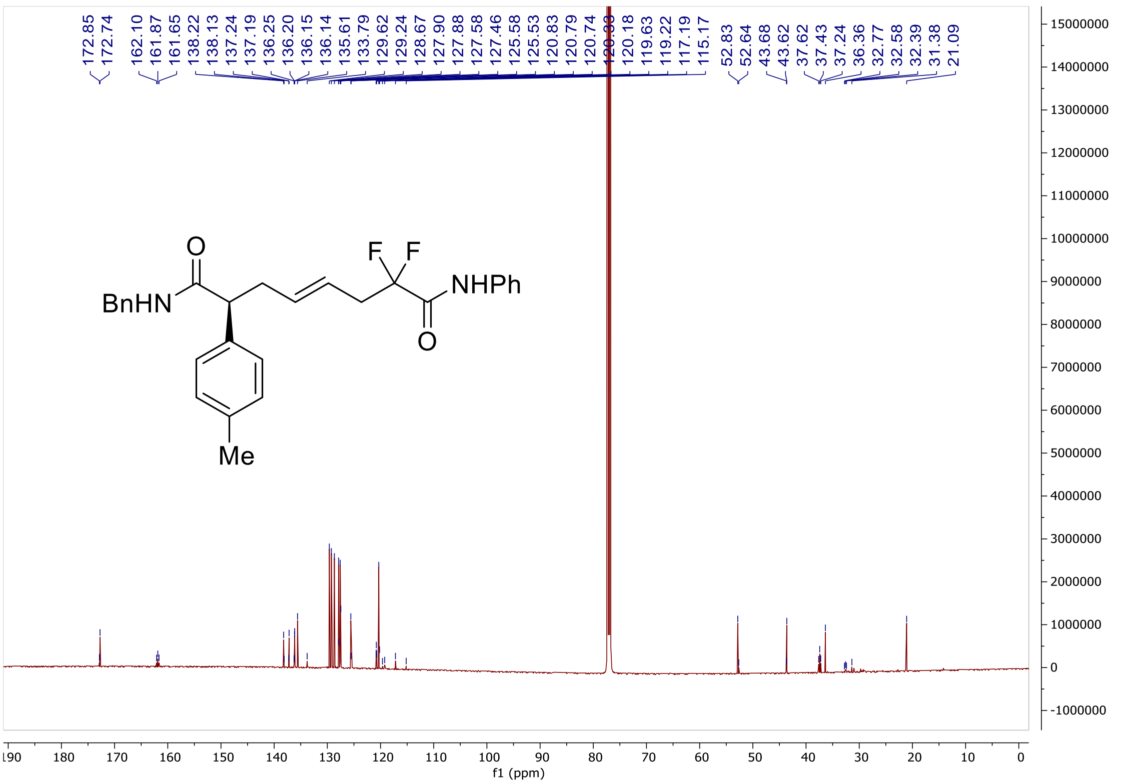

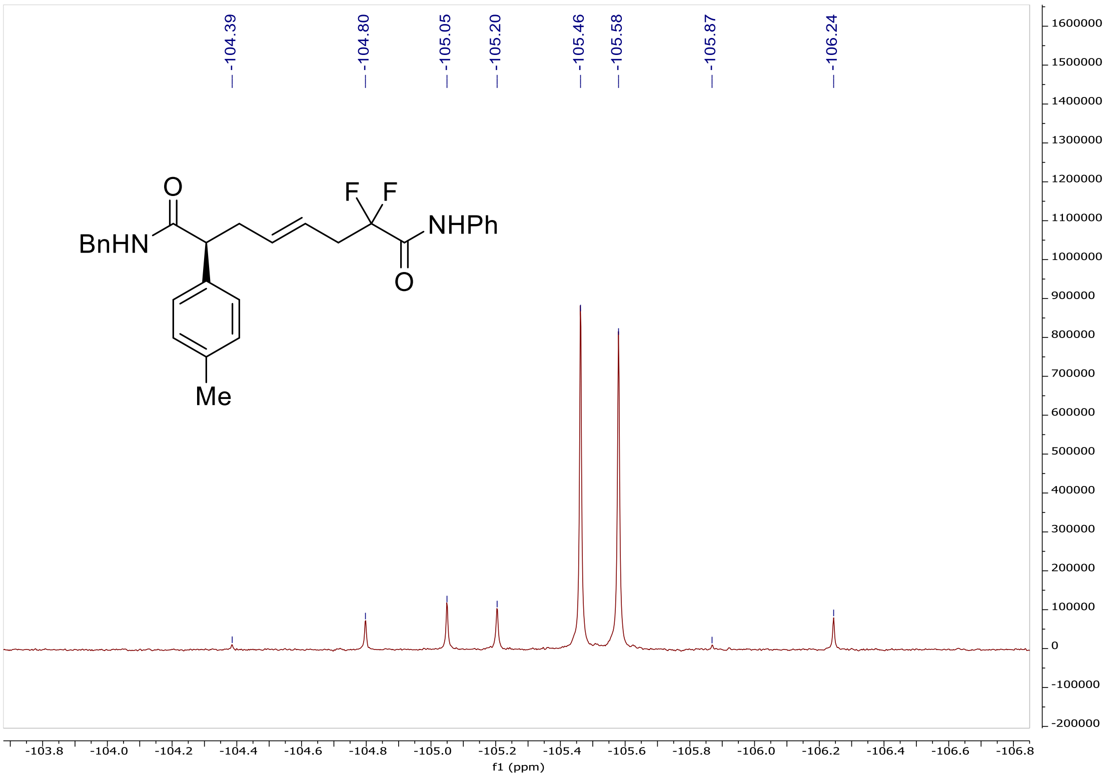

-104.39
-104.80
-105.05
-105.20
-105.46
-105.58
-105.87
-106.24
BnHN
NHPh
F
F
O
O
Me
1600000
1500000
1400000
1300000
1200000
1100000
1000000
900000
800000
700000
600000
500000
400000
300000
200000
100000
0
-100000
-200000
-103.8
-104.0
-104.2
-104.4
-104.6
-104.8
-105.0
-105.2
-105.4
-105.6
-105.8
-106.0
-106.2
-106.4
-106.6
-106.8
f1 (ppm)

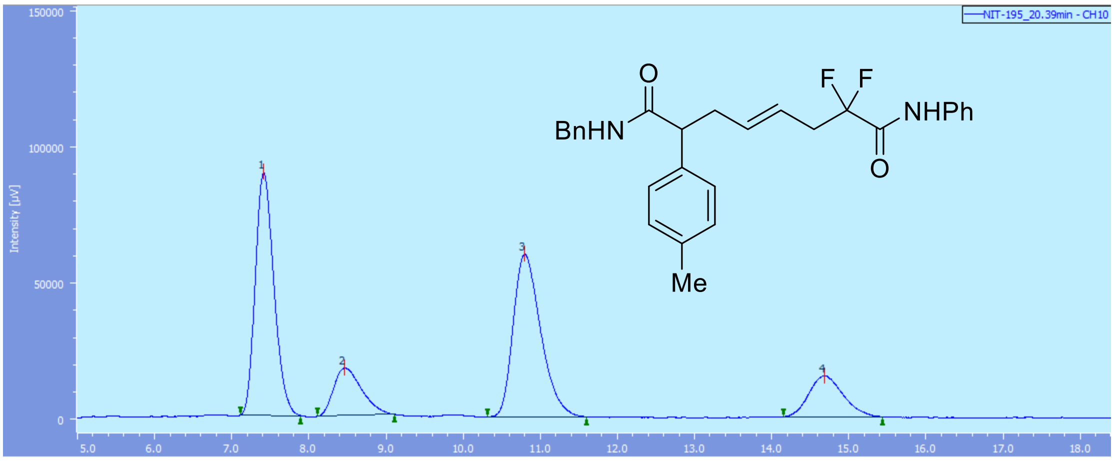

#	Peak Name	CH	tR	Area	Height	Area%	Height%	Quantity	NTP	Resolution	Symmetry Factor	Warning
1	Unknown	10	7.413	1487855	88833	38.462	49.137	N/A	4498	1.886	1.210	
2	Unknown	10	8.463	442090	17427	11.428	9.640	N/A	2523	3.531	1.409	
3	Unknown	10	10.793	1477202	59493	38.187	32.908	N/A	4423	5.324	1.342	
4	Unknown	10	14.670	461200	15033	11.922	8.316	N/A	5236	N/A	1.207	

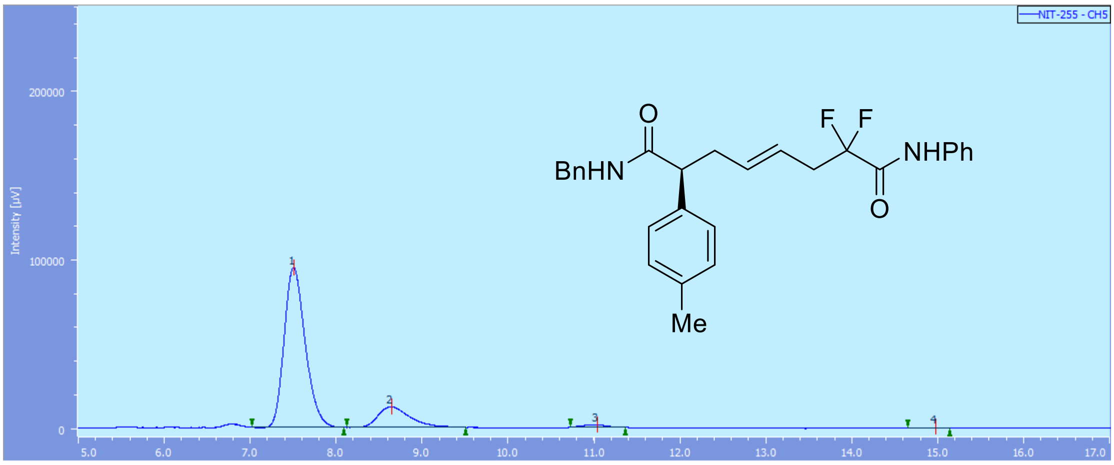

#	Peak Name	CH	tR	Area	Height	Area%	Height%	Quantity	NTP	Resolution	Symmetry Factor	Warning
1	Unknown	5	7.503	1605276	94522	82.026	87.031	N/A	4538	2.063	1.234	
2	Unknown	5	8.638	317786	12244	16.238	11.273	N/A	2760	3.964	1.481	
3	Unknown	5	11.040	31225	1570	1.596	1.446	N/A	6282	6.691	1.001	
4	Unknown	5	14.968	2736	272	0.140	0.250	N/A	9319	N/A	0.783	

(*S,E*)-*N*[1],*N*[8]-Dibenzyl-2,2-difluoro-7-(*p*-tolyl)oct-4-enediamide (2.29)

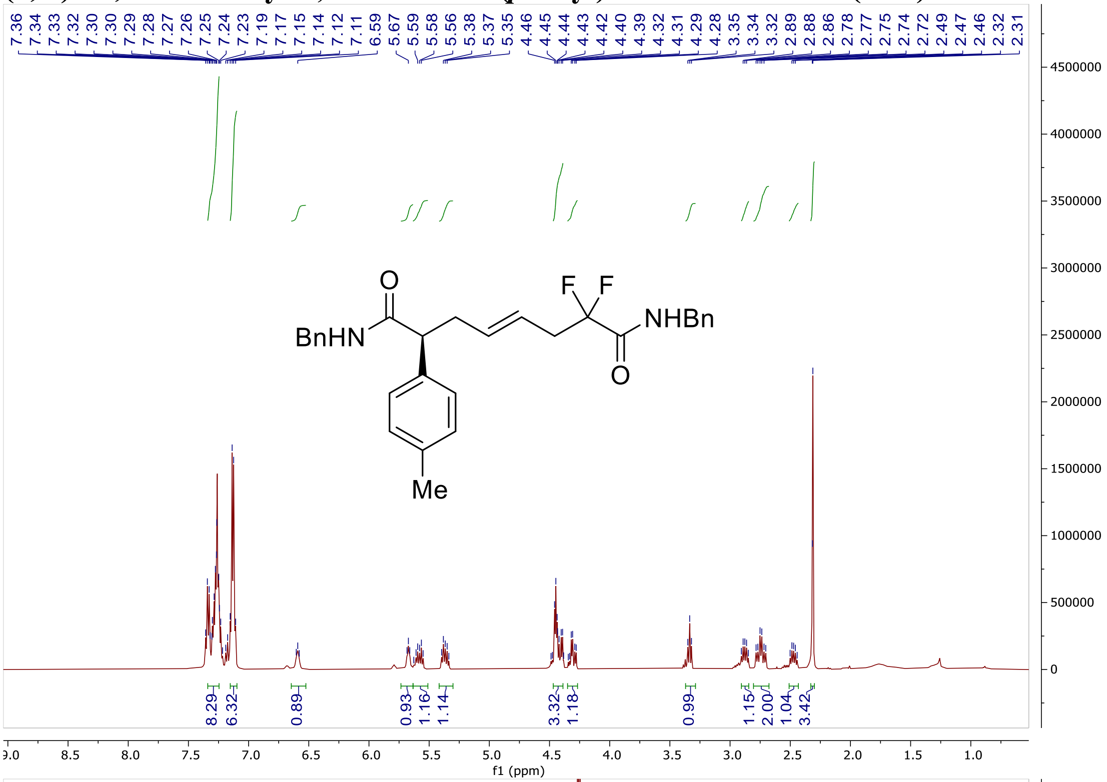

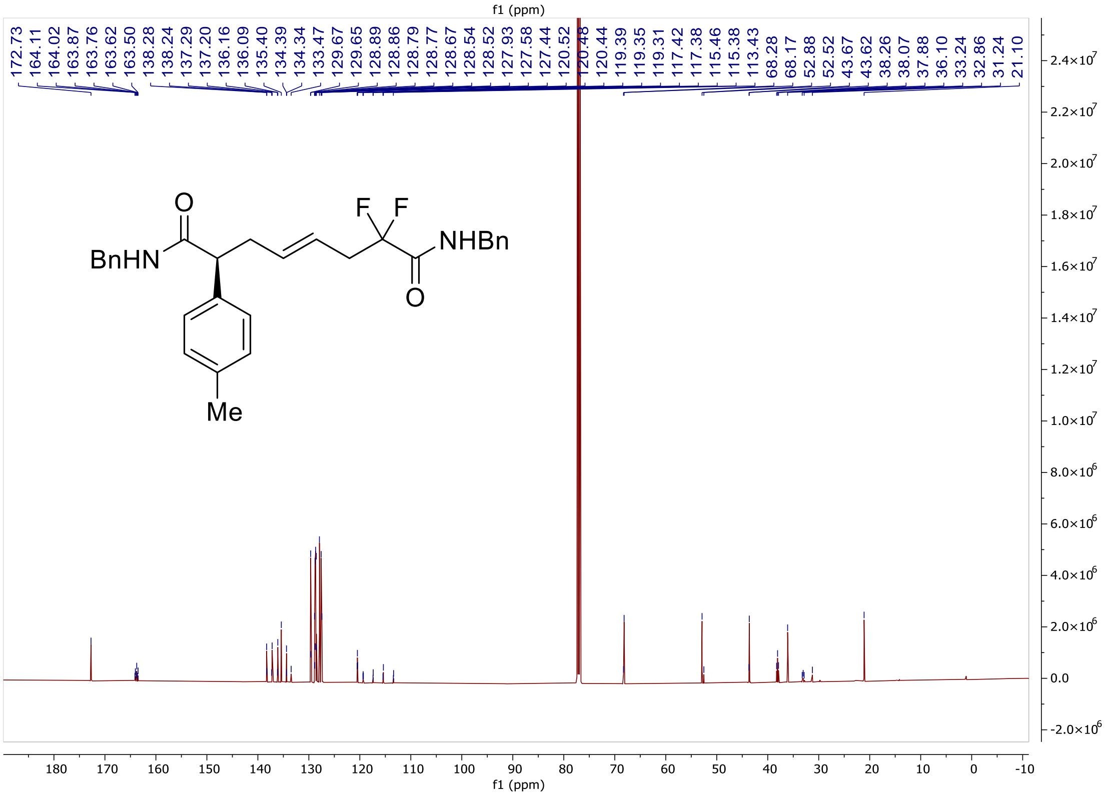

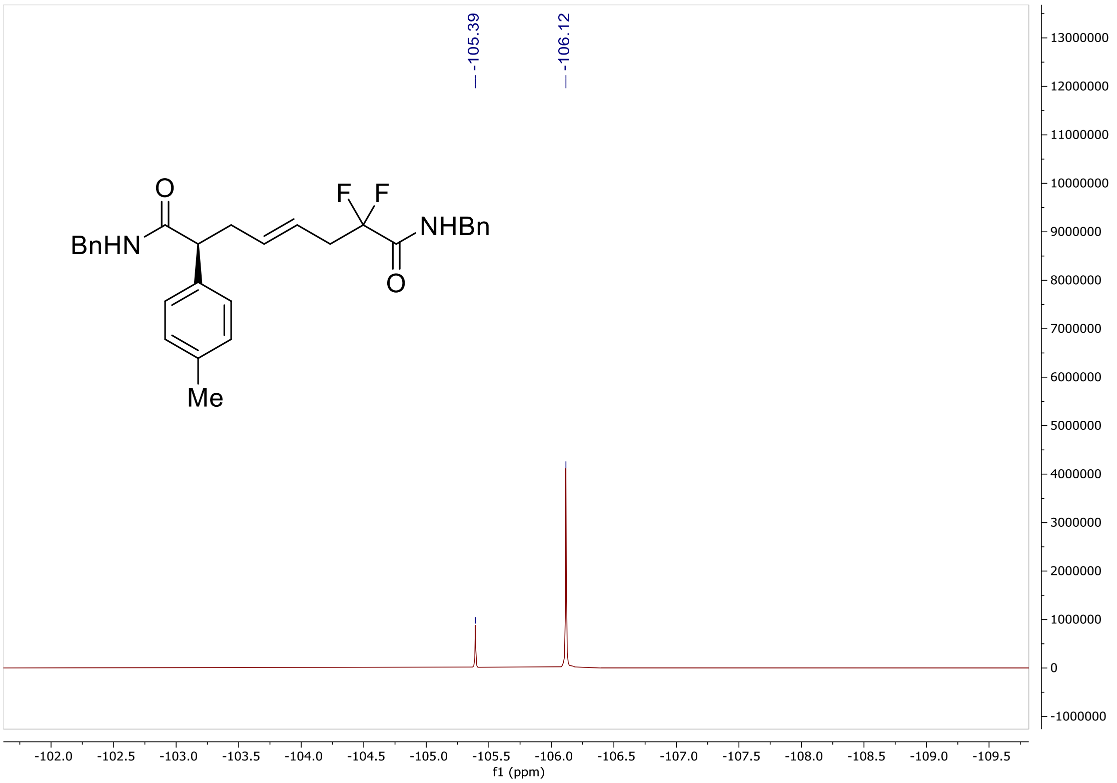

-105.39
-106.12
BnHN
O
F
F
NHBn
O
Me
13000000
12000000
11000000
10000000
9000000
8000000
7000000
6000000
5000000
4000000
3000000
2000000
1000000
0
-1000000
-102.0
-102.5
-103.0
-103.5
-104.0
-104.5
-105.0
-105.5
-106.0
-106.5
-107.0
-107.5
-108.0
-108.5
-109.0
-109.5
f1 (ppm)

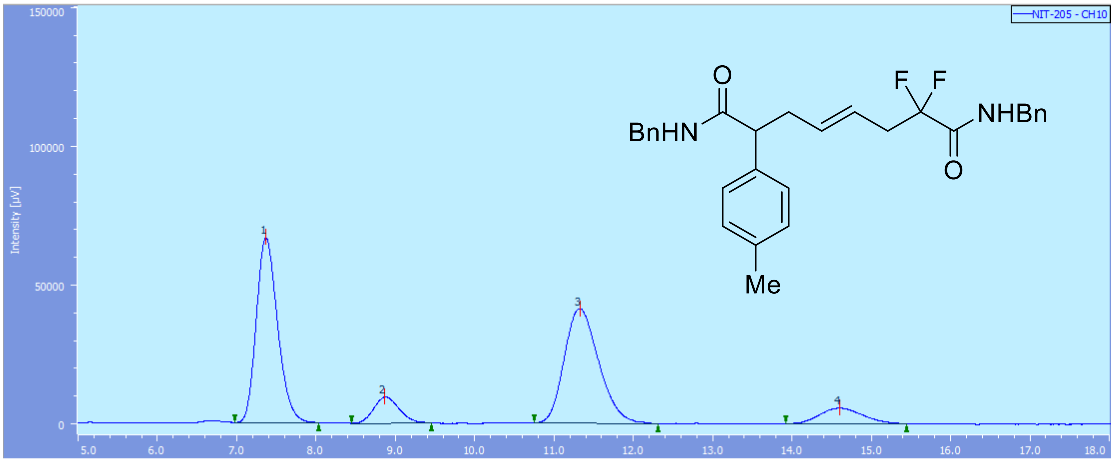

#	Peak Name	CH	tR	Area	Height	Area%	Height%	Quantity	NTP	Resolution	Symmetry Factor	Warning
1	Unknown	10	7.363	1238704	66451	42.635	54.081	N/A	3630	2.798	1.233	
2	Unknown	10	8.863	210248	9460	7.237	7.699	N/A	3658	3.565	1.198	
3	Unknown	10	11.320	1245255	41248	42.860	33.570	N/A	3238	3.675	1.221	
4	Unknown	10	14.597	211171	5714	7.268	4.650	N/A	3462	N/A	1.154	

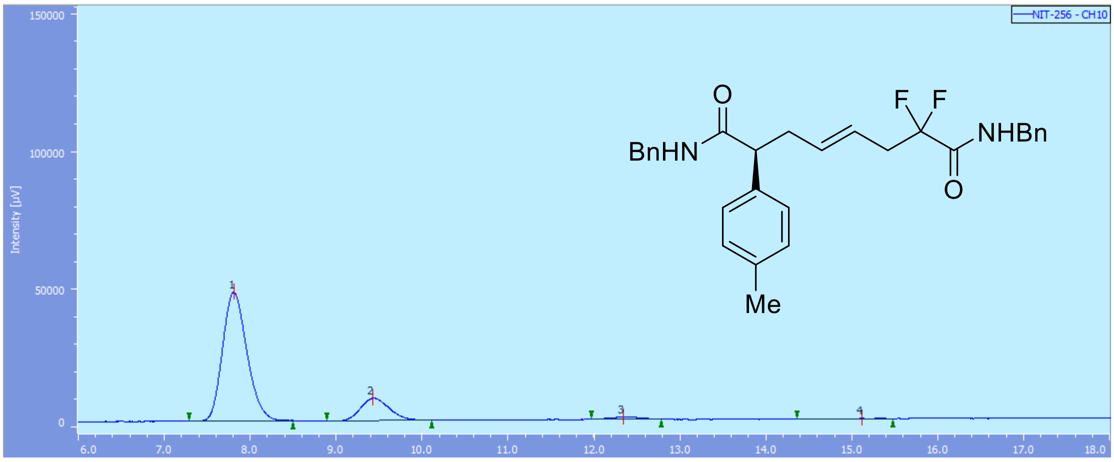

#	Peak Name	CH	tR	Area	Height	Area%	Height%	Quantity	NTP	Resolution	Symmetry Factor	Warning
1	Unknown	10	7.807	911208	46513	81.526	83.844	N/A	3641	2.882	1.201	
2	Unknown	10	9.427	185681	8074	16.613	14.553	N/A	3823	4.397	1.233	
3	Unknown	10	12.333	18586	760	1.663	1.369	N/A	4750	2.623	1.189	
4	Unknown	10	15.113	2210	129	0.198	0.233	N/A	1840	N/A	0.766	

(*S,E*)-*N*[8]-Benzyl-*N*[1]-cyclopentyl-2,2-difluoro-7-(*p*-tolyl)oct-4-enediamide (2.30)

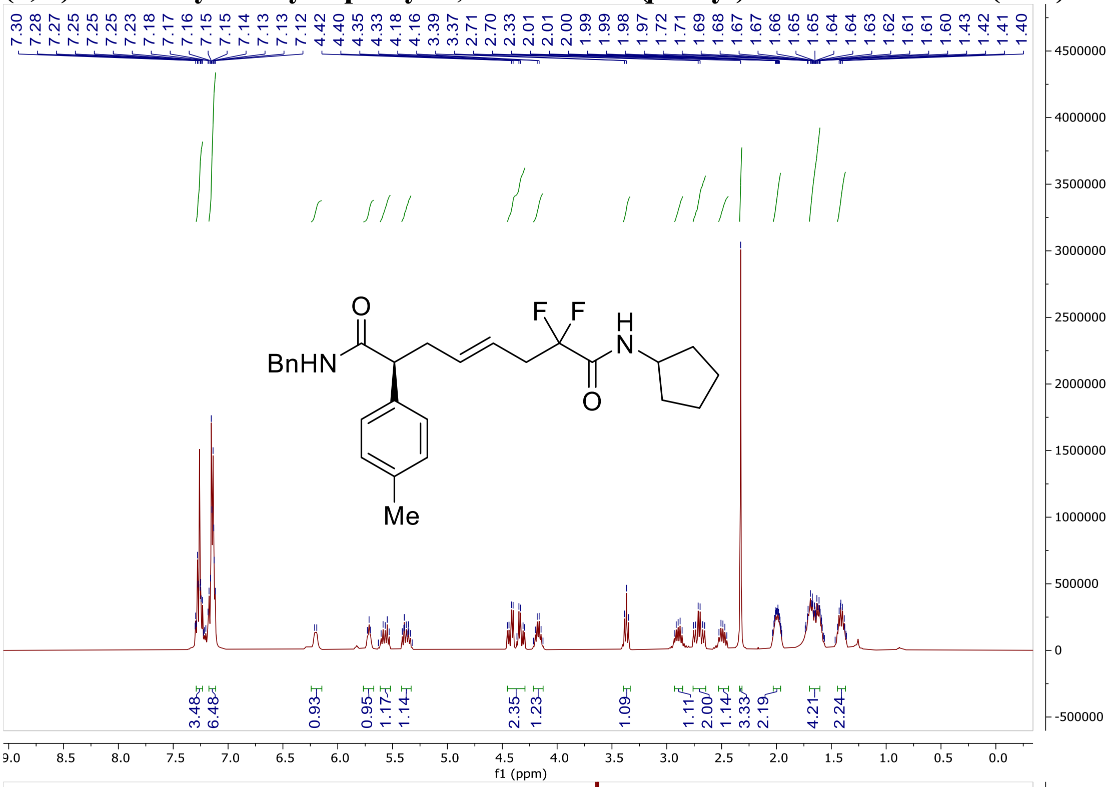

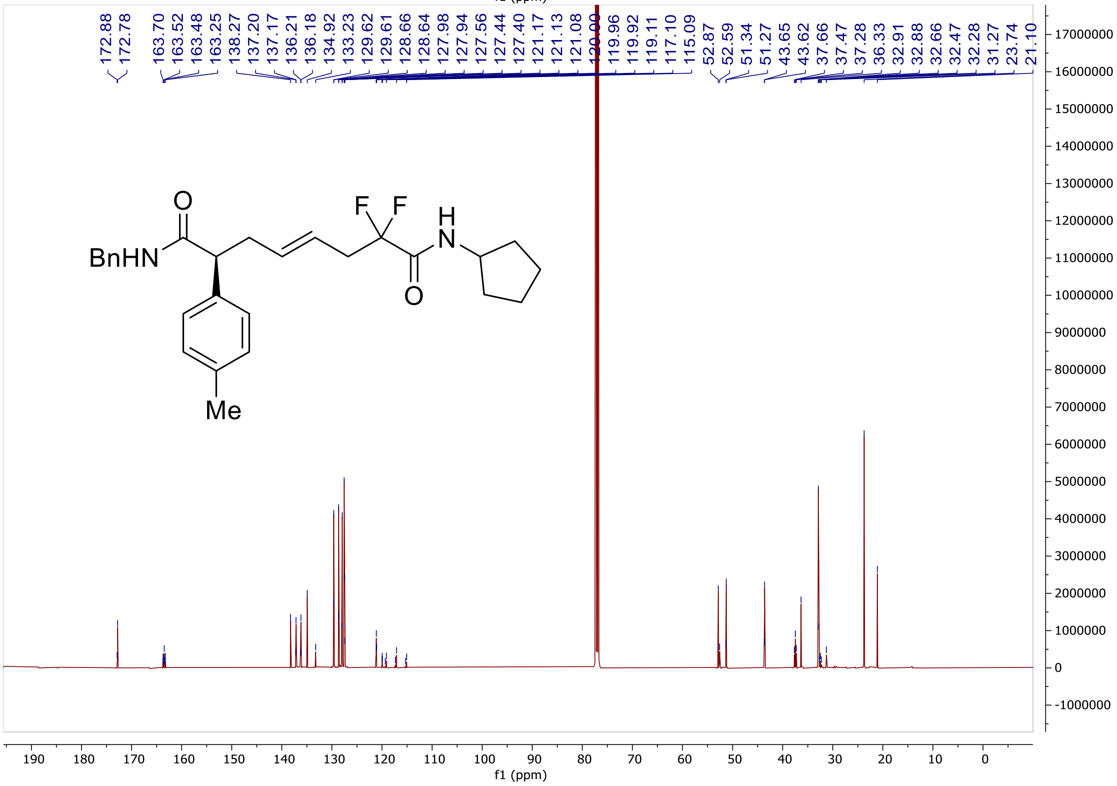

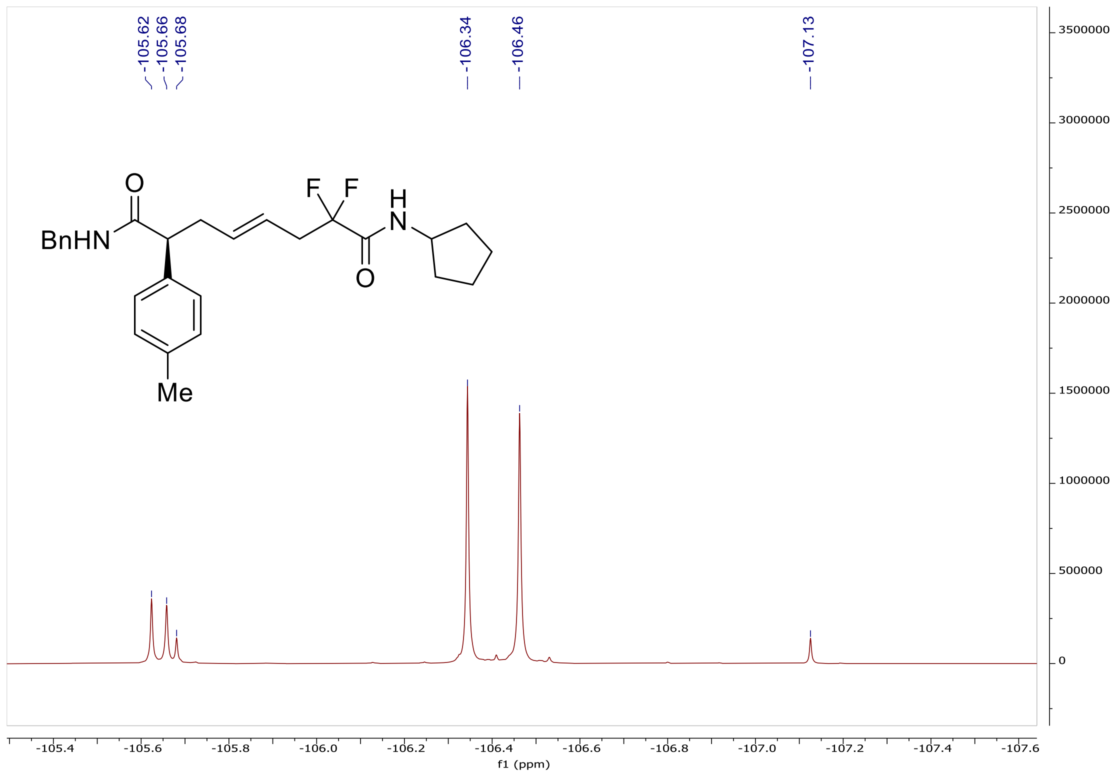
-105.62
-105.66
-105.68
-106.34
-106.46
-107.13
BnHN
O
F F
H
N
O
Me
3500000
3000000
2500000
2000000
1500000
1000000
500000
0
-105.4
-105.6
-105.8
-106.0
-106.2
-106.4
-106.6
-106.8
-107.0
-107.2
-107.4
-107.6
f1 (ppm)

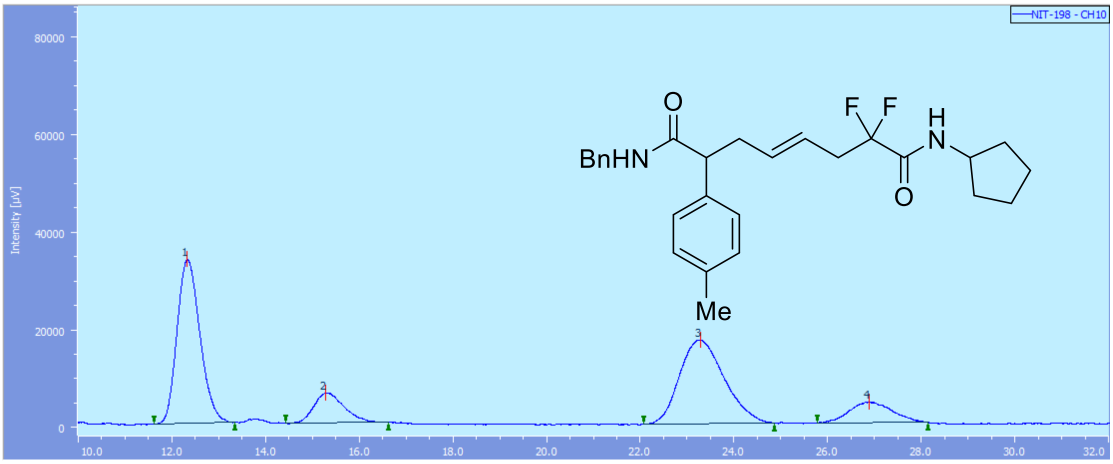

#	Peak Name	CH	tR	Area	Height	Area%	Height%	Quantity	NTP	Resolution	Symmetry Factor	Warning
1	Unknown	10	12.327	1139080	33510	40.468	55.013	N/A	3021	2.854	1.218	
2	Unknown	10	15.277	274828	6152	9.764	10.100	N/A	2701	5.447	1.376	
3	Unknown	10	23.287	1130065	17024	40.148	27.948	N/A	2759	2.078	1.171	
4	Unknown	10	26.880	270770	4227	9.620	6.939	N/A	4025	N/A	1.107	

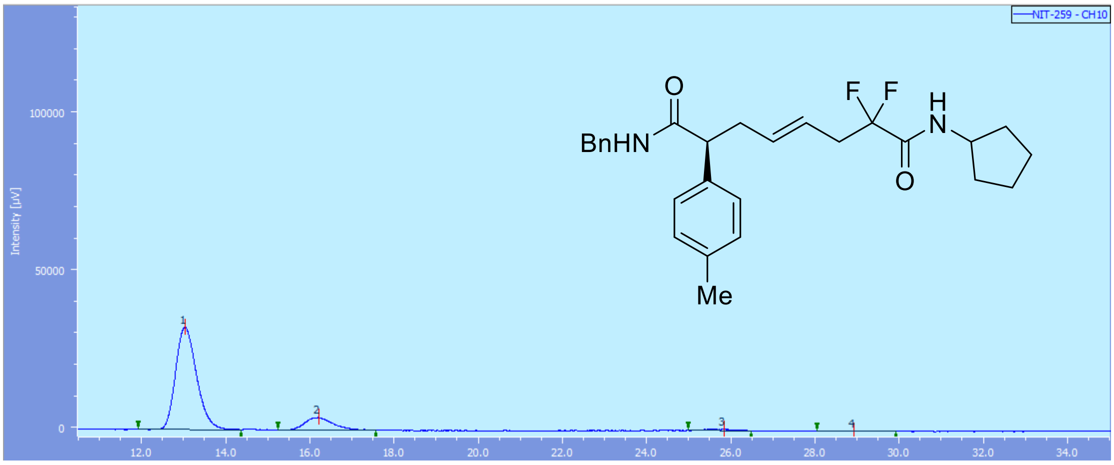

#	Peak Name	CH	tR	Area	Height	Area%	Height%	Quantity	NTP	Resolution	Symmetry Factor	Warning
1	Unknown	10	13.037	1128394	32378	84.357	87.713	N/A	3306	3.000	1.233	
2	Unknown	10	16.217	183085	3956	13.687	10.717	N/A	2832	7.049	1.199	
3	Unknown	10	25.820	19420	417	1.452	1.130	N/A	4659	1.770	0.920	
4	Unknown	10	28.913	6743	162	0.504	0.440	N/A	3373	N/A	1.068	

(*S*,*E*)-*N*-Benzyl-7,7-difluoro-8-oxo-8-(piperidin-1-yl)-2-(*p*-tolyl)oct-4-enamide (2.31)

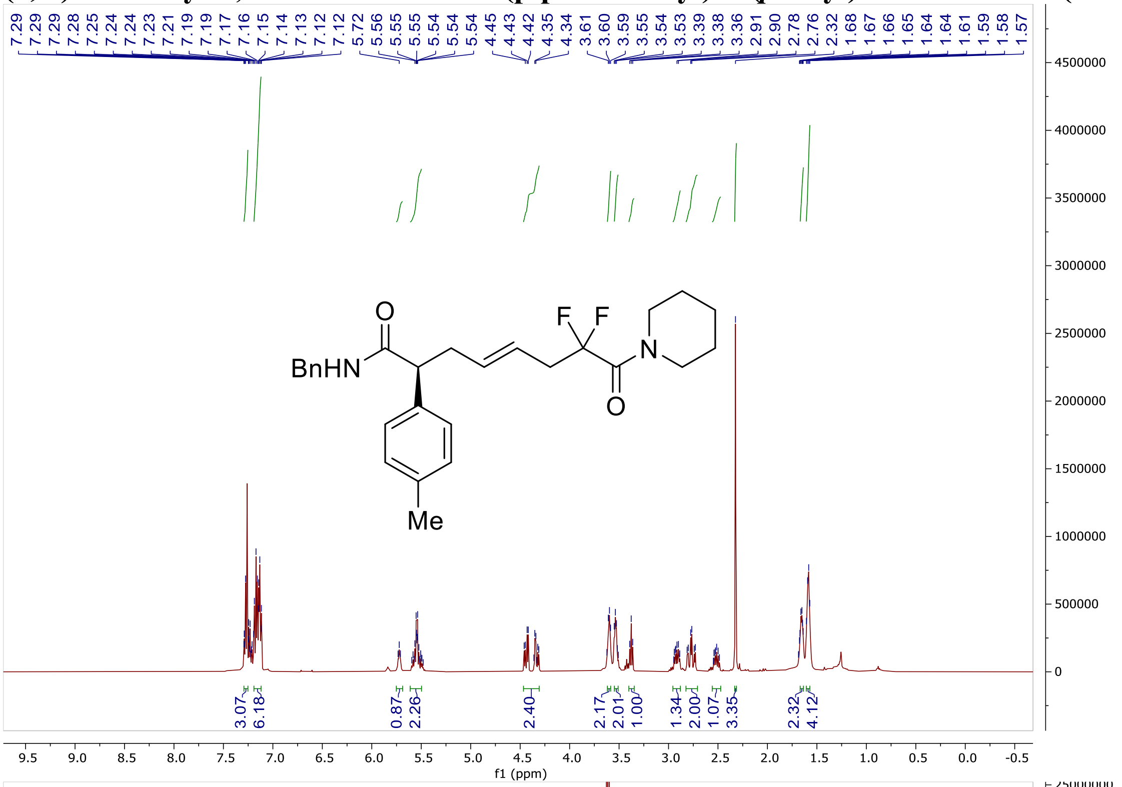

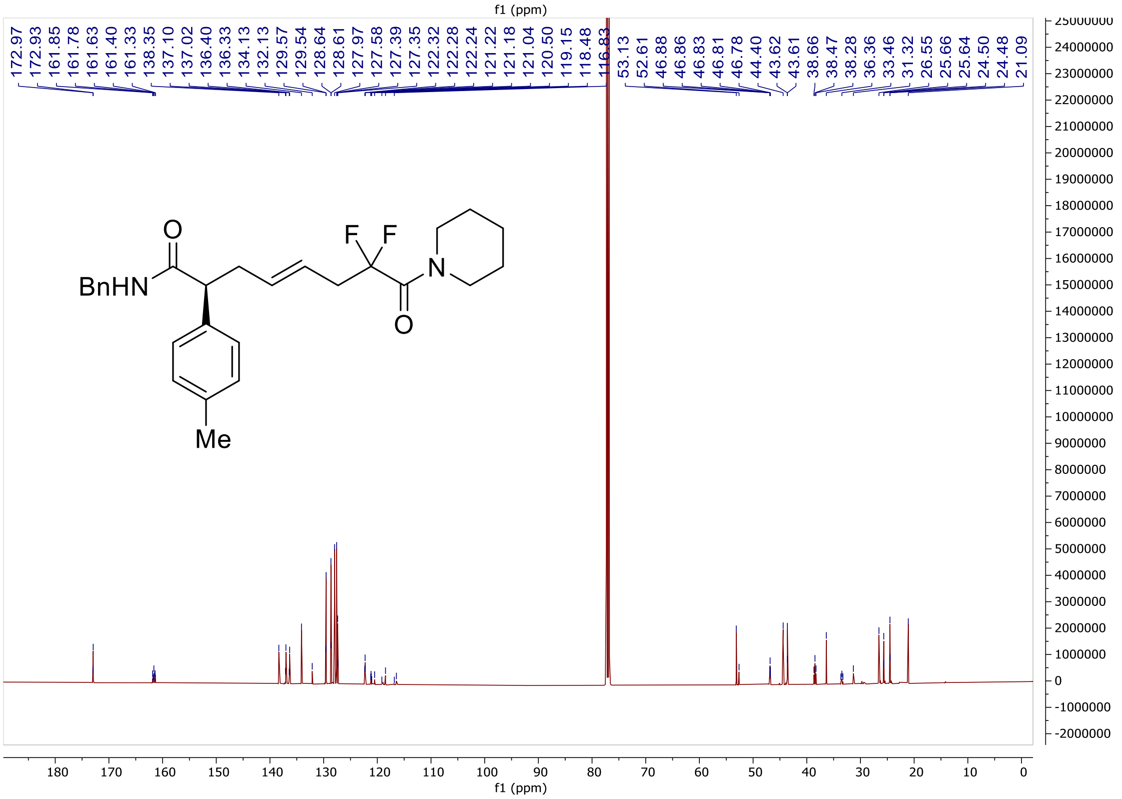

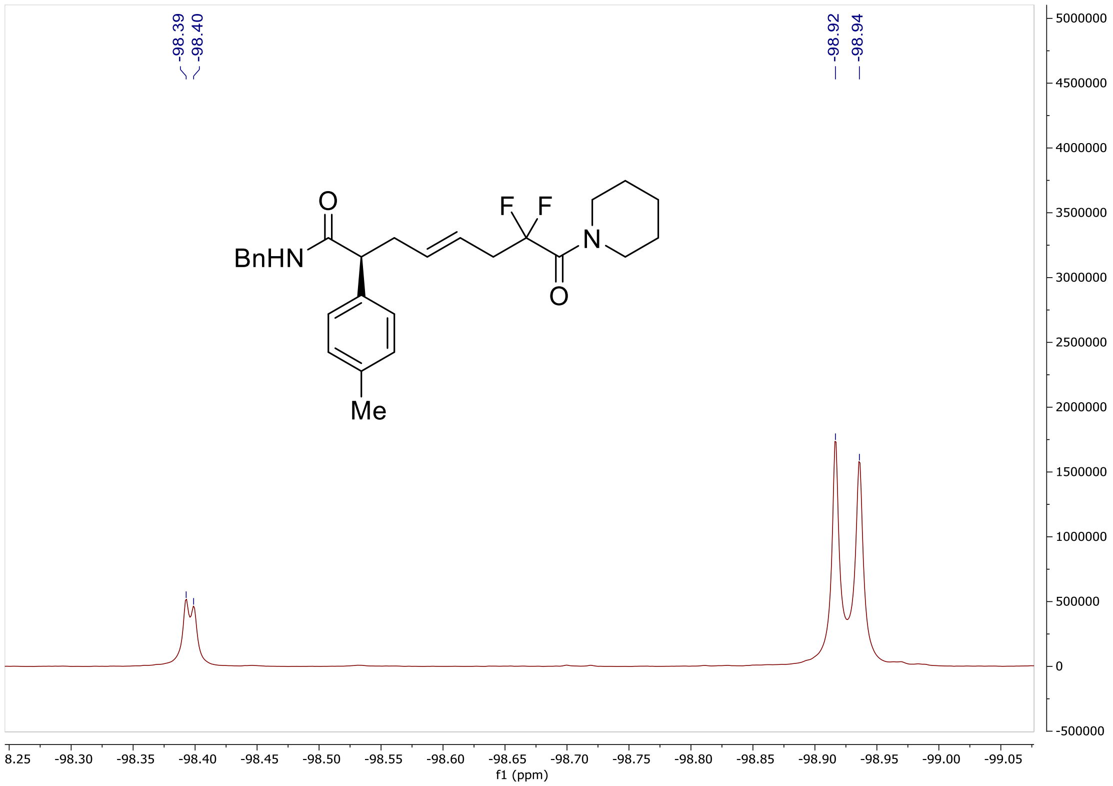

-98.39
-98.40
-98.92
-98.94
BnHN
O
F
F
N
O
Me
5000000
4500000
4000000
3500000
3000000
2500000
2000000
1500000
1000000
500000
0
-500000
8.25
-98.30
-98.35
-98.40
-98.45
-98.50
-98.55
-98.60
-98.65
-98.70
-98.75
-98.80
-98.85
-98.90
-98.95
-99.00
-99.05
f1 (ppm)

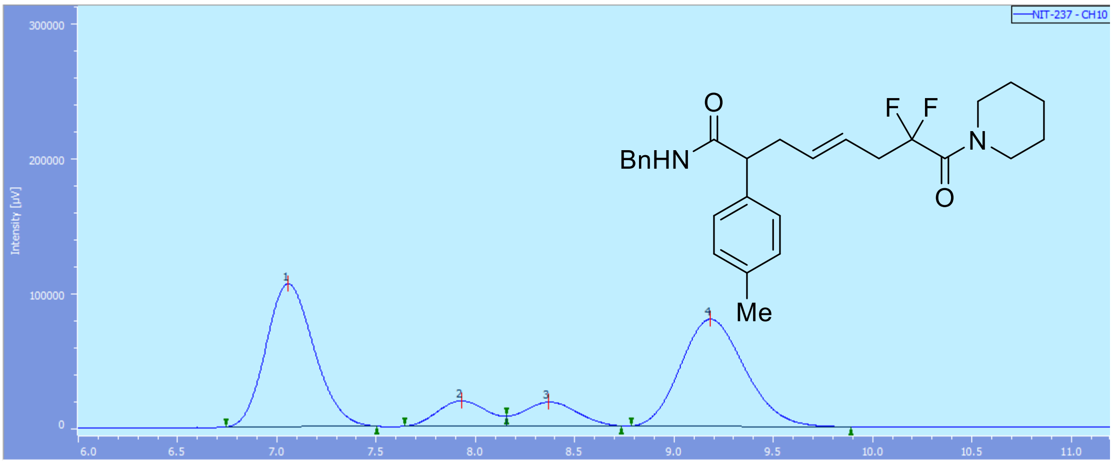

#	Peak Name	CH	tR	Area	Height	Area%	Height%	Quantity	NTP	Resolution	Symmetry Factor	Warning
1	Unknown	10	7.057	1774753	105696	42.160	47.793	N/A	4014	1.858	1.155	
2	Unknown	10	7.927	324486	18626	7.708	8.422	N/A	4129	0.850	N/A	
3	Unknown	10	8.367	335935	17616	7.980	7.965	N/A	3783	1.433	N/A	
4	Unknown	10	9.180	1774386	79217	42.151	35.820	N/A	3821	N/A	1.161	

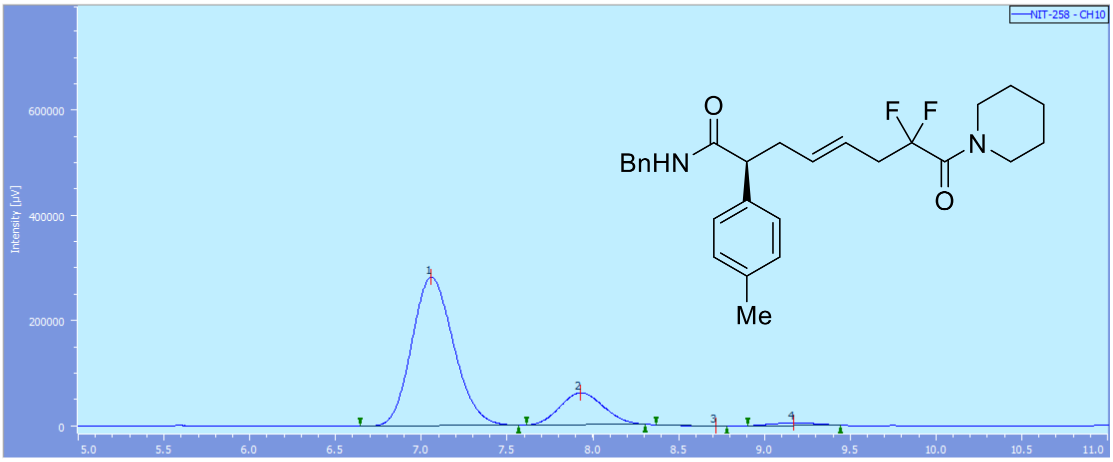

#	Peak Name	CH	tR	Area	Height	Area%	Height%	Quantity	NTP	Resolution	Symmetry Factor	Warning
1	Unknown	10	7.057	4780964	282036	80.298	80.978	N/A	3975	1.890	1.150	
2	Unknown	10	7.927	1064975	60354	17.887	17.329	N/A	4446	1.505	1.093	
3	Unknown	10	8.713	12239	541	0.206	0.155	N/A	3704	0.841	0.580	
4	Unknown	10	9.167	95850	5356	1.610	1.538	N/A	5215	N/A	1.034	

(*S*,*E*)-*N*-benzyl-2-(*p*-tolyl)-6-(*p*-tolylthio)hex-4-enamide (2.32)

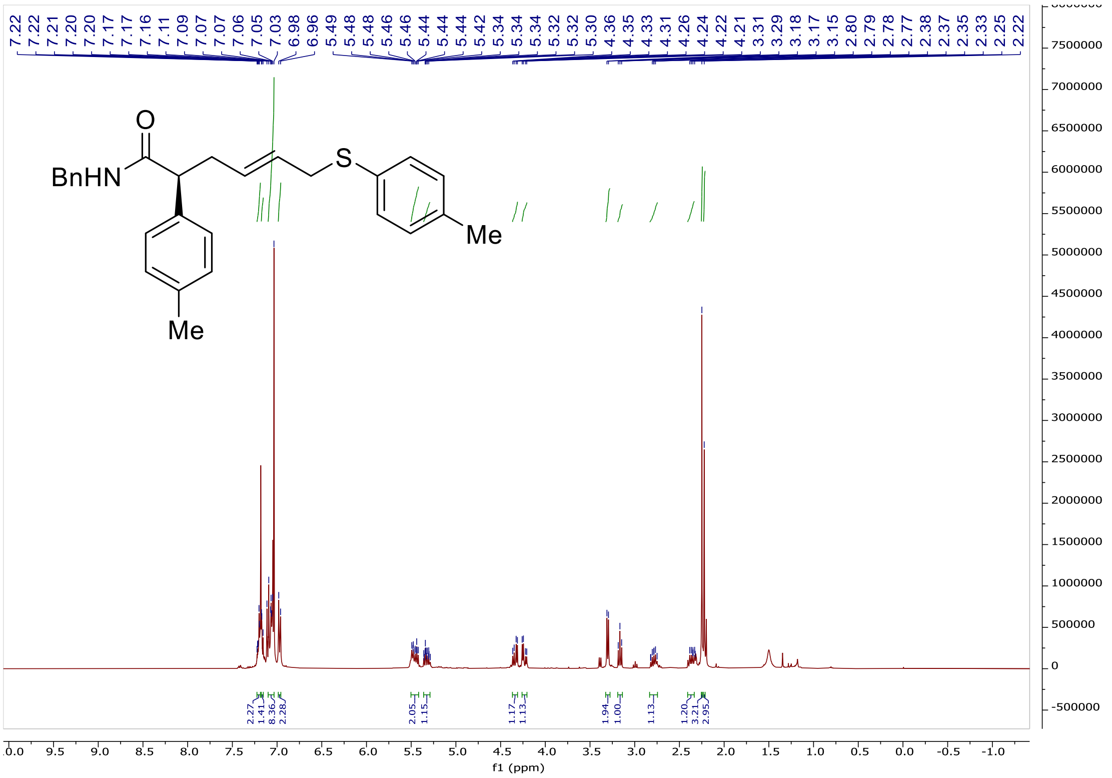

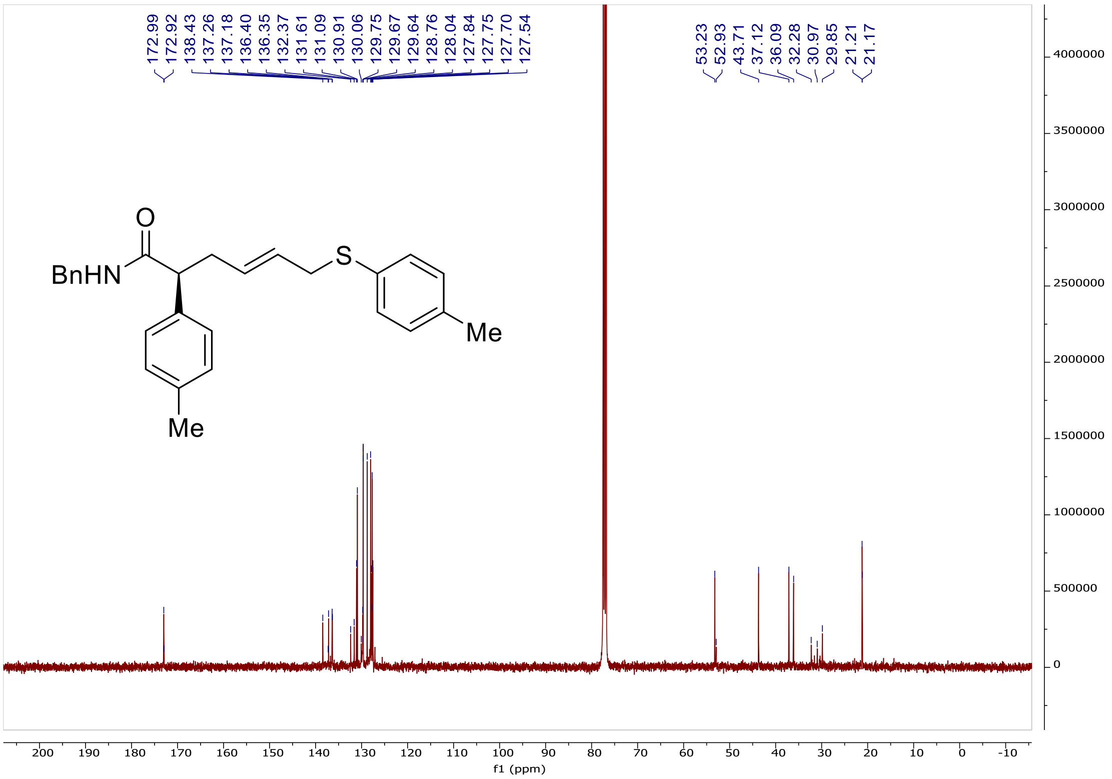

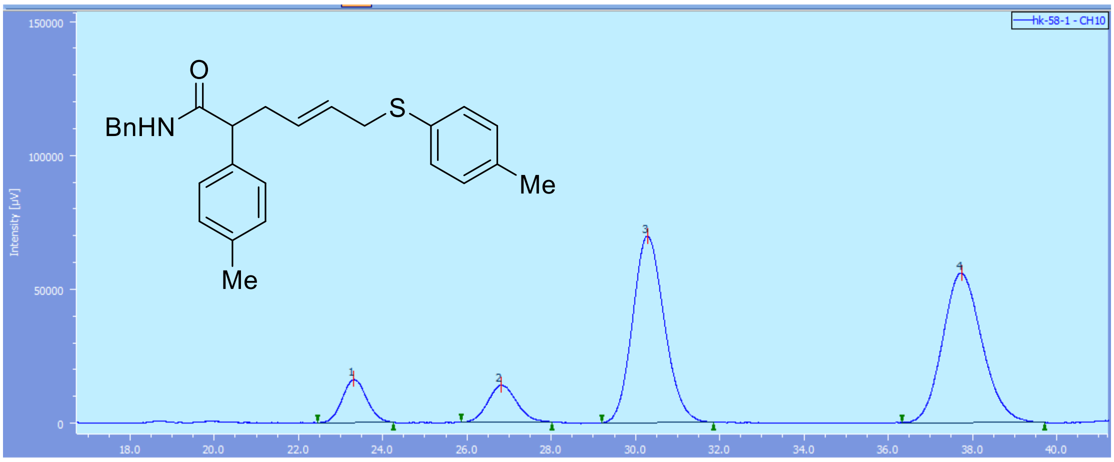

#	Peak Name	CH	tR	Area	Height	Area%	Height%	Quantity	NTP	Resolution	Symmetry Factor	Warning
1	Unknown	10	23.307	650806	16023	7.596	10.346	N/A	7632	3.018	1.103	
2	Unknown	10	26.800	651882	13713	7.608	8.854	N/A	7303	2.649	1.108	
3	Unknown	10	30.277	3643955	69508	42.529	44.879	N/A	7731	4.838	1.128	
4	Unknown	10	37.720	3621427	55633	42.267	35.921	N/A	7807	N/A	1.131	

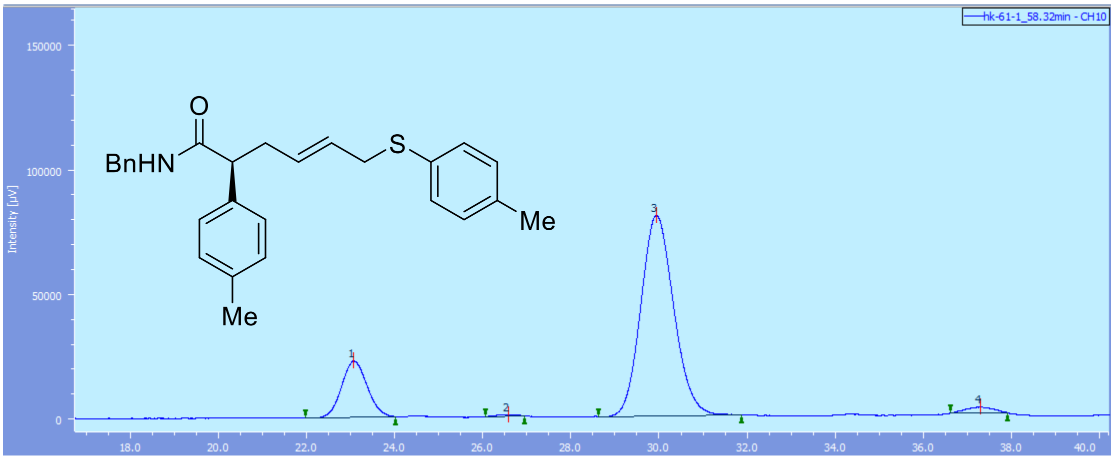

#	Peak Name	CH	tR	Area	Height	Area%	Height%	Quantity	NTP	Resolution	Symmetry Factor	Warning
1	Unknown	10	23.063	905398	22451	17.417	21.204	N/A	7449	3.334	1.092	
2	Unknown	10	26.567	23597	764	0.454	0.722	N/A	10480	2.811	0.838	
3	Unknown	10	29.927	4166737	80321	80.153	75.862	N/A	7756	5.620	1.133	
4	Unknown	10	37.273	102722	2342	1.976	2.212	N/A	13956	N/A	0.970	

(*S*,*E*)-*N*-benzyl-6-((4-methoxyphenyl)thio)-2-(*p*-tolyl)hex-4-enamide (2.33)

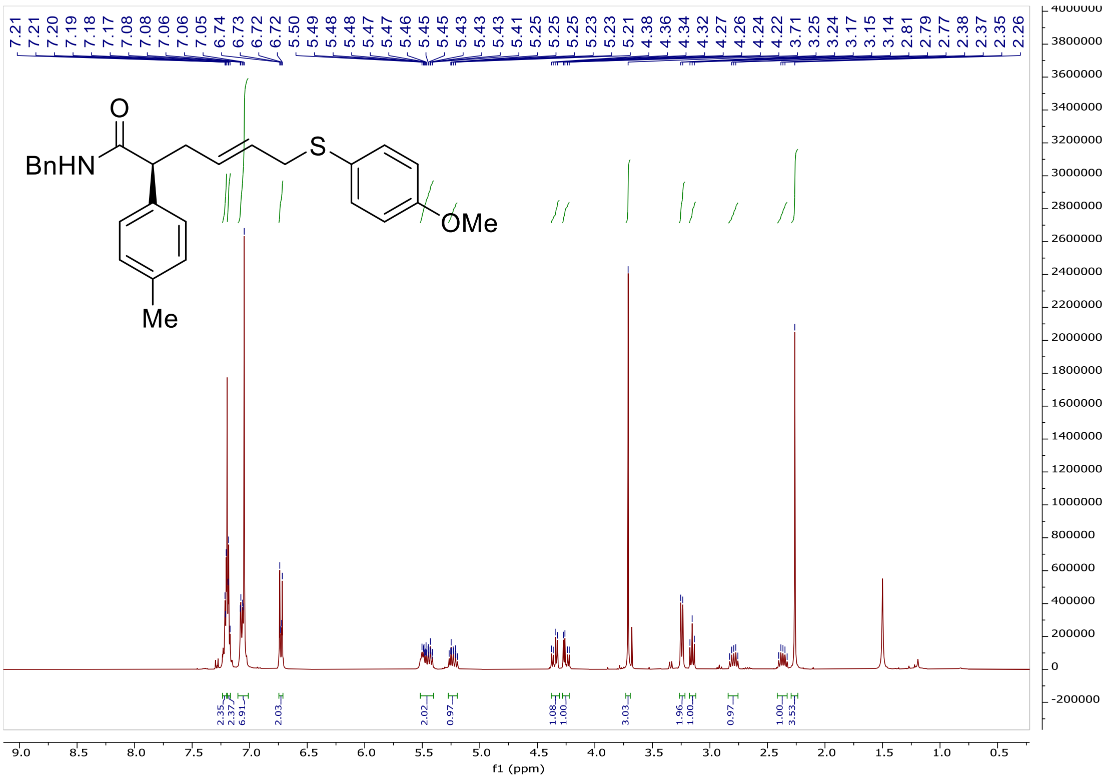

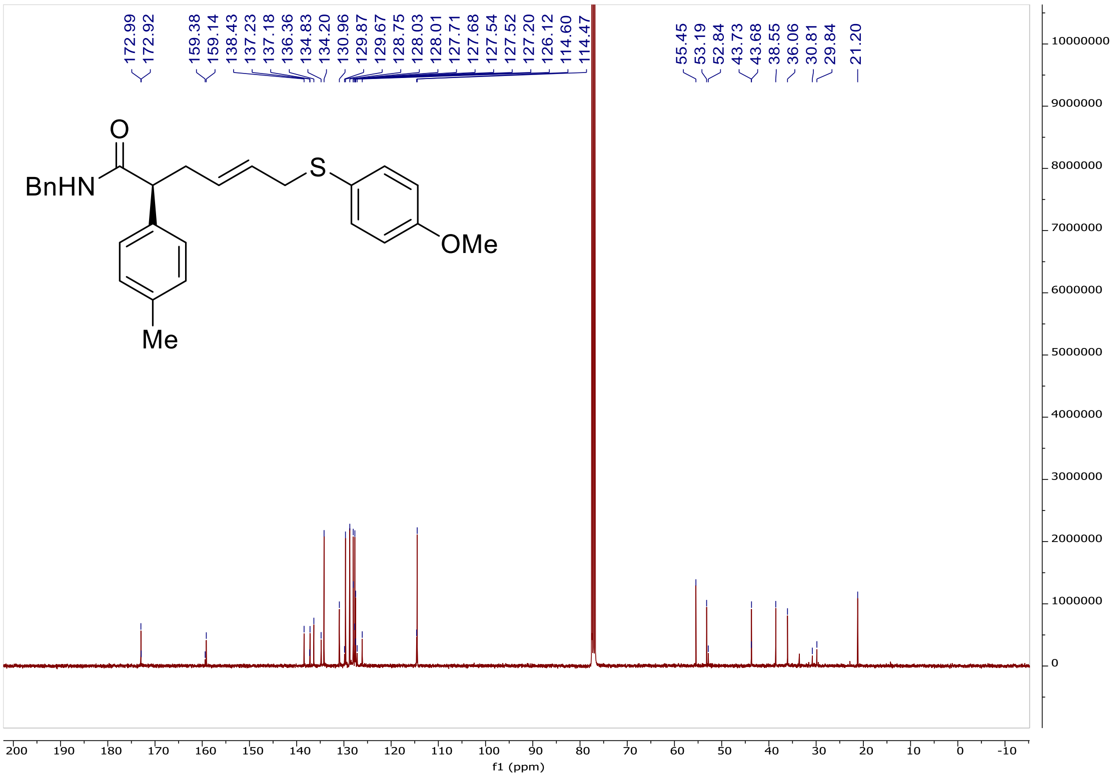

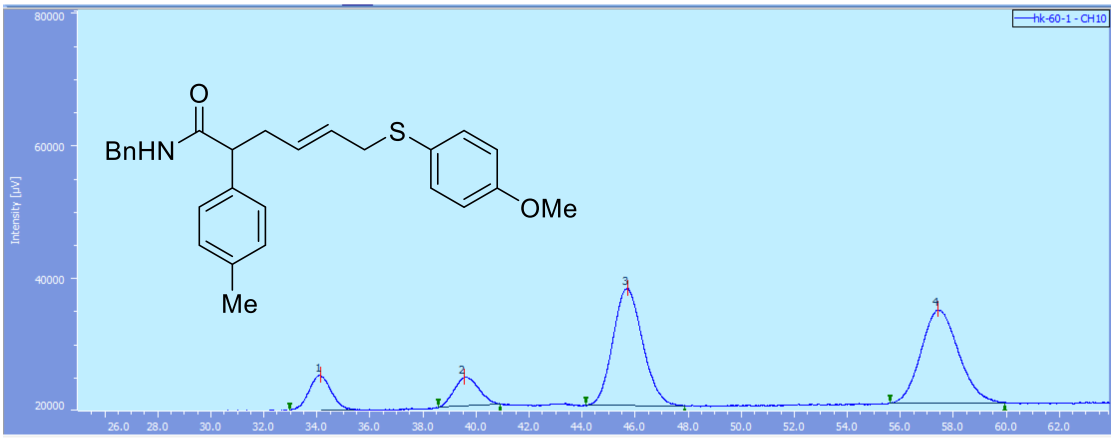

#	Peak Name	CH	tR	Area	Height	Area%	Height%	Quantity	NTP	Resolution	Symmetry Factor	Warning
1	Unknown	10	34.130	308358	5280	9.220	12.772	N/A	7973	3.330	0.946	
2	Unknown	10	39.560	283134	4391	8.466	10.622	N/A	8265	3.244	1.154	
3	Unknown	10	45.713	1376679	17622	41.163	42.624	N/A	7854	5.048	1.099	
4	Unknown	10	57.397	1376320	14049	41.152	33.981	N/A	7931	N/A	1.151	

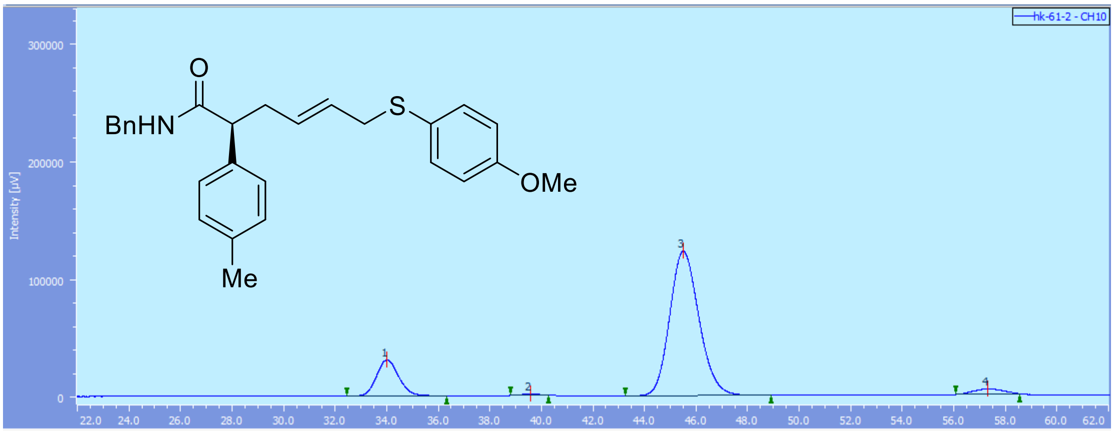

#	Peak Name	CH	tR	Area	Height	Area%	Height%	Quantity	NTP	Resolution	Symmetry Factor	Warning
1	Unknown	10	33.993	1812567	30499	15.291	19.264	N/A	7608	3.677	1.151	
2	Unknown	10	39.553	53436	1062	0.451	0.671	N/A	11526	3.366	0.987	
3	Unknown	10	45.487	9642613	122417	81.344	77.323	N/A	7789	5.471	1.160	
4	Unknown	10	57.283	345543	4341	2.915	2.742	N/A	10260	N/A	0.986	

(*S*)-*N*-Benzyl-2-(*p*-tolyl)-6-tosylhexanamide (3.1)

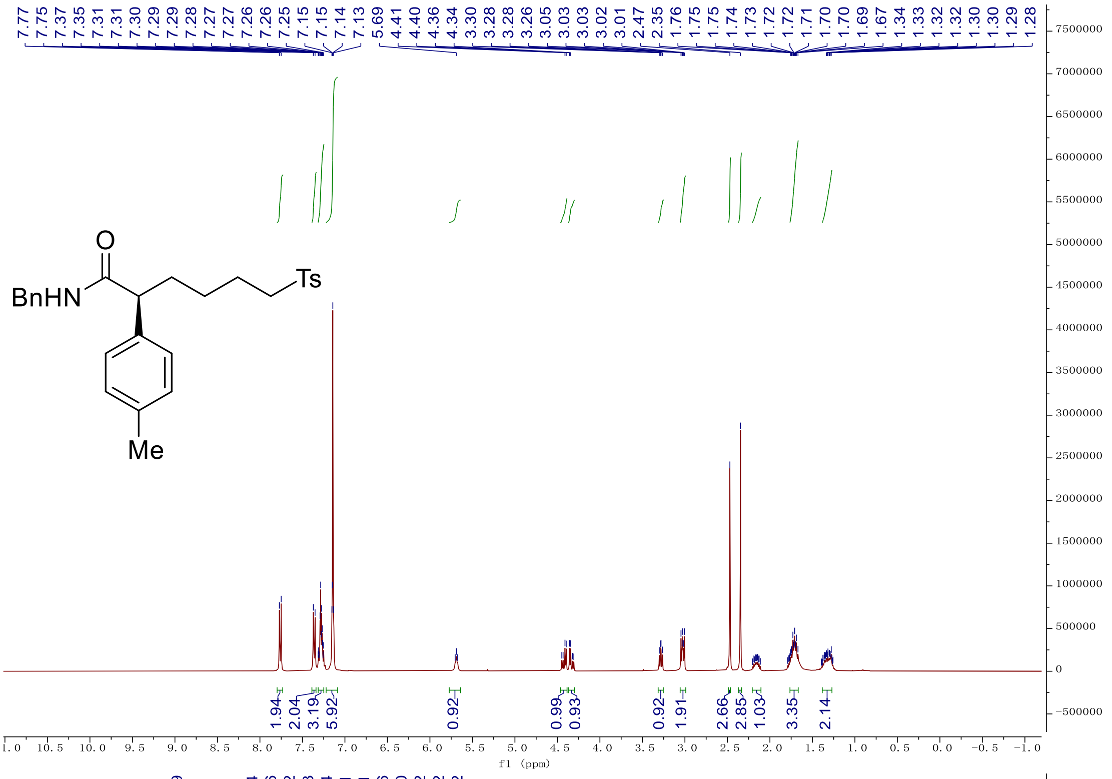

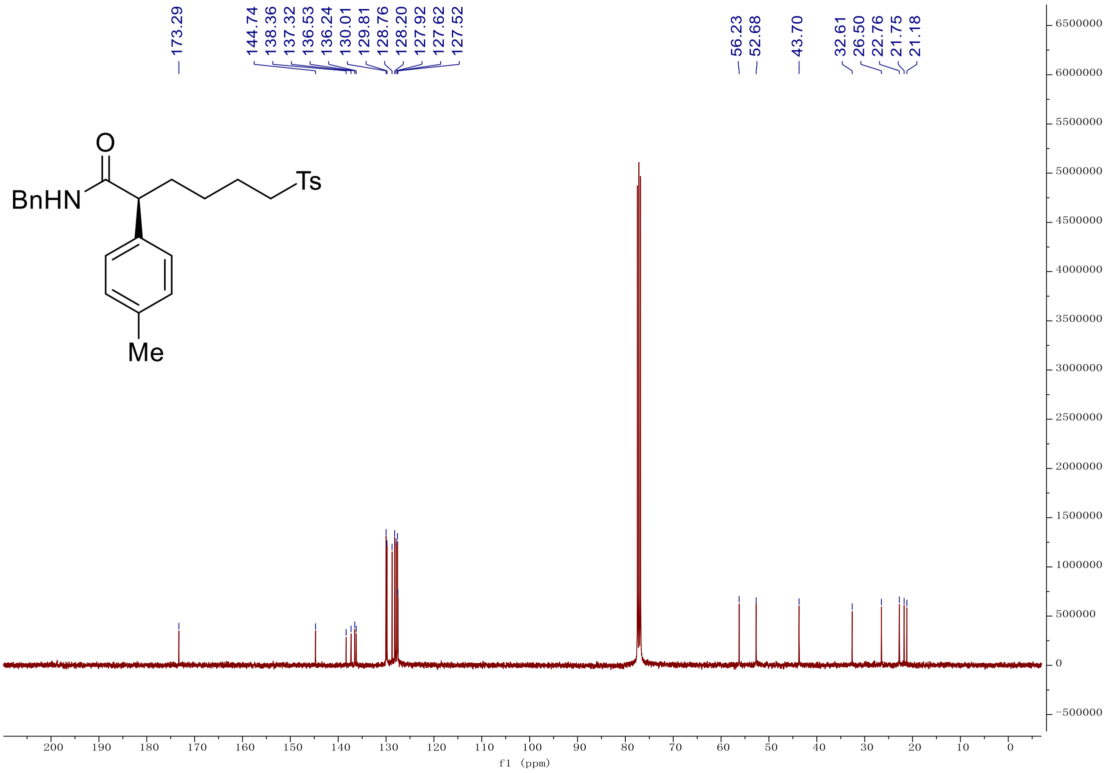

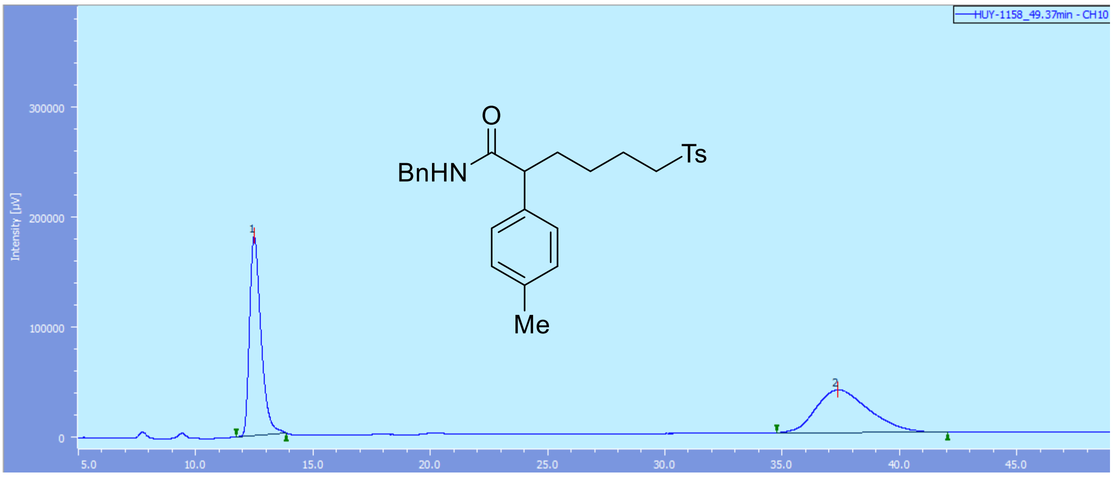

#	Peak Name	CH	tR	Area	Height	Area%	Height%	Quantity	NTP	Resolution	Symmetry Factor	Warning
1	Unknown	10	12.487	6120642	181027	50.073	82.366	N/A	3343	9.913	1.464	
2	Unknown	10	37.350	6102682	38756	49.927	17.634	N/A	1286	N/A	1.292	

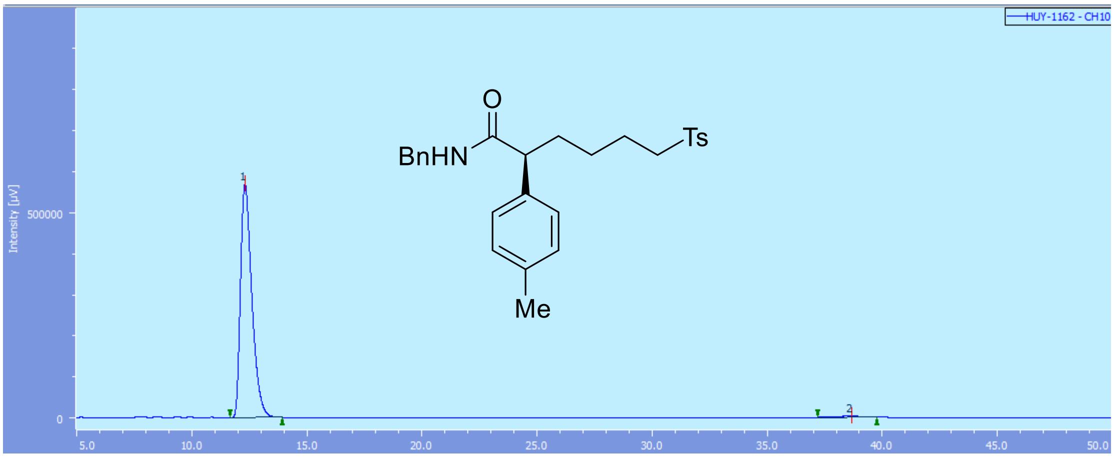

#	Peak Name	CH	tR	Area	Height	Area%	Height%	Quantity	NTP	Resolution	Symmetry Factor	Warning
1	Unknown	10	12.307	19487751	567180	98.532	99.510	N/A	3061	13.642	1.499	
2	Unknown	10	38.650	290428	2793	1.468	0.490	N/A	2686	N/A	1.005	

(3*S*,5*S*)-5-((*S*)-1-Hydroxy-2-tosylethyl)-3-(*p*-tolyl)dihydrofuran-2(3H)-one (*S*,*S*,*S*-3.2)

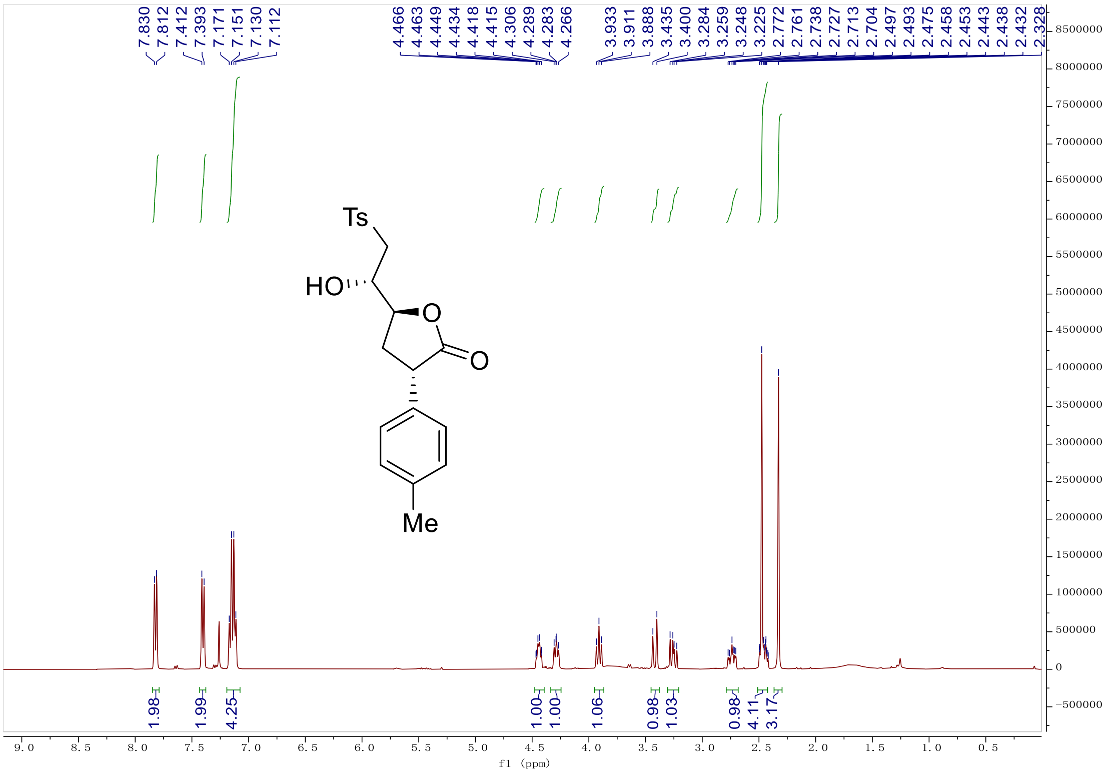

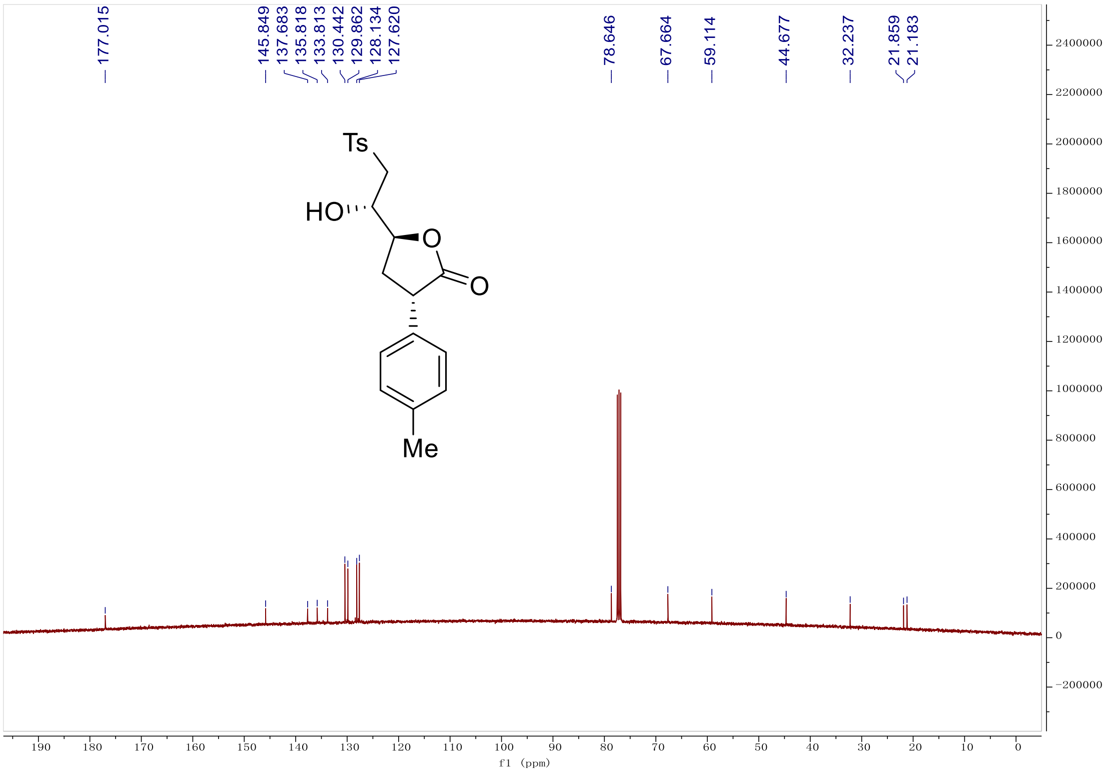

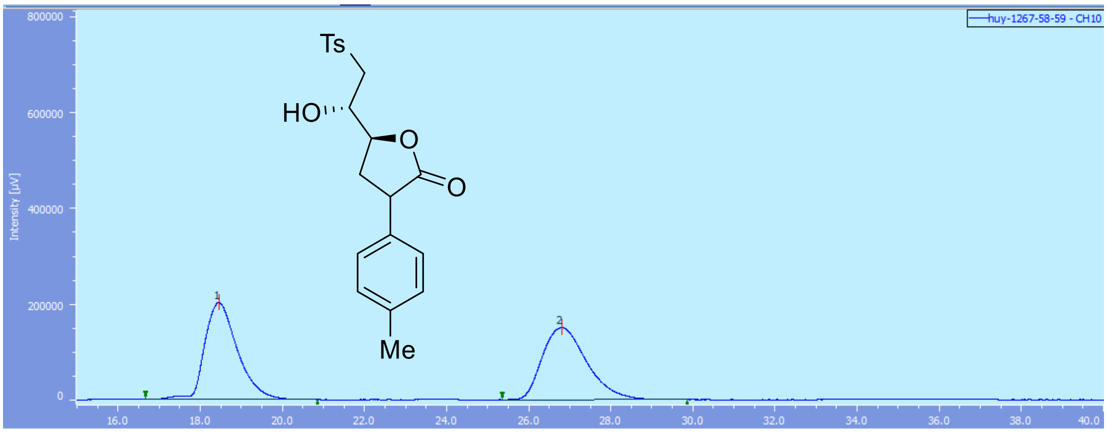

#	Peak Name	CH	tR	Area	Height	Area%	Height%	Quantity	NTP	Resolution	Symmetry Factor	Warning
1	Unknown	10	18.450	11228405	201845	49.809	57.341	N/A	2676	4.906	1.439	
2	Unknown	10	26.790	11314520	150165	50.191	42.659	N/A	2922	N/A	1.370	

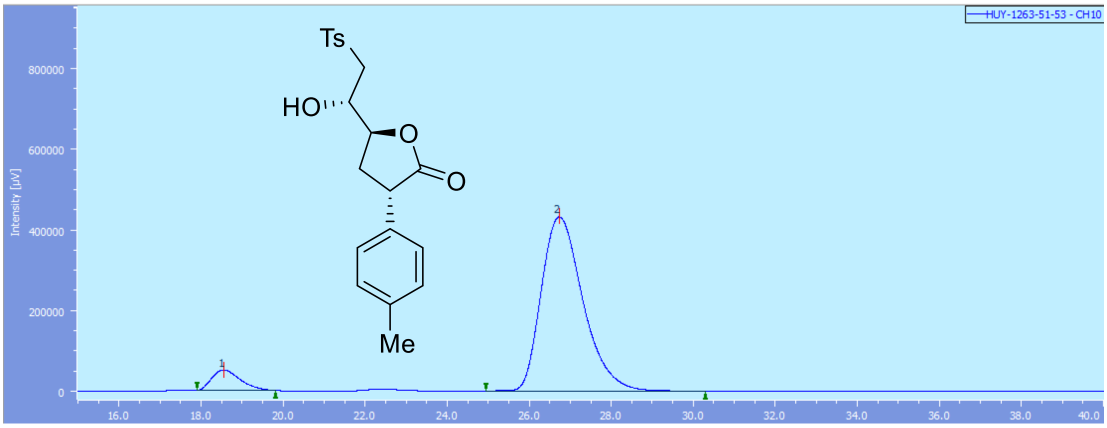

#	Peak Name	CH	tR	Area	Height	Area%	Height%	Quantity	NTP	Resolution	Symmetry Factor	Warning
1	Unknown	10	18.557	2433892	50632	7.436	10.529	N/A	3358	5.258	1.400	
2	Unknown	10	26.730	30298363	430243	92.564	89.471	N/A	3390	N/A	1.336	

(3*S*,5*R*)-5-((*R*)-1-Hydroxy-2-tosylethyl)-3-(*p*-tolyl)dihydrofuran-2(3H)-one (*S*,*R*,*R*-3.2)

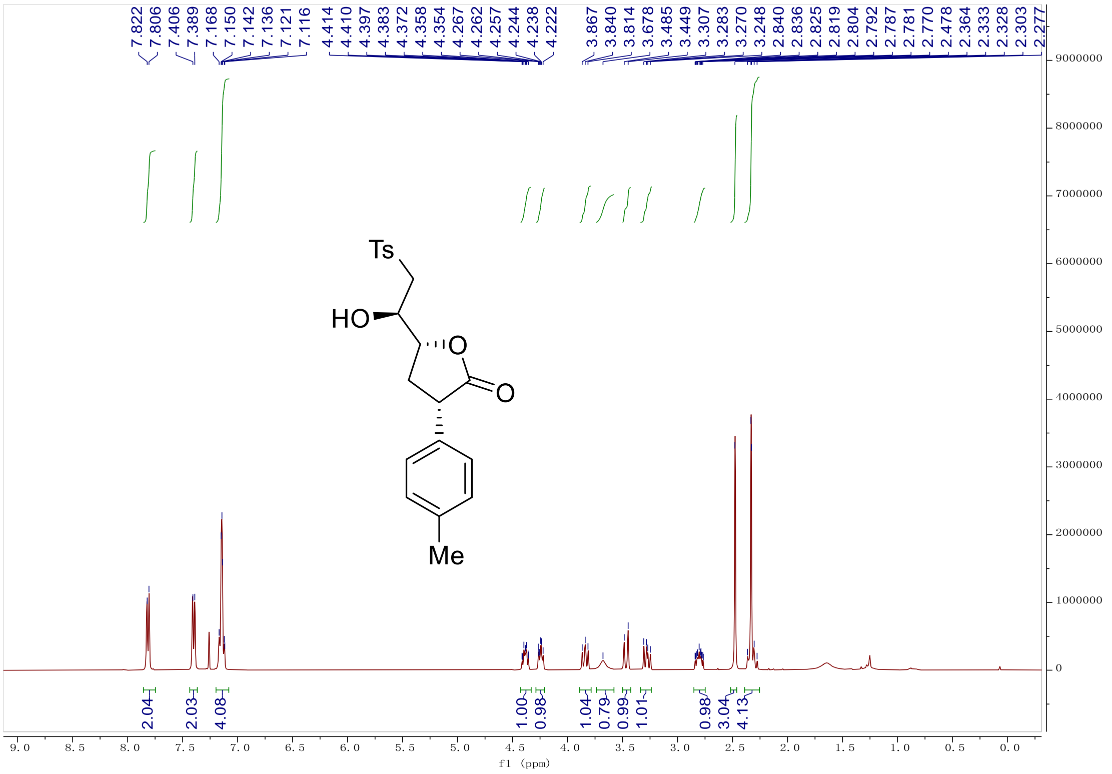

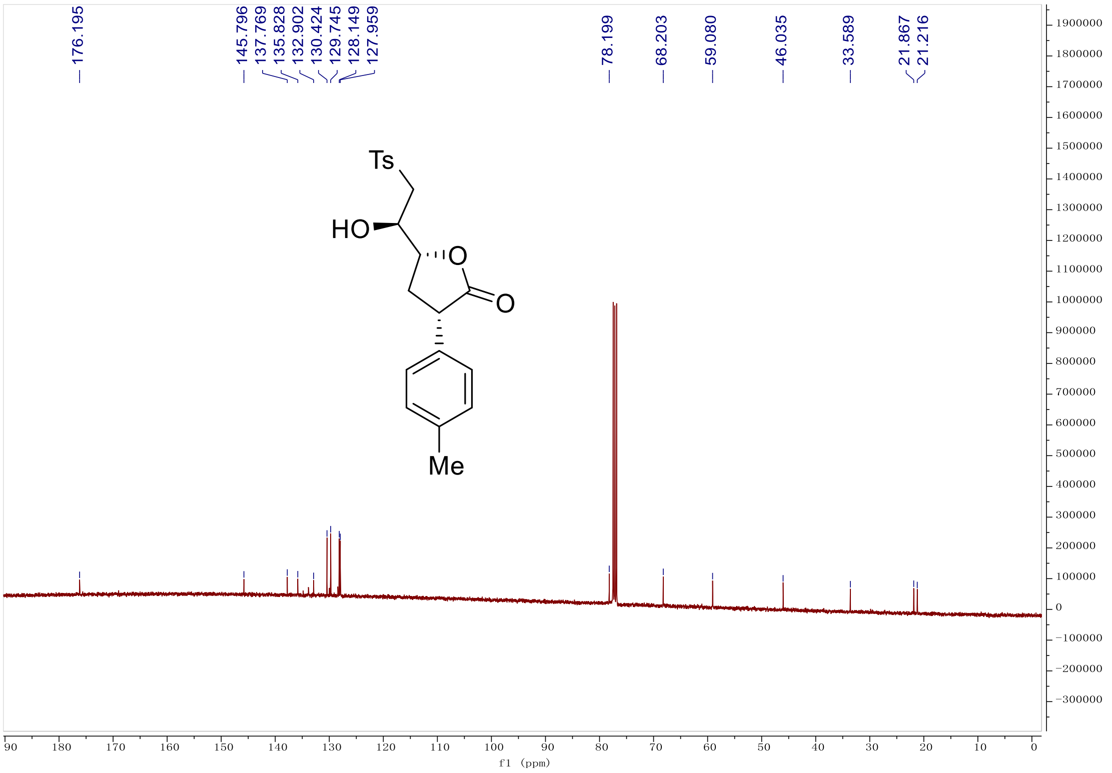

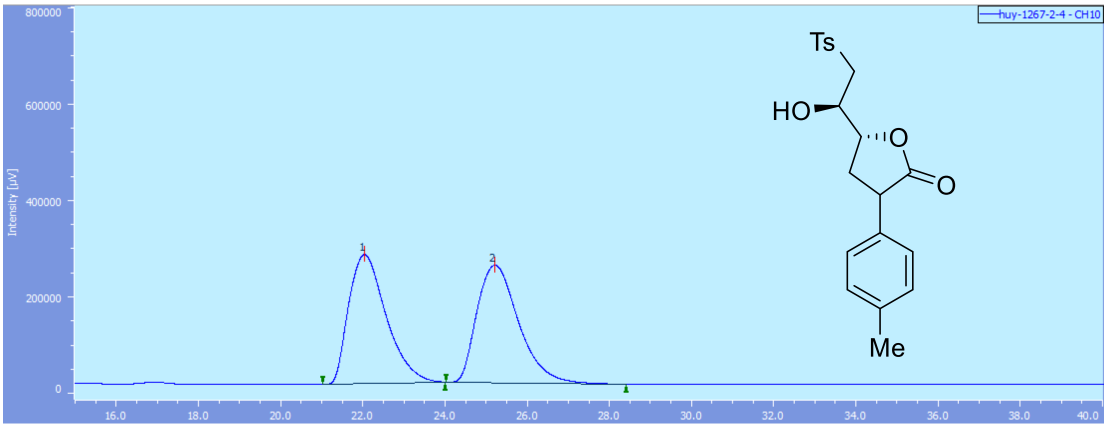

#	Peak Name	CH	tR	Area	Height	Area%	Height%	Quantity	NTP	Resolution	Symmetry Factor	Warning
1	Unknown	10	22.033	16921256	267498	49.655	52.371	N/A	2744	1.800	1.459	
2	Unknown	10	25.210	17156185	243274	50.345	47.629	N/A	2947	N/A	1.405	

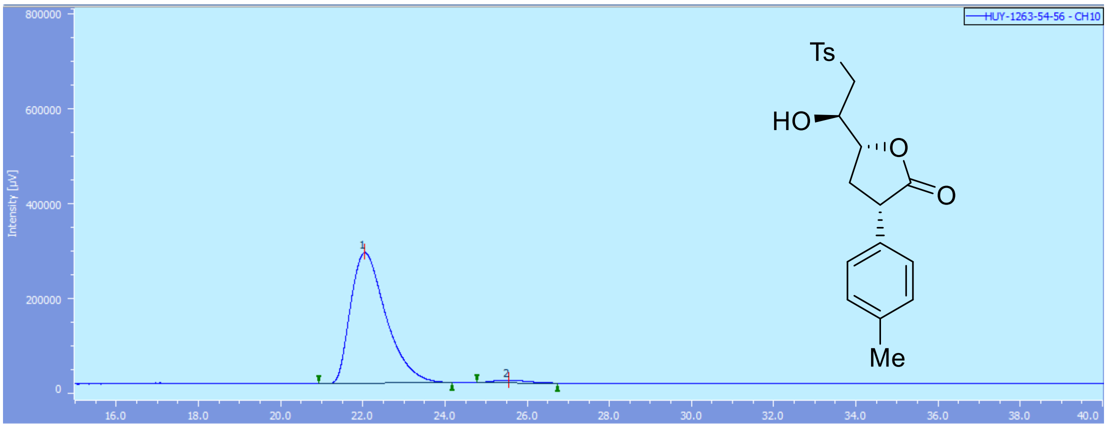

#	Peak Name	CH	tR	Area	Height	Area%	Height%	Quantity	NTP	Resolution	Symmetry Factor	Warning
1	Unknown	10	22.047	16404515	275159	98.175	98.022	N/A	3187	2.264	1.510	
2	Unknown	10	25.533	304915	5551	1.825	1.978	N/A	4481	N/A	1.218	

(*S*,*E*)-2-(*p*-Tolyl)-6-tosylhexanamide (3.3)

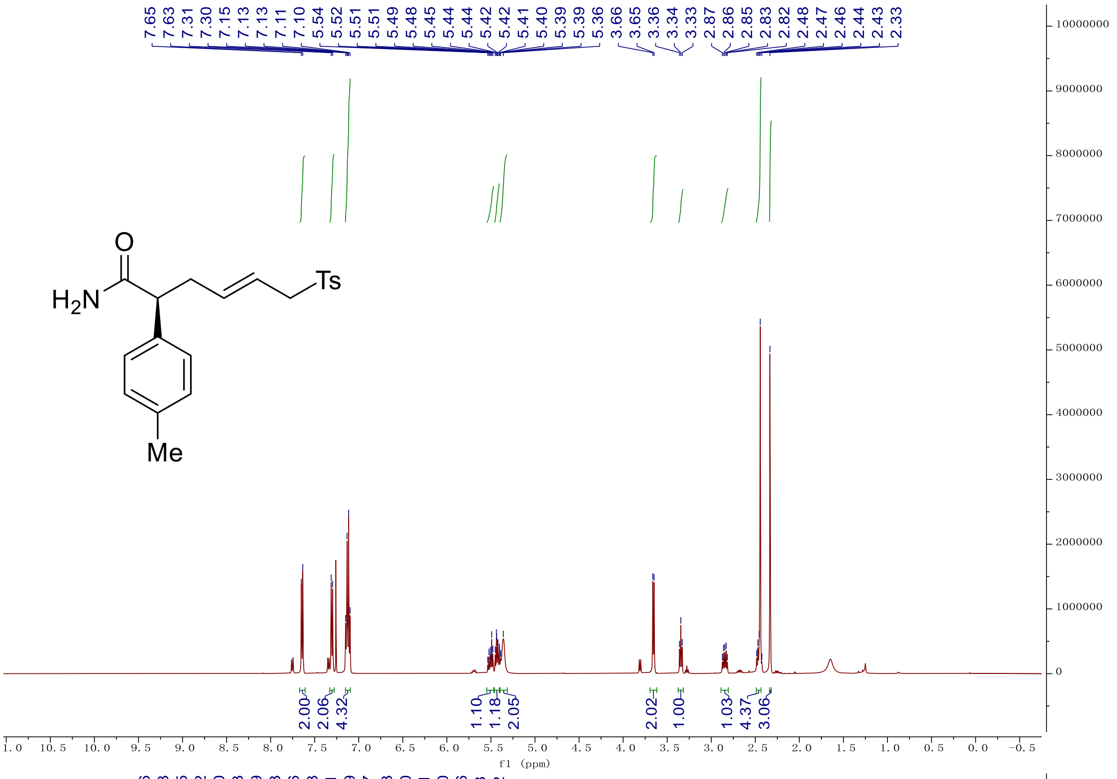

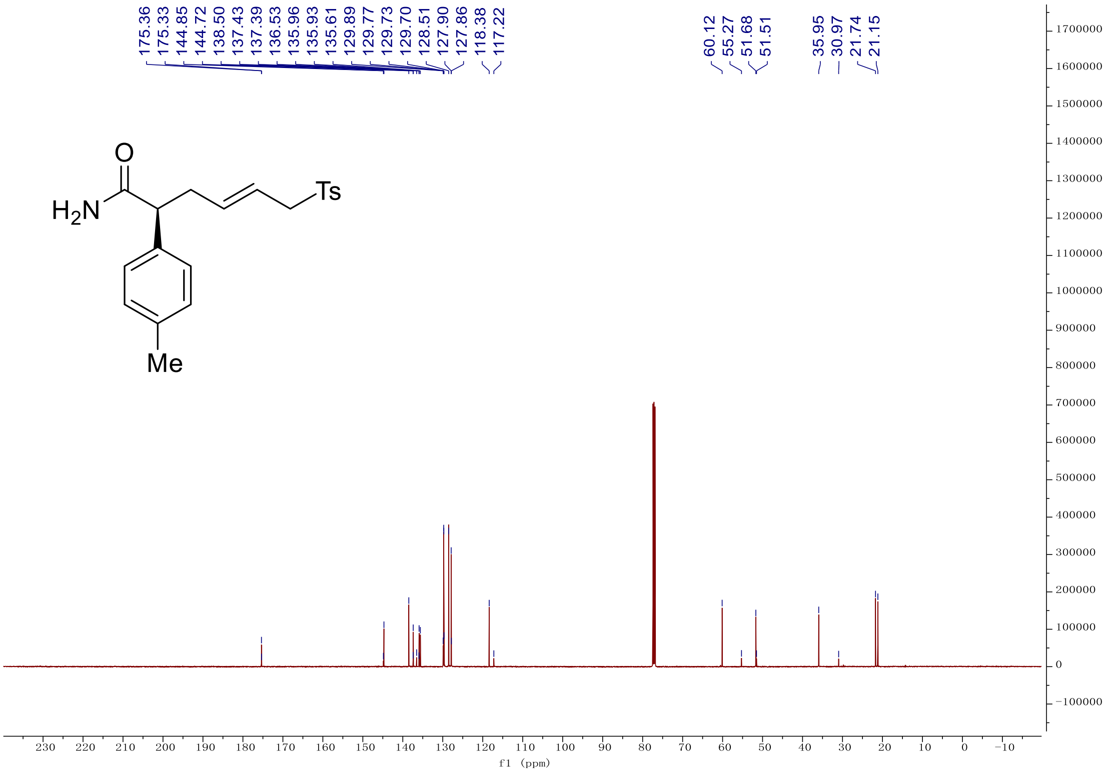

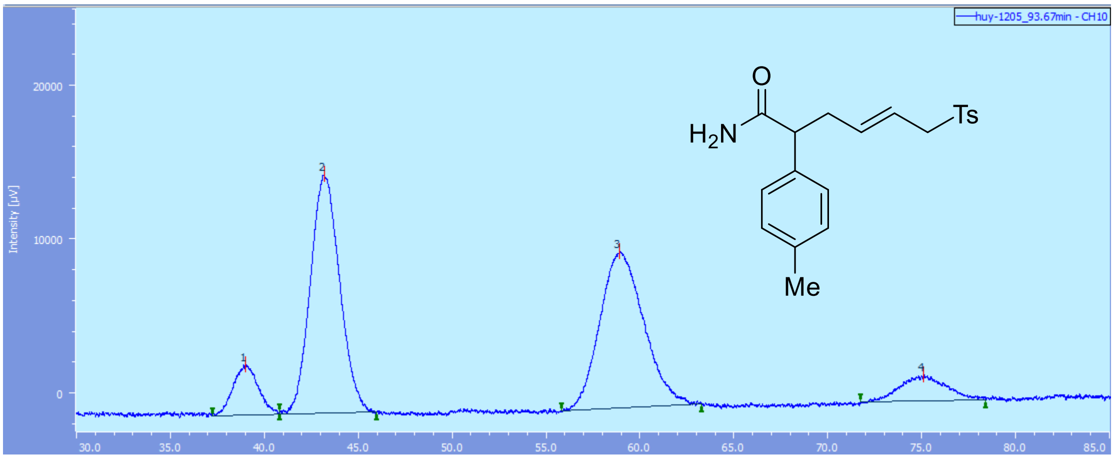

#	Peak Name	CH	tR	Area	Height	Area%	Height%	Quantity	NTP	Resolution	Symmetry Factor	Warning
1	Unknown	10	39.003	303316	3283	7.764	10.723	N/A	3984	1.571	0.982	
2	Unknown	10	43.180	1679258	15499	42.984	50.627	N/A	3649	4.403	1.118	
3	Unknown	10	58.903	1644420	10146	42.092	33.140	N/A	2999	3.620	1.214	
4	Unknown	10	75.077	279717	1687	7.160	5.510	N/A	4159	N/A	1.063	

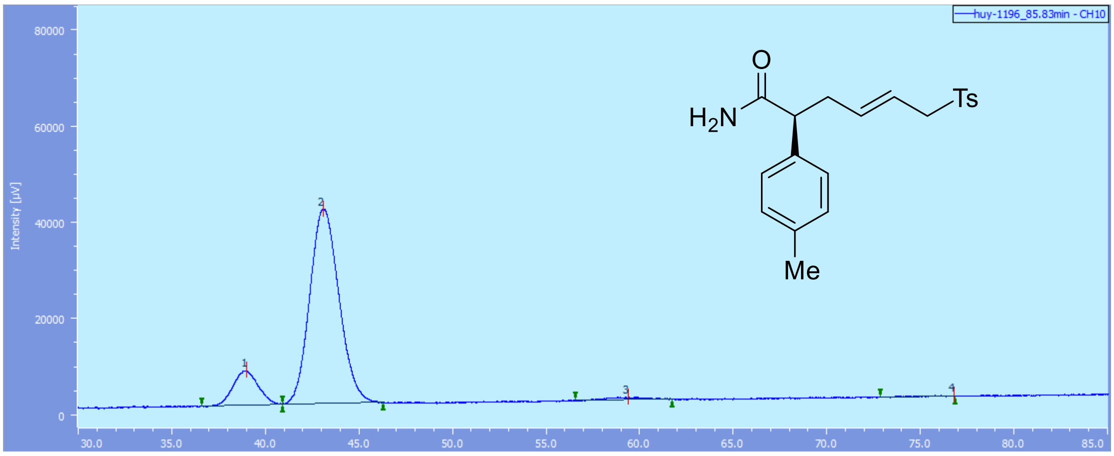

#	Peak Name	CH	tR	Area	Height	Area%	Height%	Quantity	NTP	Resolution	Symmetry Factor	Warning
1	Unknown	10	38.977	667992	7191	13.025	14.908	N/A	3941	1.549	1.004	
2	Unknown	10	43.110	4381209	40387	85.429	83.727	N/A	3620	4.627	1.124	
3	Unknown	10	59.380	63976	527	1.247	1.092	N/A	3220	3.227	0.965	
4	Unknown	10	76.753	15280	132	0.298	0.273	N/A	2157	N/A	0.505	

(S,*E*)-*N*-Benzyl-3-(2-tosylethylidene)-2,3-dihydro-1H-indene-1-carboxamide (3.4)

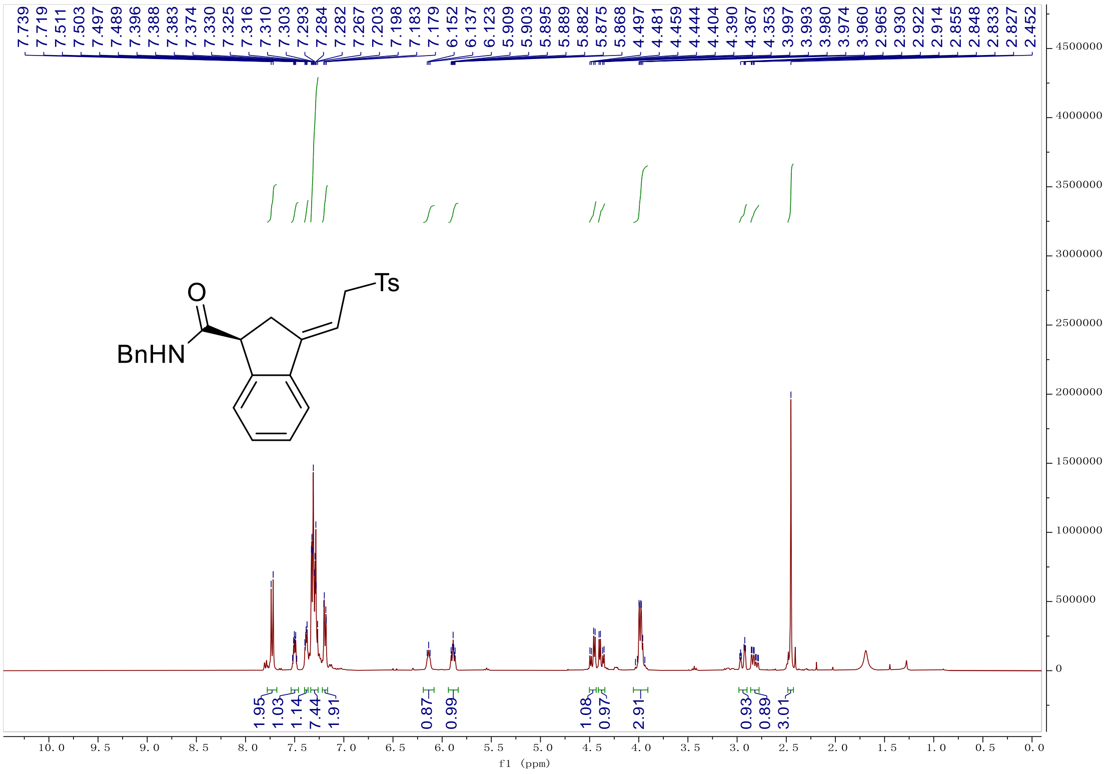

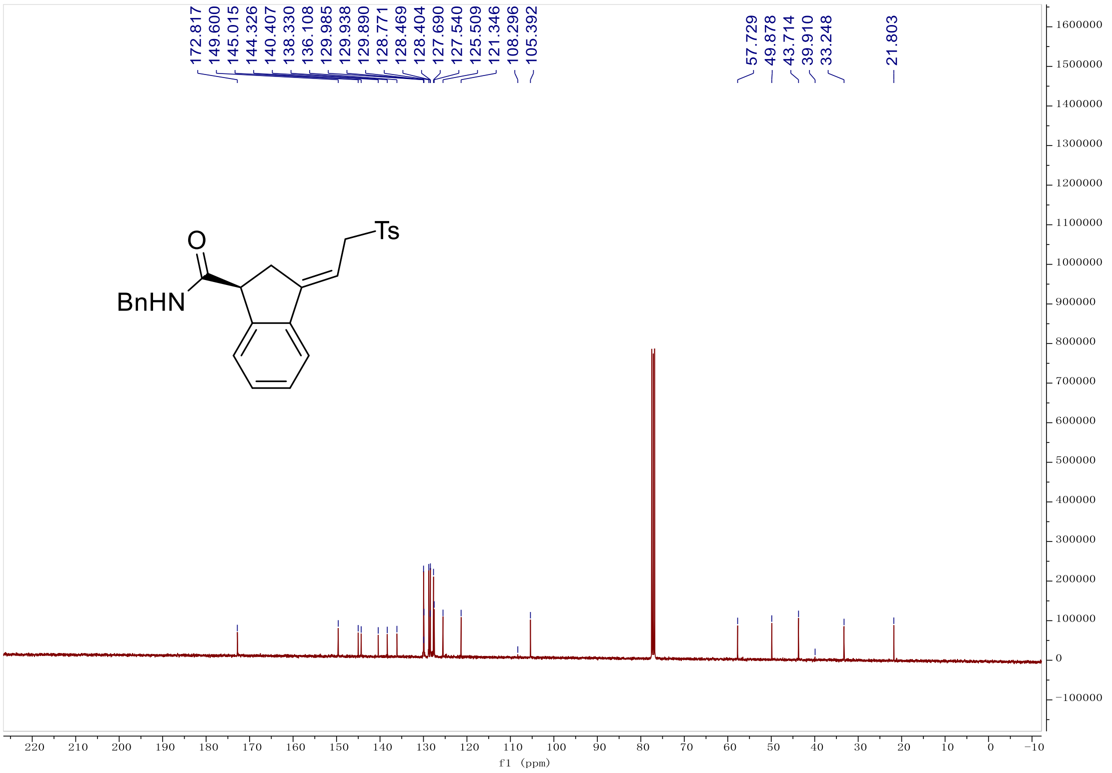

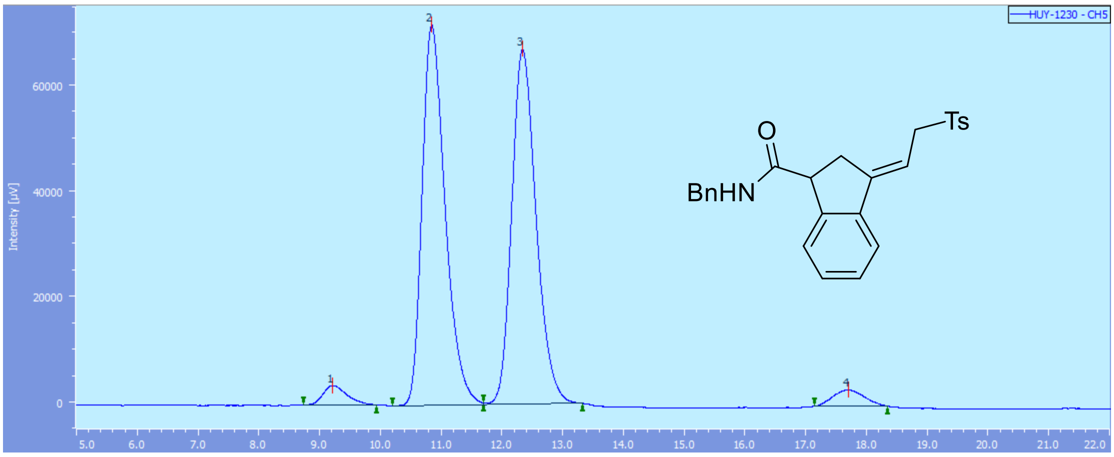

#	Peak Name	CH	tR	Area	Height	Area%	Height%	Quantity	NTP	Resolution	Symmetry Factor	Warning
1	Unknown	5	9.210	100652	3678	2.537	2.526	N/A	2668	2.348	1.338	
2	Unknown	5	10.840	1889919	71785	47.643	49.315	N/A	4077	2.139	1.373	
3	Unknown	5	12.333	1867101	67025	47.068	46.045	N/A	4679	6.292	1.241	
4	Unknown	5	17.687	109129	3076	2.751	2.113	N/A	5160	N/A	1.092	

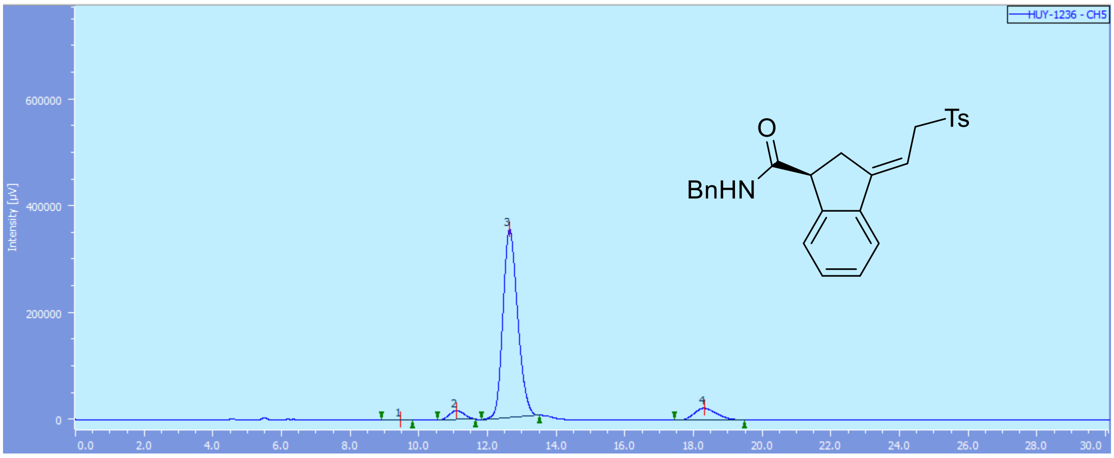

#	Peak Name	CH	tR	Area	Height	Area%	Height%	Quantity	NTP	Resolution	Symmetry Factor	Warning
1	Unknown	5	9.443	10024	535	0.084	0.137	N/A	4587	2.406	1.383	
2	Unknown	5	11.097	503234	16783	4.197	4.297	N/A	2926	1.924	1.052	
3	Unknown	5	12.633	10481388	351288	87.416	89.943	N/A	4182	5.711	1.192	
4	Unknown	5	18.290	995545	21962	8.303	5.623	N/A	3687	N/A	1.244	

8. References

(1) G. Zhao, A. Khosravi, S. Sharma, D. G. Musaev, Y. Ngai, *J. Am. Chem. Soc.* **2024**, *146*, 31391-31399.
(2) G. B. Payne, *J. Org. Chem.* **1967**, *32*, 3351-3355.
(3) X. Du, M. E. Lennon, G. Kriticou, C. Nevado, *Nat. Commun.* **2025**, *16*, 6958.
(4) C. Hervieu, M. S. Kirillova, T. Suárez, M. Müller, E. Merino, C. Nevado, *Nat. Chem.* **2021**, *13*, 327-334.
(5) Z. Liu, C. Xu, J. del Pozo, S. Torker, A. H. Hoveyda, *J. Am. Chem. Soc.* **2019**, *141*, 7137-7146.
(6) V. A. Ignatenko, N. Deligonul, R. Viswanathan, *Org. Lett.* **2010**, *12*, 3594-3597.
